# Transcriptional profile and immune infiltration in colorectal cancer reveal the significance of inducible T‐cell costimulator as a crucial immune checkpoint molecule

**DOI:** 10.1002/cam4.7097

**Published:** 2024-03-20

**Authors:** Jian Chu, Yinghang Wu, Zhanbo Qu, Jing Zhuang, Jiang Liu, Shugao Han, Wei Wu, Shuwen Han

**Affiliations:** ^1^ Huzhou Central Hospital Affiliated Central Hospital Huzhou University Huzhou China; ^2^ Fifth School of Clinical Medicine of Zhejiang Chinese Medical University (Huzhou Central Hospital) Huzhou China; ^3^ Key Laboratory of Multiomics Research and Clinical Transformation of Digestive Cancer of Huzhou Huzhou China; ^4^ Second Affiliated Hospital of School of Medicine Zhejiang University Hangzhou China

**Keywords:** ceRNA network, CRC, ICOS, immune checkpoint, immune infiltration, integration analysis

## Abstract

**Background:**

Emergence of novel immuno‐therapeutics has shown promising improvement in the clinical outcome of colorectal cancer (CRC).

**Objective:**

To identify robust immune checkpoints based on expression and immune infiltration profiles of clinical CRC samples.

**Methods:**

One dataset from The Cancer Genome Atlas database and two from Gene Expression Omnibus were independently employed for the analysis. Genes associated with overall survival were identified, and distribution of each immune checkpoint with respect to different clinical features was determined to explore key immune checkpoints. Multiple staining methods were used to verify the correlation between key immune checkpoint ICOS and clinical pathological features. Differentially expressed mRNA and long non‐coding RNA (lncRNA) were then detected for gene set enrichment analysis and gene set variation analysis to investigate the differentially enriched biological processes between low‐ and high‐expression groups. Significant immune‐related mRNAs and lncRNA were subjected to competing endogenous RNA (ceRNA) network analysis. Correlation of inducible T‐cell costimulator (ICOS) and top 10 genes in ceRNA network were further considered for validation.

**Results:**

ICOS was identified from 14 immune checkpoints as the most highly correlated gene with survival and clinical features in CRC. The expression of ICOS protein in the poorly differentiated group was lower than that in the moderately differentiated group, and the expression in different pathological stages was significant. In addition, the expressions of ICOS were negatively correlated with Ki67. A conspicuous number of immune‐related pathways were enriched in differentially expressed genes in the ICOS high‐ and low‐expression groups. Integration with immune infiltration data revealed a multitude of differentially expressed immune‐related genes enriched for ceRNA network. Furthermore, expression of top 10 genes investigated from ceRNA network showed high correlation with ICOS.

**Conclusion:**

ICOS might serve as a robust immune checkpoint for prognosis with several genes being potential targets of ICOS‐directed immunotherapy in CRC.

## 1 INTRODUCTION

As one of the most common cancers in the world, adenocarcinomas of the colon and rectum in the cells of the inner lining of the large intestine are the second most common cause of cancer death in the USA.^1^ A large number of studies have validated that the immune system exerts negative selection pressure on the tumor and influences the tumor microenvironment of colorectal cancer (CRC).^2^ Immune checkpoint blocking is an important part of immunotherapy that has unique advantages and great potential to provide a new way for tumor treatment. Following initial successes in melanoma treatment, the emergence of immunotherapy, which targets several negative regulators of T‐cell activation, has changed the therapeutic landscape of multiple types of solid cancers, including lung cancer, gastro‐esophageal cancer, and CRC.^3^, ^4^, ^5^ For instance, it has been proved that immune checkpoint inhibitors (ICIs) that target programmed cell death 1 (PD‐1), cytotoxic T lymphocyte antigen 4 (CTLA4), or programmed cell death 1 ligand 1 (PDL1) benefit metastatic CRC patients with mismatch repair‐deficient and microsatellite instability high tumors.^6^ In addition, in early clinical studies, it was found that blocking the CTLA‐4 pathway can prolong T‐cell response time, destroy cancer cells, and mediate T‐cell invasion of tumor tissue, thus leading to tumor regression and prolonged survival, while PDL1 was found to be highly expressed in a variety of tumor models, and blocking this pathway could also effectively enhance the killing effect of T cells and mediate tumor regression.^7^ However, immune checkpoint therapy has some limitations and challenges. For example, patients with primary resistance do not benefit from immunotherapy, and cancer progression may occur several months after treatment for some initial responders.^8^ Not only that the lack of reactive biomarkers and immune‐related toxicity of immune checkpoint inhibitors further limit their application in tumor therapy. Therefore, the discovery of new biomarkers will help accurately monitor the efficacy and toxicity of treatment, so that more patients can benefit from immunotherapy.

Furthermore, evidence has shown that infiltrating immune cell data exhibits better metastases and survival prediction in CRC and in other kinds of cancer. Isella et al. found that CRC patients with the dry/serrated/interstitial (SSM) transcription subtype had a poor prognosis and that the unique transcriptional and clinical features of the SSM subtype were largely attributable to its particularly rich stromal components.^9^ van den Eynde et al. discovered that the immunophenotype of the least invasive metastases was more associated with patient prognosis than other metastases.^10^ Lu et al. used machine learning strategies to construct a 24‐gene RNA marker from 395 immune‐related gene expression profiles that reflects the “immune response properties” of cancer cells and the immune microenvironment. The results suggested that a predictive model based on IO scores has better predictive and prognostic value in both the discovery and validation groups, which may help personalize the management of immunotherapy in patients with metastatic gastrointestinal cancer.^11^ Moreover, several newly discovered immune checkpoint molecules have shown promising features in preclinical data,^12^, ^13^, ^14^, ^15^, ^16^ such as LAG3, HAVCR2, TIGIT, and stimulating T‐cell co‐stimulatory receptors, AILIM or ICOS, TNFRSF4, and TNFRSF9. These immune checkpoints have also been shown to improve the effectiveness of immunotherapy.

Here, 14 immune checkpoints (including PDCD1, CTLA4, PDL1, and ICOS) that are more common in clinical studies were selected, prognostic immune checkpoints were identified, and their correlation with clinical features was analyzed to explore key immune checkpoints. Subsequently, differentially expressed mRNA and lncRNA were analyzed for gene set enrichment and gene set variation, and the immune infiltration was evaluated. In addition, ceRNA network analysis was performed based on important immune‐associated mrnas and lncRNAs. Considering the correlation of key immune checkpoints with the top 10 genes in the ceRNA network, this was validated through a public dataset. This study indicated that ICOS is a relevant immune checkpoint for the prognosis of CRC and is expected to be a monitoring target for the prognosis of CRC.

## 2 MATERIALS AND METHODS

### 2.1 Selection of immune checkpoints

A total of 14 immune checkpoints, including PDCD1, CTLA4, PDL1, LAG3, HAVCR2, TIGIT, ICOS, TNFRSF4, TNFRSF9, CD73, CD47, BTLA, SIRPA, and VTCN1, were selected for the study. The workflow of this study is shown in Figure S1.

### 2.2 Data collection for RNA‐sequencing profile and clinical features of CRC patients

The Cancer Genome Atlas (TCGA) and Gene Expression Omnibus^17^ (GEO) database were utilized to collect RNA‐sequencing (RNA‐seq) data for CRC samples. TCGA‐COADREAD (Colorectal Adenocarcinoma) cohort that contains normalized RNA‐seq data (log_2_(tpm + 0.001)) of 377 samples and shows “Rectum Adenocarcinoma” or “Colon Adenocarcinoma” in “detailed_category” was downloaded using UCSC‐Xena^18^ (https://toil.xenahubs.net). Clinical data from a total of 377 CRC patients, including gender, age, tumor location, tumor stage, TNM stage, overall survival (OS), and progression free survival (PFS) information, were also obtained from the same platform.

Further, 156 and 124 CRC samples from GSE103479 and GSE72970, respectively, were downloaded using GEO database (http://www.ncbi.nlm.nih.gov/geo/). Clinical data (GSE103479: gender, age, perineural invasion, extensive vascular invasion [EVI], lymphovascular invasion [LVI], tumor size, tumor location, T stage, N stage, recurrence, response status, CMS subtype, CRIS subtype, OS and PFS information. GSE72970: gender, age, tumor location, metastases, T stage, N stage, response status, OS and PFS information) of the two independent studies were also acquired from GEO.

### 2.3 Data preprocessing

For TCGA‐COADREAD, 60,498 Ensemble genes IDs obtained from GENCODE database^19^ (https://www.gencodegenes.org/, hg38) were converted to gene symbols. mRNA list with genes annotated as “protein coding,” and lncRNA list with genes annotated as “non_coding,” “lincRNA,” “antisense,” “sense_intronic,” “sense_overlapping,” “processed_transcript,” and “3 prime_overlapping_ncRNA” were constructed.

For datasets GSE103479 and GSE72970, array expression matrix files were downloaded directly and probes mapped to gene symbols were kept for gene expression. Average expression value for a gene was calculated from multiple probes corresponding to the same gene.

### 2.4 Detection of survival‐associated genes of 14 immune checkpoints

Prognostic genes that closely correlated with OS and PFS of CRC patients in TCGA‐COADREAD, GSE103479, and GSE72970 cohorts were distinguished by Kaplan–Meier analysis and log‐rank analysis using “survival” package (version: 2.42‐6) separately. Optimal threshold was determined with “survminer” package (version: 0.4.3) that considers gene expression, survival, and living status together. For each immune checkpoint, samples were classified into two groups according to specific gene's median expression or the determined optimal threshold. Genes significantly correlated with prognosis (*p*‐value <0.05) were obtained for downstream analysis.

### 2.5 Association analysis of immune checkpoints and clinical features

For each cohort, samples were divided into different groups according to different clinical features. Subsequent distribution and statistical analyses of each checkpoint were performed with “ggstatsplot” package (version: 0.6.5). In detail, Welch's *t*‐test was used for comparison of two groups and Welch's one‐way ANOVA was used for cases with multiple groups.

### 2.6 Validation of the correlation between key molecules and pathological features by clinical samples

#### 2.6.1 1 Subjects

A total of 30 CRC patients admitted to Huzhou Central Hospital from January 2021 to December 2021 were enrolled. Basic information and indicators regarding the retrospective study were obtained from the medical record management system of Huzhou Central Hospital and health records system of the Physical Examination Center. The clinical protocols involving the patients and the informed consent form were approved by the Chinese Clinical Trial Registry (http://www.chictr.org.cn, No. ChiCTR2200061039) and Ethics Committee of Huzhou Central Hospital (No. 202110018‐02). The inclusion criteria are as follows: (1) Patients were confirmed by a pathologic diagnosis, and the patients volunteered to participate in the study and signed the informed consent and (2) The clinical stages conformed to the criteria of stage II–IV CRC according to AJCC. On the other hand, the exclusion criteria are as follows: (1) Patients with other intestinal diseases, such as ulcerative colitis and Crohn's disease; (2) patients with another primary cancer; and (3) patients with known primary organ failure. All pathological specimens were obtained from pathological examination or surgical resection. The basic clinical information of the CRC patients is shown in Table 1.

**TABLE 1:** Basic clinical information of colorectal cancer patients.

Cases, *n*	30
Males, *n*	11
Age, years	59.27 ± 11.31
Current smoking, *n*	9
Current drinking, *n*	14
Known diabetes, *n*	7
Known hypertension, *n*	12
Stage I, *n*	4
Stage II, *n*	10
Stage III, *n*	9
Stage IV, *n*	7

#### 2.6.2 Hematoxylin–eosin (HE) staining

The slices were dewaxed and hydrated following conventional methods. The PBS cleaning solution was obtained from China Jiangsu KGB5001 Biotechnology Co., Ltd. The operation followed the instructions of the hematoxylin–eosin dye kit (Jiangsu KGA224 Biotechnology Co., Ltd., China). The slices were dried, sealed, and scanned with a digital pathology scanner.

#### 2.6.3 Immunofluorescence (IF) detection of ICOS protein

The sections were pretreated according to conventional methods, and then, antigen repair was performed according to the procedure. Rabbit Anti‐ICos (ABCAM AB215715) and TRITC Sheep anti‐Rabbit (Jackson ImmunoResearch 111‐025‐003, USA) were used in this experiment. Two drops of 3% of H_2_O_2_‐methanol solution were added to each section, blocked at room temperature (15–25°C) for 10 min, and immersed in PBS (Jiangsu KGB5001, China) for 3 times. About 1% of BSA 50–100 μL was added to block. Afterwards, the antigen–antibody reaction and the primary and secondary antibody reaction were performed. Next, the sections were soaked again with PBS 3 times, and each slide was counterstained by dropping 50–100 μL of DAPI dye solution (KGA215, Jiangsu KGI Biotechnology Co., LTD., China). Finally, the slides were sealed with anti‐extraction glue and scanned with a digital pathological section scanner (Olympus VS200, Japan).

#### 2.6.4 Immunohistochemical (IHC) staining the Ki67 proliferation index

The samples were treated according to the instructions of an EliVision Plus Kit (Fuzhou Mai Xin Biotechnology Co., LTD kit‐9902, China), and Ki67 was obtained from Abcam AB92742, UK. After the sections were processed, the protein expression in tissue cells was observed under an optical microscope. Three expression regions were selected and photographed for preservation with a bioluminescence microscope (Olympus BX43, Japan).

### 2.7 Identification of differentially expressed mRNAs and lncRNAs

In TCGA‐COADREAD cohort, 377 patients were grouped into high‐ and low‐ICOS expression groups. The “limma” package (version: 3.10.3)^20^ was applied to identify differentially expressed mRNA and lncRNA in the two groups independently. The *p*‐value from “limma” package was calculated by “Benjamini & Hochberg (BH)” method to get adjusted *p*‐value. mRNAs and lncRNAs that show adjusted *p*‐value <0.05 and absolute log (fold change) > 2 (4 fold change) were considered to be differentially expressed.

### 2.8 Enrichment analysis

For differential expressed mRNAs, the online website, namely the database for annotation, visualization and integrated discovery (DAVID)^21^ (Version6.8, https://david‐d.ncifcrf.gov/), was used to perform gene ontology (GO), generate enriched biological pathways (BP),^22^ and Kyoto encyclopedia of genes and genomes (KEGG) pathways.^23^ Terms with *p*‐value <0.5 and enriched counts >2 were considered enriched.

All mRNAs sorted by log (fold change) were loaded into “clusterProfiler” package^24^ for GSEA analysis with MSigDB^25^ (c2.cp.kegg.v7.1.symbols.gmt) dataset as background gene set. “BH” correction was also applied.

Using same MSigDB background gene set, “GSVA”^26^ was performed to calculate enrichment score for each KEGG pathway. Matrix containing KEGG pathway enrichment scores was loaded into “limma” package, and similar analysis as in the case of differential expression of mRNAs/lncRNAs was performed. Pathways with adjusted *p*‐value <0.05 and absolute log (fold change) > 0.263 (1.2 folds change) were considered significant.

### 2.9 Analysis of genes related with tumor immune infiltrating cells

Online database TIMER^27^ (https://cistrome.shinyapps.io/timer) was applied to estimate the abundance of immune cell infiltration in all colon cancer and rectal cancer samples from TCGA and detect six types of immune cells in tumor tissues such as B cells, CD4^+^ T cells, CD8^+^ T cells, neutrophils, macrophages, and dendritic cells.

Spearman correlation between the differential mRNA or differential lncRNA expression and immune infiltration abundance was calculated. Correlation test was also performed, and the differentially expressed mRNAs or lncRNAs that show absolute correlation of more than 0.4 with immune infiltration abundance were defined as immune‐related mRNAs or lncRNAs.

### 2.10 Construction of ceRNA network

For identified CD4^+^ T cell and CD8^+^ T cell related mRNAs and lncRNAs, Pearson correlation score and correlation test were performed between them in matched samples, and “BH” correction was utilized to obtain the adjusted *p*‐value. lncRNA–mRNA relationship pairs with correlation >0.7 (cooperative expression) and adjusted *p*‐value <0.05 were chosen for the subsequent ceRNA network construction.

For each lncRNA in co‐expression lncRNA–mRNA relationship, prediction module from DIANA‐LncBase v.2 database^28^ (http://carolina.imis.athena‐innovation.gr/diana_tools) was used to predict lncRNA–miRNA interaction, and lncRNA–miRNA pairs with relationship score >0.9 were obtained for further analysis.

For each mRNA in lncRNA–mRNA co‐expression relationship, the results from six commonly used databases, namely miRWalk, Targetscan, Microt4, PITA, RNA22, and miRanda, were integrated. In addition, miRWalk2.0^28^ (http://zmf.umm.uni‐heidelberg.de/apps/zmf/mirwalk2/) was then employed for miRNA predictions. In case the miRNA–mRNA relationship pairs appear in the prediction results in more than five databases, the miRNA was involved in the regulation of the corresponding target mRNA, and the miRNA–mRNA pair was selected for further studies.

Finally, using the miRNA–mRNA and lncRNA–miRNA relationship pairs obtained above, the lncRNAs and mRNAs that were predicted to be regulated by the same miRNA and showed positive co‐expression relationship (correlation >0.7) were used to construct ceRNA network. CytoNCA function^29^ from Cytoscape software^30^ (version: 3.4.0, http://chianti.ucsd.edu/cytoscape‐3.4.0/) was used for node connectivity (degree) analysis. The results were interpreted based on the knowledge that the higher the connectivity is, the higher the importance of the node in the network is when the parameter is set to without weight.

### 2.11 Validation of key genes

Two datasets GSE103479 and GSE72970 were used to extract the corresponding expression values of key mRNAs and key immune checkpoints in each sample. Subsequently, Pearson correlation of each key mRNA and key immune checkpoint were calculated within the matched samples.

### 2.12 Statistical analysis

All packages used in this study were based on R (version: 3.5.1) package. Statistical analysis was performed by GraphPad Prism 8.0.2. For measurement data, *t*‐test was selected for two groups comparison, and ANOVA was selected for multiple group comparison. Spearman coefficients were select to perform correlation analysis. *p*‐value or adjusted *p*‐value <0.05 were considered as statistically significant in the whole study.

## 3 RESULTS

### 3.1 Clinical correlation analysis

**FIGURE 1:**
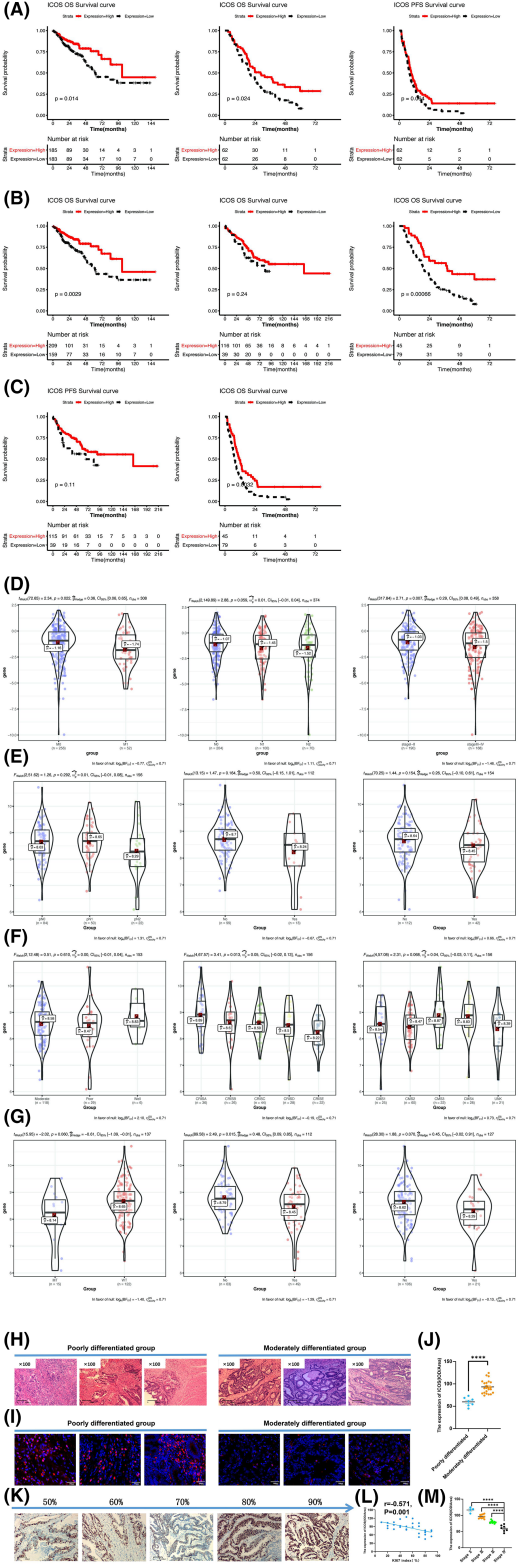
Clinical correlation. (A) K–M plots using groups classified with ICOS expression's median value for OS in TCGA‐COADREAD (top left), OS in GSE72970 (top middle), and PFS in GSE72970 (top right). (B) K–M plots using groups classified with ICOS's optimal threshold value for OS in TCGA‐COADREAD (left), GSE103479 (middle), and GSE72970 (right). (C) K–M plots using groups classified with ICOS's optimal threshold value for PFS in GSE103479 (left) and GSE72970 (right). (D) Expression of ICOS in TCGA‐COADREAD cohort under feature pathologic_M (left), pathologic_N (middle), and tumor_stage (right). (E–G) Expression of ICOS in GSE103479 cohort under feature pathologic_N, Perineural_invasion, recurrence, differentiation, CRIS subtype, CMS subtype, BRAF mutation, LVI, and EVI (order from top left to bottom right). (H) The histological structures of poorly and moderately differentiated CRC patients under 100× microscope. (I, J) The expression of ICOS protein in patients with different degrees of differentiation was detected by IF. (K, L) The expression of Ki67 was detected by IHC, and the correlation between the expression of ICOS and Ki67 was analyzed. (M) The expression of ICOS in different pathological stages was compared.

For each of the 14 immune checkpoints, each of the datasets was classified according to the median expression levels of each gene and the corresponding K–M survival curve was plotted (Figure 1). The results revealed that out of the 14 immune checkpoints, only ICOS showed significant correlation with OS in TCGA‐COADREAD and GSE72970 datasets (*p* = 0.014, *p* = 0.024, Figure 1A). However, the evident but not statistically significant difference of ICOS with PFS was also shown in GSE72970 dataset (*p* = 0.064, Figure 1A). Besides, ICOS, SIRPA, TNFRSF9, VTCN1, and PDL1 also indicated significant correlation with OS or PFS in one or two datasets (Figure S2). Moreover, same datasets were also grouped into high‐ and low‐expression groups according to the optimal expression threshold of each gene. The results revealed that in TCGA‐COADREAD data set, ICOS, BTLA, CTLA4, HAVCR2, LAG3, PDCD1, TIGIT, and TNFRSF9 were all significantly correlated with OS (*p* < 0.05) (Figure S2). In GSE103479 dataset, BTLA, CTLA4, PDL1, LAG3, and SIRPA were significantly associated with the OS and PFS (Figure S2). Although the association of ICOS does not surpass the significance threshold, it still presented a trend that the higher the expression of ICOS is, the better the prognosis of OS and PFS in CRC patients is. In GSE72970 dataset, ICOS, CTLA4, PDCD1, SIRPA, TNFRSF4, TNFRSF9, and VTCN1 were significantly associated with OS, and ICOS, PDL1, HAVCR2, NT5E, TIGIT, TNFRSF4, and TNFRSF9 were significantly related to PFS (Figure S2). Altogether, our analyses revealed that ICOS was the only immune checkpoint that had significant correlation with OS or PFS in almost all the three cohorts (*p* = 0.0029, *p* = 0.24, *p* = 0.00066, *p* = 0.11, *p* = 0.0032, Figure 1B,C).

To detect the correlation between immune checkpoints and clinical features, the distribution for each of the immune checkpoints under different clinical factors in each dataset was determined, and the genes with *p*‐value <0.05 were selected as to be significantly correlated. In the TCGA‐COADREAD dataset, ICOS was found to be significantly correlated with pathologic_M, pathologic_N, and tumor_stage (*p* = 0.022, *p* = 0.059, *p* = 0.007, Figure 1D). Besides, ICOS, SIRPA, and LAG3 also show significant correlation with three or more clinical features (Figure S3). In GSE103479 dataset, ICOS exhibited significant correlation with consensus molecular subtypes (CMS) subtype, CRC intrinsic subtypes (CRIS), and LVI out of all nine clinical features. What's more, ICOS, SIRPA, and TIGIT also show correlation with multiple clinical features (Figure S3). In GSE72970 data set, only CD47 and PDCD1 were found to be significantly related to metastasis (*p* < 0.05). According to the preliminary prognosis results in combination with the analysis mentioned above, it is obvious that ICOS might be a key immune checkpoint molecule that is not only significantly associated with prognosis but also closely correlated with clinical characteristics. By exploring the association between the expression of ICOS and each clinical feature, it was revealed that the expression of ICOS is evidently higher in N0, M0, and stage 1–2 samples in the TCGA cohort (Figure 1D, middle and right). Additionally, the results from GSE103479 dataset illustrated that ICOS has relatively higher expression in samples with no neural invasion, no recurrence, good differentiation, CRISA, CMS3, BRAF wild, or no LVI conditions (*p* = 0.292, *p* = 0.164, *p* = 0.154, *p* = 0.61, *p* = 0.013, *p* = 0.068, *p* = 0.060, *p* = 0.015, *p* = 0.07, Figure 1E–G). These results revealed that the increase in the expression of ICOS might indicate lower degree of malignancy of the tumor, which might be the reason for better prognosis.

To further verify the correlation between ICOS and clinicopathological features, a total of 30 CRC patients were enrolled to validate the association between ICOS and pathological features in CRCs, including differentiation degree, pathological stage, and the expression of Ki67. Pathological sections with different degrees of differentiation were observed under a microscope at 100× magnification (Figure 1H). The expression of ICOS protein in patients with different degrees of differentiation was detected by IF. Based on the results, the expression of ICOS protein in the poorly differentiated group was lower than that in the moderately differentiated group (*p* < 0.001, Figure 1I,J). The expression of Ki67 was detected by IHC, and the correlation between the expression of ICOS and Ki67 was analyzed. The results demonstrated that the expressions of ICOS were negatively correlated with Ki67 (*p* = −0.571, Figure 1K,L). Furthermore, the expression of ICOS in different pathological stages was compared. It was found that the expression of ICOS was the highest in stage IV and the lowest in stage I, and there were significant differences in different pathological stages (*p* < 0.001, Figure 1M).

### 3.2 Differential enrichment of mRNAs and lncRNAs in immune‐related pathways

**FIGURE 2:**
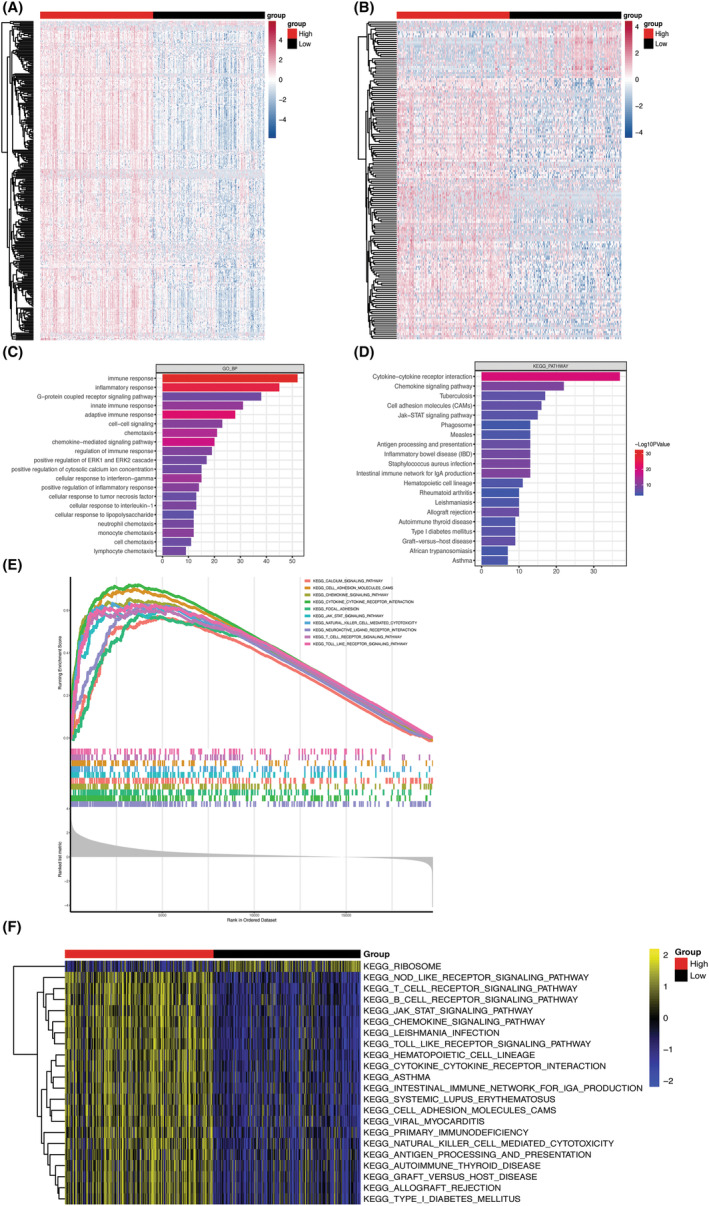
(A, B) Heat map of differentially expressed mRNAs (left) and lncRNAs (right). (C, D) Top 20 enriched GO BP and KEGG pathways. (E) Top 10 GSEA enriched KEGG pathways. (F) Heat map of up‐ and down‐regulated KEGG pathways.

To understand the role of ICOS in CRC, 289 up‐regulated and 4 down‐regulated mRNAs were identified, and 128 up‐regulated and 28 down‐regulated lncRNAs in the ICOS high‐ and low‐expression groups were also identified. The heat map of differentially expressed mRNAs and lncRNAs (Figure 2A,B) also revealed that most of the mRNAs and lncRNAs were significantly up‐regulated in ICOS high‐expression group as compared to the low‐expression group. Functional annotation of these differentially expressed genes showed that out of all the enriched 121 GO BP terms and 31 KEGG pathways (Supplementary file 1), the top 20 terms (Figure 2C,D) have close association with immunity, such as immune response, inflammatory response, chemokine signaling pathway, JAK–STAT signaling pathway, and so on.

In addition to GO analysis, GSEA pathway enrichment analysis which is based on the entire mRNA expression matrix also detected 30 up‐regulated KEGG pathways (Supplementary file 2), of which many terms (Figure 2E) reflect immune‐related signature such as chemokine signaling pathways, JAK–STAT pathway, and so on.

Besides regular pathway analysis, GSVA (Gene Set Variation Analysis) algorithm was also employed to calculate the enrichment score of each KEGG pathway in each sample, and “limma” was used to analyze differential pathways. The results revealed a total of 21 significantly up‐regulated and 1 significantly down‐regulated KEGG pathways (Figure 2F).

Some interesting immune‐related pathways, including chemokine,^31^ JAK–STAT,^32^ calcium,^33^ and other important genes, appeared more frequently in the above results, which indicates that ICOS may play more important roles by interacting with many immune‐related genes.

### 3.3 Abundance of tumor immune infiltrating cells

**FIGURE 3:**
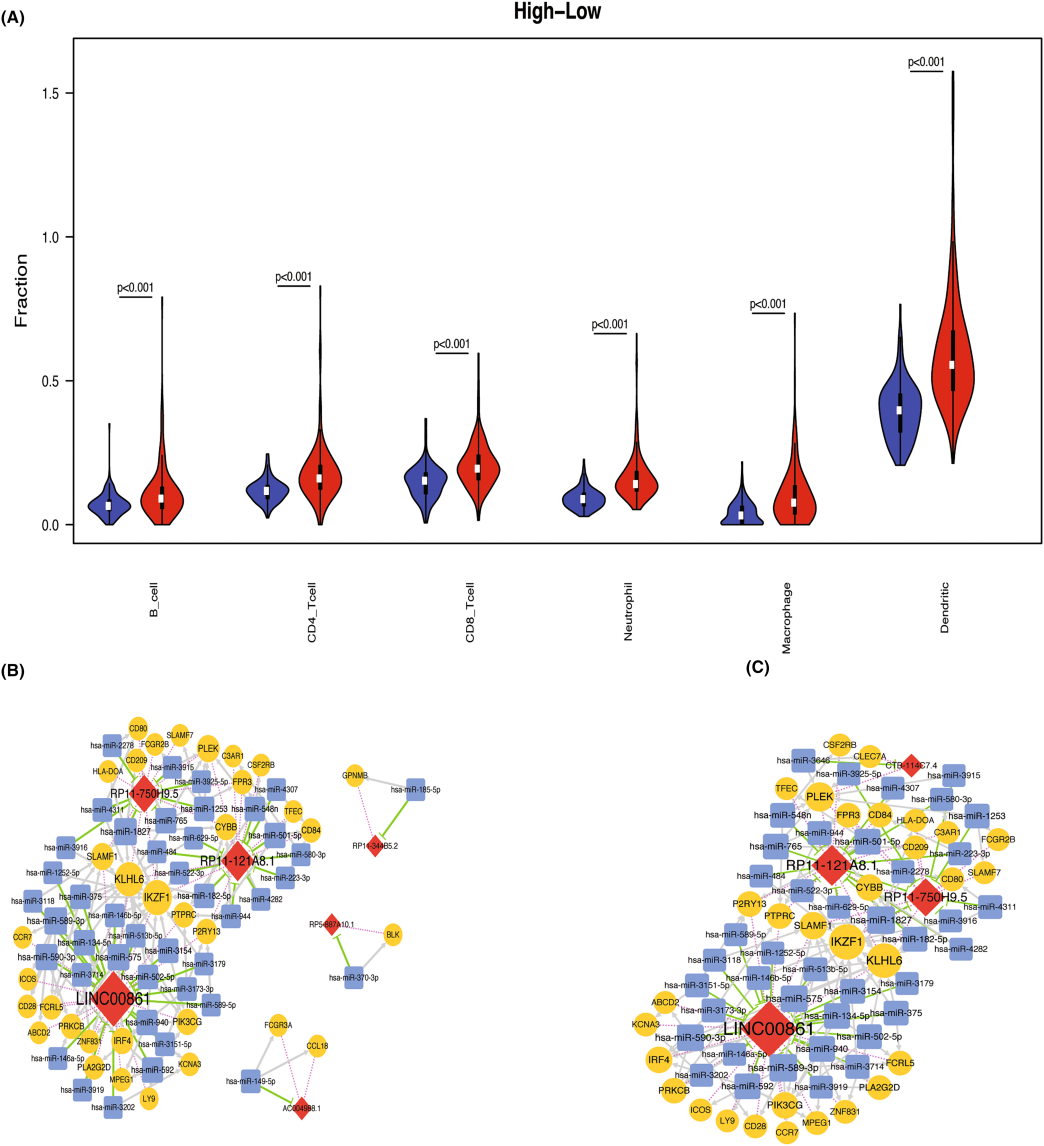
Tumor immune infiltrating cells and ceRNA network. (A) Abundance of six immune cell types in ICOS high‐ and low‐expression CRC patient groups. (B, C) ceRNA network for CD4^+^ T cells (B) and CD8^+^ T cells (C). Key‐Red diamond: up‐regulated lncRNA; yellow circle: up‐regulated mRNA; blue square: predicted miRNA; gray arrow: miRNA‐mRNA; green T‐shaped line: lncRNA–miRNA; red dotted line: lncRNA–mRNA; and size of node: degree of connection.

To obtain a more comprehensive result, online database TIMER was used to analyze the infiltration abundance of six types of immune cells (B cell, CD4^+^ T cell, CD8^+^ T cell, neutrophil, macrophage, and dendritic cells) in TCGA cohort, and then, their abundance was plotted in ICOS high‐ and low‐expression groups. The results clearly revealed that the infiltration abundance of the six immune cells types in ICOS high‐expression group was significantly higher than that in low‐expression group (all *p* < 0.001, Figure 3A).

Furthermore, Spearman correlation was calculated between the differential mRNAs and differential lncRNAs, and CD4^+^ and CD8^+^ cells, respectively. Altogether, the results revealed that 198 mRNAs and 57 lncRNAs were significantly correlated with CD4^+^ cells, and 134 mRNAs and 16 lncRNAs were significantly related to CD8^+^ cells. These findings were further utilized for the construction of the ceRNA network.

### 3.4 Construction of the ceRNA network

To design the ceRNA network, Pearson correlation was calculated based on 198 mRNAs and 57 lncRNAs that were significantly related to CD4^+^ cells obtained in the previous step. The analyses of the mRNAs and lncRNAs proved that 12 lncRNAs and 93 mRNAs developed 215 lncRNA–mRNA relationship pairs (Supplementary file 3). Further prediction of miRNA–lncRNA interactions generated 169 lncRNA–miRNA relationship pairs, including 8 lncRNAs and 160 miRNAs (Supplementary file 4). For prediction of miRNAs involved in the regulation of mRNAs, as described in the method section, a total of 3297 miRNA–mRNA relationship pairs, including 76 mRNAs and 913 miRNAs, were obtained (Supplementary file 5). Furthermore, for lncRNA–miRNA–mRNA pairs with common miRNA‐associated regulation, lncRNA–mRNA positive co‐expression relationship thresholding (*r* > 0.7) was performed. Finally, the results revealed 96 lncRNA–miRNA–mRNA relationship pairs, including 6 lncRNAs, 43 miRNAs, and 34 mRNAs in the final ceRNA network (Figure 3B; Supplementary file 6).

The same method of analysis was employed on 134 mRNAs and 16 lncRNAs that were significantly related to CD8^+^ cells. The results indicated that the existence of 162 lncRNA–mRNA relationship pairs, including 7 lncRNAs and 80 mRNAs (Supplementary file 7), 164 lncRNA–miRNA relationship pairs, including 7 lncRNAs and 154 miRNAs (Supplementary file 8), 3121 miRNA–mRNA relationship pairs, including 68 mRNAs and 897 miRNAs (Supplementary file 9), and finally 94 lncRNA–miRNA–mRNA relationship pairs, including 4 lncRNAs, 41 miRNAs, and 31 mRNAs for CD8^+^ ceRNA network (Figure 3C; Supplementary file 10).

**FIGURE 4:**
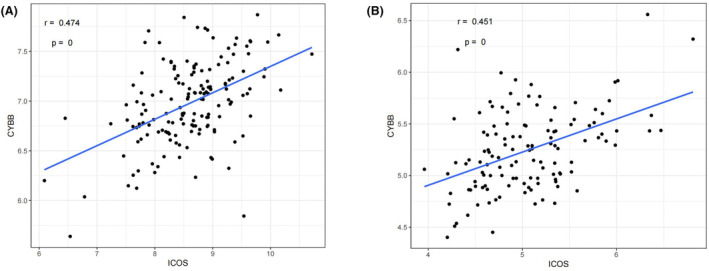
Scatter plot representing correlation between CYBB and ICOS in GSE103479 (left) and GSE72970 (right).

To determine the specific genes that might be involved in the role of ICOS in CRC, 10 mRNAs (IKZF1, KLHL6, PLEK, SLAMF1, CYBB, IRF4, PIK3CG, P2RY13, PRKCB, and PTPRC) were considered as key genes as they ranked as top 10 in both the networks (Supplementary files 6 and 10). The correlation between these 10 genes and ICOS was validated in the two independent cohorts, and it was revealed that while CYBB, IRF4, PIK3CG, PRKCB, and PTPRC were significantly positively correlated with ICOS in GSE103479 dataset, all the 10 genes were significantly positively correlated with ICOS in GSE72970 data set. Here, we only displayed the CYBB correlation result for representation purpose (*r* = 0.474, *r* = 0.451, Figure 4).

## 4 DISCUSSION

Tumor cells escape immune cell attack by inducing abnormal expression of immune checkpoints. As immune system regulators, immune checkpoints are critical in maintaining autoimmune tolerance and regulating the duration and scope of immune responses in peripheral tissues. In this study, the prognostic and clinical significance of 14 well‐studied immune checkpoint molecules was explored. The most robust targets for detailed analysis were further investigated using computational biology methods, and ICOS was identified as a potential target. In addition, multiple differentially expressed mRNAs and long non‐coding RNAs (lncRNAs) showing immune‐related features were associated with CD4^+^ and CD8^+^ cell components. Subsequently, the lncRNA–miRNA–mRNA network was established, and the top 10 associated genes in both networks were identified. This study may contribute to the diagnosis and prognosis assessment of CRC patients.

The level of target protein expression in the tumor microenvironment, infiltrated T cells, and other types of immune cells are strongly correlated with the immune checkpoint inhibitor response. Tumor mutational burden (TMB) is the variable response of CRC to immune checkpoint blockade. Bortolomeazzi et al. found the limitations of TMB as a predictor of anti‐PD1 immunotherapy response in CRC, and anti‐PD1 drugs release PD1‐PDL1 interactions between CD8^+^ T cells and macrophages, thereby promoting cytotoxic anti‐tumor activity.^34^ The consistent molecular subtypes (CMS) of CRC have different immunological, interstitial, and clinicopathological features. Distinct CAFs and C1Q^+^ TAMs can be used to explain the significant heterogeneity of CMS within the tumor, and targeted therapy of specific CAF subtypes and C1Q^+^ TAMs may promote immunotherapy response in CRC patients.^35^ In this study, it was found that ICOS can be used as a promising prognostic target, and immune‐related pathways, such as chemokine signaling pathway and JAK–STAT signaling pathway, may be responsible for the differences in prognostic outcomes. Although immune checkpoint shows great promise in treating cancers with a high mutation burden, there are still many patients who do not respond to immune checkpoint therapy. In response, combination strategies have been developed to induce ICD to increase tumor auxiliary and sensitivity to ICI therapy.^36^ For example, El‐Sayes et al. demonstrated that chemotherapy combined with oncolytic virus therapy makes CRC sensitive to immune checkpoint inhibitors in a CDC1‐dependent manner.^37^

In the past few decades, extensive research has been accomplished in exploring immune surveillance, especially PD‐1/CD274 signaling that is involved in the development and progression of cancer. The fact that the extracellular domain PD‐1 shares 21%–33% sequence identity with ICOS, which indicates that ICOS might play an important role in regulating immune microenvironment of cancer.^38^ ICOS (CD278, AILIM, H4), a member of the co‐stimulating B7‐1/B7‐2‐CD28/CTLA‐4 family, was first found to enhance T‐cell response.^39^ After TCR participation, ICOS are rapidly induced to transmit positive co‐stimulatory signals, and ICOS expression is low in naive T cells.^40^ ICOS ligands can be expressed on specialized antigen‐presenting cells (APCs), such as macrophages, dendritic cells, and endothelial cells.^41^ The most prominent biological effects of ICOS co‐stimulation are the production and maintenance of lymphoid germinal centers (GCs), the help of T cell‐dependent B cells, and antibody class switching.^42^, ^43^ In addition, the ICOS/ICOSL pathway is also important for other CD4^+^ and CD8^+^ T‐cell subsets in promoting cell proliferation, survival, and differentiation.^44^ Surprisingly, ICOS co‐stimulation regulates tumor immunity at two different levels.^8^ ICOS not only promotes the activation of anti‐tumor cytotoxic T cells, but also promote the immunosuppressive activity of Treg to play a pro‐tumor character. It is this dual role that further increases the difficulty of ICOS in tumor diagnosis and treatment. Therefore, more studies are needed to further understand the biological characteristics of ICOS in tumors.

With the deepening of research, the potential of ICOS in disease progression and prognosis is gradually emerging. For example, Strauss et al. found that ICOS expression on Tregs of tumor‐infiltrating lymphocytes in the microenvironment of metastatic melanoma is associated with poor prognosis.^45^ Additionally, the amplification of ICOS^+^ Tregs in peripheral blood after IL‐2 treatment also predicted adverse clinical outcomes.^46^ In the immune microenvironment of CRC, unlike in melanoma, ICOS expression is associated with high expression of CTLA‐4 and PD‐1 on lymphocytes.^47^ In advanced gastric cancer, the proportion of ICOS in Tregs is highly expressed.^48^ In breast cancer, ICOS have also been described as being associated with poor prognosis.^49^ Different from this result, Lee et al. found that low expression of ICOS gene in peripheral blood is related to tumor progression.^50^ Increased proportions of ICOS^+^ T cells in peripheral blood and tumor microenvironment are associated with improved prognosis.^47^ This is consistent with our findings that the increased level of ICOS expression predicts a lower malignancy of the tumor, which may be the reason for the good prognosis.

As a biomarker for emerging immunotherapies, ICOS regulates the immune response through unclear mechanisms. It has been reported that type I IFN is involved in the expression of ICOS‐L on pDCs in psoriatic lesions and melanoma, which suggested that pDCs may be involved in Treg activation through ICOS/ICOS‐L interaction.^51^, ^52^ In liver cancer, the ICOS/ICOS‐L pathway is involved in the promotion of immune tolerance by producing T‐regulatory type 1 cells (Tr1). Pedroza‐Gonzalez et al. confirmed that tumor‐infiltrated Tr1 cells provide anti‐tumor immunity through ICOS/ ICOS‐L signaling and IL‐10 production through ICOS‐L^+^ pDCs.^53^ In addition, studies have illustrated that CD4^+^ T‐cell ICOS expression increases in peripheral blood and tumor tissues of patients treated with anti‐CTLA4 for bladder cancer,^54^ breast cancer,^55^ and non‐small cell lung cancer.^56^ To clarify the biological function of ICOS in CRC, functional annotation and enrichment pathway analysis of differential genes were carried out in this study. The results proved that differential genes were mainly enriched in immune‐related pathways, including chemokine pathway and JAK–STAT pathway. It also suggested that ICOS may work by interacting with many immune‐related genes.

In this study, ICOS was determined as the most robust immune checkpoint molecule from the 14 candidates selected for survival analysis and clinical feature characterization. The analysis of differentially expressed genes combined with immune infiltration data revealed hundreds of mRNA and lncRNA in ceRNA networks as potential regulatory genes of ICOS. Finally, 10 genes with differential expression and strongly correlated with CD4^+^ and CD8^+^ T cells that might play important roles in ceRNA network were identified. Many of these 10 genes function as well‐known tumor activator or suppressor genes in various kinds of cancer. For example, KLHL6 that involved in B‐lymphocyte antigen receptor signaling and germinal‐center B‐cell maturation shows connection with all the three lncRNA and many predicted miRNAs in both the ceRNA networks and is reported to be a tumor suppressor in diffuse large B‐cell lymphoma.^57^ IKZF1 is known to be an important factor in acute lymphoblastic leukemia,^58^ IRF4 drives tumor growth in several lymphoid malignancies,^59^ and PIK3CG is closely associated with claudin‐low breast cancer migration.^60^ Considering that at least 4 of selected 10 genes were discovered to be firmly correlated with cancer progression, we can assuredly hypothesize that these 10 genes might play important roles in mediating the immune‐related functions of ICOS in CRC.

Although our study has shown encouraging results, there are still some limitations. As our study was primarily based on bioinformatics methods, additional and extensive verification is required before considering the clinical application of ICOS. Most importantly, how ICOS regulates the immune system of CRC requires further experimental exploration and validation.

## 5 CONCLUSION

In summary, 14 immune checkpoint genes were screened, and it was found that ICOS was highly correlated with the survival rate and clinical characteristics of CRC patients. The differentially expressed genes in ICOS high‐expression group were enriched in a variety of immune‐related pathways. Additionally, a ceRNA network in which the expression of top 10 genes is highly correlated with ICOS was also constructed. Our study suggested the possible mechanism of the immune checkpoint molecule ICOS and associated genes underlying the occurrence and development of CRC. The implication that ICOS might be used for prognosis and detection of CRC lays a solid foundation for further research to explore the mechanism of immune checkpoints in CRC.

## AUTHOR CONTRIBUTIONS

**Jian Chu:** Formal analysis (equal); writing – review and editing (equal). **Yinghang Wu:** Writing – original draft (equal). **Zhanbo Qu:** Methodology (equal). **Jing Zhuang:** Validation (equal). **Jiang Liu:** Investigation (equal). **Shugao Han:** Visualization (equal). **Wei Wu:** Conceptualization (equal). **Shuwen Han:** Conceptualization (equal).

## FUNDING INFORMATION

This work was supported by Key research and development project of Science and Technology Department of Zhejiang Province (No. 2022C03026), Public Welfare Technology Application Program of Huzhou (No. 2021GY05), and Zhejiang Medical and Health Technology Project (No. 2022KY1178).

## CONFLICT OF INTEREST STATEMENT

The authors declare that no potential conflicts of interest exist.

## ETHICS STATEMENT AND CONSENT TO PARTICIPATE

The clinical protocols involving the patients and the informed consent form were approved by the Chinese Clinical Trial Registry (http://www.chictr.org.cn, No. ChiCTR2200061039) and Ethics Committee of Huzhou Central Hospital (No. 202202005‐02). The datasets generated during the current study were collected from public data repositories.

## Supporting information


Supplementary file 1.

Supplementary file 2.

Supplementary file 3.

Supplementary file 4.

Supplementary file 5.

Supplementary file 6.

Supplementary file 7.

Supplementary file 8.

Supplementary file 9.

Supplementary file 10.

Figure S1.

Figure S2.

Figure S3.

## Data Availability

The datasets generated during the current study are not publicly available but obtained from corresponding authors on reasonable request.
