# Supplementary material for: Transcriptional profile and immune infiltration in colorectal cancer reveal the significance of inducible T‐cell costimulator as a crucial immune checkpoint molecule
Source: Cancer Med. 2024 Mar 20;13(6):e7097. doi: 10.1002/cam4.7097 (PMC10952025; doi:10.1002/cam4.7097)
Supplement: Supplementary file 1 [file CAM4-13-e7097-s002.pdf]

Supplementary file 1. Functional annotation and enriched pathways of DEGs.

Category	Term	Count	%	PValue	Genes	List Total	Pop Hits	Pop Total
GOTERM_BP_	GO:0006955	52	18.909091	1.81E-32	IL21, CXCL	245	421	16792
GOTERM_BP_	GO:0006954	45	16.363636	3.26E-27	CCL13, CXC	245	379	16792
GOTERM_BP_	GO:0002250	28	10.181818	3.29E-22	LILRA6, CD	245	148	16792
GOTERM_BP_	GO:0070098	20	7.2727273	2.56E-19	CCL13, CCL	245	71	16792
GOTERM_BP_	GO:0006935	21	7.6363636	9.64E-16	CCL13, CCL	245	122	16792
GOTERM_BP_	GO:0071346	15	5.4545455	5.68E-14	GBP6, GBP5	245	57	16792
GOTERM_BP_	GO:0045087	31	11.272727	1.06E-12	BLK, CD84,	245	430	16792
GOTERM_BP_	GO:0002548	12	4.3636364	1.40E-11	CCL13, CCL	245	42	16792
GOTERM_BP_	GO:0007267	23	8.3636364	2.05E-11	IL10, FCRL	245	254	16792
GOTERM_BP_	GO:0050729	14	5.0909091	3.73E-11	IL21, CCL1	245	73	16792
GOTERM_BP_	GO:0050776	19	6.9090909	1.17E-10	SH2D1A, CR	245	178	16792
GOTERM_BP_	GO:0071347	13	4.7272727	4.06E-10	CCL13, CCL	245	71	16792
GOTERM_BP_	GO:0030593	12	4.3636364	2.59E-09	CCL13, CCL	245	66	16792
GOTERM_BP_	GO:0048247	9	3.2727273	4.29E-09	CCL13, CCL	245	28	16792
GOTERM_BP_	GO:0070374	17	6.1818182	5.67E-09	CCL13, CCL	245	175	16792
GOTERM_BP_	GO:0007186	38	13.818182	9.32E-09	FCN1, CCL1	245	899	16792
GOTERM_BP_	GO:0007204	15	5.4545455	9.38E-09	XCR1, FPR1	245	134	16792
GOTERM_BP_	GO:0060326	11	4	3.10E-08	CCL13, CXC	245	65	16792
GOTERM_BP_	GO:0071356	13	4.7272727	6.76E-08	CCL13, CCL	245	110	16792
GOTERM_BP_	GO:0071222	12	4.3636364	7.68E-07	IL10, CXCL	245	113	16792
GOTERM_BP_	GO:0007165	39	14.181818	2.01E-06	DTHD1, IL2	245	1161	16792
GOTERM_BP_	GO:0042102	9	3.2727273	2.36E-06	IL21, IL6,	245	60	16792
GOTERM_BP_	GO:0006968	9	3.2727273	3.05E-06	NCR1, CXCL	245	62	16792
GOTERM_BP_	GO:0002407	6	2.1818182	3.34E-06	CXCR1, CCL	245	17	16792
GOTERM_BP_	GO:0032496	13	4.7272727	5.05E-06	CXCL9, TNF	245	164	16792
GOTERM_BP_	GO:0042832	6	2.1818182	6.12E-06	IL10, IL6,	245	19	16792
GOTERM_BP_	GO:0002430	5	1.8181818	8.53E-06	CR1, FPR1,	245	10	16792
GOTERM_BP_	GO:0031295	9	3.2727273	1.73E-05	CCL21, CD8	245	78	16792
GOTERM_BP_	GO:0007166	15	5.4545455	5.00E-05	TNFRSF13B,	245	274	16792
GOTERM_BP_	GO:0044130	5	1.8181818	6.90E-05	IL10, IFNG	245	16	16792
GOTERM_BP_	GO:0045765	6	2.1818182	7.76E-05	IL6, SFRP1	245	31	16792
GOTERM_BP_	GO:0050853	7	2.5454545	1.28E-04	BLK, IGLL5	245	54	16792
GOTERM_BP_	GO:0048304	4	1.4545455	1.61E-04	IFNG, TBX2	245	8	16792
GOTERM_BP_	GO:0030101	5	1.8181818	1.75E-04	NCR1, IL21	245	20	16792
GOTERM_BP_	GO:0042130	6	2.1818182	1.85E-04	IL10, PLA2	245	37	16792
GOTERM_BP_	GO:0008037	5	1.8181818	2.59E-04	CADM3, CLE	245	22	16792
GOTERM_BP_	GO:0042742	10	3.6363636	2.72E-04	IL10, GBP6	245	145	16792
GOTERM_BP_	GO:0045766	9	3.2727273	2.75E-04	GREM1, CYS	245	115	16792
GOTERM_BP_	GO:0007155	18	6.5454545	4.08E-04	CSF3R, SIG	245	459	16792
GOTERM_BP_	GO:0006874	8	2.9090909	4.11E-04	CCL13, CCL	245	93	16792
GOTERM_BP_	GO:0050715	5	1.8181818	4.33E-04	IL10, CLEC	245	25	16792
GOTERM_BP_	GO:0032735	5	1.8181818	4.33E-04	IFNG, IL12	245	25	16792
GOTERM_BP_	GO:0045078	4	1.4545455	6.05E-04	IL21, IL12	245	12	16792
GOTERM_BP_	GO:0032695	4	1.4545455	6.05E-04	IL10, MEFV	245	12	16792
GOTERM_BP_	GO:0001781	3	1.0909091	6.25E-04	HCAR2, IL6	245	3	16792
GOTERM_BP_	GO:0051607	10	3.6363636	7.01E-04	CXCL10, IL	245	165	16792

GOTERM_BP_GO:0007157	6	2.1818182	7.73E-04	CADM3, CD2	245	50	16792
GOTERM_BP_GO:0032740	4	1.4545455	7.78E-04	IL21, IL12	245	13	16792
GOTERM_BP_GO:0035589	4	1.4545455	9.80E-04	P2RY12, P2	245	14	16792
GOTERM_BP_GO:0019882	6	2.1818182	0.0011967	IFNG, CD20	245	55	16792
GOTERM_BP_GO:0050852	9	3.2727273	0.0014571	PTPRC, IFN	245	148	16792
GOTERM_BP_GO:0002504	4	1.4545455	0.0017724	HLA-DOA, H	245	17	16792
GOTERM_BP_GO:0072540	3	1.0909091	0.0020429	IL6, IRF4,	245	5	16792
GOTERM_BP_GO:0002690	4	1.4545455	0.0021043	CXCL10, II	245	18	16792
GOTERM_BP_GO:0045954	4	1.4545455	0.0021043	IL21, SH2I	245	18	16792
GOTERM_BP_GO:0030890	5	1.8181818	0.0024	IL21, PTPR	245	39	16792
GOTERM_BP_GO:0034105	3	1.0909091	0.003035	IL21, IL12	245	6	16792
GOTERM_BP_GO:2000107	3	1.0909091	0.003035	CCL21, CCR	245	6	16792
GOTERM_BP_GO:0001768	3	1.0909091	0.003035	CCL21, CCR	245	6	16792
GOTERM_BP_GO:0042346	4	1.4545455	0.0033213	GREM1, IL1	245	21	16792
GOTERM_BP_GO:0090023	4	1.4545455	0.0038049	CCL21, C3A	245	22	16792
GOTERM_BP_GO:0002860	3	1.0909091	0.0042084	CRTAM, IL1	245	7	16792
GOTERM_BP_GO:0002606	3	1.0909091	0.0042084	CCL21, CCR	245	7	16792
GOTERM_BP_GO:0032729	5	1.8181818	0.0043994	IL12B, CD2	245	46	16792
GOTERM_BP_GO:0007187	5	1.8181818	0.0043994	XCR1, CNR2	245	46	16792
GOTERM_BP_GO:0042110	5	1.8181818	0.0047547	CLEC7A, CI	245	47	16792
GOTERM_BP_GO:0045860	5	1.8181818	0.0047547	RELN, PTPR	245	47	16792
GOTERM_BP_GO:0034695	3	1.0909091	0.0055576	CCL21, CCR	245	8	16792
GOTERM_BP_GO:0050728	6	2.1818182	0.0058553	CNR2, IL2R	245	79	16792
GOTERM_BP_GO:0046718	6	2.1818182	0.0061734	CR1, CD209	245	80	16792
GOTERM_BP_GO:0042104	4	1.4545455	0.0068515	IL2RA, IL1	245	27	16792
GOTERM_BP_GO:0046641	3	1.0909091	0.0070773	CD80, CD28	245	9	16792
GOTERM_BP_GO:0051897	6	2.1818182	0.0075673	IL6, CCL21	245	84	16792
GOTERM_BP_GO:0002376	4	1.4545455	0.008379	VSTM1, IDO	245	29	16792
GOTERM_BP_GO:0008284	15	5.4545455	0.0084383	IL21, IL24	245	466	16792
GOTERM_BP_GO:0050900	7	2.5454545	0.0088317	CD84, SELI	245	122	16792
GOTERM_BP_GO:0006959	5	1.8181818	0.0094096	IL6, IFNG,	245	57	16792
GOTERM_BP_GO:0071392	4	1.4545455	0.0100908	IL10, IL6,	245	31	16792
GOTERM_BP_GO:0002003	3	1.0909091	0.0106076	CPA3, CMA1	245	11	16792
GOTERM_BP_GO:0042100	4	1.4545455	0.0110172	IL10, CD79	245	32	16792
GOTERM_BP_GO:0001774	3	1.0909091	0.0126081	IL13, TLR8	245	12	16792
GOTERM_BP_GO:0045086	3	1.0909091	0.0126081	IRF4, CD80	245	12	16792
GOTERM_BP_GO:0032693	3	1.0909091	0.0126081	IL12B, PDC	245	12	16792
GOTERM_BP_GO:0030574	5	1.8181818	0.0139942	MMP12, MMF	245	64	16792
GOTERM_BP_GO:0048469	4	1.4545455	0.0152016	IL21, BFSF	245	36	16792
GOTERM_BP_GO:0007200	5	1.8181818	0.0155232	CYSLTR2, F	245	66	16792
GOTERM_BP_GO:0006952	5	1.8181818	0.0163255	CD84, CXCI	245	67	16792
GOTERM_BP_GO:0031529	3	1.0909091	0.0170556	CCL21, PLE	245	14	16792
GOTERM_BP_GO:2000147	3	1.0909091	0.0170556	CCL21, CCR	245	14	16792
GOTERM_BP_GO:0032461	3	1.0909091	0.0170556	AIM2, MMP1	245	14	16792
GOTERM_BP_GO:0042517	4	1.4545455	0.0175853	IL21, IL6,	245	38	16792
GOTERM_BP_GO:0043547	16	5.8181818	0.0184585	P2RY12, CC	245	565	16792
GOTERM_BP_GO:0002223	6	2.1818182	0.0185605	CLEC4C, CI	245	105	16792
GOTERM_BP_GO:0060333	5	1.8181818	0.0197927	IFNG, IRF4	245	71	16792

GOTERM_BP_	GO:0051209	4	1.4545455	0.0215294	PTPRC, CCI	245	41	16792
GOTERM_BP_	GO:0070527	4	1.4545455	0.0215294	P2RY12, PI	245	41	16792
GOTERM_BP_	GO:0022617	5	1.8181818	0.0247213	MMP12, MMI	245	76	16792
GOTERM_BP_	GO:0046851	2	0.7272727	0.0288512	GREM1, SFI	245	2	16792
GOTERM_BP_	GO:0072610	2	0.7272727	0.0288512	CCR7, CCL1	245	2	16792
GOTERM_BP_	GO:2000473	2	0.7272727	0.0288512	PTPRC, CCI	245	2	16792
GOTERM_BP_	GO:2000041	2	0.7272727	0.0288512	SFRP1, SFI	245	2	16792
GOTERM_BP_	GO:0002302	2	0.7272727	0.0288512	EOMES, IFN	245	2	16792
GOTERM_BP_	GO:0032701	2	0.7272727	0.0288512	IL10, CD84	245	2	16792
GOTERM_BP_	GO:0038043	2	0.7272727	0.0288512	IL5RA, CSI	245	2	16792
GOTERM_BP_	GO:1904956	2	0.7272727	0.0288512	SFRP1, SFI	245	2	16792
GOTERM_BP_	GO:0050731	5	1.8181818	0.0315332	IL6, RELN,	245	82	16792
GOTERM_BP_	GO:0006928	5	1.8181818	0.0366287	IFNG, VNN2	245	86	16792
GOTERM_BP_	GO:0019233	4	1.4545455	0.0397707	CNR2, KCNI	245	52	16792
GOTERM_BP_	GO:0050718	3	1.0909091	0.0401339	AIM2, IFNG	245	22	16792
GOTERM_BP_	GO:0001954	3	1.0909091	0.0401339	SFRP1, CCI	245	22	16792
GOTERM_BP_	GO:0046427	3	1.0909091	0.0401339	IL10, IL6,	245	22	16792
GOTERM_BP_	GO:0046013	2	0.7272727	0.0429644	IL2RA, IL2	245	3	16792
GOTERM_BP_	GO:0006082	2	0.7272727	0.0429644	FMO1, FMO2	245	3	16792
GOTERM_BP_	GO:0051051	2	0.7272727	0.0429644	RSC1A1, TI	245	3	16792
GOTERM_BP_	GO:0051712	2	0.7272727	0.0429644	FCER2, IFN	245	3	16792
GOTERM_BP_	GO:0071731	2	0.7272727	0.0429644	CCR7, CCL1	245	3	16792
GOTERM_BP_	GO:0010560	2	0.7272727	0.0429644	CCL21, CCI	245	3	16792
GOTERM_BP_	GO:0002292	2	0.7272727	0.0429644	CLEC4D, CI	245	3	16792
GOTERM_BP_	GO:0002408	2	0.7272727	0.0429644	CCR7, CCL1	245	3	16792
GOTERM_BP_	GO:0032733	3	1.0909091	0.0435439	CD28, IL12	245	23	16792
GOTERM_BP_	GO:0046854	5	1.8181818	0.0481622	TRAT1, CD8	245	94	16792
KEGG_PATHW	hsa04060:C	37	13.454545	5.34E-21	IL21, CCL1	150	243	6879
KEGG_PATHW	hsa04672:I	13	4.7272727	1.86E-10	IL10, TNFI	150	47	6879
KEGG_PATHW	hsa04062:C	22	8	3.66E-10	CCL13, CCI	150	186	6879
KEGG_PATHW	hsa05150:S	13	4.7272727	1.07E-09	IL10, FPR1	150	54	6879
KEGG_PATHW	hsa05321:I	13	4.7272727	8.53E-09	IL10, IL21	150	64	6879
KEGG_PATHW	hsa05330:A	10	3.6363636	6.11E-08	IL10, IFNG	150	37	6879
KEGG_PATHW	hsa04612:A	13	4.7272727	6.45E-08	KLRC4, KII	150	76	6879
KEGG_PATHW	hsa04514:C	16	5.8181818	3.45E-07	CADM3, NRX	150	142	6879
KEGG_PATHW	hsa05332:C	9	3.2727273	3.52E-07	IL6, IFNG,	150	33	6879
KEGG_PATHW	hsa05152:T	17	6.1818182	1.16E-06	IL10, CR1,	150	177	6879
KEGG_PATHW	hsa04940:T	9	3.2727273	2.54E-06	IFNG, CD80	150	42	6879
KEGG_PATHW	hsa04630:J	15	5.4545455	2.58E-06	IL10, IL21	150	145	6879
KEGG_PATHW	hsa05320:A	9	3.2727273	1.35E-05	IL10, CD80	150	52	6879
KEGG_PATHW	hsa04640:H	11	4	1.61E-05	FCER2, IL6	150	87	6879
KEGG_PATHW	hsa05140:I	10	3.6363636	1.95E-05	IL10, FCGI	150	71	6879
KEGG_PATHW	hsa05162:M	13	4.7272727	2.76E-05	SH2D1A, II	150	133	6879
KEGG_PATHW	hsa05310:A	7	2.5454545	3.61E-05	IL10, IL13	150	30	6879
KEGG_PATHW	hsa05143:A	7	2.5454545	6.38E-05	IL10, IL6,	150	33	6879
KEGG_PATHW	hsa04145:F	13	4.7272727	9.05E-05	MSR1, FCAI	150	150	6879
KEGG_PATHW	hsa05323:F	10	3.6363636	1.10E-04	IL6, IFNG,	150	88	6879
KEGG_PATHW	hsa04650:N	11	4	2.91E-04	NCR1, FCGI	150	122	6879

KEGG_PATHW	hsa04380:(	11	4	5.16E-04	LILRA6, FC	150	131	6879
KEGG_PATHW	hsa05322:S	11	4	6.17E-04	IL10, FCGF	150	134	6879
KEGG_PATHW	hsa05340:F	6	2.1818182	7.47E-04	CD79A, PTF	150	34	6879
KEGG_PATHW	hsa05164:I	11	4	0.0043663	CXCL10, II	150	174	6879
KEGG_PATHW	hsa04660:T	8	2.9090909	0.0057948	IL10, PTPF	150	100	6879
KEGG_PATHW	hsa05416:V	6	2.1818182	0.0075506	CD80, CD28	150	57	6879
KEGG_PATHW	hsa05146:A	8	2.9090909	0.0079257	IL10, IL6,	150	106	6879
KEGG_PATHW	hsa04620:T	8	2.9090909	0.0079257	CXCL10, II	150	106	6879
KEGG_PATHW	hsa04080:N	13	4.7272727	0.0167151	P2RY13, P2	150	277	6879
KEGG_PATHW	hsa05145:T	7	2.5454545	0.0317431	IL10, IFNC	150	110	6879

Fold Enric	Bonferroni	Benjamini	FDR
8.465597	2.51E-29	2.51E-29	2.40E-29
8.1378493	4.52E-24	2.26E-24	2.16E-24
12.966795	4.56E-19	1.52E-19	1.46E-19
19.306697	3.55E-16	8.86E-17	8.48E-17
11.797658	1.38E-12	2.67E-13	2.56E-13
18.03652	7.88E-11	1.31E-11	1.26E-11
4.9411675	1.47E-09	2.09E-10	2.00E-10
19.582507	1.94E-08	2.43E-09	2.32E-09
6.2062671	2.84E-08	3.16E-09	3.02E-09
13.144423	5.16E-08	5.16E-09	4.94E-09
7.3159367	1.62E-07	1.47E-08	1.41E-08
12.549353	5.62E-07	4.68E-08	4.48E-08
12.461596	3.58E-06	2.76E-07	2.64E-07
22.030321	5.94E-06	4.24E-07	4.06E-07
6.6580525	7.86E-06	5.23E-07	5.01E-07
2.8970784	1.29E-05	7.64E-07	7.32E-07
7.672251	1.30E-05	7.64E-07	7.32E-07
11.59887	4.30E-05	2.39E-06	2.28E-06
8.1000371	9.37E-05	4.93E-06	4.72E-06
7.278454	0.0010642	5.32E-05	5.09E-05
2.3023361	0.0027795	1.32E-04	1.27E-04
10.280816	0.0032696	1.49E-04	1.42E-04
9.9491771	0.0042119	1.83E-04	1.76E-04
24.190156	0.0046131	1.93E-04	1.84E-04
5.4329517	0.0069752	2.80E-04	2.68E-04
21.643824	0.0084486	3.26E-04	3.12E-04
34.269388	0.0117504	4.37E-04	4.19E-04
7.9083203	0.0236846	8.55E-04	8.19E-04
3.7521227	0.0669342	0.0023872	0.0022855
21.418367	0.0912054	0.0031855	0.0030498
13.265569	0.1019497	0.003466	0.0033184
8.8846561	0.1620507	0.0055206	0.0052854
34.269388	0.1997991	0.0067489	0.0064614
17.134694	0.2159035	0.0071478	0.0068433
11.114396	0.226609	0.007336	0.0070235
15.576994	0.3015862	0.0099622	0.0095378
4.7268121	0.3141737	0.0100344	0.0096069
5.3639042	0.317256	0.0100344	0.0096069
2.6877951	0.4322909	0.014243	0.0136363
5.8958086	0.4346153	0.014243	0.0136363
13.707755	0.4511349	0.01427	0.0136621
13.707755	0.4511349	0.01427	0.0136621
22.846259	0.5678114	0.0190462	0.0182349
22.846259	0.5678114	0.0190462	0.0182349
68.538776	0.5794751	0.0192301	0.0184109
4.1538652	0.6216816	0.0211082	0.020209

8.2246531	0.657615	0.0224539	0.0214974
21.088854	0.66006	0.0224539	0.0214974
19.582507	0.7430077	0.0276952	0.0265154
7.4769573	0.8097998	0.0331498	0.0317376
4.1678985	0.8674791	0.0395703	0.0378846
16.126771	0.9144612	0.0472083	0.0451973
41.123265	0.9412455	0.0529889	0.0507317
15.230839	0.9460431	0.0529889	0.0507317
15.230839	0.9460431	0.0529889	0.0507317
8.7870225	0.9642203	0.0593567	0.0568282
34.269388	0.9851976	0.0712464	0.0682113
34.269388	0.9851976	0.0712464	0.0682113
34.269388	0.9851976	0.0712464	0.0682113
13.055005	0.9900586	0.0766677	0.0734017
12.461596	0.994926	0.0863895	0.0827094
29.373761	0.9971062	0.0925185	0.0885773
29.373761	0.9971062	0.0925185	0.0885773
7.4498669	0.9977816	0.0937402	0.089747
7.4498669	0.9977816	0.0937402	0.089747
7.2913591	0.9986474	0.0982869	0.0940999
7.2913591	0.9986474	0.0982869	0.0940999
25.702041	0.9995581	0.1131958	0.1083738
5.2054766	0.9997082	0.117531	0.1125243
5.1404082	0.9998127	0.1221448	0.1169415
10.153893	0.9999273	0.1336531	0.1279596
22.846259	0.9999469	0.1361406	0.1303411
4.8956268	0.9999732	0.1435722	0.1374562
9.4536242	0.9999914	0.1558278	0.1491896
2.2061838	0.9999921	0.1558278	0.1491896
3.9325527	0.9999954	0.1609459	0.1540897
6.0121733	0.999998	0.1692504	0.1620404
8.843713	0.9999992	0.1791765	0.1715437
18.692393	0.9999996	0.1859693	0.1780471
8.5673469	0.9999998	0.1907347	0.1826096
17.134694	1	0.2103889	0.2014265
17.134694	1	0.2103889	0.2014265
17.134694	1	0.2103889	0.2014265
5.3545918	1	0.2307384	0.2209091
7.6154195	1	0.247697	0.2371453
5.1923315	1	0.2499958	0.2393462
5.114834	1	0.2598952	0.2488238
14.68688	1	0.2624665	0.2512856
14.68688	1	0.2624665	0.2512856
14.68688	1	0.2624665	0.2512856
7.2146079	1	0.2676451	0.2562437
1.9409211	1	0.2764119	0.2646369
3.9165015	1	0.2764119	0.2646369
4.8266743	1	0.2916259	0.2792029

6.6867098	1	0.3106065	0.2973749
6.6867098	1	0.3106065	0.2973749
4.50913	1	0.3529793	0.3379426
68.538776	1	0.3805607	0.364349
68.538776	1	0.3805607	0.364349
68.538776	1	0.3805607	0.364349
68.538776	1	0.3805607	0.364349
68.538776	1	0.3805607	0.364349
68.538776	1	0.3805607	0.364349
68.538776	1	0.3805607	0.364349
68.538776	1	0.3805607	0.364349
4.1791936	1	0.412014	0.3944625
3.9848125	1	0.4741187	0.4539216
5.2722135	1	0.5000473	0.4787456
9.3461967	1	0.5000473	0.4787456
9.3461967	1	0.5000473	0.4787456
9.3461967	1	0.5000473	0.4787456
45.692517	1	0.5000473	0.4787456
45.692517	1	0.5000473	0.4787456
45.692517	1	0.5000473	0.4787456
45.692517	1	0.5000473	0.4787456
45.692517	1	0.5000473	0.4787456
45.692517	1	0.5000473	0.4787456
45.692517	1	0.5000473	0.4787456
45.692517	1	0.5000473	0.4787456
8.9398403	1	0.5025687	0.4811597
3.6456795	1	0.5512786	0.5277945
6.9827984	9.55E-19	9.60E-19	8.22E-19
12.684681	3.33E-08	1.68E-08	1.43E-08
5.4243011	6.55E-08	2.19E-08	1.88E-08
11.04037	1.92E-07	4.83E-08	4.13E-08
9.3153125	1.53E-06	3.07E-07	2.63E-07
12.394595	1.09E-05	1.66E-06	1.42E-06
7.8444737	1.15E-05	1.66E-06	1.42E-06
5.1673239	6.17E-05	7.05E-06	6.03E-06
12.507273	6.31E-05	7.05E-06	6.03E-06
4.4046328	2.08E-04	2.09E-05	1.79E-05
9.8271429	4.54E-04	3.87E-05	3.31E-05
4.7441379	4.62E-04	3.87E-05	3.31E-05
7.9373077	0.0024098	1.87E-04	1.60E-04
5.7983908	0.0028822	2.07E-04	1.77E-04
6.4591549	0.0034913	2.34E-04	2.01E-04
4.4825564	0.0049305	3.11E-04	2.66E-04
10.700667	0.0064378	3.82E-04	3.27E-04
9.7278788	0.0113535	6.38E-04	5.46E-04
3.9745333	0.0160674	8.57E-04	7.33E-04
5.2113636	0.019432	9.87E-04	8.44E-04
4.134918	0.0507464	0.0024935	0.0021333

3.8508397	0.0881906	0.0042189	0.0036095
3.7646269	0.104568	0.0048275	0.0041302
8.0929412	0.1252217	0.0056034	0.004794
2.8991954	0.5430929	0.0314372	0.0268963
3.6688	0.6466442	0.0401176	0.0343228
4.8273684	0.7424861	0.049194	0.0420882
3.4611321	0.759336	0.049194	0.0420882
3.4611321	0.759336	0.049194	0.0420882
2.1522744	0.9510668	0.1002909	0.0858044
2.9183636	0.9968932	0.1843146	0.1576914
