# Supplementary material for: Transcriptional profile and immune infiltration in colorectal cancer reveal the significance of inducible T‐cell costimulator as a crucial immune checkpoint molecule
Source: Cancer Med. 2024 Mar 20;13(6):e7097. doi: 10.1002/cam4.7097 (PMC10952025; doi:10.1002/cam4.7097)
Supplement: Supplementary file 2 [file CAM4-13-e7097-s003.pdf]

Supplementary file 2. 30 up-regulated KEGG pathways detected by GSEA pathway enrichment ar

	ID	Descriptic	setSize	enrichment	NES	pvalue	p.adjust	qvalues
KEGG_PATHW	KEGG_PATHW	KEGG_PATHW	322	0.5147609	1.246925	0.002997	0.0191594	0.014985
KEGG_NEURC	KEGG_NEURC	KEGG_NEURC	272	0.6272166	1.5137149	0.000999	0.0072987	0.0057085
KEGG_CYTOK	KEGG_CYTOK	KEGG_CYTOK	262	0.720303	1.7372534	0.000999	0.0072987	0.0057085
KEGG_FOCAL	KEGG_FOCAL	KEGG_FOCAL	197	0.5824967	1.3964583	0.000999	0.0072987	0.0057085
KEGG_CHEMC	KEGG_CHEMC	KEGG_CHEMC	185	0.654501	1.5692751	0.000999	0.0072987	0.0057085
KEGG_CALCI	KEGG_CALCI	KEGG_CALCI	177	0.5716953	1.3689455	0.000999	0.0072987	0.0057085
KEGG_JAK_S	KEGG_JAK_S	KEGG_JAK_S	155	0.612726	1.4643425	0.000999	0.0072987	0.0057085
KEGG_NATUF	KEGG_NATUF	KEGG_NATUF	131	0.6334755	1.5088691	0.000999	0.0072987	0.0057085
KEGG_CELL_	KEGG_CELL_	KEGG_CELL_	128	0.7014069	1.6689441	0.000999	0.0072987	0.0057085
KEGG_LEUKC	KEGG_LEUKC	KEGG_LEUKC	115	0.5833726	1.3815171	0.002997	0.0191594	0.014985
KEGG_T_CEI	KEGG_T_CEI	KEGG_T_CEI	107	0.6025178	1.424743	0.000999	0.0072987	0.0057085
KEGG_TOLL_	KEGG_TOLL_	KEGG_TOLL_	101	0.6284898	1.4835434	0.000999	0.0072987	0.0057085
KEGG_HEMAT	KEGG_HEMAT	KEGG_HEMAT	85	0.7559631	1.7749677	0.000999	0.0072987	0.0057085
KEGG_ECM_F	KEGG_ECM_F	KEGG_ECM_F	84	0.6793985	1.5931672	0.000999	0.0072987	0.0057085
KEGG_ANTIC	KEGG_ANTIC	KEGG_ANTIC	80	0.6758125	1.5814638	0.000999	0.0072987	0.0057085
KEGG_FC_EF	KEGG_FC_EF	KEGG_FC_EF	79	0.6299394	1.4725056	0.000999	0.0072987	0.0057085
KEGG_B_CEI	KEGG_B_CEI	KEGG_B_CEI	75	0.5698745	1.3285577	0.006993	0.0417249	0.032634
KEGG_COMPI	KEGG_COMPI	KEGG_COMPI	69	0.6128508	1.4275391	0.002	0.0137692	0.0107692
KEGG_VIRAI	KEGG_VIRAI	KEGG_VIRAI	68	0.6531486	1.5208111	0.001	0.0072987	0.0057085
KEGG_LEISH	KEGG_LEISH	KEGG_LEISH	68	0.7436637	1.7315693	0.001	0.0072987	0.0057085
KEGG_NOD_I	KEGG_NOD_I	KEGG_NOD_I	61	0.6553777	1.5141866	0.001001	0.0072987	0.0057085
KEGG_SYSTE	KEGG_SYSTE	KEGG_SYSTE	54	0.8290567	1.9070651	0.001002	0.0072987	0.0057085
KEGG_AUTOI	KEGG_AUTOI	KEGG_AUTOI	50	0.7852734	1.7991921	0.001002	0.0072987	0.0057085
KEGG_INTES	KEGG_INTES	KEGG_INTES	46	0.8263868	1.8796109	0.001003	0.0072987	0.0057085
KEGG_TYPE_	KEGG_TYPE_	KEGG_TYPE_	41	0.7632483	1.7250276	0.001004	0.0072987	0.0057085
KEGG_TRYPT	KEGG_TRYPT	KEGG_TRYPT	39	0.6394031	1.4427138	0.0050201	0.030986	0.0242349
KEGG_GRAFT	KEGG_GRAFT	KEGG_GRAFT	37	0.8310128	1.8656651	0.0010081	0.0072987	0.0057085
KEGG_PRIMA	KEGG_PRIMA	KEGG_PRIMA	35	0.7394573	1.6509301	0.0010101	0.0072987	0.0057085
KEGG_ALLOC	KEGG_ALLOC	KEGG_ALLOC	35	0.8055966	1.7985944	0.0010101	0.0072987	0.0057085
KEGG_ASTHM	KEGG_ASTHM	KEGG_ASTHM	28	0.8411114	1.8394704	0.0010194	0.0072987	0.0057085

nalysis basing on the entire mRNA expression matrix.

rank	leading_ec	core_enrichment
5766	tags=44%,	MMP1/PIK3CG/FGF10/IL6/PRKCB/CSF3R/FGF7/FLT3/CCNA1/PTGS2/CXCL8/PIK3R5/C
4034	tags=50%,	CTSG/FPR2/CHRNA6/HRH2/P2RY10/TSHR/FPR1/CYSLTR2/P2RY13/SSTR3/FPR3/CNR2/
3700	tags=59%,	CCL13/IFNG/IL13/IL5RA/CXCL13/CCL18/TNFRSF17/CCL7/XCL2/CCL8/CXCL9/CCR4/
3696	tags=41%,	PIK3CG/PRKCB/RELN/PIK3R5/SPP1/IGF1/ITGA4/THBS2/TNC/TNR/PAK3/ITGA11/COL
3207	tags=44%,	CCL13/CXCL13/CCL18/CCL7/XCL2/CCL8/CXCL9/CCR4/CCR8/CCL23/CCL19/CCR3/CCL
4742	tags=46%,	HRH2/CD38/CYSLTR2/PRKCB/HTR7/PTGFR/HTR2A/P2RX2/CYSLTR1/RYR1/P2RX7/PTAF
5285	tags=54%,	IFNG/IL13/IL5RA/IL12B/IL21/IL2RA/IL10/PIK3CG/IL2/IL6/CSF2RB/IL22RA2/IL
3207	tags=37%,	IFNG/NCR1/FCGR3B/KIR3DL2/KIR2DL4/SH2D1A/PIK3CG/FCGR3A/KLRD1/KIR2DL1/KI
3463	tags=57%,	ICOS/PDCD1LG2/PTPRC/HLA-DQA1/CD226/CD80/HLA-DQA2/HLA-DOA/SELL/NRXN1/CD
4205	tags=41%,	PIK3CG/CYBB/PRKCB/ITGAL/ITK/ITGAM/RHOH/PIK3R5/NCF1/ITGB2/ITGA4/NCF2/CL
3633	tags=38%,	IFNG/ICOS/PTPRC/IL10/PIK3CG/IL2/CD28/CD8A/CTLA4/ITK/CD3G/CSF2/RASGRP1/
3845	tags=40%,	TLR8/CXCL9/IL12B/CXCL10/PIK3CG/CD80/IL6/TLR7/CD86/TLR1/CCL4/CXCL8/PIK3
3359	tags=76%,	IL5RA/MS4A1/CR1/CD1B/FCER2/CD38/IL2RA/IL6/CD8A/CSF3R/HLA-DRA/CR2/IL7R/
3635	tags=60%,	RELN/SPP1/SV2B/ITGA4/THBS2/TNC/TNR/ITGA11/COL6A3/COL3A1/COMP/COL1A1/CO
3996	tags=49%,	KLRC4/KIR3DL2/KIR2DL4/HLA-DQA1/HLA-DQA2/HLA-DOA/KLRD1/KIR2DL1/KIR3DL1/
3651	tags=39%,	PLA2G2D/IL13/MS4A2/PIK3CG/PRKCB/CSF2/PIK3R5/BTK/LCP2/PLA2G2A/FCER1G/TN
5339	tags=47%,	PIK3CG/FCGR2B/CD79A/PRKCB/CR2/PIK3R5/BTK/CD22/CD19/LILRB3/CD79B/PIK3CD
4030	tags=52%,	CR1/C3AR1/F13A1/CR2/C1QB/C1QC/C1QA/C1S/C5AR1/CFH/MASP1/C1R/SERPING1/C3
4641	tags=57%,	HLA-DQA1/CD80/HLA-DQA2/HLA-DOA/CD28/HLA-DPA1/CD86/ITGAL/HLA-DRA/HLA-DR
3525	tags=65%,	IFNG/CR1/FCGR3B/IL12B/IL10/HLA-DQA1/FCGR3A/HLA-DQA2/HLA-DOA/PRKCB/HLA-
3926	tags=48%,	CCL13/CCL7/CCL8/MEFV/IL6/CCL11/CXCL8/CCL5/CCL2/IL1B/TNF/NLRP3/BIRC3/NL
1994	tags=67%,	CTSG/IFNG/FCGR3B/IL10/FCGR2B/HLA-DQA1/FCGR3A/CD80/HLA-DQA2/HLA-DOA/CD2
2397	tags=56%,	TSHR/IL10/HLA-DQA1/CD80/IL2/HLA-DQA2/HLA-DOA/CD28/HLA-DPA1/CD86/HLA-DR
2397	tags=80%,	TNFRSF17/ICOS/TNFRSF13B/IL10/HLA-DQA1/CD80/IL2/IL6/HLA-DQA2/HLA-DOA/AI
2249	tags=61%,	IFNG/IL12B/HLA-DQA1/CD80/IL2/HLA-DQA2/HLA-DOA/CD28/HLA-DPA1/CD86/HLA-D
1716	tags=23%,	IDO1/IDO2/KMO/CYP1B1/IL4I1/TDO2/KYNU/AOX1/CYP1A1
2249	tags=81%,	IFNG/KIR3DL2/HLA-DQA1/CD80/IL2/IL6/HLA-DQA2/HLA-DOA/KLRD1/KIR2DL1/KIR3
2455	tags=57%,	ICOS/TNFRSF13B/PTPRC/CD79A/AICDA/CD8A/CIITA/IL7R/BTK/CD3E/CD4/CD19/CD3
2397	tags=77%,	IFNG/IL12B/IL10/HLA-DQA1/CD80/IL2/HLA-DQA2/HLA-DOA/CD28/HLA-DPA1/CD86/
1851	tags=82%,	IL13/IL10/MS4A2/HLA-DQA1/HLA-DQA2/HLA-DOA/HLA-DPA1/HLA-DRA/HLA-DRB5/CC

CSF1R/FASLG/IGF1/WNT9B/NTRK1/GLI3/MMP2/MMP9/FGF5/SPI1/PIK3CD/COL4A4/MITF/HGF/RASSF5/FGF14/F
/C3AR1/HTR7/HRH4/NPFFR2/PTGFR/HTR2A/NPY2R/P2RX2/CYSLTR1/ADCYAP1R1/GZMA/P2RX7/MCHR1/PTAFR/CN
/IL12B/CCR8/CCL23/IL21/CCL19/CCR3/CCL1/XCR1/TNFRSF13B/CCR2/CCL21/CXCR1/CXCL10/IL2RA/IL10/CX
.6A3/COL3A1/ACTN2/PIK3CD/COMP/COL1A1/COL4A4/HGF/PARVG/PDGFRA/THBS4/COL11A1/COL5A2/COL1A2/IT
.1/XCR1/CCR2/CCL21/CXCR1/CXCL10/PIK3CG/CXCL5/CCL17/CCR7/PRKCB/CCR5/ITK/CCL22/CCL25/CXCR6/CC
R/SLC8A3/TACR1/ADRB3/GRIN2A/AVPR1A/PDGFRA/SLC8A1/PTGER3/ADCY2/DRD5/ADRA1A/CAMK4/CACNA1E/CA
.21R/IL24/CSF3R/IL2RB/IL7R/CRLF2/IL10RA/OSM/CSF2/PIK3R5/IL12RB1/LEP/OSMR/GH1/STAT4/PIK3CD/I
IR3DL1/PRKCB/KIR2DL3/ITGAL/CD48/KLRC1/CSF2/PIK3R5/LCP2/NCR3/FASLG/ITGB2/CD244/FCER1G/CD247/
)28/SIGLEC1/CD8A/HLA-DPA1/CADM3/CD86/ITGAL/HLA-DRA/CTLA4/SELP/HLA-DRB5/ITGAM/CD2/SELE/HLA-D
.DN8/MMP2/MMP9/VCAM1/ACTN2/PIK3CD/CLDN18/RASSF5/ICAM1/CXCR4/PECAM1/THY1/JAM2/PLCG2/CXCL12/V
/PIK3R5/LCP2/CD3E/CD4/PDCD1/CD3D/PAK3/CD247/CD40LG/TNF/PIK3CD/IL5/GRAP2/ZAP70/NFATC1/AKT3/V
3R5/SPP1/CXCL11/CCL3/CCL5/TLR9/IL1B/TLR6/CTSK/TNF/PIK3CD/LY96/CD14/TLR2/AKT3/CD40/STAT1/MAP
/HLA-DRB5/FLT3/ITGAM/CD3G/CD2/CSF2/CSF1R/CD3E/CD22/CD4/CD37/ITGA4/CD1E/CD19/CD7/CD3D/HLA-DR
)L4A4/CD36/THBS4/COL11A1/COL5A2/COL1A2/ITGB7/COL6A6/LAMA2/ITGB3/COL5A1/SDC2/THBS1/VTN/COL6A
/CD8A/HLA-DPA1/KIR2DL3/CIITA/HLA-DRA/HLA-DRB5/LTA/KLRC1/HLA-DPB1/CD4/HLA-DMB/HLA-DRB1/HLA-D
NF/PIK3CD/IL5/FCER1A/AKT3/PLCG2/VAV1/MAPK10/PLA2G3/IL4/PLA2G4A/MAPK12/PLA2G5/MAPK11/LAT/FYN
)/DAPP1/PIK3AP1/NFATC1/AKT3/PLCG2/VAV1/RASGRP3/CD72/RAC2/LYN/MAPK1/CARD11/NFKBIA/MALT1/NFAT
3/PLAU/SERPINE1/THBD/C7/F2R/A2M/TFPI/PLAT/SERPINC1/CFD/VWF/CFI/F3/BDKRB1/F5/PLAUR/BDKRB2/C4
RB5/HLA-DPB1/ITGB2/HLA-DMB/HLA-DRB1/SGCG/CD40LG/PRF1/HLA-DOB/SGCD/MYH13/ICAM1/LAMA2/HLA-DMA
-DPA1/HLA-DRA/HLA-DRB5/ITGAM/PTGS2/NCF1/HLA-DPB1/ITGB2/ITGA4/HLA-DMB/NCF2/HLA-DRB1/FCGR2A/I
.RC4/CXCL1/TNFAIP3/NLRP1/MAPK10/CASP1/PSTPIP1/CXCL2/CARD9/MAPK12/MAPK11/MAPK1/NAIP/CARD6/NF
28/HLA-DPA1/CD86/HLA-DRA/HLA-DRB5/HLA-DPB1/C1QB/C1QC/ELANE/HLA-DMB/HLA-DRB1/FCGR2A/C1QA/FCG
RA/CTLA4/HLA-DRB5/HLA-DPB1/FASLG/HLA-DMB/HLA-DRB1/CGA/CD40LG/PRF1/HLA-DOB/IL5/TPO/HLA-DMA/C
ICDA/CD28/HLA-DPA1/CD86/HLA-DRA/CCL25/HLA-DRB5/HLA-DPB1/ITGA4/HLA-DMB/TNFSF13B/HLA-DRB1/CD4
)RA/HLA-DRB5/LTA/HLA-DPB1/FASLG/HLA-DMB/HLA-DRB1/IL1B/TNF/PRF1/HLA-DOB/HLA-DMA/HLA-DQB1/IL1

3DL1/CD28/HLA-DPA1/KIR2DL3/CD86/HLA-DRA/HLA-DRB5/KLRC1/HLA-DPB1/FASLG/HLA-DMB/HLA-DRB1/IL1B
3D/CD40LG/ZAP70/CD40/JAK3/TNFRSF13C/LCK/CD8B
/HLA-DRA/HLA-DRB5/HLA-DPB1/FASLG/HLA-DMB/HLA-DRB1/CD40LG/TNF/PRF1/HLA-DOB/IL5/HLA-DMA/CD40/
CL11/HLA-DPB1/HLA-DMB/FCER1G/HLA-DRB1/RNASE3/CD40LG/TNF/HLA-DOB/IL5/FCER1A/HLA-DMA/CD40/HLA

PDGFRA/GLI2/CSF2RA/TGFB3/WNT1/RUNX1T1/BIRC3/LAMA2/WNT2/AKT3/DCC/FGF2/ETS1/AR/PDGFRB/PLCG2/W
NR1/TACR1/CGA/LEP/HTR1B/NR3C1/GH1/C5AR1/P2RY8/PTGDR/CHRM4/ADRB3/GRIN2A/AVPR1A/GRIA3/PTGER3/
XCL5/IL2/IL6/CCL17/CSF2RB/IL22RA2/CCR7/IL21R/TNFRSF9/IL24/CSF3R/CCR5/IL2RB/CCL22/IL7R/CCL25
TGB7/BIRC3/COL6A6/LAMA2/PDGFC/AKT3/ITGB3/COL5A1/PDGFRB/THBS1/VTN/COL6A2/VAV1/IBSP/COL6A1/FN
CL11/PPBP/CCL4/CXCL8/PIK3R5/DOCK2/NCF1/CXCL11/CCR1/CXCR2/HCK/CCL3L3/FGR/CCL3/CCL5/CCL4L2/XC
ACNA1G/PLN/CACNA1I/ADORA2A/PDGFRB/PLCG2/SPHK1/P2RX1/CHRM5/ATP2B2/HTR2B/PDE1A/CAMK2A/NOS2/AD
IL20/IL5/IL12RB2/TPO/IL11/IL9R/IL26/CSF2RA/LIFR/AKT3/SOCS3/IFNL1/CSF3/IL22/JAK3/IL19/STAT1/
/KLRK1/TYROBP/TNF/HCST/PRF1/PIK3CD/KLRC3/SH2D1B/ZAP70/ICAM1/NFATC1/PLCG2/VAV1/LCK/FAS/MICB/
DPB1/ITGB2/NCAM2/CD22/CD4/CNTN1/PDCD1/ITGA4/HLA-DMB/CD274/CLDN8/HLA-DRB1/VCAM1/NLGN4X/CD40L
VAV1/CLDN6/MSN/CLDN11/CTNNA3/MYL2/NCF4/ACTN3/JAM3/CDH5/MAPK12/MAPK11/RAC2/TXK/MYL9/MYL7/CLD
VAV1/LCK/IL4/CD8B/MAP3K8/MAPK12/MAPK11/LAT/FYN/MAPK1/CARD11/PRKCQ
PK10/LBP/TLR3/MAP3K8/MAPK12/MAPK11/TLR4/TICAM2/MAPK1/TLR5/NFKBIA
RB1/IL1B/FCGR1A/CD1C/TNF/CD33/IL5/TPO/IL11/CD14/CD36/IL9R/CSF2RA/IL1R1/ITGB3/CD1A/CSF3/CSF1
A2/IBSP/COL6A1/FN1/ITGA5/GP1BA/TNXB/COL5A3/GP5/SV2A/COL4A6/COL4A1/LAMC3/TNN/ITGA1/ITGA8/LAM
DOB/CD74/KLRC3/HLA-DMA/IFI30/CTSL/HLA-DQB1/CD8B/KLRC2/TAP1/CTSB/HLA-C/TAP2/KIR3DL3/B2M/HSPA
N/RAC2/LYN/MAPK1/PLA2G2C
C2/FOS/PIK3R3/NFATC3/PIK3CA/SOS1/PPP3CC/CHUK/PPP3R2
1B/SERPINA5/C4A/CD55/C6
A/CD40/HLA-DQB1/CAV1/MYH10/MYH11/MYH6/FYN/RAC2/HLA-C/HLA-F/CD55/MYH15/SGCA/HLA-B/HLA-E/MYH2
IL1B/FCGR1A/TNF/HLA-DOB/C3/TGFB3/TLR2/HLA-DMA/NOS2/HLA-DQB1/STAT1/IL1A/TGFB1/IL4/NCF4/JAK2/
FKBIA/PYDC1
GR1A/C1S/CD40LG/TNF/ACTN2/HLA-DOB/GRIN2A/C1R/C3/HLA-DMA/CD40/HLA-DQB1/C7
CD40/HLA-DQB1/FAS/IL4
40LG/HLA-DOB/IL5/ITGB7/CXCR4/HLA-DMA/CXCL12/CD40/HLA-DQB1/CCR9/TNFRSF13C/PIGR/TGFB1/CCR10/I
1A/FAS

B/TNF/PRF1/HLA-DOB/HLA-DMA/HLA-DQB1/IL1A/FAS

/HLA-DQB1/FAS/IL4
A-DQB1

/NT7A/WNT5A/ZBTB16/NOS2/FGFR1/STAT1/DAPK1/HHIP/FN1/KIT/MAPK10/FGF1/BCL2/FAS/TGFB1/VEGFC/GLI
/LPAR4/DRD5/S1PR4/MC5R/P2RY14/NMUR2/ADRA1A/GRIA4/GABRB3/GRIN3A/GABRG2/OPRL1/P2RY6/ADORA2A/A
5/CD27/FLT3/CXCR6/CCL11/LTA/CRLF2/IL10RA/PPBP/OSM/CSF2/TNFSF8/CCL4/CXCL8/CSF1R/CXCL11/FASLG
N1/MYLK/CAV1/MAPK10/ITGA5/TNXB/COL5A3/BCL2/VEGFC/CAV3/CAV2/MYL2/KDR/COL4A6/FLNC/COL4A1/ACTN
CL1/CCL2/WAS/CXCL6/PIK3CD/GNB4/GNGT2/GNG2/TIAM1/PF4V1/ADCY2/CXCR4/AKT3/CXCL12/VAV1/JAK3/STA
DRB2/PDE1B/MYLK/ADCY7/GRM1/AGTR1/F2R/EDNRA/P2RX5/ATP2B3/ITPR1/CHRNA7/HTR6/NOS1/EDNRB/TRPC1/
/IL6R/SOCS1/IL4/JAK2/IL13RA2/STAT2/IL6ST/IL3RA/GHR/IL15/IL7/CTF1/PRL/IFNL3/IRF9/LIF/IL9/PIA
/RAET1E/KLRC2/NCR2/SHC4/LAT/FYN/RAC2
LG/HLA-DOB/VCAN/CLDN18/NLGN1/SPN/SELPLG/ITGB7/ICAM1/PECAM1/HLA-DMA/JAM2/SDC2/CD6/CADM1/CDH2
DN5/ESAM/ROCK1/CLDN19

./KIT/THPO/IL1A/ITGA5/IL6R/GP1BA/CD1D/GP5/IL4/CD8B/CD5/ANPEP/ITGA1/IL1R2/IL3RA/IL7/ITGA2B/C
MA4/ITGAV/ITGA10/VWF/LAMA1/ITGA2B/COL4A2/LAMB4
A6/CREB1/HLA-F

2
/MAPK12/MAPK11/TLR4/MAPK1/TGFB2

IL4

1/RARB/CTNNA3/RXRG/WNT7B/SMAD4/COL4A6/FGF9/COL4A1/CDKN2B/LAMC3/RET/WNT9A/LAMA4/ITGAV/TRAF1
DORA3/GLRB/P2RX1/CALCRL/SSTR2/CHRM5/APLNR/HTR2B/GRIK1/ADRB2/F2RL2/MC2R/GRIN3B/S1PR1/NPY5R/
/CCR1/CXCR2/IL18RAP/CCL3L3/TNFSF14/CCL3/CCL5/CCL4L2/TNFSF18/XCL1/TNFSF13B/CCL2/IL12RB1/LEP
3/LAMC3/TNN/FLT4/ITGA1/ITGA8/LAMA4/ITGAV/ITGA10/SHC4/VWF/FYN/RAC2/LAMA1/ITGA2B/COL4A2/FLT1
T1/CX3CR1/CXCL1/ADCY7/RASGRP2/CCR9/CCR10/PREX1/JAK2/CXCL2/CXCL3/STAT2/CCL14/CCR6/ELMO1/ADC
PDE1C/ADCY1/GRM5/PLCB2/GNAL/PTGER1/HTR4/CACNA1H/BDKRB1/BDKRB2/ATP2B4/RYR3/CHRM2/LHCGR/ADRB
S2/PIM1/IL12A/PIK3R3/IFNGR1/IL15RA/PIK3CA/STAT3/TSLP/STAT5A/CLCF1/PIAS3/SOS1/IFNAR2/CNTF/L

/NEGR1/CD40/HLA-DQB1/CLDN6/CNTN2/CLDN11/NCAM1/CD8B/NLGN2/CNTNAP1/JAM3/CDH5/ITGA8/ITGAV/L1C

D34

./ARNT2/WNT2B/HIF1A/RAC2/LAMA1/WNT10A/ITGA2B/NKX3-1/BMP2/FZD4/COL4A2/FZD1/MAPK1/TGFB2/LAMB4
/GRM1/AGTR1/PTGER2/CHRNA3/HCRTR1/F2R/EDNRA/P2RX5/ADORA1/PTH2R/PTGIR/MTNR1A/GRIA1/LPAR1/GRIK
?/AMHR2/CXCL6/IL1B/OSMR/TNFRSF8/GH1/CD40LG/TNF/INHBA/IL20/IL5/IL12RB2/TPO/HGF/IL11/IL18R1/T
./MAPK1/PDGFD/PARVB/LAMB4/PDGFB/MYL9
CY1/GNG8/GNG11/SHC4/PLCB2/RAC2
31/CACNA1A/CCKAR/ADCY4/CACNA1B/MYLK3/AVPR1B/GNA15/GRIN1/RYR2/BST1/TBXA2R/CAMK2B
LEPR/CBL/IFNA5/EP300/CNTFR/SOCS2

CAM/PTPRM/CD34/MADCAM1/MAG

4/FLT3LG/FGF16/PDGFB/FZD2/FOXO1/KITLG/NFKBIA/SMO/LEF1/WNT10B/EGF/FGF11/FGF12/PIAS2/FOS/FGF2
K4/GABRR2/RXFP1/GABRP/CHRNA7/LPAR3/HTR6/GRIK2/GRIK3/PRSS1/UTS2R/EDNRB/S1PR3/GRID1/GABRQ/LHB
TNFSF4/PDGFRA/IL9R/TNFRSF18/IL17A/IL26/CSF2RA/TGFB3/PF4V1/IL1R1/CXCR4/LIFR/PDGFC/IFNL1/LTB/

23/APC/PTCH2/PIK3R3/TRAF3/SMAD2/PIK3CA/STAT3/EPAS1/STAT5A/PIAS3/WNT8B/SOS1/CCNE2/LAMC1/FZD1
3/GCGR/GRM2/GLP2R/AVPR2/CHRNA1/GLP1R/GHR/CHRNB2/GRM5/TAAR1/CHRNE/NPY1R/CALCR/GRIK5/S1PR2/KI
/PDGFRB/CSF3/CD70/CXCL12/IL22/CSF1/BMPR1B/CD40/IL19/CX3CR1/CXCL1/KIT/PLEKHO2/TNFRSF6B/TNFRS

[illegible]0/FZD8/ITGB1/NFKB2/CBL/E2F2/CHUK/EP300/SMAD3/RUNX1/SUFU/EGLN1/JAK1/MAPK8/WNT16/CREBBP/NFK[illegible]

[illegible]SS1R/VIPR2/PRL/GRID2/PTGER1/HTR4/BDKRB1/F2RL3/CHRNA9/MC4R/BDKRB2/GABRG3/GABBR2/GRIA2/CHRM[illegible]

[illegible]F4/CCR9/TNFRSF13C/IL1A/IL6R/TNFSF11/FAS/TGFB1/VEGFC/CCR10/IL4/CXCL2/KDR/NGFR/FLT4/IL17B/C[illegible]

[illegible]1/EGLN3/TGFBR2/PML/APC2/TGFBR1
[illegible]/GALR1/LHCGR/P2RY1/ADRA2A/NMBR
[illegible]CL3/IL1R2/CCL14/CCR6/IL6ST/IL3RA/GHR/IL15/EDA2R/IL7/TNFSF12/RELT/CTF1/BMP2/PRL/FLT1/TGFB2/

'FLT3LG/CCL26/PDGFB/IFNL3
