# Supplementary material for: Transcriptional profile and immune infiltration in colorectal cancer reveal the significance of inducible T‐cell costimulator as a crucial immune checkpoint molecule
Source: Cancer Med. 2024 Mar 20;13(6):e7097. doi: 10.1002/cam4.7097 (PMC10952025; doi:10.1002/cam4.7097)
Supplement: Supplementary file 3 [file CAM4-13-e7097-s011.pdf]

Supplementary file 3. 12 lncRNAs and 93 mRNAs developed 215 lncRNA-mRNA relationship pairs

lncRNA	mRNA	r	p.value	qvalue
RP11-284N8	IRF4	0.8755154	1.16E-117	1.31E-113
RP11-284N8	KCNA3	0.8722175	1.01E-115	5.70E-112
RP11-284N8	FCRL5	0.8715347	2.51E-115	9.43E-112
RP11-284N8	IKZF1	0.8631891	1.12E-110	3.17E-107
RP11-284N8	LAX1	0.8580363	5.91E-108	1.33E-104
RP11-284N8	LY9	0.850898	2.33E-104	4.39E-101
RP11-284N8	SLAMF7	0.8414708	6.91E-100	1.11E-96
RP11-284N8	KLHL6	0.837468	4.47E-98	6.31E-95
RP11-750H9	C3AR1	0.8370784	6.67E-98	7.63E-95
KIAA0125	FCRL5	0.8370649	6.76E-98	7.63E-95
RP11-284N8	PTPRC	0.8361809	1.67E-97	1.71E-94
RP11-284N8	MZB1	0.8326283	5.96E-96	5.61E-93
KIAA0125	MZB1	0.82915	1.82E-94	1.58E-91
RP11-750H9	FPR3	0.8280677	5.20E-94	4.19E-91
RP11-750H9	CLEC7A	0.8224638	1.06E-91	7.96E-89
RP11-750H9	CYBB	0.8173282	1.17E-89	8.04E-87
RP6-159A1.	CSF3R	0.8172924	1.21E-89	8.04E-87
RP11-284N8	SLAMF1	0.8162464	3.10E-89	1.94E-86
RP11-750H9	CD84	0.8142007	1.92E-88	1.14E-85
KIAA0125	CD79A	0.8095128	1.15E-86	6.48E-84
RP11-284N8	FYB	0.8088088	2.10E-86	1.13E-83
RP11-284N8	CD79A	0.8060219	2.25E-85	1.15E-82
RP11-750H9	MPEG1	0.8052583	4.28E-85	2.10E-82
LINC00861	IKZF1	0.8041961	1.04E-84	4.89E-82
RP11-750H9	LILRB4	0.8039641	1.26E-84	5.70E-82
KIAA0125	IGLL5	0.8029046	3.04E-84	1.32E-81
LINC00861	ZNF831	0.7993115	5.79E-83	2.42E-80
RP11-750H9	PLEK	0.7975279	2.44E-82	9.85E-80
RP11-284N8	PIK3CG	0.7958926	9.03E-82	3.52E-79
RP11-750H9	PDCD1LG2	0.7940844	3.78E-81	1.42E-78
RP11-284N8	ZNF831	0.7937591	4.89E-81	1.78E-78
AL928768.3	MZB1	0.7886777	2.52E-79	8.82E-77
RP11-284N8	PYHIN1	0.7886448	2.58E-79	8.82E-77
RP11-750H9	SIRPB2	0.7877058	5.28E-79	1.75E-76
RP11-284N8	PRKCB	0.7874429	6.45E-79	2.08E-76
AL928768.3	JCHAIN	0.7870178	8.90E-79	2.79E-76
RP11-750H9	CSF2RB	0.786387	1.44E-78	4.36E-76
RP11-284N8	TLR10	0.7863583	1.47E-78	4.36E-76
RP11-750H9	FCGR3A	0.7860463	1.86E-78	5.37E-76
RP11-284N8	CCR4	0.7856087	2.58E-78	7.28E-76
RP11-284N8	CD28	0.7830588	1.73E-77	4.78E-75
RP11-750H9	TLR8	0.7830009	1.81E-77	4.87E-75
RP11-750H9	TLR7	0.7827314	2.21E-77	5.80E-75
RP11-750H9	SCIMP	0.7825679	2.50E-77	6.40E-75
RP11-284N8	P2RY10	0.7807816	9.31E-77	2.33E-74
RP6-159A1.	CSF2RB	0.7794942	2.38E-76	5.85E-74

RP11-750H9	FYB	0.7776851	8.85E-76	2.13E-73
LINC00861	PTPRC	0.7764605	2.14E-75	5.02E-73
LINC00861	IRF4	0.7763436	2.32E-75	5.35E-73
RP11-284N8	JCHAIN	0.7761052	2.75E-75	6.22E-73
RP11-284N8	IGLL5	0.7751219	5.56E-75	1.23E-72
RP11-750H9	PTPRC	0.7746686	7.67E-75	1.66E-72
RP11-750H9	GPNMB	0.7745992	8.06E-75	1.72E-72
RP11-284N8	MPEG1	0.7740437	1.19E-74	2.50E-72
RP11-750H9	MRC1	0.7739007	1.32E-74	2.71E-72
RP11-284N8	CSF2RB	0.7732321	2.12E-74	4.27E-72
AL928768.3	CD79A	0.7728263	2.82E-74	5.59E-72
RP11-284N8	TRAT1	0.7724765	3.61E-74	7.02E-72
RP11-284N8	SCIMP	0.7719963	5.05E-74	9.67E-72
RP11-750H9	MSR1	0.7706201	1.32E-73	2.48E-71
AC004988.1	GPNMB	0.7704315	1.51E-73	2.79E-71
RP11-284N8	NUGGC	0.7697926	2.35E-73	4.27E-71
LINC00861	KLHL6	0.76873	4.89E-73	8.76E-71
RP11-284N8	TNFRSF17	0.7677615	9.51E-73	1.68E-70
RP11-750H9	CSF3R	0.7658004	3.63E-72	6.30E-70
RP11-750H9	FCN1	0.7654486	4.60E-72	7.87E-70
LINC00861	SLAMF1	0.7648904	6.72E-72	1.13E-69
RP11-750H9	IL21R	0.7634409	1.78E-71	2.96E-69
RP11-284N8	THEMIS	0.7633634	1.88E-71	3.07E-69
RP6-159A1.	FPR1	0.7628379	2.67E-71	4.31E-69
RP11-750H9	FCGR2B	0.7616436	5.93E-71	9.42E-69
RP11-750H9	FPR1	0.7612903	7.50E-71	1.17E-68
RP11-121A8	CD84	0.7607423	1.08E-70	1.67E-68
RP6-159A1.	CLEC7A	0.7607039	1.11E-70	1.69E-68
RP11-354E1	CPA3	0.7606492	1.15E-70	1.73E-68
RP11-284N8	FCRLA	0.7603965	1.36E-70	2.01E-68
RP6-159A1.	MEFV	0.7598844	1.90E-70	2.79E-68
AC004988.1	MSR1	0.7583315	5.27E-70	7.63E-68
RP6-159A1.	PLEK	0.757744	7.74E-70	1.11E-67
LINC00861	CCR4	0.7575187	8.96E-70	1.26E-67
LINC00861	ABCD2	0.7568689	1.37E-69	1.91E-67
RP11-284N8	FCRL3	0.7557602	2.81E-69	3.86E-67
RP11-284N8	CD38	0.7549828	4.63E-69	6.30E-67
RP11-284N8	CCR2	0.7546886	5.60E-69	7.52E-67
RP11-750H9	KLHL6	0.7542939	7.22E-69	9.58E-67
LINC00861	P2RY10	0.7537155	1.05E-68	1.37E-66
RP11-284N8	IL21R	0.7534813	1.21E-68	1.58E-66
RP6-159A1.	P2RY13	0.7529037	1.76E-68	2.25E-66
KIAA0125	IRF4	0.7521963	2.75E-68	3.49E-66
RP11-284N8	STAP1	0.75153	4.20E-68	5.27E-66
RP11-750H9	GPR141	0.7505513	7.80E-68	9.68E-66
RP11-750H9	CCR2	0.7504966	8.08E-68	9.91E-66
RP11-284N8	ABCD2	0.7504629	8.25E-68	1.00E-65
RP11-284N8	P2RY13	0.7500692	1.06E-67	1.27E-65

RP11-750H9	IL2RA	0.7497691	1.28E-67	1.52E-65
RP11-750H9	P2RY13	0.7495822	1.44E-67	1.69E-65
KIAA0125	JCHAIN	0.7495606	1.45E-67	1.69E-65
RP11-121A8	PTPRC	0.749482	1.53E-67	1.76E-65
RP11-284N8	SH2D1A	0.7489754	2.10E-67	2.39E-65
RP11-284N8	FCRL2	0.7488858	2.22E-67	2.51E-65
LINC00861	PRKCB	0.7487918	2.35E-67	2.63E-65
RP11-121A8	FPR3	0.7486561	2.56E-67	2.84E-65
RP11-750H9	PIK3CG	0.7481508	3.51E-67	3.85E-65
RP11-750H9	IKZF1	0.7479975	3.86E-67	4.19E-65
RP5-887A10	MS4A1	0.7477358	4.55E-67	4.89E-65
KIAA0125	TNFRSF17	0.7476909	4.68E-67	4.98E-65
AL928768.3	IGLL5	0.7471005	6.75E-67	7.12E-65
RP11-284N8	PNOC	0.7465541	9.47E-67	9.89E-65
RP11-750H9	HRH2	0.7464701	9.97E-67	1.03E-64
RP11-121A8	PDCD1LG2	0.74607	1.28E-66	1.31E-64
LINC00861	THEMIS	0.7456419	1.66E-66	1.69E-64
RP6-159A1.	PTPRC	0.7451569	2.24E-66	2.26E-64
AL928768.3	FCRL5	0.7451135	2.30E-66	2.30E-64
LINC00861	KCNA3	0.7449873	2.49E-66	2.46E-64
LINC00861	LY9	0.7443443	3.69E-66	3.62E-64
LINC00861	TLR10	0.7441643	4.12E-66	4.01E-64
RP11-121A8	C3AR1	0.7441185	4.23E-66	4.06E-64
RP11-121A8	CYBB	0.7441128	4.25E-66	4.06E-64
RP6-159A1.	SELL	0.7437795	5.21E-66	4.94E-64
LINC00861	LAX1	0.7434769	6.27E-66	5.89E-64
RP11-121A8	MPEG1	0.7424238	1.19E-65	1.11E-63
RP11-750H9	HLA-DOA	0.7423361	1.25E-65	1.16E-63
RP11-750H9	LILRA6	0.742081	1.46E-65	1.34E-63
RP11-284N8	GPR174	0.7410127	2.79E-65	2.54E-63
LINC00861	FYB	0.7408317	3.11E-65	2.81E-63
RP11-121A8	FYB	0.7401924	4.57E-65	4.10E-63
AL928768.3	TNFRSF13B	0.7399851	5.18E-65	4.60E-63
RP11-284N8	CCR7	0.7391768	8.40E-65	7.41E-63
RP11-121A8	IKZF1	0.7391282	8.65E-65	7.57E-63
LINC00861	CCR7	0.7384892	1.27E-64	1.10E-62
RP11-284N8	TLR7	0.7373544	2.49E-64	2.14E-62
RP11-284N8	AMPD1	0.7366076	3.87E-64	3.31E-62
LINC00861	SLAMF7	0.7365374	4.03E-64	3.42E-62
RP11-284N8	MS4A1	0.7353153	8.28E-64	6.97E-62
RP11-284N8	CR1	0.7350552	9.65E-64	8.06E-62
RP11-284N8	SELL	0.7343876	1.43E-63	1.18E-61
RP11-284N8	CYBB	0.7337505	2.07E-63	1.70E-61
LINC00861	PYHIN1	0.7336902	2.14E-63	1.75E-61
RP11-750H9	TFEC	0.7335855	2.28E-63	1.85E-61
RP6-159A1.	MPEG1	0.7333865	2.56E-63	2.06E-61
RP11-354E1	MS4A2	0.7332006	2.85E-63	2.28E-61
KIAA0125	FCRL2	0.7330724	3.07E-63	2.44E-61

RP11-284N8	TIGIT	0.7330622	3.09E-63	2.44E-61
AL928768.3	TNFRSF17	0.7321884	5.13E-63	4.02E-61
RP5-887A10	CD79A	0.7317404	6.64E-63	5.17E-61
AC004988.1	FCGR3A	0.7307785	1.16E-62	8.94E-61
RP11-121A8	P2RY13	0.7304358	1.41E-62	1.08E-60
RP6-159A1.	CD209	0.7300241	1.78E-62	1.36E-60
RP6-159A1.	SIRPB2	0.7299605	1.85E-62	1.40E-60
RP11-284N8	PLA2G2D	0.7294835	2.43E-62	1.83E-60
RP11-121A8	CLEC7A	0.7293838	2.58E-62	1.92E-60
RP11-284N8	HLA-DOA	0.7289591	3.28E-62	2.44E-60
RP11-284N8	TIFAB	0.7288065	3.58E-62	2.64E-60
LINC00861	MPEG1	0.7284381	4.42E-62	3.24E-60
RP11-284N8	CD84	0.7280082	5.64E-62	4.11E-60
RP11-121A8	LILRB4	0.7278089	6.32E-62	4.57E-60
RP11-284N8	GZMK	0.7275742	7.22E-62	5.19E-60
LINC00861	P2RY13	0.7275227	7.43E-62	5.31E-60
LINC00861	CD28	0.7275038	7.51E-62	5.33E-60
RP11-750H9	GBP5	0.7274818	7.60E-62	5.36E-60
RP11-121A8	SCIMP	0.7269408	1.03E-61	7.24E-60
RP11-121A8	TFEC	0.7257265	2.05E-61	1.43E-59
RP6-159A1.	FCN1	0.7250563	2.99E-61	2.07E-59
RP11-750H9	CR1	0.7248361	3.38E-61	2.32E-59
LINC00861	TRAT1	0.722843	1.03E-60	7.02E-59
KIAA0125	LAX1	0.7227982	1.05E-60	7.16E-59
RP5-887A10	FCRL1	0.7214997	2.16E-60	1.46E-58
RP11-121A8	PLEK	0.7214099	2.27E-60	1.53E-58
RP11-284N8	CD226	0.7213286	2.38E-60	1.59E-58
RP11-750H9	LILRB5	0.7210541	2.76E-60	1.83E-58
RP11-121A8	CSF2RB	0.7209046	3.00E-60	1.98E-58
RP11-284N8	GBP5	0.7206978	3.36E-60	2.21E-58
RP6-159A1.	KLHL6	0.7198155	5.46E-60	3.56E-58
RP6-159A1.	MRC1	0.718736	9.85E-60	6.39E-58
RP11-284N8	ICOS	0.7185131	1.11E-59	7.17E-58
RP5-887A10	BLK	0.7184581	1.15E-59	7.35E-58
RP11-750H9	KCNJ15	0.717908	1.55E-59	9.86E-58
RP11-750H9	CCR4	0.7178497	1.60E-59	1.01E-57
RP11-344B5	GPNMB	0.7178008	1.64E-59	1.03E-57
RP11-121A8	TLR8	0.7173313	2.11E-59	1.33E-57
KIAA0125	PNOC	0.7167635	2.88E-59	1.79E-57
KIAA0125	FCRLA	0.7166587	3.04E-59	1.89E-57
KIAA0125	LY9	0.7163392	3.62E-59	2.23E-57
LINC00861	PIK3CG	0.715979	4.39E-59	2.69E-57
LINC00861	FCRL3	0.7154408	5.87E-59	3.58E-57
RP11-121A8	SIRPB2	0.7152363	6.55E-59	3.97E-57
LINC00861	FCRL5	0.7150564	7.22E-59	4.34E-57
RP11-750H9	CD209	0.715051	7.24E-59	4.34E-57
RP11-13P5.	COL10A1	0.7149238	7.75E-59	4.63E-57
RP6-159A1.	CYBB	0.7145656	9.39E-59	5.58E-57

RP11-750H9	CD28	0.7138362	1.39E-58	8.20E-57
RP11-750H9	SIGLEC1	0.7133314	1.82E-58	1.07E-56
RP11-750H9	SLAMF1	0.7122883	3.16E-58	1.85E-56
LINC00861	SCIMP	0.7121197	3.46E-58	2.01E-56
AC004988.1	CCL18	0.7117055	4.31E-58	2.49E-56
RP11-750H9	CD80	0.7115856	4.59E-58	2.64E-56
RP11-284N8	IL2RA	0.710899	6.59E-58	3.78E-56
RP11-284N8	TNFRSF13B	0.7094377	1.42E-57	8.10E-56
RP11-121A8	TLR7	0.7093132	1.52E-57	8.61E-56
RP6-159A1.	IKZF1	0.709271	1.55E-57	8.75E-56
RP6-159A1.	CR1	0.7086469	2.15E-57	1.21E-55
RP11-121A8	CCR2	0.7081352	2.81E-57	1.57E-55
KIAA0125	SLAMF7	0.7080052	3.01E-57	1.67E-55
RP11-121A8	KLHL6	0.7078791	3.21E-57	1.78E-55
RP6-159A1.	TLR8	0.7062085	7.64E-57	4.20E-55
KIAA0125	NUGGC	0.7054662	1.12E-56	6.14E-55
LINC00861	TIGIT	0.7051994	1.29E-56	7.01E-55
RP6-159A1.	PIK3CG	0.7051843	1.30E-56	7.03E-55
RP11-284N8	PLEK	0.7047969	1.58E-56	8.54E-55
LINC00861	ICOS	0.7045575	1.79E-56	9.61E-55
RP11-750H9	CYSLTR2	0.7029403	4.09E-56	2.19E-54
RP11-284N8	CLEC7A	0.7022343	5.86E-56	3.12E-54
LINC00861	PLA2G2D	0.7016972	7.70E-56	4.08E-54
RP11-750H9	SLAMF7	0.7008091	1.21E-55	6.36E-54
LINC00861	CCR2	0.7003923	1.49E-55	7.82E-54

[illegible].
