# Supplementary material for: Transcriptional profile and immune infiltration in colorectal cancer reveal the significance of inducible T‐cell costimulator as a crucial immune checkpoint molecule
Source: Cancer Med. 2024 Mar 20;13(6):e7097. doi: 10.1002/cam4.7097 (PMC10952025; doi:10.1002/cam4.7097)
Supplement: Supplementary file 4 [file CAM4-13-e7097-s004.pdf]

Supplementary file 4. Further prediction of miRNA-lncRNA interactions generated 169 lncRNA

	lnc	miRNA	score
23452	RP11-750H9	hsa-miR-19	0.9987905
45067	RP11-750H9	hsa-miR-14	0.9944836
52893	RP11-354E1	hsa-miR-30	0.9925551
59600	LINC00861	hsa-miR-39	0.9907617
66525	RP11-750H9	hsa-miR-14	0.9888886
67825	RP11-750H9	hsa-miR-22	0.9885431
71386	RP11-750H9	hsa-miR-12	0.9875342
76735	AC004988.1	hsa-miR-47	0.9860176
82809	LINC00861	hsa-miR-39	0.9842868
83394	LINC00861	hsa-miR-47	0.9841177
84498	LINC00861	hsa-miR-51	0.9838158
90169	LINC00861	hsa-miR-56	0.9821683
92802	RP11-121A8	hsa-miR-68	0.9814253
95312	RP11-121A8	hsa-miR-46	0.9807154
95481	RP11-354E1	hsa-miR-39	0.9806658
98725	LINC00861	hsa-miR-31	0.9797233
98846	RP11-750H9	hsa-miR-50	0.9796858
104097	RP11-121A8	hsa-miR-18	0.9781538
107815	LINC00861	hsa-miR-31	0.9770308
108904	RP11-750H9	hsa-miR-50	0.9767012
109913	LINC00861	hsa-miR-47	0.9763928
111408	RP11-750H9	hsa-miR-51	0.9759464
113225	RP11-750H9	hsa-miR-18	0.9754284
115077	RP11-121A8	hsa-miR-47	0.9748712
117085	LINC00861	hsa-miR-31	0.9742839
117131	LINC00861	hsa-miR-58	0.9742701
117400	RP11-354E1	hsa-miR-47	0.9741905
118645	RP11-750H9	hsa-miR-67	0.9738397
119348	LINC00861	hsa-miR-68	0.9736328
119693	LINC00861	hsa-miR-59	0.9735309
121335	RP11-750H9	hsa-miR-47	0.9730551
126063	LINC00861	hsa-miR-68	0.9716716
127767	LINC00861	hsa-miR-13	0.9711585
128119	LINC00861	hsa-miR-58	0.9710557
136121	RP11-121A8	hsa-miR-48	0.9686858
139464	RP11-121A8	hsa-miR-39	0.9676945
145159	LINC00861	hsa-miR-32	0.9660024
147550	AC004988.1	hsa-miR-12	0.9652926
150282	RP11-354E1	hsa-miR-68	0.9644715
151573	LINC00861	hsa-miR-67	0.9640647
151922	RP11-121A8	hsa-miR-67	0.9639525
152803	RP11-750H9	hsa-miR-65	0.9636804
156348	RP11-121A8	hsa-miR-50	0.9625969
156387	RP11-750H9	hsa-miR-47	0.9625887
159082	LINC00861	hsa-miR-75	0.9617821
159244	RP11-121A8	hsa-miR-62	0.9617285

159625	RP11-121A8	hsa-miR-43	0.9616206
160336	LINC00861	hsa-miR-47	0.9614104
161102	LINC00861	hsa-miR-39	0.961178
163512	RP11-750H9	hsa-miR-67	0.960469
163907	RP11-354E1	hsa-miR-47	0.9603473
164342	LINC00861	hsa-miR-67	0.9602192
165158	RP5-887A10	hsa-miR-47	0.9599777
165657	RP11-354E1	hsa-miR-67	0.9598334
165744	RP11-344B5	hsa-miR-18	0.9598115
165820	LINC00861	hsa-miR-68	0.9597863
171197	LINC00861	hsa-miR-58	0.9581587
172015	RP5-887A10	hsa-miR-47	0.9579254
172805	RP11-354E1	hsa-miR-67	0.9576849
174950	RP11-354E1	hsa-miR-50	0.9570574
176765	RP11-750H9	hsa-miR-12	0.9565452
179227	RP11-750H9	hsa-miR-76	0.9558251
182374	RP5-887A10	hsa-miR-36	0.9549081
182568	AC004988.1	hsa-miR-44	0.9548467
182780	RP5-887A10	hsa-miR-36	0.9547859
184353	AC004988.1	hsa-miR-11	0.9543216
186175	RP11-121A8	hsa-miR-49	0.9537954
186726	LINC00861	hsa-miR-67	0.9536395
187053	AC004988.1	hsa-miR-19	0.9535315
189892	RP11-354E1	hsa-miR-80	0.9527171
190138	RP5-887A10	hsa-miR-47	0.952651
195501	RP5-887A10	hsa-miR-46	0.9511155
195822	RP11-750H9	hsa-miR-68	0.9510266
196591	RP5-887A10	hsa-miR-46	0.9508214
198330	RP11-121A8	hsa-miR-42	0.9503326
198386	RP11-750H9	hsa-miR-44	0.9503155
201936	AC004988.1	hsa-miR-19	0.9492849
202487	LINC00861	hsa-miR-37	0.9491435
204246	RP11-354E1	hsa-miR-30	0.9486467
206922	LINC00861	hsa-miR-67	0.9479047
208009	RP11-354E1	hsa-miR-65	0.9476055
210573	LINC00861	hsa-miR-65	0.9469004
212604	LINC00861	hsa-miR-71	0.9463216
213623	LINC00861	hsa-miR-39	0.9460407
213832	RP11-354E1	hsa-miR-30	0.9459845
221839	LINC00861	hsa-miR-58	0.9437497
222771	RP5-887A10	hsa-miR-46	0.9434819
225606	AC004988.1	hsa-miR-67	0.9427181
226454	RP11-750H9	hsa-miR-67	0.9424838
228831	RP11-750H9	hsa-miR-61	0.941811
229185	LINC00861	hsa-miR-46	0.941716
230778	LINC00861	hsa-miR-71	0.9412736
232265	RP11-750H9	hsa-miR-12	0.9408723
233422	RP11-121A8	hsa-miR-50	0.9405449

235787	LINC00861	hsa-miR-71	0.939882
236573	RP11-354E1	hsa-miR-47	0.9396695
239100	LINC00861	hsa-miR-65	0.938972
248482	RP11-354E1	hsa-miR-47	0.9363776
248718	LINC00861	hsa-miR-94	0.9363081
249152	RP11-121A8	hsa-miR-94	0.9361918
249153	LINC00861	hsa-miR-31	0.9361916
251099	RP11-354E1	hsa-miR-67	0.9356807
251473	LINC00861	hsa-miR-31	0.9355718
255420	RP11-750H9	hsa-miR-32	0.9345057
255700	RP11-750H9	hsa-miR-39	0.934441
257362	LINC00861	hsa-miR-67	0.9339924
257628	RP11-121A8	hsa-miR-52	0.9339233
257755	LINC00861	hsa-miR-45	0.9338878
258427	LINC00861	hsa-miR-47	0.9337001
259751	LINC00861	hsa-miR-67	0.9333418
259759	AC004988.1	hsa-miR-68	0.9333402
261194	RP5-887A10	hsa-miR-12	0.9329489
266750	RP11-750H9	hsa-miR-68	0.931424
269463	RP5-887A10	hsa-miR-60	0.9306856
275751	LINC00861	hsa-miR-30	0.9289918
276072	RP5-887A10	hsa-miR-60	0.9288995
277875	RP5-887A10	hsa-miR-60	0.9284278
281206	RP11-121A8	hsa-miR-59	0.9275477
281596	LINC00861	hsa-miR-55	0.9274353
284488	RP11-750H9	hsa-miR-57	0.9266694
287045	RP5-887A10	hsa-miR-37	0.9259966
287635	LINC00861	hsa-miR-57	0.9258492
287699	RP5-887A10	hsa-miR-37	0.9258302
287816	RP5-887A10	hsa-miR-37	0.9258024
289109	LINC00861	hsa-miR-43	0.9254559
289256	AC004988.1	hsa-miR-61	0.9254157
289320	LINC00861	hsa-miR-31	0.9253997
289367	RP11-750H9	hsa-miR-75	0.9253819
290047	LINC00861	hsa-miR-57	0.9252029
290728	RP11-354E1	hsa-miR-50	0.9250202
294000	LINC00861	hsa-miR-89	0.9241595
294083	RP11-750H9	hsa-miR-76	0.9241433
296071	LINC00861	hsa-miR-89	0.9236272
297100	RP11-121A8	hsa-miR-20	0.9233683
299889	LINC00861	hsa-miR-44	0.922646
299938	AC004988.1	hsa-miR-14	0.9226332
301238	RP11-354E1	hsa-miR-65	0.9222914
301340	LINC00861	hsa-miR-71	0.9222611
302427	RP11-750H9	hsa-miR-32	0.9219751
304996	RP5-887A10	hsa-miR-47	0.9213095
306531	RP5-887A10	hsa-miR-47	0.9209185
307327	LINC00861	hsa-miR-57	0.9207188

312201	AC004988.1	hsa-miR-67	0.9194644
314588	LINC00861	hsa-miR-67	0.9188537
314961	RP11-750H9	hsa-miR-46	0.9187666
316361	RP11-354E1	hsa-miR-39	0.918387
316692	RP11-354E1	hsa-miR-54	0.918301
318133	LINC00861	hsa-miR-31	0.9179437
318297	RP11-354E1	hsa-miR-51	0.9179014
318298	RP11-354E1	hsa-miR-51	0.9179014
318873	RP11-121A8	hsa-miR-20	0.917749
319942	AC004988.1	hsa-miR-56	0.9174801
324343	RP11-121A8	hsa-miR-48	0.9163644
326493	AC004988.1	hsa-miR-36	0.9158159
328269	LINC00861	hsa-miR-44	0.9153603
328328	RP11-121A8	hsa-miR-58	0.9153422
328485	LINC00861	hsa-miR-65	0.9153032
330851	RP11-750H9	hsa-miR-43	0.9146963
331820	LINC00861	hsa-miR-67	0.914448
332185	LINC00861	hsa-miR-14	0.9143577
332475	LINC00861	hsa-miR-31	0.9142823
333600	LINC00861	hsa-miR-59	0.9140054
334308	RP11-121A8	hsa-miR-54	0.91383
334770	LINC00861	hsa-miR-13	0.9137104
334819	AC004988.1	hsa-miR-19	0.9137006
337304	AC004988.1	hsa-miR-18	0.9130803
337652	RP5-887A10	hsa-miR-47	0.912996
337653	RP5-887A10	hsa-miR-31	0.912996
340093	RP5-887A10	hsa-miR-47	0.9123804
340094	RP5-887A10	hsa-miR-31	0.9123804
341845	AC004988.1	hsa-miR-42	0.9119391
344548	RP5-887A10	hsa-miR-47	0.9113005
345088	RP11-13P5.	hsa-miR-37	0.911166
345340	RP11-13P5.	hsa-miR-50	0.9111078
345665	RP11-354E1	hsa-miR-67	0.9110238
346726	RP11-354E1	hsa-miR-66	0.9107559
354219	AC004988.1	hsa-miR-67	0.9089061
356533	RP11-121A8	hsa-miR-65	0.9083429
356703	RP11-354E1	hsa-miR-57	0.9082977
356707	RP11-121A8	hsa-miR-60	0.9082973
358222	RP11-121A8	hsa-miR-39	0.9079206
358490	LINC00861	hsa-miR-31	0.9078593
359275	LINC00861	hsa-miR-67	0.9076607
360737	RP11-13P5.	hsa-miR-51	0.9073142
362456	LINC00861	hsa-miR-50	0.9069177
362483	RP11-750H9	hsa-miR-45	0.9069122
363430	RP11-750H9	hsa-miR-39	0.9066817
365300	LINC00861	hsa-miR-68	0.9062382
365519	AC004988.1	hsa-miR-31	0.9061822
367189	LINC00861	hsa-miR-14	0.9057853

367203	RP11-121A8	hsa-miR-22	0.9057825
367450	RP11-354E1	hsa-miR-54	0.9057154
367523	RP11-13P5.	hsa-miR-14	0.9056935
369132	RP11-750H9	hsa-miR-46	0.9052955
369616	AC004988.1	hsa-miR-43	0.905175
371835	RP11-121A8	hsa-miR-50	0.9046283
373419	RP11-13P5.	hsa-miR-12	0.9042392
373894	LINC00861	hsa-miR-80	0.904129
374961	RP11-121A8	hsa-miR-40	0.9038692
378421	LINC00861	hsa-miR-47	0.90302
379563	RP11-750H9	hsa-miR-44	0.9027644
379814	LINC00861	hsa-miR-37	0.9027135
380224	AC004988.1	hsa-miR-33	0.9026136
382689	LINC00861	hsa-miR-57	0.9020327
385733	RP11-750H9	hsa-miR-47	0.9013166
385989	RP11-750H9	hsa-miR-39	0.9012594
386312	RP11-121A8	hsa-miR-67	0.9011787
386762	RP11-13P5.	hsa-miR-20	0.9010671
388686	RP11-750H9	hsa-miR-75	0.9006274
389253	LINC00861	hsa-miR-12	0.9004918
390688	LINC00861	hsa-miR-31	0.9001589

-miRNA relationship pairs which include 8 lncRNAs and 160 miRNAs.
