# Supplementary material for: Transcriptional profile and immune infiltration in colorectal cancer reveal the significance of inducible T‐cell costimulator as a crucial immune checkpoint molecule
Source: Cancer Med. 2024 Mar 20;13(6):e7097. doi: 10.1002/cam4.7097 (PMC10952025; doi:10.1002/cam4.7097)
Supplement: Supplementary file 5 [file CAM4-13-e7097-s012.pdf]

Supplementary file 5. miRNAs involved in the regulation of mRNAs. 3,297 miRNA-mRNA relatic

Gene	miRNA
ABCD2	hsa-miR-491-3p
ABCD2	hsa-miR-508-3p
ABCD2	hsa-miR-548c-3p
ABCD2	hsa-miR-548g-3p
ABCD2	hsa-miR-548k
ABCD2	hsa-miR-561-3p
ABCD2	hsa-miR-567
ABCD2	hsa-miR-579-3p
ABCD2	hsa-miR-588
ABCD2	hsa-miR-590-3p
ABCD2	hsa-miR-607
ABCD2	hsa-miR-1305
ABCD2	hsa-miR-656-3p
ABCD2	hsa-miR-137
ABCD2	hsa-miR-142-5p
ABCD2	hsa-miR-889-3p
ABCD2	hsa-miR-944
ABCD2	hsa-miR-155-5p
ABCD2	hsa-miR-182-5p
ABCD2	hsa-miR-106b-5p
ABCD2	hsa-miR-203a
ABCD2	hsa-miR-205-5p
ABCD2	hsa-miR-20a-5p
ABCD2	hsa-miR-297
ABCD2	hsa-miR-340-5p
ABCD2	hsa-miR-421
BLK	hsa-miR-486-3p
BLK	hsa-miR-512-3p
BLK	hsa-miR-1283
BLK	hsa-miR-541-3p
BLK	hsa-miR-1287-5p
BLK	hsa-miR-1289
BLK	hsa-miR-615-5p
BLK	hsa-miR-622
BLK	hsa-miR-645
BLK	hsa-miR-663a
BLK	hsa-miR-744-5p
BLK	hsa-miR-937-3p
BLK	hsa-miR-22-3p
BLK	hsa-miR-3164
BLK	hsa-miR-324-3p
BLK	hsa-miR-342-5p
BLK	hsa-miR-370-3p
C3AR1	hsa-miR-767-3p
C3AR1	hsa-miR-1178-3p
C3AR1	hsa-miR-1287-5p

C3AR1	hsa-miR-580-3p
C3AR1	hsa-miR-589-5p
C3AR1	hsa-miR-769-3p
C3AR1	hsa-miR-3148
C3AR1	hsa-miR-1225-3p
CCL18	hsa-miR-579-3p
CCL18	hsa-miR-586
CCL18	hsa-miR-149-5p
CCL18	hsa-miR-24-3p
CCL18	hsa-miR-299-5p
CCR2	hsa-miR-487a-3p
CCR2	hsa-miR-150-5p
CCR2	hsa-miR-2115-3p
CCR4	hsa-miR-1255a
CCR4	hsa-miR-320a
CCR4	hsa-miR-320b
CCR4	hsa-miR-320c
CCR4	hsa-miR-320d
CCR7	hsa-let-7e-5p
CCR7	hsa-let-7f-5p
CCR7	hsa-miR-616-3p
CCR7	hsa-miR-646
CCR7	hsa-miR-98-5p
CCR7	hsa-let-7a-5p
CCR7	hsa-miR-21-5p
CCR7	hsa-miR-298
CCR7	hsa-let-7b-5p
CCR7	hsa-let-7c-5p
CCR7	hsa-miR-432-5p
CCR7	hsa-miR-1267
CCR7	hsa-miR-483-3p
CCR7	hsa-miR-518c-5p
CCR7	hsa-let-7g-5p
CCR7	hsa-miR-532-3p
CCR7	hsa-miR-542-3p
CCR7	hsa-let-7i-5p
CCR7	hsa-miR-129-5p
CCR7	hsa-miR-574-5p
CCR7	hsa-miR-1296-5p
CCR7	hsa-miR-589-3p
CCR7	hsa-miR-1298-5p
CCR7	hsa-miR-623
CCR7	hsa-miR-636
CCR7	hsa-miR-642a-5p
CCR7	hsa-miR-644a
CCR7	hsa-miR-650
CCR7	hsa-miR-658
CCR7	hsa-miR-659-3p

CCR7	hsa-miR-661
CCR7	hsa-miR-767-3p
CCR7	hsa-miR-147a
CCR7	hsa-miR-1914-3p
CCR7	hsa-miR-199a-5p
CCR7	hsa-miR-199b-5p
CCR7	hsa-miR-211-5p
CCR7	hsa-miR-30b-3p
CCR7	hsa-miR-30c-1-3p
CCR7	hsa-miR-30c-2-3p
CCR7	hsa-miR-3140-3p
CCR7	hsa-miR-3160-3p
CCR7	hsa-miR-3176
CCR7	hsa-miR-320a
CCR7	hsa-miR-320b
CCR7	hsa-miR-320c
CCR7	hsa-miR-320d
CCR7	hsa-miR-339-5p
CCR7	hsa-miR-3663-5p
CCR7	hsa-miR-3689a-3p
CCR7	hsa-miR-3689b-3p
CCR7	hsa-let-7d-5p
CCR7	hsa-miR-409-3p
CD209	hsa-miR-1252-5p
CD209	hsa-miR-1287-5p
CD209	hsa-miR-576-3p
CD209	hsa-miR-596
CD209	hsa-miR-342-5p
CD209	hsa-miR-1228-3p
CD209	hsa-miR-1254
CD209	hsa-miR-1265
CD209	hsa-miR-490-3p
CD209	hsa-miR-1275
CD209	hsa-miR-508-5p
CD209	hsa-miR-515-5p
CD209	hsa-miR-1283
CD209	hsa-miR-539-5p
CD209	hsa-miR-1286
CD209	hsa-miR-1289
CD209	hsa-miR-548k
CD209	hsa-miR-557
CD209	hsa-miR-571
CD209	hsa-miR-583
CD209	hsa-miR-589-5p
CD209	hsa-miR-608
CD209	hsa-miR-615-5p
CD209	hsa-miR-637
CD209	hsa-miR-646

CD209	hsa-miR-650
CD209	hsa-miR-663a
CD209	hsa-miR-671-5p
CD209	hsa-miR-708-5p
CD209	hsa-miR-744-5p
CD209	hsa-miR-765
CD209	hsa-miR-766-3p
CD209	hsa-miR-769-5p
CD209	hsa-miR-873-5p
CD209	hsa-miR-891b
CD209	hsa-miR-940
CD209	hsa-miR-182-5p
CD209	hsa-miR-1827
CD209	hsa-miR-185-5p
CD209	hsa-miR-186-5p
CD209	hsa-miR-188-3p
CD209	hsa-miR-216a-5p
CD209	hsa-miR-24-3p
CD209	hsa-miR-28-5p
CD209	hsa-miR-298
CD209	hsa-miR-1203
CD209	hsa-miR-31-5p
CD209	hsa-miR-3179
CD209	hsa-miR-330-5p
CD209	hsa-miR-342-3p
CD209	hsa-miR-3662
CD209	hsa-miR-1243
CD209	hsa-miR-3928-3p
CD209	hsa-miR-423-3p
CD209	hsa-miR-423-5p
CD209	hsa-miR-4257
CD226	hsa-miR-501-5p
CD226	hsa-miR-511-5p
CD226	hsa-miR-877-3p
CD226	hsa-miR-17-3p
CD226	hsa-miR-182-5p
CD226	hsa-miR-204-5p
CD226	hsa-miR-211-5p
CD28	hsa-miR-449a
CD28	hsa-miR-452-5p
CD28	hsa-miR-1264
CD28	hsa-miR-1271-5p
CD28	hsa-miR-494-3p
CD28	hsa-miR-495-3p
CD28	hsa-miR-496
CD28	hsa-miR-497-5p
CD28	hsa-miR-498
CD28	hsa-miR-499a-5p

CD28	hsa-miR-500a-5p
CD28	hsa-miR-513a-5p
CD28	hsa-miR-519d-3p
CD28	hsa-miR-520a-3p
CD28	hsa-miR-520b
CD28	hsa-miR-520c-3p
CD28	hsa-miR-1284
CD28	hsa-miR-520d-3p
CD28	hsa-miR-526b-5p
CD28	hsa-miR-545-3p
CD28	hsa-miR-548c-3p
CD28	hsa-miR-548g-3p
CD28	hsa-miR-548k
CD28	hsa-miR-556-3p
CD28	hsa-miR-570-3p
CD28	hsa-miR-574-5p
CD28	hsa-miR-579-3p
CD28	hsa-miR-583
CD28	hsa-miR-589-3p
CD28	hsa-miR-590-3p
CD28	hsa-miR-595
CD28	hsa-miR-606
CD28	hsa-miR-607
CD28	hsa-miR-1299
CD28	hsa-miR-613
CD28	hsa-miR-1
CD28	hsa-miR-616-5p
CD28	hsa-miR-629-5p
CD28	hsa-miR-633
CD28	hsa-miR-635
CD28	hsa-miR-656-3p
CD28	hsa-miR-659-3p
CD28	hsa-miR-663b
CD28	hsa-miR-101-3p
CD28	hsa-miR-136-5p
CD28	hsa-miR-142-5p
CD28	hsa-miR-144-3p
CD28	hsa-miR-708-5p
CD28	hsa-miR-758-3p
CD28	hsa-miR-888-5p
CD28	hsa-miR-93-5p
CD28	hsa-miR-96-5p
CD28	hsa-miR-155-5p
CD28	hsa-miR-15a-5p
CD28	hsa-miR-15b-5p
CD28	hsa-miR-16-5p
CD28	hsa-miR-17-5p
CD28	hsa-miR-183-5p

CD28	hsa-miR-106a-5p
CD28	hsa-miR-188-5p
CD28	hsa-miR-106b-5p
CD28	hsa-miR-195-5p
CD28	hsa-miR-206
CD28	hsa-miR-20a-5p
CD28	hsa-miR-20b-5p
CD28	hsa-miR-1179
CD28	hsa-miR-224-5p
CD28	hsa-miR-24-3p
CD28	hsa-miR-1183
CD28	hsa-miR-27a-3p
CD28	hsa-miR-27b-3p
CD28	hsa-miR-28-5p
CD28	hsa-miR-299-5p
CD28	hsa-miR-300
CD28	hsa-miR-1202
CD28	hsa-miR-31-5p
CD28	hsa-miR-1224-5p
CD28	hsa-miR-337-3p
CD28	hsa-miR-338-5p
CD28	hsa-miR-34a-5p
CD28	hsa-miR-1233-3p
CD28	hsa-miR-369-3p
CD28	hsa-miR-371a-5p
CD28	hsa-miR-372-3p
CD28	hsa-miR-1237-3p
CD28	hsa-miR-381-3p
CD28	hsa-miR-384
CD28	hsa-miR-425-5p
CD28	hsa-miR-1248
CD38	hsa-miR-499a-5p
CD38	hsa-miR-576-5p
CD38	hsa-miR-579-3p
CD38	hsa-miR-624-3p
CD80	hsa-miR-1276
CD80	hsa-miR-573
CD80	hsa-let-7e-5p
CD80	hsa-miR-1258
CD80	hsa-let-7f-5p
CD80	hsa-miR-497-5p
CD80	hsa-miR-511-5p
CD80	hsa-miR-517-5p
CD80	hsa-miR-518a-5p
CD80	hsa-let-7g-5p
CD80	hsa-miR-527
CD80	hsa-miR-544a
CD80	hsa-let-7i-5p

CD80	hsa-miR-586
CD80	hsa-miR-593-3p
CD80	hsa-miR-601
CD80	hsa-miR-1303
CD80	hsa-miR-628-5p
CD80	hsa-miR-630
CD80	hsa-miR-138-1-3p
CD80	hsa-miR-141-3p
CD80	hsa-miR-103a-3p
CD80	hsa-miR-921
CD80	hsa-miR-149-5p
CD80	hsa-miR-98-5p
CD80	hsa-let-7a-5p
CD80	hsa-miR-107
CD80	hsa-miR-219a-2-3p
CD80	hsa-miR-2278
CD80	hsa-miR-29a-3p
CD80	hsa-miR-29b-3p
CD80	hsa-miR-29c-3p
CD80	hsa-let-7b-5p
CD80	hsa-miR-3182
CD80	hsa-miR-3200-5p
CD80	hsa-miR-330-3p
CD80	hsa-miR-1226-3p
CD80	hsa-let-7c-5p
CD80	hsa-let-7d-5p
CD80	hsa-miR-424-5p
CD80	hsa-miR-4311
CD84	hsa-miR-1252-5p
CD84	hsa-miR-1254
CD84	hsa-miR-1256
CD84	hsa-miR-450b-5p
CD84	hsa-miR-125a-3p
CD84	hsa-miR-452-3p
CD84	hsa-miR-1264
CD84	hsa-miR-126-5p
CD84	hsa-miR-1273e
CD84	hsa-miR-485-5p
CD84	hsa-miR-488-5p
CD84	hsa-miR-496
CD84	hsa-miR-501-5p
CD84	hsa-miR-511-5p
CD84	hsa-miR-512-3p
CD84	hsa-miR-513a-3p
CD84	hsa-miR-513b-5p
CD84	hsa-miR-516b-5p
CD84	hsa-miR-517-5p
CD84	hsa-miR-518d-5p

CD84	hsa-miR-518e-5p
CD84	hsa-miR-518f-5p
CD84	hsa-miR-519a-3p
CD84	hsa-miR-519a-5p
CD84	hsa-miR-519b-3p
CD84	hsa-miR-519b-5p
CD84	hsa-miR-519c-3p
CD84	hsa-miR-519c-5p
CD84	hsa-miR-519d-3p
CD84	hsa-miR-519e-3p
CD84	hsa-miR-520a-3p
CD84	hsa-miR-520a-5p
CD84	hsa-miR-520b
CD84	hsa-miR-520c-3p
CD84	hsa-miR-520c-5p
CD84	hsa-miR-520d-3p
CD84	hsa-miR-520e
CD84	hsa-miR-520g-3p
CD84	hsa-miR-520h
CD84	hsa-miR-1285-3p
CD84	hsa-miR-522-5p
CD84	hsa-miR-523-5p
CD84	hsa-miR-525-5p
CD84	hsa-miR-526a
CD84	hsa-miR-526b-3p
CD84	hsa-miR-532-3p
CD84	hsa-miR-541-3p
CD84	hsa-miR-1286
CD84	hsa-miR-548a-3p
CD84	hsa-miR-548a-5p
CD84	hsa-miR-548b-5p
CD84	hsa-miR-548d-5p
CD84	hsa-miR-548e-3p
CD84	hsa-miR-548i
CD84	hsa-miR-548j-5p
CD84	hsa-miR-548l
CD84	hsa-miR-548x-3p
CD84	hsa-miR-551b-5p
CD84	hsa-miR-552-3p
CD84	hsa-miR-556-5p
CD84	hsa-miR-569
CD84	hsa-miR-574-5p
CD84	hsa-miR-575
CD84	hsa-miR-582-5p
CD84	hsa-miR-586
CD84	hsa-miR-590-5p
CD84	hsa-miR-597-5p
CD84	hsa-miR-600

CD84	hsa-miR-1299
CD84	hsa-miR-1301-3p
CD84	hsa-miR-612
CD84	hsa-miR-617
CD84	hsa-miR-622
CD84	hsa-miR-647
CD84	hsa-miR-648
CD84	hsa-miR-654-5p
CD84	hsa-let-7a-3p
CD84	hsa-miR-130b-5p
CD84	hsa-miR-1324
CD84	hsa-miR-138-5p
CD84	hsa-miR-142-3p
CD84	hsa-miR-143-3p
CD84	hsa-miR-145-3p
CD84	hsa-miR-708-5p
CD84	hsa-miR-103a-2-5p
CD84	hsa-miR-146a-3p
CD84	hsa-miR-7-5p
CD84	hsa-miR-760
CD84	hsa-miR-761
CD84	hsa-miR-767-3p
CD84	hsa-miR-767-5p
CD84	hsa-miR-875-3p
CD84	hsa-miR-876-3p
CD84	hsa-miR-890
CD84	hsa-miR-148b-5p
CD84	hsa-miR-921
CD84	hsa-miR-922
CD84	hsa-miR-93-5p
CD84	hsa-miR-936
CD84	hsa-miR-940
CD84	hsa-miR-942-5p
CD84	hsa-miR-9-5p
CD84	hsa-miR-150-5p
CD84	hsa-miR-155-5p
CD84	hsa-miR-15a-5p
CD84	hsa-miR-17-5p
CD84	hsa-miR-106a-5p
CD84	hsa-miR-185-5p
CD84	hsa-miR-186-3p
CD84	hsa-miR-106b-5p
CD84	hsa-miR-1911-3p
CD84	hsa-miR-1912
CD84	hsa-miR-1914-5p
CD84	hsa-miR-199a-5p
CD84	hsa-miR-199b-5p
CD84	hsa-miR-19a-5p

CD84	hsa-miR-19b-1-5p
CD84	hsa-miR-19b-2-5p
CD84	hsa-miR-205-3p
CD84	hsa-miR-20a-5p
CD84	hsa-miR-20b-3p
CD84	hsa-miR-20b-5p
CD84	hsa-miR-2115-3p
CD84	hsa-miR-2117
CD84	hsa-let-7b-3p
CD84	hsa-miR-218-5p
CD84	hsa-miR-219a-1-3p
CD84	hsa-miR-222-5p
CD84	hsa-miR-223-3p
CD84	hsa-miR-26a-1-3p
CD84	hsa-miR-26a-2-3p
CD84	hsa-miR-26b-3p
CD84	hsa-miR-28-5p
CD84	hsa-miR-300
CD84	hsa-miR-302a-3p
CD84	hsa-miR-302c-3p
CD84	hsa-miR-302d-3p
CD84	hsa-miR-30b-3p
CD84	hsa-miR-1197
CD84	hsa-miR-3116
CD84	hsa-miR-3119
CD84	hsa-miR-3123
CD84	hsa-miR-3126-3p
CD84	hsa-miR-3130-5p
CD84	hsa-miR-1200
CD84	hsa-miR-3139
CD84	hsa-miR-3148
CD84	hsa-miR-3150b-3p
CD84	hsa-miR-3151-5p
CD84	hsa-miR-3153
CD84	hsa-miR-3154
CD84	hsa-miR-3155a
CD84	hsa-miR-3157-5p
CD84	hsa-miR-3159
CD84	hsa-miR-3161
CD84	hsa-miR-3163
CD84	hsa-miR-3164
CD84	hsa-miR-3180-5p
CD84	hsa-miR-1207-5p
CD84	hsa-miR-3185
CD84	hsa-miR-3187-3p
CD84	hsa-miR-3189-3p
CD84	hsa-miR-3199
CD84	hsa-miR-328-3p

CD84	hsa-miR-331-3p
CD84	hsa-miR-335-3p
CD84	hsa-miR-33a-3p
CD84	hsa-miR-122-5p
CD84	hsa-miR-34a-3p
CD84	hsa-miR-1226-5p
CD84	hsa-miR-3614-5p
CD84	hsa-miR-3616-3p
CD84	hsa-miR-3616-5p
CD84	hsa-miR-3619-5p
CD84	hsa-miR-3622b-5p
CD84	hsa-miR-3646
CD84	hsa-miR-3653
CD84	hsa-miR-3657
CD84	hsa-miR-3663-5p
CD84	hsa-miR-3664-5p
CD84	hsa-miR-3667-3p
CD84	hsa-miR-3680-3p
CD84	hsa-miR-3684
CD84	hsa-miR-3685
CD84	hsa-miR-372-3p
CD84	hsa-miR-373-3p
CD84	hsa-miR-374a-3p
CD84	hsa-miR-1237-3p
CD84	hsa-miR-381-3p
CD84	hsa-miR-3907
CD84	hsa-miR-3908
CD84	hsa-miR-3921
CD84	hsa-miR-3929
CD84	hsa-miR-3934-5p
CD84	hsa-miR-1245a
CD84	hsa-miR-4253
CD84	hsa-miR-4260
CD84	hsa-miR-4264
CD84	hsa-miR-4269
CD84	hsa-miR-4273
CD84	hsa-miR-4277
CD84	hsa-miR-4288
CD84	hsa-miR-4298
CD84	hsa-miR-4302
CD84	hsa-miR-4306
CD84	hsa-miR-4307
CD84	hsa-miR-4308
CD84	hsa-miR-4309
CD84	hsa-miR-431-5p
CD84	hsa-miR-432-5p
CLEC7A	hsa-miR-582-5p
CLEC7A	hsa-miR-645

CLEC7A	hsa-miR-655-3p
CLEC7A	hsa-miR-101-3p
CLEC7A	hsa-miR-874-3p
CLEC7A	hsa-miR-3646
CLEC7A	hsa-miR-369-3p
CLEC7A	hsa-miR-374c-5p
COL10A1	hsa-miR-1276
COL10A1	hsa-miR-513a-3p
COL10A1	hsa-miR-561-3p
COL10A1	hsa-miR-1297
COL10A1	hsa-miR-101-3p
COL10A1	hsa-miR-26b-5p
COL10A1	hsa-miR-384
COL10A1	hsa-miR-488-3p
COL10A1	hsa-miR-511-5p
COL10A1	hsa-miR-520d-5p
COL10A1	hsa-miR-581
COL10A1	hsa-miR-586
COL10A1	hsa-miR-624-3p
COL10A1	hsa-miR-625-5p
COL10A1	hsa-miR-664a-3p
COL10A1	hsa-miR-767-5p
COL10A1	hsa-miR-1827
COL10A1	hsa-miR-23a-3p
COL10A1	hsa-miR-23b-3p
COL10A1	hsa-miR-26a-5p
COL10A1	hsa-miR-302b-5p
COL10A1	hsa-miR-302d-5p
COL10A1	hsa-miR-3119
COL10A1	hsa-miR-3200-3p
COL10A1	hsa-miR-376a-3p
COL10A1	hsa-miR-376b-3p
CPA3	hsa-miR-155-5p
CPA3	hsa-miR-432-5p
CR1	hsa-miR-544a
CR1	hsa-miR-628-3p
CR1	hsa-miR-140-5p
CSF2RB	hsa-miR-452-3p
CSF2RB	hsa-miR-548l
CSF2RB	hsa-miR-548n
CSF2RB	hsa-miR-548t-5p
CSF2RB	hsa-miR-596
CSF2RB	hsa-miR-671-5p
CSF2RB	hsa-miR-181a-5p
CSF2RB	hsa-miR-181c-5p
CSF2RB	hsa-miR-19a-3p
CSF2RB	hsa-miR-19b-3p
CSF2RB	hsa-miR-2355-5p

CSF2RB	hsa-miR-3167
CSF3R	hsa-miR-671-5p
CSF3R	hsa-miR-30b-3p
CSF3R	hsa-miR-3689a-3p
CSF3R	hsa-miR-3689b-3p
CYBB	hsa-miR-1267
CYBB	hsa-miR-486-3p
CYBB	hsa-miR-496
CYBB	hsa-miR-127-5p
CYBB	hsa-miR-520c-3p
CYBB	hsa-miR-520d-3p
CYBB	hsa-miR-596
CYBB	hsa-miR-608
CYBB	hsa-miR-1303
CYBB	hsa-miR-653-5p
CYBB	hsa-miR-1323
CYBB	hsa-miR-138-5p
CYBB	hsa-miR-758-3p
CYBB	hsa-miR-147a
CYBB	hsa-miR-874-3p
CYBB	hsa-miR-9-5p
CYBB	hsa-miR-106a-5p
CYBB	hsa-miR-186-5p
CYBB	hsa-miR-205-5p
CYBB	hsa-miR-27a-3p
CYBB	hsa-miR-27b-3p
CYBB	hsa-miR-298
CYBB	hsa-miR-302d-3p
CYBB	hsa-miR-302e
CYBB	hsa-miR-1207-5p
CYBB	hsa-miR-372-3p
CYBB	hsa-miR-374b-5p
CYBB	hsa-miR-1243
CYBB	hsa-miR-425-5p
CYBB	hsa-miR-1270
CYBB	hsa-miR-1271-5p
CYBB	hsa-miR-1272
CYBB	hsa-miR-513a-3p
CYBB	hsa-miR-128-3p
CYBB	hsa-miR-519c-3p
CYBB	hsa-miR-520a-3p
CYBB	hsa-miR-520b
CYBB	hsa-miR-1284
CYBB	hsa-miR-520e
CYBB	hsa-miR-526b-5p
CYBB	hsa-miR-1286
CYBB	hsa-miR-548a-3p
CYBB	hsa-miR-548aa

CYBB	hsa-miR-548e-3p
CYBB	hsa-miR-548f-3p
CYBB	hsa-miR-129-5p
CYBB	hsa-miR-574-5p
CYBB	hsa-miR-582-3p
CYBB	hsa-miR-588
CYBB	hsa-miR-589-3p
CYBB	hsa-miR-1299
CYBB	hsa-miR-1304-5p
CYBB	hsa-miR-645
CYBB	hsa-miR-647
CYBB	hsa-miR-655-3p
CYBB	hsa-miR-664a-3p
CYBB	hsa-miR-130b-5p
CYBB	hsa-miR-143-5p
CYBB	hsa-miR-145-5p
CYBB	hsa-miR-769-5p
CYBB	hsa-miR-146b-3p
CYBB	hsa-miR-148a-3p
CYBB	hsa-miR-875-3p
CYBB	hsa-miR-875-5p
CYBB	hsa-miR-885-3p
CYBB	hsa-miR-148b-3p
CYBB	hsa-miR-92a-2-5p
CYBB	hsa-miR-935
CYBB	hsa-miR-938
CYBB	hsa-miR-944
CYBB	hsa-miR-150-5p
CYBB	hsa-miR-152-3p
CYBB	hsa-miR-182-3p
CYBB	hsa-miR-182-5p
CYBB	hsa-miR-1827
CYBB	hsa-miR-200b-3p
CYBB	hsa-miR-200c-3p
CYBB	hsa-miR-204-5p
CYBB	hsa-miR-20b-5p
CYBB	hsa-miR-211-5p
CYBB	hsa-miR-219a-2-3p
CYBB	hsa-miR-26b-3p
CYBB	hsa-miR-29b-1-5p
CYBB	hsa-miR-29b-2-5p
CYBB	hsa-miR-302a-3p
CYBB	hsa-miR-302b-3p
CYBB	hsa-miR-302c-3p
CYBB	hsa-miR-30c-1-3p
CYBB	hsa-miR-30c-2-3p
CYBB	hsa-miR-3148
CYBB	hsa-miR-3151-5p

CYBB	hsa-miR-3162-5p
CYBB	hsa-miR-323a-3p
CYBB	hsa-miR-34c-3p
CYBB	hsa-miR-361-3p
CYBB	hsa-miR-3658
CYBB	hsa-miR-3671
CYBB	hsa-miR-3679-3p
CYBB	hsa-miR-3682-3p
CYBB	hsa-miR-369-3p
CYBB	hsa-miR-373-3p
CYBB	hsa-miR-374a-5p
CYBB	hsa-miR-1238-3p
CYBB	hsa-miR-4277
CYBB	hsa-miR-4282
CYBB	hsa-miR-4287
CYBB	hsa-miR-429
CYBB	hsa-miR-432-5p
CYSLTR2	hsa-miR-500a-5p
CYSLTR2	hsa-miR-1290
CYSLTR2	hsa-miR-141-5p
CYSLTR2	hsa-miR-920
CYSLTR2	hsa-miR-936
CYSLTR2	hsa-miR-939-5p
CYSLTR2	hsa-miR-1184
CYSLTR2	hsa-miR-3122
CYSLTR2	hsa-miR-3150a-3p
CYSLTR2	hsa-miR-3614-3p
CYSLTR2	hsa-miR-4300
FCGR2B	hsa-miR-1253
FCGR2B	hsa-miR-644a
FCGR2B	hsa-miR-3185
FCGR2B	hsa-miR-3911
FCGR3A	hsa-miR-1261
FCGR3A	hsa-miR-1270
FCGR3A	hsa-miR-520a-5p
FCGR3A	hsa-miR-525-5p
FCGR3A	hsa-miR-556-3p
FCGR3A	hsa-miR-1292-5p
FCGR3A	hsa-miR-620
FCGR3A	hsa-miR-636
FCGR3A	hsa-miR-145-5p
FCGR3A	hsa-miR-7-5p
FCGR3A	hsa-miR-890
FCGR3A	hsa-miR-149-5p
FCGR3A	hsa-miR-218-5p
FCGR3A	hsa-miR-330-5p
FCGR3A	hsa-miR-375
FCGR3A	hsa-miR-378a-3p

FCGR3A	hsa-miR-409-3p
FCGR3A	hsa-miR-422a
FCN1	hsa-miR-3622b-5p
FCRL1	hsa-miR-600
FCRL1	hsa-miR-936
FCRL1	hsa-miR-1827
FCRL1	hsa-miR-27a-3p
FCRL1	hsa-miR-27b-3p
FCRL1	hsa-miR-490-5p
FCRL1	hsa-miR-515-3p
FCRL1	hsa-miR-1285-3p
FCRL1	hsa-miR-550a-5p
FCRL1	hsa-miR-561-3p
FCRL1	hsa-miR-576-5p
FCRL1	hsa-miR-590-3p
FCRL1	hsa-miR-661
FCRL1	hsa-miR-665
FCRL1	hsa-miR-758-3p
FCRL1	hsa-miR-759
FCRL1	hsa-miR-888-5p
FCRL1	hsa-miR-196a-5p
FCRL1	hsa-miR-196b-5p
FCRL1	hsa-miR-202-3p
FCRL1	hsa-miR-2114-5p
FCRL1	hsa-miR-31-5p
FCRL1	hsa-miR-3180-5p
FCRL1	hsa-miR-1208
FCRL1	hsa-miR-338-5p
FCRL1	hsa-miR-340-5p
FCRL1	hsa-miR-1231
FCRL1	hsa-miR-374a-5p
FCRL1	hsa-miR-374b-5p
FCRL1	hsa-miR-3919
FCRL2	hsa-miR-1253
FCRL2	hsa-miR-548c-3p
FCRL2	hsa-miR-15a-5p
FCRL2	hsa-miR-15b-5p
FCRL2	hsa-miR-16-5p
FCRL2	hsa-miR-195-5p
FCRL2	hsa-miR-3681-3p
FCRL5	hsa-miR-1253
FCRL5	hsa-miR-1270
FCRL5	hsa-miR-513a-5p
FCRL5	hsa-miR-185-5p
FCRL5	hsa-miR-339-5p
FCRL5	hsa-miR-421
FCRL5	hsa-miR-448
FCRL5	hsa-miR-125a-3p

FCRL5	hsa-miR-455-3p
FCRL5	hsa-miR-484
FCRL5	hsa-miR-485-5p
FCRL5	hsa-miR-486-3p
FCRL5	hsa-miR-488-3p
FCRL5	hsa-miR-497-5p
FCRL5	hsa-miR-509-3p
FCRL5	hsa-miR-510-5p
FCRL5	hsa-miR-513a-3p
FCRL5	hsa-miR-542-3p
FCRL5	hsa-miR-1290
FCRL5	hsa-miR-1291
FCRL5	hsa-miR-552-3p
FCRL5	hsa-miR-580-3p
FCRL5	hsa-miR-589-3p
FCRL5	hsa-miR-612
FCRL5	hsa-miR-620
FCRL5	hsa-miR-629-3p
FCRL5	hsa-miR-632
FCRL5	hsa-miR-635
FCRL5	hsa-miR-1305
FCRL5	hsa-miR-642a-5p
FCRL5	hsa-miR-651-5p
FCRL5	hsa-miR-671-5p
FCRL5	hsa-miR-134-5p
FCRL5	hsa-miR-138-5p
FCRL5	hsa-miR-758-3p
FCRL5	hsa-miR-765
FCRL5	hsa-miR-766-3p
FCRL5	hsa-miR-769-5p
FCRL5	hsa-miR-103a-3p
FCRL5	hsa-miR-148a-3p
FCRL5	hsa-miR-873-5p
FCRL5	hsa-miR-876-5p
FCRL5	hsa-miR-922
FCRL5	hsa-miR-936
FCRL5	hsa-miR-942-5p
FCRL5	hsa-miR-153-3p
FCRL5	hsa-miR-15a-5p
FCRL5	hsa-miR-15b-5p
FCRL5	hsa-miR-105-5p
FCRL5	hsa-miR-181b-5p
FCRL5	hsa-miR-181d-5p
FCRL5	hsa-miR-107
FCRL5	hsa-miR-195-5p
FCRL5	hsa-miR-204-5p
FCRL5	hsa-miR-211-5p
FCRL5	hsa-miR-214-3p

FCRL5	hsa-miR-1200
FCRL5	hsa-miR-3156-5p
FCRL5	hsa-miR-324-5p
FCRL5	hsa-miR-330-3p
FCRL5	hsa-miR-335-3p
FCRL5	hsa-miR-122-5p
FCRL5	hsa-miR-1231
FCRL5	hsa-miR-371a-3p
FCRL5	hsa-miR-375
FCRL5	hsa-miR-424-5p
FCRL5	hsa-miR-1248
FCRL5	hsa-miR-4308
FCRLA	hsa-miR-3167
FCRLA	hsa-miR-3170
FCRLA	hsa-miR-3652
FCRLA	hsa-miR-4317
FPR3	hsa-miR-433-3p
FPR3	hsa-miR-1254
FPR3	hsa-miR-1258
FPR3	hsa-miR-125a-5p
FPR3	hsa-miR-455-5p
FPR3	hsa-miR-125b-5p
FPR3	hsa-miR-1266-5p
FPR3	hsa-miR-501-5p
FPR3	hsa-miR-583
FPR3	hsa-miR-617
FPR3	hsa-miR-622
FPR3	hsa-miR-649
FPR3	hsa-miR-138-5p
FPR3	hsa-miR-758-3p
FPR3	hsa-miR-765
FPR3	hsa-miR-769-3p
FPR3	hsa-miR-938
FPR3	hsa-miR-942-5p
FPR3	hsa-miR-181b-5p
FPR3	hsa-miR-181c-5p
FPR3	hsa-miR-181d-5p
FPR3	hsa-miR-186-5p
FPR3	hsa-miR-193a-5p
FPR3	hsa-miR-198
FPR3	hsa-miR-1197
FPR3	hsa-miR-1233-3p
FYB	hsa-miR-450b-5p
FYB	hsa-miR-548a-3p
FYB	hsa-miR-548s
FYB	hsa-miR-578
FYB	hsa-miR-106a-3p
FYB	hsa-miR-302c-5p

FYB	hsa-miR-320a
FYB	hsa-miR-320b
FYB	hsa-miR-320c
FYB	hsa-miR-320d
FYB	hsa-miR-3671
FYB	hsa-miR-374a-5p
FYB	hsa-miR-3926
FYB	hsa-miR-411-5p
FYB	hsa-miR-4260
GBP5	hsa-miR-508-3p
GBP5	hsa-miR-548c-3p
GBP5	hsa-miR-578
GBP5	hsa-miR-599
GBP5	hsa-miR-139-5p
GBP5	hsa-miR-338-3p
GBP5	hsa-miR-340-5p
GBP5	hsa-miR-383-5p
GBP5	hsa-miR-410-3p
GPNMB	hsa-miR-185-5p
GPNMB	hsa-miR-338-5p
GPNMB	hsa-miR-4306
HLA-DOA	hsa-miR-1251-5p
HLA-DOA	hsa-miR-455-3p
HLA-DOA	hsa-miR-510-5p
HLA-DOA	hsa-miR-128-3p
HLA-DOA	hsa-miR-519d-3p
HLA-DOA	hsa-miR-520g-3p
HLA-DOA	hsa-miR-520h
HLA-DOA	hsa-miR-532-3p
HLA-DOA	hsa-miR-588
HLA-DOA	hsa-miR-1303
HLA-DOA	hsa-miR-645
HLA-DOA	hsa-miR-922
HLA-DOA	hsa-miR-93-5p
HLA-DOA	hsa-miR-17-5p
HLA-DOA	hsa-miR-188-3p
HLA-DOA	hsa-miR-188-5p
HLA-DOA	hsa-miR-203a
HLA-DOA	hsa-miR-20b-5p
HLA-DOA	hsa-miR-296-3p
HLA-DOA	hsa-miR-1197
HLA-DOA	hsa-miR-362-3p
HLA-DOA	hsa-miR-383-5p
HLA-DOA	hsa-miR-423-5p
HLA-DOA	hsa-miR-1254
HLA-DOA	hsa-miR-486-3p
HLA-DOA	hsa-miR-486-5p
HLA-DOA	hsa-miR-127-3p

HLA-DOA	hsa-miR-508-5p
HLA-DOA	hsa-miR-512-3p
HLA-DOA	hsa-miR-513a-5p
HLA-DOA	hsa-miR-513b-5p
HLA-DOA	hsa-miR-520b
HLA-DOA	hsa-miR-520c-3p
HLA-DOA	hsa-miR-520e
HLA-DOA	hsa-miR-520f-3p
HLA-DOA	hsa-miR-1285-3p
HLA-DOA	hsa-miR-1289
HLA-DOA	hsa-miR-548p
HLA-DOA	hsa-miR-554
HLA-DOA	hsa-miR-574-5p
HLA-DOA	hsa-miR-583
HLA-DOA	hsa-miR-589-5p
HLA-DOA	hsa-miR-591
HLA-DOA	hsa-miR-612
HLA-DOA	hsa-miR-616-3p
HLA-DOA	hsa-miR-635
HLA-DOA	hsa-miR-1305
HLA-DOA	hsa-miR-647
HLA-DOA	hsa-miR-650
HLA-DOA	hsa-miR-130a-3p
HLA-DOA	hsa-miR-130b-3p
HLA-DOA	hsa-miR-665
HLA-DOA	hsa-miR-130b-5p
HLA-DOA	hsa-miR-133a-3p
HLA-DOA	hsa-miR-133b
HLA-DOA	hsa-miR-135a-5p
HLA-DOA	hsa-miR-135b-5p
HLA-DOA	hsa-miR-140-3p
HLA-DOA	hsa-miR-143-3p
HLA-DOA	hsa-miR-708-5p
HLA-DOA	hsa-miR-759
HLA-DOA	hsa-miR-766-3p
HLA-DOA	hsa-miR-767-3p
HLA-DOA	hsa-miR-770-5p
HLA-DOA	hsa-miR-147a
HLA-DOA	hsa-miR-875-3p
HLA-DOA	hsa-miR-875-5p
HLA-DOA	hsa-miR-877-3p
HLA-DOA	hsa-miR-885-3p
HLA-DOA	hsa-miR-935
HLA-DOA	hsa-miR-943
HLA-DOA	hsa-miR-150-5p
HLA-DOA	hsa-miR-1827
HLA-DOA	hsa-miR-183-5p
HLA-DOA	hsa-miR-106a-5p

HLA-DOA	hsa-miR-185-3p
HLA-DOA	hsa-miR-106b-5p
HLA-DOA	hsa-miR-192-3p
HLA-DOA	hsa-miR-193a-3p
HLA-DOA	hsa-miR-19a-3p
HLA-DOA	hsa-miR-19b-3p
HLA-DOA	hsa-miR-1178-3p
HLA-DOA	hsa-miR-20a-5p
HLA-DOA	hsa-miR-22-5p
HLA-DOA	hsa-miR-2355-5p
HLA-DOA	hsa-miR-27a-3p
HLA-DOA	hsa-miR-27b-3p
HLA-DOA	hsa-miR-301a-3p
HLA-DOA	hsa-miR-301b
HLA-DOA	hsa-miR-3065-3p
HLA-DOA	hsa-miR-3119
HLA-DOA	hsa-miR-1205
HLA-DOA	hsa-miR-3160-3p
HLA-DOA	hsa-miR-3183
HLA-DOA	hsa-miR-3184-5p
HLA-DOA	hsa-miR-325
HLA-DOA	hsa-miR-329-3p
HLA-DOA	hsa-miR-330-5p
HLA-DOA	hsa-miR-1225-5p
HLA-DOA	hsa-miR-3614-3p
HLA-DOA	hsa-miR-1228-3p
HLA-DOA	hsa-miR-3667-3p
HLA-DOA	hsa-miR-3681-3p
HLA-DOA	hsa-miR-370-3p
HLA-DOA	hsa-miR-372-3p
HLA-DOA	hsa-miR-377-3p
HLA-DOA	hsa-miR-378a-5p
HLA-DOA	hsa-miR-3921
HLA-DOA	hsa-miR-3926
HLA-DOA	hsa-miR-4252
HLA-DOA	hsa-miR-4265
HLA-DOA	hsa-miR-4296
HLA-DOA	hsa-miR-4322
HRH2	hsa-miR-4257
ICOS	hsa-let-7a-2-3p
ICOS	hsa-let-7f-5p
ICOS	hsa-let-7g-3p
ICOS	hsa-miR-1283
ICOS	hsa-let-7g-5p
ICOS	hsa-let-7i-5p
ICOS	hsa-miR-576-3p
ICOS	hsa-miR-589-3p
ICOS	hsa-miR-609

ICOS	hsa-miR-647
ICOS	hsa-miR-767-3p
ICOS	hsa-miR-892b
ICOS	hsa-miR-922
ICOS	hsa-miR-149-5p
ICOS	hsa-let-7a-5p
ICOS	hsa-miR-193b-3p
ICOS	hsa-miR-1972
ICOS	hsa-miR-24-3p
ICOS	hsa-miR-297
ICOS	hsa-miR-29a-3p
ICOS	hsa-miR-29b-3p
ICOS	hsa-miR-29c-3p
ICOS	hsa-let-7b-5p
ICOS	hsa-miR-30a-3p
ICOS	hsa-miR-30e-3p
ICOS	hsa-miR-3136-5p
ICOS	hsa-miR-3139
ICOS	hsa-miR-3163
ICOS	hsa-miR-3170
ICOS	hsa-miR-3180-5p
ICOS	hsa-miR-323b-5p
ICOS	hsa-miR-338-3p
ICOS	hsa-miR-33a-3p
ICOS	hsa-miR-3605-5p
ICOS	hsa-let-7c-5p
ICOS	hsa-miR-379-5p
ICOS	hsa-miR-3915
ICOS	hsa-miR-1243
ICOS	hsa-miR-3927-3p
ICOS	hsa-miR-3929
ICOS	hsa-let-7d-5p
IKZF1	hsa-miR-448
IKZF1	hsa-miR-449b-5p
IKZF1	hsa-miR-450b-3p
IKZF1	hsa-miR-455-3p
IKZF1	hsa-miR-1261
IKZF1	hsa-miR-486-5p
IKZF1	hsa-miR-512-3p
IKZF1	hsa-miR-513a-3p
IKZF1	hsa-miR-513c-5p
IKZF1	hsa-miR-515-3p
IKZF1	hsa-miR-128-3p
IKZF1	hsa-miR-519e-3p
IKZF1	hsa-miR-520a-3p
IKZF1	hsa-miR-520b
IKZF1	hsa-miR-520c-3p
IKZF1	hsa-miR-520d-3p

IKZF1	hsa-miR-520f-3p
IKZF1	hsa-miR-539-5p
IKZF1	hsa-miR-542-3p
IKZF1	hsa-miR-1291
IKZF1	hsa-miR-579-3p
IKZF1	hsa-miR-583
IKZF1	hsa-miR-603
IKZF1	hsa-miR-612
IKZF1	hsa-miR-623
IKZF1	hsa-miR-628-5p
IKZF1	hsa-miR-1305
IKZF1	hsa-miR-130a-3p
IKZF1	hsa-miR-659-3p
IKZF1	hsa-miR-130b-3p
IKZF1	hsa-miR-1324
IKZF1	hsa-miR-137
IKZF1	hsa-miR-140-5p
IKZF1	hsa-miR-708-5p
IKZF1	hsa-miR-145-5p
IKZF1	hsa-miR-767-3p
IKZF1	hsa-miR-769-3p
IKZF1	hsa-miR-146b-3p
IKZF1	hsa-miR-148a-3p
IKZF1	hsa-miR-876-3p
IKZF1	hsa-miR-148b-3p
IKZF1	hsa-miR-890
IKZF1	hsa-miR-891b
IKZF1	hsa-miR-940
IKZF1	hsa-miR-943
IKZF1	hsa-miR-96-5p
IKZF1	hsa-miR-152-3p
IKZF1	hsa-miR-105-5p
IKZF1	hsa-miR-182-5p
IKZF1	hsa-miR-186-5p
IKZF1	hsa-miR-188-5p
IKZF1	hsa-miR-197-3p
IKZF1	hsa-miR-19a-3p
IKZF1	hsa-miR-19b-3p
IKZF1	hsa-miR-218-5p
IKZF1	hsa-miR-27a-3p
IKZF1	hsa-miR-27b-3p
IKZF1	hsa-miR-28-5p
IKZF1	hsa-miR-301a-3p
IKZF1	hsa-miR-301b
IKZF1	hsa-miR-302a-3p
IKZF1	hsa-miR-302b-3p
IKZF1	hsa-miR-302c-3p
IKZF1	hsa-miR-302d-3p

IKZF1	hsa-miR-320a
IKZF1	hsa-miR-320b
IKZF1	hsa-miR-320c
IKZF1	hsa-miR-320d
IKZF1	hsa-miR-323b-5p
IKZF1	hsa-miR-329-3p
IKZF1	hsa-miR-330-3p
IKZF1	hsa-miR-33a-5p
IKZF1	hsa-miR-33b-5p
IKZF1	hsa-miR-342-5p
IKZF1	hsa-miR-34b-5p
IKZF1	hsa-miR-34c-3p
IKZF1	hsa-miR-34c-5p
IKZF1	hsa-miR-1229-3p
IKZF1	hsa-miR-370-3p
IKZF1	hsa-miR-371a-5p
IKZF1	hsa-miR-373-3p
IKZF1	hsa-miR-1238-3p
IKZF1	hsa-miR-410-3p
IKZF1	hsa-miR-421
IKZF1	hsa-miR-4329
IKZF1	hsa-miR-1252-5p
IKZF1	hsa-miR-449a
IKZF1	hsa-miR-449c-5p
IKZF1	hsa-miR-450b-5p
IKZF1	hsa-miR-125a-3p
IKZF1	hsa-miR-125b-2-3p
IKZF1	hsa-miR-1266-5p
IKZF1	hsa-miR-1270
IKZF1	hsa-miR-1271-5p
IKZF1	hsa-miR-484
IKZF1	hsa-miR-486-3p
IKZF1	hsa-miR-487a-3p
IKZF1	hsa-miR-491-5p
IKZF1	hsa-miR-495-3p
IKZF1	hsa-miR-496
IKZF1	hsa-miR-497-3p
IKZF1	hsa-miR-1276
IKZF1	hsa-miR-509-3p
IKZF1	hsa-miR-1279
IKZF1	hsa-miR-514b-5p
IKZF1	hsa-miR-515-5p
IKZF1	hsa-miR-516b-5p
IKZF1	hsa-miR-518a-5p
IKZF1	hsa-miR-1283
IKZF1	hsa-miR-519a-3p
IKZF1	hsa-miR-519b-3p
IKZF1	hsa-miR-520e

IKZF1	hsa-miR-522-3p
IKZF1	hsa-miR-524-3p
IKZF1	hsa-miR-525-3p
IKZF1	hsa-miR-527
IKZF1	hsa-miR-548a-3p
IKZF1	hsa-miR-1287-5p
IKZF1	hsa-miR-548b-5p
IKZF1	hsa-miR-548c-3p
IKZF1	hsa-miR-548e-3p
IKZF1	hsa-miR-548j-5p
IKZF1	hsa-miR-548l
IKZF1	hsa-miR-552-3p
IKZF1	hsa-miR-559
IKZF1	hsa-miR-575
IKZF1	hsa-miR-582-5p
IKZF1	hsa-miR-588
IKZF1	hsa-miR-1299
IKZF1	hsa-miR-608
IKZF1	hsa-miR-1301-3p
IKZF1	hsa-miR-613
IKZF1	hsa-miR-1302
IKZF1	hsa-miR-622
IKZF1	hsa-miR-624-3p
IKZF1	hsa-miR-626
IKZF1	hsa-miR-629-3p
IKZF1	hsa-miR-632
IKZF1	hsa-miR-634
IKZF1	hsa-miR-636
IKZF1	hsa-miR-641
IKZF1	hsa-miR-642a-5p
IKZF1	hsa-miR-642b-3p
IKZF1	hsa-miR-651-5p
IKZF1	hsa-miR-654-3p
IKZF1	hsa-miR-661
IKZF1	hsa-miR-662
IKZF1	hsa-miR-665
IKZF1	hsa-miR-130b-5p
IKZF1	hsa-miR-1321
IKZF1	hsa-miR-1323
IKZF1	hsa-miR-133a-3p
IKZF1	hsa-miR-675-5p
IKZF1	hsa-miR-133b
IKZF1	hsa-miR-138-2-3p
IKZF1	hsa-miR-142-3p
IKZF1	hsa-miR-143-5p
IKZF1	hsa-miR-145-3p
IKZF1	hsa-miR-146b-5p
IKZF1	hsa-miR-875-3p

IKZF1	hsa-miR-876-5p
IKZF1	hsa-miR-888-5p
IKZF1	hsa-miR-892b
IKZF1	hsa-miR-921
IKZF1	hsa-miR-92a-3p
IKZF1	hsa-miR-92b-3p
IKZF1	hsa-miR-93-3p
IKZF1	hsa-miR-934
IKZF1	hsa-miR-149-5p
IKZF1	hsa-miR-942-5p
IKZF1	hsa-miR-9-5p
IKZF1	hsa-miR-150-5p
IKZF1	hsa-miR-153-3p
IKZF1	hsa-miR-17-3p
IKZF1	hsa-miR-1825
IKZF1	hsa-miR-1827
IKZF1	hsa-miR-185-3p
IKZF1	hsa-miR-187-5p
IKZF1	hsa-miR-190a-5p
IKZF1	hsa-miR-190b
IKZF1	hsa-miR-1910-5p
IKZF1	hsa-miR-1915-3p
IKZF1	hsa-miR-193b-3p
IKZF1	hsa-miR-195-3p
IKZF1	hsa-miR-1972
IKZF1	hsa-miR-199a-3p
IKZF1	hsa-miR-199a-5p
IKZF1	hsa-miR-199b-3p
IKZF1	hsa-miR-199b-5p
IKZF1	hsa-miR-10b-3p
IKZF1	hsa-miR-205-5p
IKZF1	hsa-miR-1178-3p
IKZF1	hsa-miR-20a-3p
IKZF1	hsa-miR-2110
IKZF1	hsa-miR-214-3p
IKZF1	hsa-miR-217
IKZF1	hsa-miR-224-3p
IKZF1	hsa-miR-24-3p
IKZF1	hsa-miR-26b-5p
IKZF1	hsa-miR-297
IKZF1	hsa-miR-299-5p
IKZF1	hsa-miR-29b-1-5p
IKZF1	hsa-miR-29b-2-5p
IKZF1	hsa-miR-302c-5p
IKZF1	hsa-miR-302e
IKZF1	hsa-miR-3065-5p
IKZF1	hsa-miR-1197
IKZF1	hsa-miR-3115

IKZF1	hsa-miR-3120-3p
IKZF1	hsa-miR-3122
IKZF1	hsa-miR-3125
IKZF1	hsa-miR-3132
IKZF1	hsa-miR-3138
IKZF1	hsa-miR-3148
IKZF1	hsa-miR-3154
IKZF1	hsa-miR-3158-3p
IKZF1	hsa-miR-31-5p
IKZF1	hsa-miR-3174
IKZF1	hsa-miR-3179
IKZF1	hsa-miR-3185
IKZF1	hsa-miR-3200-5p
IKZF1	hsa-miR-320e
IKZF1	hsa-miR-328-3p
IKZF1	hsa-miR-331-5p
IKZF1	hsa-miR-338-5p
IKZF1	hsa-miR-33b-3p
IKZF1	hsa-miR-340-5p
IKZF1	hsa-miR-342-3p
IKZF1	hsa-miR-345-5p
IKZF1	hsa-miR-34a-5p
IKZF1	hsa-miR-34b-3p
IKZF1	hsa-miR-1226-5p
IKZF1	hsa-miR-361-3p
IKZF1	hsa-miR-362-3p
IKZF1	hsa-miR-3653
IKZF1	hsa-miR-3666
IKZF1	hsa-miR-3667-3p
IKZF1	hsa-miR-3678-3p
IKZF1	hsa-miR-3681-3p
IKZF1	hsa-miR-3692-5p
IKZF1	hsa-miR-3714
IKZF1	hsa-miR-375
IKZF1	hsa-miR-376a-3p
IKZF1	hsa-miR-376b-3p
IKZF1	hsa-miR-1237-3p
IKZF1	hsa-miR-3911
IKZF1	hsa-miR-3913-5p
IKZF1	hsa-miR-3916
IKZF1	hsa-miR-409-3p
IKZF1	hsa-miR-4263
IKZF1	hsa-miR-4277
IKZF1	hsa-miR-4295
IKZF1	hsa-miR-4320
IKZF1	hsa-miR-432-5p
IL2RA	hsa-miR-502-5p
IL2RA	hsa-miR-635

IL2RA	hsa-miR-143-3p
IL2RA	hsa-miR-30a-5p
IL2RA	hsa-miR-30c-5p
IL2RA	hsa-miR-30d-5p
IL2RA	hsa-miR-30e-5p
IL2RA	hsa-miR-1237-3p
IL2RA	hsa-miR-1252-5p
IL2RA	hsa-miR-454-3p
IL2RA	hsa-miR-489-3p
IL2RA	hsa-miR-497-5p
IL2RA	hsa-miR-500a-3p
IL2RA	hsa-miR-507
IL2RA	hsa-miR-511-5p
IL2RA	hsa-miR-513c-5p
IL2RA	hsa-miR-548a-3p
IL2RA	hsa-miR-1288-3p
IL2RA	hsa-miR-574-5p
IL2RA	hsa-miR-578
IL2RA	hsa-miR-581
IL2RA	hsa-miR-595
IL2RA	hsa-miR-1299
IL2RA	hsa-miR-617
IL2RA	hsa-miR-624-3p
IL2RA	hsa-miR-629-3p
IL2RA	hsa-miR-629-5p
IL2RA	hsa-miR-1305
IL2RA	hsa-miR-647
IL2RA	hsa-miR-661
IL2RA	hsa-miR-134-5p
IL2RA	hsa-miR-145-5p
IL2RA	hsa-miR-103a-2-5p
IL2RA	hsa-miR-759
IL2RA	hsa-miR-147a
IL2RA	hsa-miR-877-5p
IL2RA	hsa-miR-888-5p
IL2RA	hsa-miR-892b
IL2RA	hsa-miR-922
IL2RA	hsa-miR-95-3p
IL2RA	hsa-miR-15a-5p
IL2RA	hsa-miR-15b-5p
IL2RA	hsa-miR-16-5p
IL2RA	hsa-miR-185-5p
IL2RA	hsa-miR-195-5p
IL2RA	hsa-miR-19a-5p
IL2RA	hsa-miR-2115-5p
IL2RA	hsa-miR-21-5p
IL2RA	hsa-miR-24-3p
IL2RA	hsa-miR-26a-5p

IL2RA	hsa-miR-29a-3p
IL2RA	hsa-miR-29b-3p
IL2RA	hsa-miR-30b-5p
IL2RA	hsa-miR-1200
IL2RA	hsa-miR-3182
IL2RA	hsa-miR-324-3p
IL2RA	hsa-miR-3663-5p
IL2RA	hsa-miR-3667-3p
IL2RA	hsa-miR-3692-5p
IL2RA	hsa-miR-370-3p
IL2RA	hsa-miR-371a-5p
IL2RA	hsa-miR-378a-5p
IL2RA	hsa-miR-409-3p
IL2RA	hsa-miR-424-5p
IL2RA	hsa-miR-1248
IL2RA	hsa-miR-431-5p
IRF4	hsa-miR-125a-5p
IRF4	hsa-miR-125b-5p
IRF4	hsa-miR-1276
IRF4	hsa-miR-513a-5p
IRF4	hsa-miR-513c-5p
IRF4	hsa-miR-128-3p
IRF4	hsa-miR-545-3p
IRF4	hsa-miR-548a-5p
IRF4	hsa-miR-548c-5p
IRF4	hsa-miR-548j-5p
IRF4	hsa-miR-1291
IRF4	hsa-miR-573
IRF4	hsa-miR-579-3p
IRF4	hsa-miR-587
IRF4	hsa-miR-597-5p
IRF4	hsa-miR-616-5p
IRF4	hsa-miR-651-5p
IRF4	hsa-miR-671-5p
IRF4	hsa-miR-146b-3p
IRF4	hsa-miR-103a-3p
IRF4	hsa-miR-185-5p
IRF4	hsa-miR-107
IRF4	hsa-miR-1178-3p
IRF4	hsa-miR-214-3p
IRF4	hsa-miR-27a-3p
IRF4	hsa-miR-27b-3p
IRF4	hsa-miR-30a-5p
IRF4	hsa-miR-30c-5p
IRF4	hsa-miR-30d-5p
IRF4	hsa-miR-30e-5p
IRF4	hsa-miR-1207-3p
IRF4	hsa-miR-328-3p

IRF4	hsa-miR-330-3p
IRF4	hsa-miR-373-3p
IRF4	hsa-miR-1245a
IRF4	hsa-miR-421
IRF4	hsa-miR-1254
IRF4	hsa-miR-449a
IRF4	hsa-miR-449b-5p
IRF4	hsa-miR-450b-3p
IRF4	hsa-miR-452-5p
IRF4	hsa-miR-1270
IRF4	hsa-let-7f-5p
IRF4	hsa-miR-486-3p
IRF4	hsa-miR-488-3p
IRF4	hsa-miR-491-3p
IRF4	hsa-miR-491-5p
IRF4	hsa-miR-496
IRF4	hsa-miR-497-5p
IRF4	hsa-miR-1275
IRF4	hsa-miR-501-3p
IRF4	hsa-miR-502-3p
IRF4	hsa-miR-509-3-5p
IRF4	hsa-miR-509-5p
IRF4	hsa-miR-510-5p
IRF4	hsa-miR-511-5p
IRF4	hsa-miR-512-3p
IRF4	hsa-miR-512-5p
IRF4	hsa-miR-516a-3p
IRF4	hsa-miR-516b-3p
IRF4	hsa-miR-1283
IRF4	hsa-let-7g-5p
IRF4	hsa-miR-520a-5p
IRF4	hsa-miR-520b
IRF4	hsa-miR-520c-3p
IRF4	hsa-miR-520d-3p
IRF4	hsa-miR-520e
IRF4	hsa-miR-524-5p
IRF4	hsa-miR-525-5p
IRF4	hsa-miR-539-5p
IRF4	hsa-miR-541-3p
IRF4	hsa-miR-545-5p
IRF4	hsa-miR-548b-5p
IRF4	hsa-miR-548d-5p
IRF4	hsa-miR-548e-3p
IRF4	hsa-miR-548f-3p
IRF4	hsa-miR-548h-5p
IRF4	hsa-miR-548i
IRF4	hsa-miR-548n
IRF4	hsa-miR-548p

IRF4	hsa-miR-548w
IRF4	hsa-miR-559
IRF4	hsa-miR-564
IRF4	hsa-let-7i-5p
IRF4	hsa-miR-129-5p
IRF4	hsa-miR-574-5p
IRF4	hsa-miR-575
IRF4	hsa-miR-576-3p
IRF4	hsa-miR-578
IRF4	hsa-miR-583
IRF4	hsa-miR-590-3p
IRF4	hsa-miR-592
IRF4	hsa-miR-1301-3p
IRF4	hsa-miR-617
IRF4	hsa-miR-1303
IRF4	hsa-miR-622
IRF4	hsa-miR-625-5p
IRF4	hsa-miR-641
IRF4	hsa-miR-1305
IRF4	hsa-miR-648
IRF4	hsa-miR-653-5p
IRF4	hsa-miR-654-5p
IRF4	hsa-miR-658
IRF4	hsa-miR-659-3p
IRF4	hsa-miR-665
IRF4	hsa-miR-670-5p
IRF4	hsa-miR-1321
IRF4	hsa-miR-134-5p
IRF4	hsa-miR-141-5p
IRF4	hsa-miR-744-5p
IRF4	hsa-miR-7-5p
IRF4	hsa-miR-765
IRF4	hsa-miR-766-3p
IRF4	hsa-miR-767-5p
IRF4	hsa-miR-769-3p
IRF4	hsa-miR-147a
IRF4	hsa-miR-873-5p
IRF4	hsa-miR-889-3p
IRF4	hsa-miR-890
IRF4	hsa-miR-892a
IRF4	hsa-miR-922
IRF4	hsa-miR-93-3p
IRF4	hsa-miR-942-5p
IRF4	hsa-miR-9-5p
IRF4	hsa-miR-98-5p
IRF4	hsa-miR-155-3p
IRF4	hsa-miR-15a-5p
IRF4	hsa-miR-15b-5p

IRF4	hsa-miR-16-5p
IRF4	hsa-miR-17-3p
IRF4	hsa-miR-1825
IRF4	hsa-miR-183-5p
IRF4	hsa-let-7a-5p
IRF4	hsa-miR-186-5p
IRF4	hsa-miR-195-5p
IRF4	hsa-miR-200b-3p
IRF4	hsa-miR-200c-3p
IRF4	hsa-miR-203a
IRF4	hsa-miR-204-5p
IRF4	hsa-miR-205-5p
IRF4	hsa-miR-20b-3p
IRF4	hsa-miR-2115-5p
IRF4	hsa-miR-211-5p
IRF4	hsa-miR-216a-5p
IRF4	hsa-miR-217
IRF4	hsa-miR-219a-2-3p
IRF4	hsa-miR-221-3p
IRF4	hsa-miR-222-3p
IRF4	hsa-miR-224-5p
IRF4	hsa-miR-1183
IRF4	hsa-miR-26a-5p
IRF4	hsa-miR-26b-5p
IRF4	hsa-miR-1184
IRF4	hsa-miR-296-3p
IRF4	hsa-miR-296-5p
IRF4	hsa-miR-298
IRF4	hsa-miR-302a-3p
IRF4	hsa-miR-302d-3p
IRF4	hsa-miR-302e
IRF4	hsa-miR-3065-5p
IRF4	hsa-let-7b-5p
IRF4	hsa-miR-30a-3p
IRF4	hsa-miR-30b-5p
IRF4	hsa-miR-30d-3p
IRF4	hsa-miR-30e-3p
IRF4	hsa-miR-1200
IRF4	hsa-miR-3141
IRF4	hsa-miR-3148
IRF4	hsa-miR-3171
IRF4	hsa-miR-3173-3p
IRF4	hsa-miR-3180-5p
IRF4	hsa-miR-3185
IRF4	hsa-miR-1208
IRF4	hsa-miR-320a
IRF4	hsa-miR-320b
IRF4	hsa-miR-320c

IRF4	hsa-miR-320d
IRF4	hsa-miR-326
IRF4	hsa-miR-339-5p
IRF4	hsa-miR-1225-5p
IRF4	hsa-miR-34a-5p
IRF4	hsa-miR-34c-5p
IRF4	hsa-miR-3619-5p
IRF4	hsa-miR-3665
IRF4	hsa-miR-1229-3p
IRF4	hsa-miR-3678-3p
IRF4	hsa-miR-3680-3p
IRF4	hsa-miR-3681-3p
IRF4	hsa-miR-3685
IRF4	hsa-miR-372-3p
IRF4	hsa-miR-373-5p
IRF4	hsa-miR-384
IRF4	hsa-miR-3908
IRF4	hsa-miR-3915
IRF4	hsa-miR-1243
IRF4	hsa-let-7d-5p
IRF4	hsa-miR-424-5p
IRF4	hsa-miR-4251
IRF4	hsa-miR-4268
IRF4	hsa-miR-429
IRF4	hsa-miR-1248
IRF4	hsa-miR-4306
IRF4	hsa-miR-4319
IRF4	hsa-miR-432-5p
KCNA3	hsa-miR-4330
KCNA3	hsa-miR-433-3p
KCNA3	hsa-miR-449c-5p
KCNA3	hsa-miR-452-5p
KCNA3	hsa-miR-493-3p
KCNA3	hsa-let-7g-3p
KCNA3	hsa-miR-516b-5p
KCNA3	hsa-miR-541-5p
KCNA3	hsa-miR-548d-3p
KCNA3	hsa-miR-548z
KCNA3	hsa-miR-551b-5p
KCNA3	hsa-miR-561-3p
KCNA3	hsa-miR-578
KCNA3	hsa-miR-590-5p
KCNA3	hsa-miR-1298-5p
KCNA3	hsa-miR-1299
KCNA3	hsa-miR-629-5p
KCNA3	hsa-miR-643
KCNA3	hsa-miR-648
KCNA3	hsa-miR-140-5p

KCNA3	hsa-miR-802
KCNA3	hsa-miR-148a-5p
KCNA3	hsa-miR-105-5p
KCNA3	hsa-miR-185-3p
KCNA3	hsa-miR-20b-3p
KCNA3	hsa-miR-216b-5p
KCNA3	hsa-miR-217
KCNA3	hsa-miR-297
KCNA3	hsa-miR-30b-3p
KCNA3	hsa-miR-30d-3p
KCNA3	hsa-miR-3151-5p
KCNA3	hsa-miR-3158-3p
KCNA3	hsa-miR-3160-3p
KCNA3	hsa-miR-1207-5p
KCNA3	hsa-miR-3192-5p
KCNA3	hsa-miR-3202
KCNA3	hsa-miR-324-3p
KCNA3	hsa-miR-335-3p
KCNA3	hsa-miR-34b-5p
KCNA3	hsa-miR-3658
KCNA3	hsa-miR-1229-3p
KCNA3	hsa-miR-3682-3p
KCNA3	hsa-miR-3689a-3p
KCNA3	hsa-miR-3689b-3p
KCNA3	hsa-miR-382-5p
KCNA3	hsa-miR-383-5p
KCNA3	hsa-miR-3914
KCNA3	hsa-miR-3925-5p
KCNA3	hsa-miR-3936
KCNA3	hsa-miR-3944-3p
KCNA3	hsa-miR-4298
KCNA3	hsa-miR-432-5p
KCNJ15	hsa-miR-1273e
KCNJ15	hsa-miR-513c-5p
KCNJ15	hsa-miR-1
KCNJ15	hsa-miR-636
KCNJ15	hsa-miR-643
KCNJ15	hsa-miR-711
KCNJ15	hsa-miR-203a
KCNJ15	hsa-miR-206
KCNJ15	hsa-miR-3149
KCNJ15	hsa-miR-3646
KCNJ15	hsa-miR-4328
KLHL6	hsa-let-7e-5p
KLHL6	hsa-miR-450b-5p
KLHL6	hsa-miR-125a-5p
KLHL6	hsa-miR-452-5p
KLHL6	hsa-miR-125b-5p

KLHL6	hsa-let-7f-5p
KLHL6	hsa-miR-488-3p
KLHL6	hsa-miR-499a-5p
KLHL6	hsa-miR-502-5p
KLHL6	hsa-miR-513b-5p
KLHL6	hsa-miR-513c-5p
KLHL6	hsa-let-7g-5p
KLHL6	hsa-miR-1284
KLHL6	hsa-miR-520f-3p
KLHL6	hsa-miR-542-3p
KLHL6	hsa-miR-543
KLHL6	hsa-miR-548a-3p
KLHL6	hsa-miR-1290
KLHL6	hsa-miR-1291
KLHL6	hsa-miR-550a-5p
KLHL6	hsa-let-7i-5p
KLHL6	hsa-miR-573
KLHL6	hsa-miR-575
KLHL6	hsa-miR-582-3p
KLHL6	hsa-miR-593-3p
KLHL6	hsa-miR-617
KLHL6	hsa-miR-618
KLHL6	hsa-miR-619-3p
KLHL6	hsa-miR-622
KLHL6	hsa-miR-629-3p
KLHL6	hsa-miR-631
KLHL6	hsa-miR-649
KLHL6	hsa-miR-661
KLHL6	hsa-miR-1323
KLHL6	hsa-miR-142-5p
KLHL6	hsa-miR-143-3p
KLHL6	hsa-miR-766-3p
KLHL6	hsa-miR-146b-3p
KLHL6	hsa-miR-876-5p
KLHL6	hsa-miR-98-5p
KLHL6	hsa-miR-182-5p
KLHL6	hsa-let-7a-5p
KLHL6	hsa-miR-107
KLHL6	hsa-miR-199a-3p
KLHL6	hsa-miR-199b-3p
KLHL6	hsa-miR-216a-5p
KLHL6	hsa-miR-221-3p
KLHL6	hsa-miR-222-3p
KLHL6	hsa-let-7b-5p
KLHL6	hsa-miR-1200
KLHL6	hsa-miR-1208
KLHL6	hsa-miR-326
KLHL6	hsa-miR-330-5p

KLHL6	hsa-let-7c-5p
KLHL6	hsa-miR-362-5p
KLHL6	hsa-miR-1231
KLHL6	hsa-miR-370-3p
KLHL6	hsa-miR-373-3p
KLHL6	hsa-miR-378a-5p
KLHL6	hsa-miR-1238-3p
KLHL6	hsa-miR-124-3p
KLHL6	hsa-miR-1244
KLHL6	hsa-miR-1248
KLHL6	hsa-miR-1254
KLHL6	hsa-miR-448
KLHL6	hsa-miR-125b-2-3p
KLHL6	hsa-miR-1264
KLHL6	hsa-let-7f-2-3p
KLHL6	hsa-miR-1270
KLHL6	hsa-miR-1276
KLHL6	hsa-miR-500a-5p
KLHL6	hsa-miR-500b-5p
KLHL6	hsa-miR-506-3p
KLHL6	hsa-miR-507
KLHL6	hsa-miR-508-5p
KLHL6	hsa-miR-510-5p
KLHL6	hsa-miR-512-3p
KLHL6	hsa-miR-512-5p
KLHL6	hsa-miR-513a-5p
KLHL6	hsa-miR-1281
KLHL6	hsa-miR-515-3p
KLHL6	hsa-miR-515-5p
KLHL6	hsa-miR-516a-3p
KLHL6	hsa-miR-516b-3p
KLHL6	hsa-miR-518d-5p
KLHL6	hsa-miR-519b-5p
KLHL6	hsa-miR-519c-5p
KLHL6	hsa-miR-519e-3p
KLHL6	hsa-miR-520b
KLHL6	hsa-miR-520c-3p
KLHL6	hsa-miR-520c-5p
KLHL6	hsa-miR-526a
KLHL6	hsa-miR-541-5p
KLHL6	hsa-miR-548c-3p
KLHL6	hsa-miR-1289
KLHL6	hsa-miR-548e-3p
KLHL6	hsa-miR-548f-3p
KLHL6	hsa-miR-548g-3p
KLHL6	hsa-miR-548o-3p
KLHL6	hsa-miR-548q
KLHL6	hsa-miR-548s

KLHL6	hsa-miR-548v
KLHL6	hsa-miR-563
KLHL6	hsa-miR-129-5p
KLHL6	hsa-miR-574-5p
KLHL6	hsa-miR-576-5p
KLHL6	hsa-miR-578
KLHL6	hsa-miR-579-3p
KLHL6	hsa-miR-583
KLHL6	hsa-miR-588
KLHL6	hsa-miR-589-3p
KLHL6	hsa-miR-590-3p
KLHL6	hsa-miR-596
KLHL6	hsa-miR-600
KLHL6	hsa-miR-1299
KLHL6	hsa-miR-616-3p
KLHL6	hsa-miR-627-5p
KLHL6	hsa-miR-629-5p
KLHL6	hsa-miR-630
KLHL6	hsa-miR-635
KLHL6	hsa-miR-1305
KLHL6	hsa-miR-644a
KLHL6	hsa-miR-646
KLHL6	hsa-miR-659-3p
KLHL6	hsa-miR-663a
KLHL6	hsa-miR-664a-3p
KLHL6	hsa-miR-670-5p
KLHL6	hsa-miR-132-3p
KLHL6	hsa-miR-134-5p
KLHL6	hsa-miR-101-3p
KLHL6	hsa-miR-138-2-3p
KLHL6	hsa-miR-138-5p
KLHL6	hsa-miR-139-5p
KLHL6	hsa-miR-103a-2-5p
KLHL6	hsa-miR-7-5p
KLHL6	hsa-miR-765
KLHL6	hsa-miR-767-3p
KLHL6	hsa-miR-769-5p
KLHL6	hsa-miR-103a-3p
KLHL6	hsa-miR-890
KLHL6	hsa-miR-892a
KLHL6	hsa-miR-922
KLHL6	hsa-miR-93-3p
KLHL6	hsa-miR-935
KLHL6	hsa-miR-150-5p
KLHL6	hsa-miR-155-3p
KLHL6	hsa-miR-1825
KLHL6	hsa-miR-1827
KLHL6	hsa-miR-186-3p

KLHL6	hsa-miR-192-5p
KLHL6	hsa-miR-193a-5p
KLHL6	hsa-miR-1976
KLHL6	hsa-miR-19a-5p
KLHL6	hsa-miR-19b-1-5p
KLHL6	hsa-miR-19b-2-5p
KLHL6	hsa-miR-203a
KLHL6	hsa-miR-208a-3p
KLHL6	hsa-miR-212-3p
KLHL6	hsa-miR-219a-2-3p
KLHL6	hsa-miR-221-5p
KLHL6	hsa-miR-224-3p
KLHL6	hsa-miR-26a-5p
KLHL6	hsa-miR-26b-5p
KLHL6	hsa-miR-27b-5p
KLHL6	hsa-miR-296-3p
KLHL6	hsa-miR-297
KLHL6	hsa-miR-299-5p
KLHL6	hsa-miR-29b-1-5p
KLHL6	hsa-miR-1197
KLHL6	hsa-miR-3126-3p
KLHL6	hsa-miR-3128
KLHL6	hsa-miR-3129-5p
KLHL6	hsa-miR-3144-3p
KLHL6	hsa-miR-3160-3p
KLHL6	hsa-miR-3164
KLHL6	hsa-miR-3167
KLHL6	hsa-miR-1207-5p
KLHL6	hsa-miR-3200-5p
KLHL6	hsa-miR-320a
KLHL6	hsa-miR-338-3p
KLHL6	hsa-miR-338-5p
KLHL6	hsa-miR-339-5p
KLHL6	hsa-miR-342-3p
KLHL6	hsa-miR-342-5p
KLHL6	hsa-miR-34b-5p
KLHL6	hsa-miR-1226-3p
KLHL6	hsa-miR-361-3p
KLHL6	hsa-miR-3646
KLHL6	hsa-miR-3653
KLHL6	hsa-miR-3659
KLHL6	hsa-miR-365a-5p
KLHL6	hsa-miR-3661
KLHL6	hsa-miR-3667-3p
KLHL6	hsa-miR-3670
KLHL6	hsa-miR-3680-3p
KLHL6	hsa-miR-3689a-5p
KLHL6	hsa-miR-3689b-5p

KLHL6	hsa-miR-371a-5p
KLHL6	hsa-miR-372-3p
KLHL6	hsa-miR-375
KLHL6	hsa-miR-379-3p
KLHL6	hsa-miR-380-3p
KLHL6	hsa-miR-380-5p
KLHL6	hsa-miR-3921
KLHL6	hsa-let-7d-5p
KLHL6	hsa-miR-1245a
KLHL6	hsa-miR-410-3p
KLHL6	hsa-miR-411-3p
KLHL6	hsa-miR-4256
KLHL6	hsa-miR-1246
KLHL6	hsa-miR-4302
KLHL6	hsa-miR-4311
LAX1	hsa-miR-1261
LAX1	hsa-miR-543
LAX1	hsa-miR-545-3p
LAX1	hsa-miR-450b-5p
LAX1	hsa-miR-1267
LAX1	hsa-miR-127-5p
LAX1	hsa-miR-512-3p
LAX1	hsa-miR-520b
LAX1	hsa-miR-520c-3p
LAX1	hsa-miR-520d-3p
LAX1	hsa-miR-520e
LAX1	hsa-miR-520g-3p
LAX1	hsa-miR-1290
LAX1	hsa-miR-548p
LAX1	hsa-miR-578
LAX1	hsa-miR-581
LAX1	hsa-miR-601
LAX1	hsa-miR-1299
LAX1	hsa-miR-1301-3p
LAX1	hsa-miR-628-3p
LAX1	hsa-miR-1305
LAX1	hsa-miR-644a
LAX1	hsa-miR-132-3p
LAX1	hsa-miR-103a-2-5p
LAX1	hsa-miR-767-3p
LAX1	hsa-miR-924
LAX1	hsa-miR-93-5p
LAX1	hsa-miR-17-5p
LAX1	hsa-miR-106b-5p
LAX1	hsa-miR-19a-5p
LAX1	hsa-miR-19b-1-5p
LAX1	hsa-miR-19b-2-5p
LAX1	hsa-miR-203a

LAX1	hsa-miR-204-5p
LAX1	hsa-miR-20b-5p
LAX1	hsa-miR-211-5p
LAX1	hsa-miR-212-3p
LAX1	hsa-miR-22-3p
LAX1	hsa-miR-27b-3p
LAX1	hsa-miR-1200
LAX1	hsa-miR-325
LAX1	hsa-miR-378a-5p
LAX1	hsa-miR-3938
LAX1	hsa-miR-421
LILRB4	hsa-miR-574-5p
LILRB4	hsa-miR-3170
LY9	hsa-miR-581
LY9	hsa-miR-592
LY9	hsa-miR-141-3p
LY9	hsa-miR-151a-5p
LY9	hsa-miR-1827
LY9	hsa-miR-200a-3p
MEFV	hsa-miR-502-5p
MEFV	hsa-miR-622
MEFV	hsa-miR-646
MEFV	hsa-miR-766-3p
MEFV	hsa-miR-767-3p
MEFV	hsa-miR-940
MEFV	hsa-miR-1827
MEFV	hsa-miR-362-5p
MEFV	hsa-miR-433-3p
MEFV	hsa-miR-1257
MEFV	hsa-miR-1262
MEFV	hsa-miR-1272
MEFV	hsa-miR-485-5p
MEFV	hsa-miR-491-3p
MEFV	hsa-miR-500b-5p
MEFV	hsa-miR-503-5p
MEFV	hsa-miR-508-5p
MEFV	hsa-miR-128-3p
MEFV	hsa-miR-566
MEFV	hsa-miR-1295a
MEFV	hsa-miR-129-5p
MEFV	hsa-miR-574-5p
MEFV	hsa-miR-576-5p
MEFV	hsa-miR-592
MEFV	hsa-miR-621
MEFV	hsa-miR-645
MEFV	hsa-miR-658
MEFV	hsa-miR-661
MEFV	hsa-miR-664a-3p

MEFV	hsa-miR-664a-5p
MEFV	hsa-miR-146a-3p
MEFV	hsa-miR-891a-5p
MEFV	hsa-miR-92a-3p
MEFV	hsa-miR-92b-3p
MEFV	hsa-miR-15a-5p
MEFV	hsa-miR-15b-5p
MEFV	hsa-miR-16-5p
MEFV	hsa-miR-186-5p
MEFV	hsa-miR-300
MEFV	hsa-miR-30b-3p
MEFV	hsa-miR-3147
MEFV	hsa-miR-3190-5p
MEFV	hsa-miR-335-5p
MEFV	hsa-miR-1226-5p
MEFV	hsa-miR-1227-3p
MEFV	hsa-miR-363-3p
MEFV	hsa-miR-3656
MEFV	hsa-miR-367-3p
MEFV	hsa-miR-377-5p
MEFV	hsa-miR-3925-5p
MEFV	hsa-miR-412-3p
MEFV	hsa-miR-4253
MPEG1	hsa-miR-1254
MPEG1	hsa-miR-1287-5p
MPEG1	hsa-miR-574-5p
MPEG1	hsa-miR-581
MPEG1	hsa-miR-586
MPEG1	hsa-miR-603
MPEG1	hsa-miR-612
MPEG1	hsa-miR-875-3p
MPEG1	hsa-miR-940
MPEG1	hsa-miR-942-5p
MPEG1	hsa-miR-155-5p
MPEG1	hsa-miR-211-5p
MPEG1	hsa-miR-34b-5p
MPEG1	hsa-miR-449c-5p
MPEG1	hsa-miR-1258
MPEG1	hsa-miR-125a-3p
MPEG1	hsa-miR-1270
MPEG1	hsa-miR-1271-5p
MPEG1	hsa-miR-486-3p
MPEG1	hsa-miR-508-3p
MPEG1	hsa-miR-509-3p
MPEG1	hsa-miR-511-5p
MPEG1	hsa-miR-515-5p
MPEG1	hsa-miR-516a-3p
MPEG1	hsa-miR-516b-3p

MPEG1	hsa-miR-1285-3p
MPEG1	hsa-miR-548c-3p
MPEG1	hsa-miR-551b-5p
MPEG1	hsa-miR-579-3p
MPEG1	hsa-miR-592
MPEG1	hsa-miR-593-3p
MPEG1	hsa-miR-622
MPEG1	hsa-miR-651-5p
MPEG1	hsa-miR-132-3p
MPEG1	hsa-miR-140-5p
MPEG1	hsa-miR-888-5p
MPEG1	hsa-miR-890
MPEG1	hsa-miR-105-5p
MPEG1	hsa-miR-185-5p
MPEG1	hsa-miR-186-5p
MPEG1	hsa-miR-188-5p
MPEG1	hsa-miR-1915-3p
MPEG1	hsa-miR-194-5p
MPEG1	hsa-miR-203a
MPEG1	hsa-miR-204-5p
MPEG1	hsa-miR-206
MPEG1	hsa-miR-212-3p
MPEG1	hsa-miR-296-3p
MPEG1	hsa-miR-3148
MPEG1	hsa-miR-1224-5p
MPEG1	hsa-miR-122-5p
MPEG1	hsa-miR-361-3p
MPEG1	hsa-miR-3680-3p
MPEG1	hsa-miR-371a-5p
MPEG1	hsa-miR-1236-3p
MPEG1	hsa-miR-381-3p
MPEG1	hsa-miR-1244
MPEG1	hsa-miR-425-5p
MPEG1	hsa-miR-4257
MPEG1	hsa-miR-4325
MRC1	hsa-miR-130a-5p
MRC1	hsa-miR-196a-5p
MRC1	hsa-miR-196b-5p
MRC1	hsa-miR-1185-5p
MRC1	hsa-miR-3679-5p
MRC1	hsa-miR-3684
MS4A1	hsa-miR-499a-3p
MS4A1	hsa-miR-516b-5p
MS4A1	hsa-miR-576-3p
MS4A1	hsa-miR-578
MS4A1	hsa-miR-589-5p
MS4A1	hsa-miR-613
MS4A1	hsa-miR-1

MS4A1	hsa-miR-643
MS4A1	hsa-miR-130a-5p
MS4A1	hsa-miR-143-5p
MS4A1	hsa-miR-146a-5p
MS4A1	hsa-miR-146b-5p
MS4A1	hsa-miR-149-5p
MS4A1	hsa-miR-206
MS4A1	hsa-miR-215-5p
MS4A1	hsa-miR-23a-3p
MS4A1	hsa-miR-28-5p
MS4A1	hsa-miR-30a-3p
MS4A1	hsa-miR-30e-3p
MS4A1	hsa-miR-3126-3p
MS4A1	hsa-miR-3166
MS4A1	hsa-miR-3661
MSR1	hsa-miR-526b-3p
MSR1	hsa-miR-33b-5p
MSR1	hsa-miR-34b-5p
MSR1	hsa-miR-3680-3p
NUGGC	hsa-miR-450b-5p
NUGGC	hsa-miR-1285-3p
NUGGC	hsa-miR-548b-5p
NUGGC	hsa-miR-548j-5p
NUGGC	hsa-miR-136-5p
NUGGC	hsa-miR-330-5p
NUGGC	hsa-miR-450a-5p
NUGGC	hsa-miR-1262
NUGGC	hsa-miR-507
NUGGC	hsa-miR-512-3p
NUGGC	hsa-miR-516b-5p
NUGGC	hsa-miR-520a-3p
NUGGC	hsa-miR-520b
NUGGC	hsa-miR-520c-3p
NUGGC	hsa-miR-520d-3p
NUGGC	hsa-miR-520e
NUGGC	hsa-miR-520g-3p
NUGGC	hsa-miR-548a-5p
NUGGC	hsa-miR-548c-5p
NUGGC	hsa-miR-548d-5p
NUGGC	hsa-miR-548h-5p
NUGGC	hsa-miR-548i
NUGGC	hsa-miR-548n
NUGGC	hsa-miR-562
NUGGC	hsa-miR-564
NUGGC	hsa-miR-587
NUGGC	hsa-miR-135b-5p
NUGGC	hsa-miR-760
NUGGC	hsa-miR-766-3p

NUGGC	hsa-miR-892a
NUGGC	hsa-miR-92a-3p
NUGGC	hsa-miR-92b-3p
NUGGC	hsa-miR-93-5p
NUGGC	hsa-miR-17-5p
NUGGC	hsa-miR-106a-5p
NUGGC	hsa-miR-106b-5p
NUGGC	hsa-miR-20b-5p
NUGGC	hsa-miR-24-3p
NUGGC	hsa-miR-372-3p
NUGGC	hsa-let-7d-5p
P2RY13	hsa-miR-1256
P2RY13	hsa-miR-1261
P2RY13	hsa-miR-501-5p
P2RY13	hsa-miR-589-5p
P2RY13	hsa-miR-625-5p
P2RY13	hsa-miR-92a-2-5p
P2RY13	hsa-miR-936
P2RY13	hsa-miR-199a-3p
P2RY13	hsa-miR-199b-3p
P2RY13	hsa-miR-205-3p
P2RY13	hsa-miR-216b-5p
P2RY13	hsa-miR-219a-2-3p
P2RY13	hsa-miR-3126-3p
P2RY13	hsa-miR-3129-5p
P2RY13	hsa-miR-3151-5p
PDCD1LG2	hsa-miR-1252-5p
PDCD1LG2	hsa-miR-512-3p
PDCD1LG2	hsa-miR-519a-3p
PDCD1LG2	hsa-miR-519b-3p
PDCD1LG2	hsa-miR-519c-3p
PDCD1LG2	hsa-miR-520a-3p
PDCD1LG2	hsa-miR-520a-5p
PDCD1LG2	hsa-miR-520b
PDCD1LG2	hsa-miR-520c-3p
PDCD1LG2	hsa-miR-520d-3p
PDCD1LG2	hsa-miR-520e
PDCD1LG2	hsa-miR-520f-3p
PDCD1LG2	hsa-miR-525-5p
PDCD1LG2	hsa-miR-526b-3p
PDCD1LG2	hsa-miR-636
PDCD1LG2	hsa-miR-758-3p
PDCD1LG2	hsa-miR-93-5p
PDCD1LG2	hsa-miR-17-5p
PDCD1LG2	hsa-miR-106a-5p
PDCD1LG2	hsa-miR-106b-5p
PDCD1LG2	hsa-miR-198
PDCD1LG2	hsa-miR-19a-3p

PDCD1LG2	hsa-miR-19b-3p
PDCD1LG2	hsa-miR-20a-5p
PDCD1LG2	hsa-miR-2113
PDCD1LG2	hsa-miR-3171
PDCD1LG2	hsa-miR-3173-3p
PDCD1LG2	hsa-miR-372-3p
PDCD1LG2	hsa-miR-4257
PIK3CG	hsa-miR-1256
PIK3CG	hsa-miR-1276
PIK3CG	hsa-miR-506-3p
PIK3CG	hsa-miR-507
PIK3CG	hsa-miR-552-3p
PIK3CG	hsa-miR-557
PIK3CG	hsa-miR-561-3p
PIK3CG	hsa-miR-1305
PIK3CG	hsa-miR-650
PIK3CG	hsa-miR-671-5p
PIK3CG	hsa-miR-1321
PIK3CG	hsa-miR-142-3p
PIK3CG	hsa-miR-143-3p
PIK3CG	hsa-miR-892a
PIK3CG	hsa-miR-940
PIK3CG	hsa-miR-186-5p
PIK3CG	hsa-miR-219a-1-3p
PIK3CG	hsa-miR-1207-5p
PIK3CG	hsa-miR-1208
PIK3CG	hsa-miR-124-3p
PIK3CG	hsa-miR-1255a
PIK3CG	hsa-miR-1261
PIK3CG	hsa-miR-1265
PIK3CG	hsa-miR-1267
PIK3CG	hsa-miR-487a-3p
PIK3CG	hsa-miR-490-3p
PIK3CG	hsa-miR-493-3p
PIK3CG	hsa-miR-498
PIK3CG	hsa-miR-500a-5p
PIK3CG	hsa-miR-502-5p
PIK3CG	hsa-miR-1279
PIK3CG	hsa-miR-513b-5p
PIK3CG	hsa-miR-514a-3p
PIK3CG	hsa-miR-515-5p
PIK3CG	hsa-miR-519e-5p
PIK3CG	hsa-miR-520g-3p
PIK3CG	hsa-miR-520h
PIK3CG	hsa-miR-548a-3p
PIK3CG	hsa-miR-548a-5p
PIK3CG	hsa-miR-1288-3p
PIK3CG	hsa-miR-548b-5p

PIK3CG	hsa-miR-548c-5p
PIK3CG	hsa-miR-548d-5p
PIK3CG	hsa-miR-548e-3p
PIK3CG	hsa-miR-548h-5p
PIK3CG	hsa-miR-548i
PIK3CG	hsa-miR-548j-5p
PIK3CG	hsa-miR-548k
PIK3CG	hsa-miR-548m
PIK3CG	hsa-miR-548w
PIK3CG	hsa-miR-571
PIK3CG	hsa-miR-581
PIK3CG	hsa-miR-583
PIK3CG	hsa-miR-592
PIK3CG	hsa-miR-597-5p
PIK3CG	hsa-miR-1299
PIK3CG	hsa-miR-634
PIK3CG	hsa-miR-139-5p
PIK3CG	hsa-miR-708-3p
PIK3CG	hsa-miR-146b-3p
PIK3CG	hsa-miR-875-3p
PIK3CG	hsa-miR-885-5p
PIK3CG	hsa-miR-890
PIK3CG	hsa-miR-92a-3p
PIK3CG	hsa-miR-944
PIK3CG	hsa-miR-105-5p
PIK3CG	hsa-miR-1972
PIK3CG	hsa-miR-202-3p
PIK3CG	hsa-miR-205-3p
PIK3CG	hsa-miR-219a-2-3p
PIK3CG	hsa-miR-224-5p
PIK3CG	hsa-miR-22-5p
PIK3CG	hsa-miR-1182
PIK3CG	hsa-miR-24-3p
PIK3CG	hsa-miR-1184
PIK3CG	hsa-miR-28-3p
PIK3CG	hsa-miR-3121-3p
PIK3CG	hsa-miR-1200
PIK3CG	hsa-miR-1202
PIK3CG	hsa-miR-3145-3p
PIK3CG	hsa-miR-3154
PIK3CG	hsa-miR-32-5p
PIK3CG	hsa-miR-335-3p
PIK3CG	hsa-miR-122-5p
PIK3CG	hsa-miR-34a-3p
PIK3CG	hsa-miR-1227-3p
PIK3CG	hsa-miR-3622b-5p
PIK3CG	hsa-miR-362-5p
PIK3CG	hsa-miR-363-3p

PIK3CG	hsa-miR-3657
PIK3CG	hsa-miR-378a-3p
PIK3CG	hsa-miR-380-3p
PIK3CG	hsa-miR-3920
PIK3CG	hsa-miR-3924
PIK3CG	hsa-miR-3926
PIK3CG	hsa-miR-422a
PIK3CG	hsa-miR-4309
PIK3CG	hsa-miR-4315
PLA2G2D	hsa-miR-1254
PLA2G2D	hsa-miR-550a-5p
PLA2G2D	hsa-miR-578
PLA2G2D	hsa-miR-650
PLA2G2D	hsa-miR-661
PLA2G2D	hsa-miR-665
PLA2G2D	hsa-miR-765
PLA2G2D	hsa-miR-920
PLA2G2D	hsa-miR-940
PLA2G2D	hsa-miR-383-5p
PLA2G2D	hsa-miR-423-5p
PLA2G2D	hsa-miR-1256
PLA2G2D	hsa-miR-485-5p
PLA2G2D	hsa-miR-486-3p
PLA2G2D	hsa-miR-1275
PLA2G2D	hsa-miR-505-5p
PLA2G2D	hsa-miR-513a-5p
PLA2G2D	hsa-miR-516b-5p
PLA2G2D	hsa-miR-552-3p
PLA2G2D	hsa-miR-1294
PLA2G2D	hsa-miR-562
PLA2G2D	hsa-miR-581
PLA2G2D	hsa-miR-593-5p
PLA2G2D	hsa-miR-597-5p
PLA2G2D	hsa-miR-602
PLA2G2D	hsa-miR-617
PLA2G2D	hsa-miR-623
PLA2G2D	hsa-miR-625-5p
PLA2G2D	hsa-miR-629-3p
PLA2G2D	hsa-miR-631
PLA2G2D	hsa-miR-635
PLA2G2D	hsa-miR-645
PLA2G2D	hsa-miR-647
PLA2G2D	hsa-miR-649
PLA2G2D	hsa-miR-659-3p
PLA2G2D	hsa-miR-668-3p
PLA2G2D	hsa-miR-938
PLA2G2D	hsa-miR-939-5p
PLA2G2D	hsa-miR-9-5p

PLA2G2D	hsa-miR-150-5p
PLA2G2D	hsa-miR-1827
PLA2G2D	hsa-miR-1912
PLA2G2D	hsa-miR-19a-3p
PLA2G2D	hsa-miR-19b-3p
PLA2G2D	hsa-miR-222-5p
PLA2G2D	hsa-miR-299-3p
PLA2G2D	hsa-miR-1197
PLA2G2D	hsa-miR-3116
PLA2G2D	hsa-miR-1205
PLA2G2D	hsa-miR-3176
PLA2G2D	hsa-miR-1207-5p
PLA2G2D	hsa-miR-3184-5p
PLA2G2D	hsa-miR-3612
PLA2G2D	hsa-miR-1226-5p
PLA2G2D	hsa-miR-3620-3p
PLA2G2D	hsa-miR-1233-3p
PLA2G2D	hsa-miR-3714
PLA2G2D	hsa-miR-3915
PLA2G2D	hsa-miR-3919
PLA2G2D	hsa-miR-3922-3p
PLA2G2D	hsa-miR-1243
PLA2G2D	hsa-miR-4254
PLA2G2D	hsa-miR-4265
PLA2G2D	hsa-miR-4284
PLA2G2D	hsa-miR-1248
PLA2G2D	hsa-miR-4322
PLEK	hsa-miR-1253
PLEK	hsa-miR-1269a
PLEK	hsa-miR-490-5p
PLEK	hsa-miR-513c-5p
PLEK	hsa-miR-539-5p
PLEK	hsa-miR-583
PLEK	hsa-miR-634
PLEK	hsa-miR-645
PLEK	hsa-miR-141-3p
PLEK	hsa-miR-146a-5p
PLEK	hsa-miR-181a-5p
PLEK	hsa-miR-181c-5p
PLEK	hsa-miR-206
PLEK	hsa-miR-216a-5p
PLEK	hsa-miR-218-5p
PLEK	hsa-miR-1184
PLEK	hsa-miR-1197
PLEK	hsa-miR-335-5p
PLEK	hsa-miR-34c-3p
PLEK	hsa-miR-1226-3p
PLEK	hsa-miR-422a

PLEK	hsa-miR-1254
PLEK	hsa-miR-1255a
PLEK	hsa-miR-448
PLEK	hsa-miR-1255b-5p
PLEK	hsa-miR-1260a
PLEK	hsa-miR-1265
PLEK	hsa-miR-1270
PLEK	hsa-miR-485-5p
PLEK	hsa-miR-486-3p
PLEK	hsa-miR-494-3p
PLEK	hsa-miR-513a-5p
PLEK	hsa-miR-514b-5p
PLEK	hsa-miR-520a-5p
PLEK	hsa-miR-1285-3p
PLEK	hsa-miR-525-5p
PLEK	hsa-miR-1286
PLEK	hsa-miR-545-3p
PLEK	hsa-miR-1288-3p
PLEK	hsa-miR-548n
PLEK	hsa-miR-1291
PLEK	hsa-miR-558
PLEK	hsa-miR-1292-5p
PLEK	hsa-miR-129-5p
PLEK	hsa-miR-576-3p
PLEK	hsa-miR-579-3p
PLEK	hsa-miR-588
PLEK	hsa-miR-589-3p
PLEK	hsa-miR-590-3p
PLEK	hsa-miR-1299
PLEK	hsa-miR-1301-3p
PLEK	hsa-miR-613
PLEK	hsa-miR-1
PLEK	hsa-miR-615-5p
PLEK	hsa-miR-617
PLEK	hsa-miR-1303
PLEK	hsa-miR-625-5p
PLEK	hsa-miR-636
PLEK	hsa-miR-644a
PLEK	hsa-miR-1306-3p
PLEK	hsa-miR-1323
PLEK	hsa-miR-708-5p
PLEK	hsa-miR-760
PLEK	hsa-miR-765
PLEK	hsa-miR-146b-5p
PLEK	hsa-miR-148a-3p
PLEK	hsa-miR-148b-3p
PLEK	hsa-miR-922
PLEK	hsa-miR-15b-3p

PLEK	hsa-miR-17-3p
PLEK	hsa-miR-181b-5p
PLEK	hsa-miR-181d-5p
PLEK	hsa-miR-185-3p
PLEK	hsa-miR-185-5p
PLEK	hsa-miR-186-5p
PLEK	hsa-miR-200a-3p
PLEK	hsa-miR-205-5p
PLEK	hsa-miR-22-3p
PLEK	hsa-miR-24-3p
PLEK	hsa-miR-27a-3p
PLEK	hsa-miR-28-3p
PLEK	hsa-miR-3138
PLEK	hsa-miR-31-5p
PLEK	hsa-miR-3192-5p
PLEK	hsa-miR-320a
PLEK	hsa-miR-320b
PLEK	hsa-miR-320c
PLEK	hsa-miR-320d
PLEK	hsa-miR-326
PLEK	hsa-miR-331-3p
PLEK	hsa-miR-340-5p
PLEK	hsa-miR-34b-3p
PLEK	hsa-miR-34b-5p
PLEK	hsa-miR-3616-3p
PLEK	hsa-miR-3646
PLEK	hsa-miR-1229-3p
PLEK	hsa-miR-377-3p
PLEK	hsa-miR-378a-3p
PLEK	hsa-miR-378b
PLEK	hsa-miR-378c
PLEK	hsa-miR-382-5p
PLEK	hsa-miR-3915
PLEK	hsa-miR-3922-3p
PLEK	hsa-miR-3925-5p
PLEK	hsa-miR-1244
PLEK	hsa-miR-423-5p
PLEK	hsa-miR-4262
PLEK	hsa-miR-4277
PNOC	hsa-miR-449a
PNOC	hsa-miR-449b-5p
PNOC	hsa-miR-516b-5p
PNOC	hsa-miR-596
PNOC	hsa-miR-222-5p
PNOC	hsa-miR-296-3p
PNOC	hsa-miR-3150a-3p
PNOC	hsa-miR-34a-5p
PRKCB	hsa-miR-449b-5p

PRKCB	hsa-miR-1276
PRKCB	hsa-miR-520g-3p
PRKCB	hsa-miR-520h
PRKCB	hsa-miR-541-3p
PRKCB	hsa-miR-1291
PRKCB	hsa-miR-550a-5p
PRKCB	hsa-miR-129-5p
PRKCB	hsa-miR-573
PRKCB	hsa-miR-1299
PRKCB	hsa-miR-629-3p
PRKCB	hsa-miR-765
PRKCB	hsa-miR-873-5p
PRKCB	hsa-miR-182-5p
PRKCB	hsa-miR-186-5p
PRKCB	hsa-miR-300
PRKCB	hsa-miR-330-3p
PRKCB	hsa-let-7c-5p
PRKCB	hsa-miR-381-3p
PRKCB	hsa-miR-432-5p
PRKCB	hsa-miR-448
PRKCB	hsa-miR-1255b-5p
PRKCB	hsa-let-7e-5p
PRKCB	hsa-miR-449a
PRKCB	hsa-miR-450b-5p
PRKCB	hsa-miR-1260a
PRKCB	hsa-miR-1261
PRKCB	hsa-miR-1267
PRKCB	hsa-let-7f-5p
PRKCB	hsa-miR-484
PRKCB	hsa-miR-490-5p
PRKCB	hsa-miR-491-3p
PRKCB	hsa-miR-491-5p
PRKCB	hsa-miR-495-3p
PRKCB	hsa-miR-511-5p
PRKCB	hsa-miR-513a-5p
PRKCB	hsa-miR-515-3p
PRKCB	hsa-miR-515-5p
PRKCB	hsa-miR-518a-5p
PRKCB	hsa-let-7g-5p
PRKCB	hsa-miR-520a-3p
PRKCB	hsa-miR-520b
PRKCB	hsa-miR-520c-3p
PRKCB	hsa-miR-520d-3p
PRKCB	hsa-miR-520d-5p
PRKCB	hsa-miR-520e
PRKCB	hsa-miR-520f-3p
PRKCB	hsa-miR-524-5p
PRKCB	hsa-miR-527

PRKCB	hsa-miR-539-5p
PRKCB	hsa-miR-1289
PRKCB	hsa-miR-548p
PRKCB	hsa-miR-550a-3p
PRKCB	hsa-miR-550b-3p
PRKCB	hsa-miR-559
PRKCB	hsa-miR-561-3p
PRKCB	hsa-miR-1294
PRKCB	hsa-miR-567
PRKCB	hsa-let-7i-5p
PRKCB	hsa-miR-570-3p
PRKCB	hsa-miR-575
PRKCB	hsa-miR-1296-5p
PRKCB	hsa-miR-583
PRKCB	hsa-miR-587
PRKCB	hsa-miR-1297
PRKCB	hsa-miR-590-3p
PRKCB	hsa-miR-605-5p
PRKCB	hsa-miR-626
PRKCB	hsa-miR-627-5p
PRKCB	hsa-miR-635
PRKCB	hsa-miR-641
PRKCB	hsa-miR-645
PRKCB	hsa-miR-647
PRKCB	hsa-miR-651-5p
PRKCB	hsa-miR-654-5p
PRKCB	hsa-miR-130a-5p
PRKCB	hsa-miR-138-5p
PRKCB	hsa-miR-140-3p
PRKCB	hsa-miR-141-3p
PRKCB	hsa-miR-142-5p
PRKCB	hsa-miR-7-5p
PRKCB	hsa-miR-146a-5p
PRKCB	hsa-miR-146b-5p
PRKCB	hsa-miR-802
PRKCB	hsa-miR-148a-3p
PRKCB	hsa-miR-874-3p
PRKCB	hsa-miR-875-3p
PRKCB	hsa-miR-148b-3p
PRKCB	hsa-miR-890
PRKCB	hsa-miR-891b
PRKCB	hsa-miR-892a
PRKCB	hsa-miR-938
PRKCB	hsa-miR-939-5p
PRKCB	hsa-miR-944
PRKCB	hsa-miR-98-5p
PRKCB	hsa-miR-99b-3p
PRKCB	hsa-miR-152-3p

PRKCB	hsa-miR-105-5p
PRKCB	hsa-miR-183-5p
PRKCB	hsa-let-7a-5p
PRKCB	hsa-miR-185-5p
PRKCB	hsa-miR-188-3p
PRKCB	hsa-miR-194-3p
PRKCB	hsa-miR-197-3p
PRKCB	hsa-miR-198
PRKCB	hsa-miR-199a-3p
PRKCB	hsa-miR-199b-3p
PRKCB	hsa-miR-200a-3p
PRKCB	hsa-miR-200b-3p
PRKCB	hsa-miR-200c-3p
PRKCB	hsa-miR-200c-5p
PRKCB	hsa-miR-203a
PRKCB	hsa-miR-20a-3p
PRKCB	hsa-miR-223-3p
PRKCB	hsa-miR-22-3p
PRKCB	hsa-miR-26a-5p
PRKCB	hsa-miR-26b-5p
PRKCB	hsa-miR-1184
PRKCB	hsa-miR-27a-3p
PRKCB	hsa-miR-27b-3p
PRKCB	hsa-miR-298
PRKCB	hsa-miR-299-3p
PRKCB	hsa-miR-302a-3p
PRKCB	hsa-miR-302b-3p
PRKCB	hsa-miR-302c-3p
PRKCB	hsa-miR-302d-3p
PRKCB	hsa-miR-302e
PRKCB	hsa-let-7b-5p
PRKCB	hsa-miR-30a-3p
PRKCB	hsa-miR-30b-3p
PRKCB	hsa-miR-30c-1-3p
PRKCB	hsa-miR-30c-2-3p
PRKCB	hsa-miR-30d-3p
PRKCB	hsa-miR-1197
PRKCB	hsa-miR-30e-3p
PRKCB	hsa-miR-3129-5p
PRKCB	hsa-miR-1202
PRKCB	hsa-miR-3149
PRKCB	hsa-miR-3153
PRKCB	hsa-miR-1205
PRKCB	hsa-miR-1207-3p
PRKCB	hsa-miR-3192-5p
PRKCB	hsa-miR-320a
PRKCB	hsa-miR-320b
PRKCB	hsa-miR-320c

PRKCB	hsa-miR-320d
PRKCB	hsa-miR-1224-5p
PRKCB	hsa-miR-328-3p
PRKCB	hsa-miR-338-5p
PRKCB	hsa-miR-33b-3p
PRKCB	hsa-miR-33b-5p
PRKCB	hsa-miR-340-5p
PRKCB	hsa-miR-34a-5p
PRKCB	hsa-miR-34c-5p
PRKCB	hsa-miR-3607-3p
PRKCB	hsa-miR-3607-5p
PRKCB	hsa-miR-361-5p
PRKCB	hsa-miR-365a-3p
PRKCB	hsa-miR-3670
PRKCB	hsa-miR-3689a-3p
PRKCB	hsa-miR-3689b-3p
PRKCB	hsa-miR-372-3p
PRKCB	hsa-miR-373-3p
PRKCB	hsa-miR-376a-3p
PRKCB	hsa-miR-376b-3p
PRKCB	hsa-miR-380-3p
PRKCB	hsa-miR-382-5p
PRKCB	hsa-miR-3909
PRKCB	hsa-miR-1244
PRKCB	hsa-let-7d-5p
PRKCB	hsa-miR-4265
PRKCB	hsa-miR-429
PRKCB	hsa-miR-4296
PTPRC	hsa-let-7a-2-3p
PTPRC	hsa-miR-466
PTPRC	hsa-miR-126-5p
PTPRC	hsa-miR-498
PTPRC	hsa-let-7g-3p
PTPRC	hsa-miR-517-5p
PTPRC	hsa-miR-526b-5p
PTPRC	hsa-miR-548i
PTPRC	hsa-miR-548l
PTPRC	hsa-miR-548n
PTPRC	hsa-miR-548y
PTPRC	hsa-miR-587
PTPRC	hsa-miR-138-1-3p
PTPRC	hsa-miR-7-2-3p
PTPRC	hsa-miR-944
PTPRC	hsa-miR-199a-3p
PTPRC	hsa-miR-199b-3p
PTPRC	hsa-miR-302c-5p
PTPRC	hsa-miR-3121-3p
PTPRC	hsa-miR-3129-5p

PTPRC	hsa-miR-3173-3p
PTPRC	hsa-miR-32-3p
PTPRC	hsa-miR-329-3p
PTPRC	hsa-miR-335-3p
PTPRC	hsa-miR-362-3p
PTPRC	hsa-miR-3675-3p
PTPRC	hsa-miR-3692-3p
SELL	hsa-miR-1252-5p
SELL	hsa-miR-485-5p
SELL	hsa-miR-491-5p
SELL	hsa-miR-1276
SELL	hsa-miR-501-5p
SELL	hsa-miR-143-3p
SELL	hsa-miR-144-3p
SELL	hsa-miR-423-5p
SELL	hsa-miR-433-3p
SELL	hsa-miR-454-3p
SELL	hsa-miR-486-3p
SELL	hsa-miR-489-3p
SELL	hsa-miR-494-3p
SELL	hsa-miR-500a-5p
SELL	hsa-miR-507
SELL	hsa-miR-1286
SELL	hsa-miR-545-3p
SELL	hsa-miR-548a-3p
SELL	hsa-miR-548e-3p
SELL	hsa-miR-548f-3p
SELL	hsa-miR-548p
SELL	hsa-miR-557
SELL	hsa-miR-575
SELL	hsa-miR-587
SELL	hsa-miR-603
SELL	hsa-miR-607
SELL	hsa-miR-1301-3p
SELL	hsa-miR-621
SELL	hsa-miR-637
SELL	hsa-miR-1305
SELL	hsa-miR-130a-3p
SELL	hsa-miR-130b-3p
SELL	hsa-miR-1321
SELL	hsa-miR-708-5p
SELL	hsa-miR-148a-3p
SELL	hsa-miR-148b-3p
SELL	hsa-miR-92a-2-5p
SELL	hsa-miR-939-5p
SELL	hsa-miR-942-5p
SELL	hsa-miR-96-5p
SELL	hsa-miR-152-3p

SELL	hsa-miR-105-5p
SELL	hsa-miR-186-5p
SELL	hsa-miR-204-5p
SELL	hsa-miR-28-5p
SELL	hsa-miR-301a-3p
SELL	hsa-miR-301b
SELL	hsa-miR-1193
SELL	hsa-miR-3150a-3p
SELL	hsa-miR-3184-5p
SELL	hsa-miR-3185
SELL	hsa-miR-320a
SELL	hsa-miR-320b
SELL	hsa-miR-320c
SELL	hsa-miR-320d
SELL	hsa-miR-3911
SELL	hsa-miR-3929
SELL	hsa-miR-4311
SELL	hsa-miR-4322
SH2D1A	hsa-miR-1257
SH2D1A	hsa-miR-501-5p
SH2D1A	hsa-miR-1290
SH2D1A	hsa-miR-589-3p
SH2D1A	hsa-miR-1301-3p
SH2D1A	hsa-miR-1305
SH2D1A	hsa-miR-651-5p
SH2D1A	hsa-miR-182-5p
SH2D1A	hsa-miR-31-5p
SH2D1A	hsa-miR-34b-3p
SH2D1A	hsa-miR-361-5p
SH2D1A	hsa-miR-1253
SH2D1A	hsa-miR-496
SH2D1A	hsa-miR-499a-5p
SH2D1A	hsa-miR-500a-5p
SH2D1A	hsa-miR-508-5p
SH2D1A	hsa-miR-510-5p
SH2D1A	hsa-miR-513a-3p
SH2D1A	hsa-miR-516a-3p
SH2D1A	hsa-miR-516b-3p
SH2D1A	hsa-miR-1283
SH2D1A	hsa-miR-520f-3p
SH2D1A	hsa-miR-522-3p
SH2D1A	hsa-miR-532-3p
SH2D1A	hsa-miR-548c-3p
SH2D1A	hsa-miR-548e-3p
SH2D1A	hsa-miR-548f-3p
SH2D1A	hsa-miR-548l
SH2D1A	hsa-miR-593-3p
SH2D1A	hsa-miR-605-5p

SH2D1A	hsa-miR-607
SH2D1A	hsa-miR-641
SH2D1A	hsa-miR-655-3p
SH2D1A	hsa-miR-664a-3p
SH2D1A	hsa-miR-135a-5p
SH2D1A	hsa-miR-135b-5p
SH2D1A	hsa-miR-140-5p
SH2D1A	hsa-miR-142-5p
SH2D1A	hsa-miR-146a-5p
SH2D1A	hsa-miR-770-5p
SH2D1A	hsa-miR-146b-5p
SH2D1A	hsa-miR-885-5p
SH2D1A	hsa-miR-890
SH2D1A	hsa-miR-891b
SH2D1A	hsa-miR-935
SH2D1A	hsa-miR-944
SH2D1A	hsa-miR-192-5p
SH2D1A	hsa-miR-205-5p
SH2D1A	hsa-miR-2115-5p
SH2D1A	hsa-miR-211-5p
SH2D1A	hsa-miR-215-5p
SH2D1A	hsa-miR-1184
SH2D1A	hsa-miR-1206
SH2D1A	hsa-let-7c-3p
SH2D1A	hsa-miR-340-5p
SH2D1A	hsa-miR-362-5p
SH2D1A	hsa-miR-1233-3p
SH2D1A	hsa-miR-369-3p
SH2D1A	hsa-miR-374a-5p
SH2D1A	hsa-miR-374b-5p
SH2D1A	hsa-miR-374c-5p
SIGLEC1	hsa-miR-1254
SIGLEC1	hsa-miR-1255a
SIGLEC1	hsa-miR-516a-3p
SIGLEC1	hsa-miR-516b-3p
SIGLEC1	hsa-miR-520a-5p
SIGLEC1	hsa-miR-525-5p
SIGLEC1	hsa-miR-539-5p
SIGLEC1	hsa-miR-1289
SIGLEC1	hsa-miR-556-5p
SIGLEC1	hsa-miR-653-5p
SIGLEC1	hsa-miR-670-5p
SIGLEC1	hsa-miR-133a-3p
SIGLEC1	hsa-miR-133b
SIGLEC1	hsa-miR-766-3p
SIGLEC1	hsa-miR-936
SIGLEC1	hsa-miR-185-5p
SIGLEC1	hsa-miR-1914-5p

SIGLEC1	hsa-miR-196a-3p
SIGLEC1	hsa-miR-218-5p
SIGLEC1	hsa-miR-222-5p
SIGLEC1	hsa-miR-224-5p
SIGLEC1	hsa-miR-27a-3p
SIGLEC1	hsa-miR-27b-3p
SIGLEC1	hsa-miR-302b-5p
SIGLEC1	hsa-miR-302d-5p
SIGLEC1	hsa-miR-30b-3p
SIGLEC1	hsa-miR-30c-1-3p
SIGLEC1	hsa-miR-30c-2-3p
SIGLEC1	hsa-miR-3116
SIGLEC1	hsa-miR-3128
SIGLEC1	hsa-miR-1200
SIGLEC1	hsa-miR-3138
SIGLEC1	hsa-miR-3154
SIGLEC1	hsa-miR-3158-3p
SIGLEC1	hsa-miR-31-5p
SIGLEC1	hsa-miR-3174
SIGLEC1	hsa-miR-1207-5p
SIGLEC1	hsa-miR-3188
SIGLEC1	hsa-miR-3616-3p
SIGLEC1	hsa-miR-3646
SIGLEC1	hsa-miR-3670
SIGLEC1	hsa-miR-3689a-3p
SIGLEC1	hsa-miR-3689b-3p
SIGLEC1	hsa-miR-3919
SIGLEC1	hsa-miR-4259
SIGLEC1	hsa-miR-4287
SIGLEC1	hsa-miR-4306
SIGLEC1	hsa-miR-4323
SIRPB2	hsa-miR-125a-3p
SIRPB2	hsa-miR-1266-5p
SIRPB2	hsa-miR-517-5p
SIRPB2	hsa-miR-1301-3p
SIRPB2	hsa-miR-612
SIRPB2	hsa-miR-650
SIRPB2	hsa-miR-1321
SIRPB2	hsa-miR-149-5p
SIRPB2	hsa-miR-940
SIRPB2	hsa-miR-185-5p
SIRPB2	hsa-miR-28-3p
SIRPB2	hsa-miR-3150b-3p
SIRPB2	hsa-miR-3158-3p
SIRPB2	hsa-miR-345-5p
SIRPB2	hsa-miR-3612
SIRPB2	hsa-miR-3650
SIRPB2	hsa-miR-3652

SIRPB2	hsa-miR-3661
SIRPB2	hsa-miR-3714
SIRPB2	hsa-miR-3934-5p
SIRPB2	hsa-miR-4270
SIRPB2	hsa-miR-4306
SLAMF1	hsa-miR-671-5p
SLAMF1	hsa-miR-145-5p
SLAMF1	hsa-miR-103a-3p
SLAMF1	hsa-miR-154-5p
SLAMF1	hsa-miR-107
SLAMF1	hsa-miR-219a-1-3p
SLAMF1	hsa-miR-1184
SLAMF1	hsa-miR-1205
SLAMF1	hsa-miR-346
SLAMF1	hsa-miR-450b-5p
SLAMF1	hsa-miR-494-3p
SLAMF1	hsa-miR-507
SLAMF1	hsa-miR-513a-5p
SLAMF1	hsa-miR-548a-5p
SLAMF1	hsa-miR-548c-5p
SLAMF1	hsa-miR-548d-5p
SLAMF1	hsa-miR-548h-5p
SLAMF1	hsa-miR-548j-5p
SLAMF1	hsa-miR-579-3p
SLAMF1	hsa-miR-587
SLAMF1	hsa-miR-589-3p
SLAMF1	hsa-miR-629-5p
SLAMF1	hsa-miR-632
SLAMF1	hsa-miR-130a-5p
SLAMF1	hsa-miR-134-5p
SLAMF1	hsa-miR-758-3p
SLAMF1	hsa-miR-7-5p
SLAMF1	hsa-miR-1827
SLAMF1	hsa-miR-199a-5p
SLAMF1	hsa-miR-199b-5p
SLAMF1	hsa-miR-19a-5p
SLAMF1	hsa-miR-19b-1-5p
SLAMF1	hsa-miR-19b-2-5p
SLAMF1	hsa-miR-205-5p
SLAMF1	hsa-let-7b-3p
SLAMF1	hsa-miR-26a-5p
SLAMF1	hsa-miR-27a-3p
SLAMF1	hsa-miR-27b-3p
SLAMF1	hsa-miR-2861
SLAMF1	hsa-miR-297
SLAMF1	hsa-miR-299-3p
SLAMF1	hsa-miR-30a-5p
SLAMF1	hsa-miR-30d-5p

SLAMF1	hsa-miR-30e-5p
SLAMF1	hsa-miR-3118
SLAMF1	hsa-miR-1200
SLAMF1	hsa-miR-3154
SLAMF1	hsa-miR-3171
SLAMF1	hsa-miR-3180-5p
SLAMF1	hsa-miR-323a-3p
SLAMF1	hsa-miR-323a-5p
SLAMF1	hsa-miR-1226-3p
SLAMF1	hsa-miR-361-5p
SLAMF1	hsa-miR-3659
SLAMF1	hsa-miR-374a-5p
SLAMF1	hsa-miR-374b-5p
SLAMF1	hsa-miR-381-3p
SLAMF1	hsa-miR-4260
SLAMF7	hsa-miR-1253
SLAMF7	hsa-miR-545-3p
SLAMF7	hsa-miR-635
SLAMF7	hsa-miR-326
SLAMF7	hsa-miR-378a-5p
SLAMF7	hsa-miR-1244
SLAMF7	hsa-miR-1257
SLAMF7	hsa-miR-512-3p
SLAMF7	hsa-miR-519a-3p
SLAMF7	hsa-miR-519b-3p
SLAMF7	hsa-miR-519c-3p
SLAMF7	hsa-miR-520b
SLAMF7	hsa-miR-520c-3p
SLAMF7	hsa-miR-520e
SLAMF7	hsa-miR-520f-3p
SLAMF7	hsa-miR-1285-3p
SLAMF7	hsa-miR-532-5p
SLAMF7	hsa-miR-1295a
SLAMF7	hsa-miR-579-3p
SLAMF7	hsa-miR-584-5p
SLAMF7	hsa-miR-1298-5p
SLAMF7	hsa-miR-1301-3p
SLAMF7	hsa-miR-612
SLAMF7	hsa-miR-619-3p
SLAMF7	hsa-miR-629-5p
SLAMF7	hsa-miR-1304-5p
SLAMF7	hsa-miR-1305
SLAMF7	hsa-miR-1324
SLAMF7	hsa-miR-136-5p
SLAMF7	hsa-miR-875-3p
SLAMF7	hsa-miR-885-5p
SLAMF7	hsa-miR-93-5p
SLAMF7	hsa-miR-149-5p

SLAMF7	hsa-miR-17-5p
SLAMF7	hsa-miR-181b-5p
SLAMF7	hsa-miR-181d-5p
SLAMF7	hsa-miR-183-3p
SLAMF7	hsa-miR-190b
SLAMF7	hsa-miR-106b-5p
SLAMF7	hsa-miR-192-5p
SLAMF7	hsa-miR-20b-5p
SLAMF7	hsa-miR-2116-5p
SLAMF7	hsa-miR-216a-5p
SLAMF7	hsa-miR-28-3p
SLAMF7	hsa-miR-3149
SLAMF7	hsa-miR-3150b-3p
SLAMF7	hsa-miR-1224-3p
SLAMF7	hsa-miR-324-3p
SLAMF7	hsa-miR-330-5p
SLAMF7	hsa-miR-342-3p
SLAMF7	hsa-miR-3691-5p
SLAMF7	hsa-miR-3692-5p
SLAMF7	hsa-miR-372-3p
SLAMF7	hsa-miR-4307
SLAMF7	hsa-miR-4314
STAP1	hsa-miR-548a-5p
STAP1	hsa-miR-548b-5p
STAP1	hsa-miR-152-3p
STAP1	hsa-miR-3148
STAP1	hsa-miR-32-3p
TFEC	hsa-miR-448
TFEC	hsa-miR-454-3p
TFEC	hsa-miR-126-5p
TFEC	hsa-miR-1270
TFEC	hsa-let-7f-5p
TFEC	hsa-miR-489-3p
TFEC	hsa-miR-491-3p
TFEC	hsa-miR-497-5p
TFEC	hsa-miR-501-5p
TFEC	hsa-miR-513c-5p
TFEC	hsa-miR-514b-5p
TFEC	hsa-miR-515-5p
TFEC	hsa-let-7g-5p
TFEC	hsa-miR-519e-5p
TFEC	hsa-miR-1284
TFEC	hsa-miR-544a
TFEC	hsa-miR-545-3p
TFEC	hsa-miR-545-5p
TFEC	hsa-miR-561-3p
TFEC	hsa-miR-567
TFEC	hsa-miR-568

TFEC	hsa-let-7i-5p
TFEC	hsa-miR-579-3p
TFEC	hsa-miR-586
TFEC	hsa-miR-590-3p
TFEC	hsa-miR-613
TFEC	hsa-miR-1
TFEC	hsa-miR-620
TFEC	hsa-miR-1305
TFEC	hsa-miR-130a-3p
TFEC	hsa-miR-130a-5p
TFEC	hsa-miR-130b-3p
TFEC	hsa-miR-675-3p
TFEC	hsa-miR-138-2-3p
TFEC	hsa-miR-145-5p
TFEC	hsa-miR-146a-5p
TFEC	hsa-miR-146b-5p
TFEC	hsa-miR-802
TFEC	hsa-miR-148a-5p
TFEC	hsa-miR-888-5p
TFEC	hsa-miR-148b-5p
TFEC	hsa-miR-936
TFEC	hsa-miR-98-5p
TFEC	hsa-miR-153-3p
TFEC	hsa-miR-15a-5p
TFEC	hsa-miR-15b-5p
TFEC	hsa-miR-16-5p
TFEC	hsa-miR-183-3p
TFEC	hsa-let-7a-5p
TFEC	hsa-miR-1912
TFEC	hsa-miR-195-5p
TFEC	hsa-miR-199a-3p
TFEC	hsa-miR-199b-3p
TFEC	hsa-miR-204-5p
TFEC	hsa-miR-205-3p
TFEC	hsa-miR-206
TFEC	hsa-miR-211-5p
TFEC	hsa-miR-216b-5p
TFEC	hsa-miR-222-5p
TFEC	hsa-miR-1182
TFEC	hsa-miR-297
TFEC	hsa-miR-301a-3p
TFEC	hsa-miR-301b
TFEC	hsa-miR-302f
TFEC	hsa-let-7b-5p
TFEC	hsa-miR-30a-3p
TFEC	hsa-miR-30c-1-3p
TFEC	hsa-miR-30d-3p
TFEC	hsa-miR-30e-3p

TFEC	hsa-miR-3126-3p
TFEC	hsa-miR-3148
TFEC	hsa-miR-3156-5p
TFEC	hsa-miR-323a-3p
TFEC	hsa-miR-335-3p
TFEC	hsa-miR-337-3p
TFEC	hsa-miR-338-3p
TFEC	hsa-miR-33a-3p
TFEC	hsa-miR-3606-5p
TFEC	hsa-let-7c-5p
TFEC	hsa-miR-3613-5p
TFEC	hsa-miR-3616-5p
TFEC	hsa-miR-3646
TFEC	hsa-miR-3658
TFEC	hsa-miR-3662
TFEC	hsa-miR-3671
TFEC	hsa-miR-3674
TFEC	hsa-miR-3680-3p
TFEC	hsa-miR-3680-5p
TFEC	hsa-miR-3681-3p
TFEC	hsa-miR-374a-3p
TFEC	hsa-miR-374b-5p
TFEC	hsa-miR-376a-5p
TFEC	hsa-miR-376c-3p
TFEC	hsa-miR-379-3p
TFEC	hsa-miR-380-3p
TFEC	hsa-miR-382-5p
TFEC	hsa-miR-3924
TFEC	hsa-let-7d-5p
TFEC	hsa-miR-411-3p
TFEC	hsa-miR-4251
TFEC	hsa-miR-4263
TFEC	hsa-miR-429
TFEC	hsa-miR-4328
THEMIS	hsa-miR-488-5p
THEMIS	hsa-miR-543
THEMIS	hsa-miR-548g-3p
THEMIS	hsa-miR-548h-5p
THEMIS	hsa-miR-559
THEMIS	hsa-miR-1305
THEMIS	hsa-miR-7-1-3p
THEMIS	hsa-miR-154-5p
THEMIS	hsa-miR-181b-5p
THEMIS	hsa-miR-181c-5p
THEMIS	hsa-miR-181d-5p
THEMIS	hsa-miR-29b-2-5p
THEMIS	hsa-miR-30d-3p
THEMIS	hsa-miR-3148

THEMIS	hsa-miR-3658
TIFAB	hsa-miR-1266-5p
TIFAB	hsa-miR-497-5p
TIFAB	hsa-miR-548a-5p
TIFAB	hsa-miR-548b-5p
TIFAB	hsa-miR-548c-5p
TIFAB	hsa-miR-548d-5p
TIFAB	hsa-miR-548h-5p
TIFAB	hsa-miR-548i
TIFAB	hsa-miR-548j-5p
TIFAB	hsa-miR-548l
TIFAB	hsa-miR-559
TIFAB	hsa-miR-621
TIFAB	hsa-miR-142-5p
TIFAB	hsa-miR-147a
TIFAB	hsa-miR-153-3p
TIFAB	hsa-miR-15a-5p
TIFAB	hsa-miR-15b-5p
TIFAB	hsa-miR-195-5p
TIGIT	hsa-miR-450b-5p
TIGIT	hsa-miR-485-5p
TIGIT	hsa-miR-507
TIGIT	hsa-miR-545-3p
TIGIT	hsa-miR-613
TIGIT	hsa-miR-767-3p
TIGIT	hsa-miR-942-5p
TIGIT	hsa-miR-1253
TIGIT	hsa-miR-1255a
TIGIT	hsa-miR-1257
TIGIT	hsa-miR-483-3p
TIGIT	hsa-miR-518c-5p
TIGIT	hsa-miR-520a-5p
TIGIT	hsa-miR-526b-5p
TIGIT	hsa-miR-548c-3p
TIGIT	hsa-miR-548g-3p
TIGIT	hsa-miR-548o-3p
TIGIT	hsa-miR-548p
TIGIT	hsa-miR-557
TIGIT	hsa-miR-588
TIGIT	hsa-miR-593-3p
TIGIT	hsa-miR-603
TIGIT	hsa-miR-605-5p
TIGIT	hsa-miR-623
TIGIT	hsa-miR-654-3p
TIGIT	hsa-miR-656-3p
TIGIT	hsa-miR-1323
TIGIT	hsa-miR-143-3p
TIGIT	hsa-miR-105-5p

TIGIT	hsa-miR-1976
TIGIT	hsa-miR-203a
TIGIT	hsa-miR-204-5p
TIGIT	hsa-miR-211-5p
TIGIT	hsa-miR-224-3p
TIGIT	hsa-miR-23a-3p
TIGIT	hsa-miR-1185-5p
TIGIT	hsa-miR-30a-3p
TIGIT	hsa-miR-30d-3p
TIGIT	hsa-miR-30e-3p
TIGIT	hsa-miR-3120-3p
TIGIT	hsa-miR-3180-5p
TIGIT	hsa-miR-330-5p
TIGIT	hsa-miR-335-3p
TIGIT	hsa-miR-338-5p
TIGIT	hsa-miR-33a-3p
TIGIT	hsa-miR-345-5p
TIGIT	hsa-miR-1226-3p
TIGIT	hsa-miR-3605-5p
TIGIT	hsa-miR-3680-3p
TIGIT	hsa-miR-3685
TIGIT	hsa-miR-1236-3p
TIGIT	hsa-miR-409-3p
TIGIT	hsa-miR-4264
TLR10	hsa-miR-551b-5p
TLR7	hsa-miR-127-5p
TLR7	hsa-miR-512-3p
TLR7	hsa-miR-519a-3p
TLR7	hsa-miR-519b-3p
TLR7	hsa-miR-520a-3p
TLR7	hsa-miR-520b
TLR7	hsa-miR-520c-3p
TLR7	hsa-miR-520d-3p
TLR7	hsa-miR-520e
TLR7	hsa-miR-130a-3p
TLR7	hsa-miR-659-3p
TLR7	hsa-miR-1323
TLR7	hsa-miR-9-5p
TLR7	hsa-miR-372-3p
TLR7	hsa-miR-432-5p
TLR7	hsa-miR-1255b-5p
TLR7	hsa-miR-449c-5p
TLR7	hsa-miR-454-3p
TLR7	hsa-miR-488-3p
TLR7	hsa-miR-1276
TLR7	hsa-miR-511-5p
TLR7	hsa-miR-519c-3p
TLR7	hsa-miR-520g-3p

TLR7	hsa-miR-520h
TLR7	hsa-miR-525-5p
TLR7	hsa-miR-542-3p
TLR7	hsa-miR-548a-3p
TLR7	hsa-miR-548b-5p
TLR7	hsa-miR-1289
TLR7	hsa-miR-548j-5p
TLR7	hsa-miR-548m
TLR7	hsa-miR-559
TLR7	hsa-miR-568
TLR7	hsa-miR-586
TLR7	hsa-miR-607
TLR7	hsa-miR-629-3p
TLR7	hsa-miR-648
TLR7	hsa-miR-130b-3p
TLR7	hsa-miR-133a-3p
TLR7	hsa-miR-133b
TLR7	hsa-miR-138-2-3p
TLR7	hsa-miR-758-3p
TLR7	hsa-miR-770-5p
TLR7	hsa-miR-93-5p
TLR7	hsa-miR-942-5p
TLR7	hsa-miR-150-5p
TLR7	hsa-miR-17-5p
TLR7	hsa-miR-106b-5p
TLR7	hsa-miR-19a-3p
TLR7	hsa-miR-19b-3p
TLR7	hsa-miR-20b-5p
TLR7	hsa-miR-299-5p
TLR7	hsa-miR-29b-2-5p
TLR7	hsa-miR-301a-3p
TLR7	hsa-miR-301b
TLR7	hsa-miR-302a-3p
TLR7	hsa-miR-302c-3p
TLR7	hsa-miR-3132
TLR7	hsa-miR-1225-3p
TLR7	hsa-miR-340-5p
TLR7	hsa-miR-3666
TLR7	hsa-miR-371a-5p
TLR7	hsa-miR-373-3p
TLR7	hsa-miR-424-5p
TLR7	hsa-miR-4277
TLR8	hsa-miR-1269a
TLR8	hsa-miR-224-3p
TLR8	hsa-miR-3120-3p
TLR8	hsa-miR-323a-5p
TLR8	hsa-miR-3942-5p
TLR8	hsa-miR-4265

TLR8	hsa-miR-4322
TNFRSF17	hsa-miR-150-5p
TNFRSF17	hsa-miR-539-5p
TRAT1	hsa-miR-129-5p
TRAT1	hsa-miR-498
TRAT1	hsa-miR-561-3p
TRAT1	hsa-miR-708-5p
TRAT1	hsa-miR-149-5p
TRAT1	hsa-miR-942-5p
TRAT1	hsa-miR-944
TRAT1	hsa-miR-96-5p
TRAT1	hsa-miR-28-5p
ZNF831	hsa-miR-1256
ZNF831	hsa-miR-449a
ZNF831	hsa-miR-449b-5p
ZNF831	hsa-miR-449c-5p
ZNF831	hsa-miR-485-5p
ZNF831	hsa-miR-487a-3p
ZNF831	hsa-miR-497-5p
ZNF831	hsa-miR-498
ZNF831	hsa-miR-500a-5p
ZNF831	hsa-miR-501-5p
ZNF831	hsa-miR-502-5p
ZNF831	hsa-miR-513a-5p
ZNF831	hsa-miR-515-5p
ZNF831	hsa-miR-518a-5p
ZNF831	hsa-miR-519e-3p
ZNF831	hsa-miR-519e-5p
ZNF831	hsa-miR-522-3p
ZNF831	hsa-miR-527
ZNF831	hsa-miR-539-5p
ZNF831	hsa-miR-544a
ZNF831	hsa-miR-548a-3p
ZNF831	hsa-miR-548a-5p
ZNF831	hsa-miR-548b-5p
ZNF831	hsa-miR-548c-5p
ZNF831	hsa-miR-548d-5p
ZNF831	hsa-miR-548g-3p
ZNF831	hsa-miR-548h-5p
ZNF831	hsa-miR-548i
ZNF831	hsa-miR-548j-5p
ZNF831	hsa-miR-548n
ZNF831	hsa-miR-548w
ZNF831	hsa-miR-548y
ZNF831	hsa-miR-551b-5p
ZNF831	hsa-miR-571
ZNF831	hsa-miR-573
ZNF831	hsa-miR-575

ZNF831	hsa-miR-576-3p
ZNF831	hsa-miR-587
ZNF831	hsa-miR-595
ZNF831	hsa-miR-636
ZNF831	hsa-miR-654-3p
ZNF831	hsa-miR-130b-3p
ZNF831	hsa-miR-1323
ZNF831	hsa-miR-141-3p
ZNF831	hsa-miR-142-3p
ZNF831	hsa-miR-143-3p
ZNF831	hsa-miR-103a-2-5p
ZNF831	hsa-miR-767-5p
ZNF831	hsa-miR-874-3p
ZNF831	hsa-miR-92a-1-5p
ZNF831	hsa-miR-939-5p
ZNF831	hsa-miR-154-3p
ZNF831	hsa-miR-15a-5p
ZNF831	hsa-miR-15b-3p
ZNF831	hsa-miR-15b-5p
ZNF831	hsa-miR-1825
ZNF831	hsa-miR-182-5p
ZNF831	hsa-miR-1827
ZNF831	hsa-miR-183-3p
ZNF831	hsa-miR-185-5p
ZNF831	hsa-miR-1911-3p
ZNF831	hsa-miR-19a-3p
ZNF831	hsa-miR-19a-5p
ZNF831	hsa-miR-19b-1-5p
ZNF831	hsa-miR-19b-2-5p
ZNF831	hsa-miR-19b-3p
ZNF831	hsa-miR-205-3p
ZNF831	hsa-miR-20b-5p
ZNF831	hsa-miR-2110
ZNF831	hsa-miR-2113
ZNF831	hsa-miR-212-3p
ZNF831	hsa-miR-218-5p
ZNF831	hsa-miR-2278
ZNF831	hsa-miR-25-3p
ZNF831	hsa-miR-27a-3p
ZNF831	hsa-miR-27b-3p
ZNF831	hsa-miR-29a-5p
ZNF831	hsa-miR-301b
ZNF831	hsa-miR-302b-5p
ZNF831	hsa-miR-3120-3p
ZNF831	hsa-miR-3130-3p
ZNF831	hsa-miR-3140-3p
ZNF831	hsa-miR-3150a-3p
ZNF831	hsa-miR-3153

ZNF831	hsa-miR-1206
ZNF831	hsa-miR-3183
ZNF831	hsa-miR-3188
ZNF831	hsa-miR-320e
ZNF831	hsa-miR-330-3p
ZNF831	hsa-miR-330-5p
ZNF831	hsa-miR-33b-3p
ZNF831	hsa-miR-33b-5p
ZNF831	hsa-miR-346
ZNF831	hsa-miR-34a-5p
ZNF831	hsa-miR-34b-5p
ZNF831	hsa-miR-34c-5p
ZNF831	hsa-miR-3611
ZNF831	hsa-miR-362-5p
ZNF831	hsa-miR-3658
ZNF831	hsa-miR-3667-5p
ZNF831	hsa-miR-3670
ZNF831	hsa-miR-367-3p
ZNF831	hsa-miR-369-3p
ZNF831	hsa-miR-373-5p
ZNF831	hsa-miR-1236-3p
ZNF831	hsa-miR-3915
ZNF831	hsa-miR-3916
ZNF831	hsa-miR-3929
ZNF831	hsa-miR-409-5p
ZNF831	hsa-miR-424-5p
ZNF831	hsa-miR-4263
ZNF831	hsa-miR-4269
ZNF831	hsa-miR-4270
ZNF831	hsa-miR-4288
ZNF831	hsa-miR-4306
ZNF831	hsa-miR-431-5p
ZNF831	hsa-miR-4318
ZNF831	hsa-miR-4326
ZNF831	hsa-miR-4327

›nship pairs including 76 mRNAs and 913 miRNAs were obtained.
