# Supplementary material for: Transcriptional profile and immune infiltration in colorectal cancer reveal the significance of inducible T‐cell costimulator as a crucial immune checkpoint molecule
Source: Cancer Med. 2024 Mar 20;13(6):e7097. doi: 10.1002/cam4.7097 (PMC10952025; doi:10.1002/cam4.7097)
Supplement: Supplementary file 7 [file CAM4-13-e7097-s005.pdf]

Supplementary file 7. Analyzing 134 mRNAs and 16 lncRNAs that were significantly related t

lncRNA	mRNA	r	p.value	qvalue
RP11-750H9	C3AR1	0.8370784	6.67E-98	1.59E-95
RP11-121A8	C3AR1	0.7441185	4.23E-66	1.01E-64
RP11-284N8	TLR7	0.7373544	2.49E-64	5.28E-63
RP11-750H9	TLR7	0.7827314	2.21E-77	1.39E-75
RP11-121A8	TLR7	0.7093132	1.52E-57	2.20E-56
RP11-284N8	FYB	0.8088088	2.10E-86	2.82E-84
LINC00861	FYB	0.7408317	3.11E-65	6.95E-64
RP11-750H9	FYB	0.7776851	8.85E-76	5.13E-74
RP11-121A8	FYB	0.7401924	4.57E-65	1.01E-63
RP11-284N8	SLAMF1	0.8162464	3.10E-89	4.75E-87
LINC00861	SLAMF1	0.7648904	6.72E-72	2.72E-70
RP11-750H9	SLAMF1	0.7122883	3.16E-58	4.71E-57
CTB-114C7.	CSF3R	0.7176122	1.82E-59	2.90E-58
RP11-750H9	CSF3R	0.7658004	3.63E-72	1.52E-70
RP11-291B2	CD8A	0.7530916	1.56E-68	4.91E-67
RP11-750H9	GPNMB	0.7745992	8.06E-75	4.11E-73
RP11-750H9	LILRB5	0.7210541	2.76E-60	4.66E-59
RP11-284N8	CD28	0.7830588	1.73E-77	1.16E-75
LINC00861	CD28	0.7275038	7.51E-62	1.35E-60
RP11-750H9	CD28	0.7138362	1.39E-58	2.08E-57
RP11-750H9	TFEC	0.7335855	2.28E-63	4.56E-62
RP11-121A8	TFEC	0.7257265	2.05E-61	3.60E-60
RP11-750H9	KCNJ15	0.717908	1.55E-59	2.51E-58
RP11-284N8	MPEG1	0.7740437	1.19E-74	5.96E-73
LINC00861	MPEG1	0.7284381	4.42E-62	8.31E-61
RP11-750H9	MPEG1	0.8052583	4.28E-85	5.39E-83
RP11-121A8	MPEG1	0.7424238	1.19E-65	2.77E-64
RP11-284N8	KLHL6	0.837468	4.47E-98	1.20E-95
LINC00861	KLHL6	0.76873	4.89E-73	2.10E-71
RP11-750H9	KLHL6	0.7542939	7.22E-69	2.38E-67
RP11-121A8	KLHL6	0.7078791	3.21E-57	4.59E-56
RP11-284N8	PRKCB	0.7874429	6.45E-79	5.12E-77
LINC00861	PRKCB	0.7487918	2.35E-67	6.47E-66
RP11-284N8	IL21R	0.7534813	1.21E-68	3.89E-67
RP11-750H9	IL21R	0.7634409	1.78E-71	7.08E-70
RP11-284N8	IKZF1	0.8631891	1.12E-110	6.02E-108
LINC00861	IKZF1	0.8041961	1.04E-84	1.24E-82
RP11-750H9	IKZF1	0.7479975	3.86E-67	1.02E-65
RP11-121A8	IKZF1	0.7391282	8.65E-65	1.87E-63
RP11-284N8	NUGGC	0.7697926	2.35E-73	1.03E-71
RP11-284N8	CCR7	0.7391768	8.40E-65	1.84E-63
LINC00861	CCR7	0.7384892	1.27E-64	2.72E-63
RP11-284N8	CSF2RB	0.7732321	2.12E-74	1.01E-72
RP11-750H9	CSF2RB	0.786387	1.44E-78	1.08E-76
RP11-121A8	CSF2RB	0.7209046	3.00E-60	5.03E-59
RP11-284N8	SCIMP	0.7719963	5.05E-74	2.31E-72

LINC00861	SCIMP	0.7121197	3.46E-58	5.11E-57
RP11-750H9	SCIMP	0.7825679	2.50E-77	1.53E-75
RP11-121A8	SCIMP	0.7269408	1.03E-61	1.83E-60
RP11-750H9	SIRPB2	0.7877058	5.28E-79	4.35E-77
RP11-121A8	SIRPB2	0.7152363	6.55E-59	1.01E-57
RP11-750H9	MSR1	0.7706201	1.32E-73	5.90E-72
RP11-291B2	TBX21	0.7032985	3.41E-56	4.68E-55
RP11-284N8	CYBB	0.7337505	2.07E-63	4.22E-62
RP11-750H9	CYBB	0.8173282	1.17E-89	1.93E-87
RP11-121A8	CYBB	0.7441128	4.25E-66	1.01E-64
CTB-114C7.	LILRA6	0.7008991	1.15E-55	1.55E-54
RP11-750H9	LILRA6	0.742081	1.46E-65	3.34E-64
RP11-284N8	TLR10	0.7863583	1.47E-78	1.08E-76
LINC00861	TLR10	0.7441643	4.12E-66	1.00E-64
RP11-284N8	SELL	0.7343876	1.43E-63	2.94E-62
RP11-284N8	HLA-DOA	0.7289591	3.28E-62	6.28E-61
RP11-750H9	HLA-DOA	0.7423361	1.25E-65	2.89E-64
RP11-284N8	STAP1	0.75153	4.20E-68	1.31E-66
RP11-284N8	CLEC7A	0.7022343	5.86E-56	7.95E-55
CTB-114C7.	CLEC7A	0.7140332	1.25E-58	1.88E-57
RP11-750H9	CLEC7A	0.8224638	1.06E-91	1.89E-89
RP11-121A8	CLEC7A	0.7293838	2.58E-62	4.97E-61
RP11-750H9	FPR3	0.8280677	5.20E-94	1.01E-91
RP11-121A8	FPR3	0.7486561	2.56E-67	6.96E-66
RP11-284N8	LY9	0.850898	2.33E-104	8.34E-102
LINC00861	LY9	0.7443443	3.69E-66	9.09E-65
RP11-750H9	CD80	0.7115856	4.59E-58	6.74E-57
AC104820.2	THEMIS	0.7037831	2.66E-56	3.68E-55
RP11-284N8	THEMIS	0.7633634	1.88E-71	7.32E-70
LINC00861	THEMIS	0.7456419	1.66E-66	4.19E-65
RP11-750H9	FCGR3A	0.7860463	1.86E-78	1.33E-76
RP11-284N8	TIGIT	0.7330622	3.09E-63	6.13E-62
LINC00861	TIGIT	0.7051994	1.29E-56	1.82E-55
RP11-284N8	CD226	0.7213286	2.38E-60	4.04E-59
RP11-750H9	FCGR2B	0.7616436	5.93E-71	2.27E-69
RP11-284N8	PIK3CG	0.7958926	9.03E-82	8.80E-80
LINC00861	PIK3CG	0.715979	4.39E-59	6.87E-58
RP11-750H9	PIK3CG	0.7481508	3.51E-67	9.41E-66
RP11-284N8	PYHIN1	0.7886448	2.58E-79	2.21E-77
LINC00861	PYHIN1	0.7336902	2.14E-63	4.33E-62
RP11-284N8	PLEK	0.7047969	1.58E-56	2.22E-55
CTB-114C7.	PLEK	0.7171972	2.27E-59	3.58E-58
RP11-750H9	PLEK	0.7975279	2.44E-82	2.49E-80
RP11-121A8	PLEK	0.7214099	2.27E-60	3.89E-59
RP11-284N8	ABCD2	0.7504629	8.25E-68	2.46E-66
LINC00861	ABCD2	0.7568689	1.37E-69	4.81E-68
RP11-284N8	CD84	0.7280082	5.64E-62	1.05E-60
RP11-750H9	CD84	0.8142007	1.92E-88	2.74E-86

RP11-121A8	CD84	0.7607423	1.08E-70	3.98E-69
RP11-284N8	SH2D1A	0.7489754	2.10E-67	5.84E-66
RP11-750H9	CD209	0.715051	7.24E-59	1.10E-57
RP11-284N8	P2RY13	0.7500692	1.06E-67	3.10E-66
LINC00861	P2RY13	0.7275227	7.43E-62	1.35E-60
RP11-750H9	P2RY13	0.7495822	1.44E-67	4.10E-66
RP11-121A8	P2RY13	0.7304358	1.41E-62	2.77E-61
RP11-284N8	IL2RA	0.710899	6.59E-58	9.62E-57
RP11-750H9	IL2RA	0.7497691	1.28E-67	3.70E-66
RP11-750H9	LILRB4	0.8039641	1.26E-84	1.42E-82
RP11-121A8	LILRB4	0.7278089	6.32E-62	1.17E-60
RP11-284N8	IRF4	0.8755154	1.16E-117	2.49E-114
LINC00861	IRF4	0.7763436	2.32E-75	1.28E-73
RP11-750H9	CYSLTR2	0.7029403	4.09E-56	5.58E-55
RP11-750H9	MRC1	0.7739007	1.32E-74	6.44E-73
RP11-284N8	CD38	0.7549828	4.63E-69	1.58E-67
RP11-284N8	FCRLA	0.7603965	1.36E-70	4.92E-69
RP11-284N8	PTPRC	0.8361809	1.67E-97	3.58E-95
LINC00861	PTPRC	0.7764605	2.14E-75	1.20E-73
RP11-750H9	PTPRC	0.7746686	7.67E-75	4.01E-73
RP11-121A8	PTPRC	0.749482	1.53E-67	4.31E-66
CTB-114C7.	FPR1	0.7208356	3.12E-60	5.18E-59
RP11-750H9	FPR1	0.7612903	7.50E-71	2.82E-69
RP11-750H9	PDCD1LG2	0.7940844	3.78E-81	3.53E-79
RP11-121A8	PDCD1LG2	0.74607	1.28E-66	3.26E-65
RP11-750H9	FCN1	0.7654486	4.60E-72	1.90E-70
RP11-284N8	CCR2	0.7546886	5.60E-69	1.88E-67
LINC00861	CCR2	0.7003923	1.49E-55	1.97E-54
RP11-750H9	CCR2	0.7504966	8.08E-68	2.44E-66
RP11-121A8	CCR2	0.7081352	2.81E-57	4.04E-56
RP11-284N8	LAX1	0.8580363	5.91E-108	2.53E-105
LINC00861	LAX1	0.7434769	6.27E-66	1.48E-64
RP11-284N8	TRAT1	0.7724765	3.61E-74	1.68E-72
LINC00861	TRAT1	0.722843	1.03E-60	1.78E-59
RP11-284N8	SLAMF7	0.8414708	6.91E-100	2.12E-97
LINC00861	SLAMF7	0.7365374	4.03E-64	8.47E-63
RP11-750H9	SLAMF7	0.7008091	1.21E-55	1.61E-54
RP11-284N8	ZNF831	0.7937591	4.89E-81	4.36E-79
LINC00861	ZNF831	0.7993115	5.79E-83	6.21E-81
RP11-284N8	ICOS	0.7185131	1.11E-59	1.82E-58
LINC00861	ICOS	0.7045575	1.79E-56	2.49E-55
RP11-284N8	GZMK	0.7275742	7.22E-62	1.32E-60
RP11-284N8	CCR4	0.7856087	2.58E-78	1.78E-76
LINC00861	CCR4	0.7575187	8.96E-70	3.20E-68
RP11-750H9	CCR4	0.7178497	1.60E-59	2.57E-58
RP11-284N8	FCRL3	0.7557602	2.81E-69	9.71E-68
LINC00861	FCRL3	0.7154408	5.87E-59	9.12E-58
RP11-284N8	P2RY10	0.7807816	9.31E-77	5.54E-75

LINC00861	P2RY10	0.7537155	1.05E-68	3.40E-67
RP11-284N8	GBP5	0.7206978	3.36E-60	5.55E-59
RP11-750H9	GBP5	0.7274818	7.60E-62	1.36E-60
RP11-284N8	PNOC	0.7465541	9.47E-67	2.48E-65
RP11-284N8	TIFAB	0.7288065	3.58E-62	6.79E-61
RP11-750H9	HRH2	0.7464701	9.97E-67	2.58E-65
RP11-284N8	FCRL5	0.8715347	2.51E-115	1.79E-112
LINC00861	FCRL5	0.7150564	7.22E-59	1.10E-57
RP11-750H9	TLR8	0.7830009	1.81E-77	1.18E-75
RP11-121A8	TLR8	0.7173313	2.11E-59	3.36E-58
RP11-284N8	CR1	0.7350552	9.65E-64	2.01E-62
RP11-750H9	CR1	0.7248361	3.38E-61	5.89E-60
RP11-284N8	KCNA3	0.8722175	1.01E-115	1.08E-112
LINC00861	KCNA3	0.7449873	2.49E-66	6.20E-65
RP11-284N8	IGLL5	0.7751219	5.56E-75	2.98E-73
CTB-114C7.	FPR2	0.705149	1.32E-56	1.86E-55
RP11-750H9	GPR141	0.7505513	7.80E-68	2.39E-66
RP11-284N8	GPR174	0.7410127	2.79E-65	6.30E-64
RP11-284N8	PLA2G2D	0.7294835	2.43E-62	4.74E-61
LINC00861	PLA2G2D	0.7016972	7.70E-56	1.04E-54

:o CD8+ cells, 162 lncRNA-mRNA relationship pairs including 7 lncRNAs and 80 mRNAs were obt

[illegible]ained.
