# Supplementary material for: Transcriptional profile and immune infiltration in colorectal cancer reveal the significance of inducible T‐cell costimulator as a crucial immune checkpoint molecule
Source: Cancer Med. 2024 Mar 20;13(6):e7097. doi: 10.1002/cam4.7097 (PMC10952025; doi:10.1002/cam4.7097)
Supplement: Supplementary file 8 [file CAM4-13-e7097-s007.pdf]

Supplementary file 8. 164 lncRNA-miRNA relationship pairs including 7 lncRNAs and 154 miRN

lncRNA	miRNA
RP11-750H9	hsa-miR-1909-5p
RP11-291B2	hsa-miR-6797-3p
RP11-750H9	hsa-miR-149-3p
LINC00861	hsa-miR-3928-5p
RP11-750H9	hsa-miR-2278
CTB-114C7.	hsa-miR-4755-5p
RP11-750H9	hsa-miR-1253
CTB-114C7.	hsa-miR-5006-3p
RP11-291B2	hsa-miR-1304-3p
LINC00861	hsa-miR-4775
CTB-114C7.	hsa-miR-29a-3p
LINC00861	hsa-miR-513b-5p
LINC00861	hsa-miR-5681a
RP11-121A8	hsa-miR-6839-3p
RP11-121A8	hsa-miR-4654
RP11-291B2	hsa-miR-3920
LINC00861	hsa-miR-3154
RP11-750H9	hsa-miR-5008-5p
AC104820.2	hsa-miR-7152-5p
RP11-121A8	hsa-miR-182-5p
RP11-291B2	hsa-miR-495-3p
LINC00861	hsa-miR-3179
LINC00861	hsa-miR-4797-3p
RP11-750H9	hsa-miR-5197-3p
RP11-750H9	hsa-miR-1827
RP11-121A8	hsa-miR-4742-3p
CTB-114C7.	hsa-miR-3163
LINC00861	hsa-miR-3173-3p
LINC00861	hsa-miR-589-3p
RP11-750H9	hsa-miR-6797-5p
LINC00861	hsa-miR-6806-3p
AC104820.2	hsa-miR-6823-5p
LINC00861	hsa-miR-590-3p
RP11-750H9	hsa-miR-4779
CTB-114C7.	hsa-miR-29c-3p
CTB-114C7.	hsa-miR-6081
LINC00861	hsa-miR-6867-5p
RP11-291B2	hsa-miR-4762-3p
LINC00861	hsa-miR-134-5p
CTB-114C7.	hsa-miR-2117
RP11-121A8	hsa-miR-484
RP11-121A8	hsa-miR-3936
RP11-291B2	hsa-miR-4276
AC104820.2	hsa-miR-6826-5p
LINC00861	hsa-miR-3202
LINC00861	hsa-miR-6757-5p

RP11-121A8	hsa-miR-6778-3p
RP11-750H9	hsa-miR-651-3p
RP11-121A8	hsa-miR-501-5p
CTB-114C7.	hsa-miR-29b-3p
LINC00861	hsa-miR-7515
RP11-121A8	hsa-miR-629-5p
RP11-121A8	hsa-miR-4307
LINC00861	hsa-miR-4724-3p
LINC00861	hsa-miR-3919
LINC00861	hsa-miR-6722-3p
CTB-114C7.	hsa-miR-4496
CTB-114C7.	hsa-miR-105-5p
RP11-750H9	hsa-miR-1249-5p
RP11-750H9	hsa-miR-765
CTB-114C7.	hsa-miR-1205
RP11-121A8	hsa-miR-4999-5p
LINC00861	hsa-miR-6752-5p
RP11-750H9	hsa-miR-6883-5p
AC104820.2	hsa-miR-6875-3p
RP11-121A8	hsa-miR-4282
RP11-750H9	hsa-miR-4468
CTB-114C7.	hsa-miR-3646
LINC00861	hsa-miR-375
CTB-114C7.	hsa-miR-584-3p
LINC00861	hsa-miR-6736-3p
AC104820.2	hsa-miR-889-3p
LINC00861	hsa-miR-6504-5p
LINC00861	hsa-miR-7152-5p
RP11-291B2	hsa-miR-4743-3p
LINC00861	hsa-miR-3928-3p
AC104820.2	hsa-miR-1237-3p
AC104820.2	hsa-miR-340-5p
AC104820.2	hsa-miR-3922-3p
LINC00861	hsa-miR-589-5p
RP11-750H9	hsa-miR-6739-5p
AC104820.2	hsa-miR-875-5p
RP11-750H9	hsa-miR-6124
LINC00861	hsa-miR-4651
LINC00861	hsa-miR-7110-5p
RP11-121A8	hsa-miR-5010-3p
RP11-291B2	hsa-miR-507
LINC00861	hsa-miR-7154-3p
LINC00861	hsa-miR-6515-5p
LINC00861	hsa-miR-940
RP11-121A8	hsa-miR-944
LINC00861	hsa-miR-3123
LINC00861	hsa-miR-3151-5p
RP11-750H9	hsa-miR-3202

RP11-750H9	hsa-miR-3925-5p
LINC00861	hsa-miR-6756-5p
RP11-121A8	hsa-miR-522-3p
LINC00861	hsa-miR-4533
LINC00861	hsa-miR-4762-3p
LINC00861	hsa-miR-6766-5p
CTB-114C7.	hsa-miR-6737-3p
AC104820.2	hsa-miR-5693
RP11-291B2	hsa-miR-3680-3p
LINC00861	hsa-miR-3064-5p
CTB-114C7.	hsa-miR-7853-5p
RP11-121A8	hsa-miR-597-3p
LINC00861	hsa-miR-5582-5p
AC104820.2	hsa-miR-6783-3p
RP11-750H9	hsa-miR-5787
LINC00861	hsa-miR-575
LINC00861	hsa-miR-4305
LINC00861	hsa-miR-3118
RP11-750H9	hsa-miR-7515
LINC00861	hsa-miR-5701
RP11-291B2	hsa-miR-597-3p
AC104820.2	hsa-miR-590-3p
AC104820.2	hsa-miR-664a-3p
LINC00861	hsa-miR-892c-5p
RP11-121A8	hsa-miR-202-3p
CTB-114C7.	hsa-miR-6833-3p
LINC00861	hsa-miR-4419a
LINC00861	hsa-miR-7153-5p
RP11-750H9	hsa-miR-4695-5p
RP11-291B2	hsa-miR-181b-2-3p
RP11-121A8	hsa-miR-2052
CTB-114C7.	hsa-miR-1252-3p
AC104820.2	hsa-miR-561-5p
RP11-121A8	hsa-miR-4803
LINC00861	hsa-miR-4483
RP11-121A8	hsa-miR-580-3p
RP11-291B2	hsa-miR-181b-3p
RP11-750H9	hsa-miR-4311
LINC00861	hsa-miR-6783-5p
LINC00861	hsa-miR-146b-5p
AC104820.2	hsa-miR-4279
LINC00861	hsa-miR-592
RP11-121A8	hsa-miR-548n
LINC00861	hsa-miR-1304-3p
CTB-114C7.	hsa-miR-95-5p
CTB-114C7.	hsa-miR-4433b-3p
RP11-121A8	hsa-miR-6513-3p
RP11-121A8	hsa-miR-6087

RP11-121A8	hsa-miR-3944-5p
LINC00861	hsa-miR-6758-5p
AC104820.2	hsa-miR-4316
LINC00861	hsa-miR-502-5p
RP11-750H9	hsa-miR-4533
RP11-750H9	hsa-miR-3916
LINC00861	hsa-miR-6826-5p
LINC00861	hsa-miR-146a-5p
RP11-121A8	hsa-miR-223-3p
RP11-121A8	hsa-miR-5087
LINC00861	hsa-miR-8063
RP11-121A8	hsa-miR-409-5p
AC104820.2	hsa-miR-4774-3p
LINC00861	hsa-miR-4715-5p
RP11-750H9	hsa-miR-4496
LINC00861	hsa-miR-3714
RP11-750H9	hsa-miR-4756-5p
RP11-750H9	hsa-miR-3915
RP11-121A8	hsa-miR-6796-5p
AC104820.2	hsa-miR-1323
LINC00861	hsa-miR-1252-5p
CTB-114C7.	hsa-miR-6817-3p

NAs
