# Supplementary material for: Transcriptional profile and immune infiltration in colorectal cancer reveal the significance of inducible T‐cell costimulator as a crucial immune checkpoint molecule
Source: Cancer Med. 2024 Mar 20;13(6):e7097. doi: 10.1002/cam4.7097 (PMC10952025; doi:10.1002/cam4.7097)
Supplement: Supplementary file 9 [file CAM4-13-e7097-s008.pdf]

Supplementary file 9. 3,121 miRNA-mRNA relationship pairs including 68 mRNAs and 897 miRNA

| miRNA | mRNA |
|---|---|
| hsa-miR-49 | ABCD2 |
| hsa-miR-50 | ABCD2 |
| hsa-miR-54 | ABCD2 |
| hsa-miR-54 | ABCD2 |
| hsa-miR-54 | ABCD2 |
| hsa-miR-56 | ABCD2 |
| hsa-miR-56 | ABCD2 |
| hsa-miR-57 | ABCD2 |
| hsa-miR-58 | ABCD2 |
| hsa-miR-59 | ABCD2 |
| hsa-miR-60 | ABCD2 |
| hsa-miR-13 | ABCD2 |
| hsa-miR-65 | ABCD2 |
| hsa-miR-13 | ABCD2 |
| hsa-miR-14 | ABCD2 |
| hsa-miR-88 | ABCD2 |
| hsa-miR-94 | ABCD2 |
| hsa-miR-15 | ABCD2 |
| hsa-miR-18 | ABCD2 |
| hsa-miR-10 | ABCD2 |
| hsa-miR-20 | ABCD2 |
| hsa-miR-20 | ABCD2 |
| hsa-miR-20 | ABCD2 |
| hsa-miR-29 | ABCD2 |
| hsa-miR-34 | ABCD2 |
| hsa-miR-42 | ABCD2 |
| hsa-miR-76 | C3AR1 |
| hsa-miR-11 | C3AR1 |
| hsa-miR-12 | C3AR1 |
| hsa-miR-58 | C3AR1 |
| hsa-miR-58 | C3AR1 |
| hsa-miR-76 | C3AR1 |
| hsa-miR-31 | C3AR1 |
| hsa-miR-12 | C3AR1 |
| hsa-miR-48 | CCR2 |
| hsa-miR-15 | CCR2 |
| hsa-miR-21 | CCR2 |
| hsa-miR-12 | CCR4 |
| hsa-miR-32 | CCR4 |
| hsa-miR-32 | CCR4 |
| hsa-miR-32 | CCR4 |
| hsa-miR-32 | CCR4 |
| hsa-let-7e | CCR7 |
| hsa-let-7f | CCR7 |
| hsa-miR-61 | CCR7 |
| hsa-miR-64 | CCR7 |

| | |
|---|---|
| hsa-miR-98 | CCR7 |
| hsa-let-7a | CCR7 |
| hsa-miR-21 | CCR7 |
| hsa-miR-29 | CCR7 |
| hsa-let-7b | CCR7 |
| hsa-let-7c | CCR7 |
| hsa-miR-43 | CCR7 |
| hsa-miR-12 | CCR7 |
| hsa-miR-48 | CCR7 |
| hsa-miR-51 | CCR7 |
| hsa-let-7g | CCR7 |
| hsa-miR-53 | CCR7 |
| hsa-miR-54 | CCR7 |
| hsa-let-7i | CCR7 |
| hsa-miR-12 | CCR7 |
| hsa-miR-57 | CCR7 |
| hsa-miR-12 | CCR7 |
| hsa-miR-58 | CCR7 |
| hsa-miR-12 | CCR7 |
| hsa-miR-62 | CCR7 |
| hsa-miR-63 | CCR7 |
| hsa-miR-64 | CCR7 |
| hsa-miR-64 | CCR7 |
| hsa-miR-65 | CCR7 |
| hsa-miR-65 | CCR7 |
| hsa-miR-65 | CCR7 |
| hsa-miR-66 | CCR7 |
| hsa-miR-76 | CCR7 |
| hsa-miR-14 | CCR7 |
| hsa-miR-19 | CCR7 |
| hsa-miR-19 | CCR7 |
| hsa-miR-19 | CCR7 |
| hsa-miR-21 | CCR7 |
| hsa-miR-30 | CCR7 |
| hsa-miR-30 | CCR7 |
| hsa-miR-30 | CCR7 |
| hsa-miR-31 | CCR7 |
| hsa-miR-31 | CCR7 |
| hsa-miR-31 | CCR7 |
| hsa-miR-32 | CCR7 |
| hsa-miR-32 | CCR7 |
| hsa-miR-32 | CCR7 |
| hsa-miR-32 | CCR7 |
| hsa-miR-33 | CCR7 |
| hsa-miR-36 | CCR7 |
| hsa-miR-36 | CCR7 |
| hsa-miR-36 | CCR7 |
| hsa-let-7c | CCR7 |

| | |
|---|---|
| hsa-miR-40 | CCR7 |
| hsa-miR-12 | CD209 |
| hsa-miR-12 | CD209 |
| hsa-miR-57 | CD209 |
| hsa-miR-59 | CD209 |
| hsa-miR-34 | CD209 |
| hsa-miR-12 | CD209 |
| hsa-miR-12 | CD209 |
| hsa-miR-12 | CD209 |
| hsa-miR-49 | CD209 |
| hsa-miR-12 | CD209 |
| hsa-miR-50 | CD209 |
| hsa-miR-51 | CD209 |
| hsa-miR-12 | CD209 |
| hsa-miR-53 | CD209 |
| hsa-miR-12 | CD209 |
| hsa-miR-12 | CD209 |
| hsa-miR-54 | CD209 |
| hsa-miR-55 | CD209 |
| hsa-miR-57 | CD209 |
| hsa-miR-58 | CD209 |
| hsa-miR-58 | CD209 |
| hsa-miR-60 | CD209 |
| hsa-miR-61 | CD209 |
| hsa-miR-63 | CD209 |
| hsa-miR-64 | CD209 |
| hsa-miR-65 | CD209 |
| hsa-miR-66 | CD209 |
| hsa-miR-67 | CD209 |
| hsa-miR-70 | CD209 |
| hsa-miR-74 | CD209 |
| hsa-miR-76 | CD209 |
| hsa-miR-76 | CD209 |
| hsa-miR-76 | CD209 |
| hsa-miR-87 | CD209 |
| hsa-miR-89 | CD209 |
| hsa-miR-94 | CD209 |
| hsa-miR-18 | CD209 |
| hsa-miR-18 | CD209 |
| hsa-miR-18 | CD209 |
| hsa-miR-18 | CD209 |
| hsa-miR-18 | CD209 |
| hsa-miR-21 | CD209 |
| hsa-miR-24 | CD209 |
| hsa-miR-28 | CD209 |
| hsa-miR-29 | CD209 |
| hsa-miR-12 | CD209 |
| hsa-miR-31 | CD209 |

| | |
|---|---|
| hsa-miR-31 | CD209 |
| hsa-miR-33 | CD209 |
| hsa-miR-34 | CD209 |
| hsa-miR-36 | CD209 |
| hsa-miR-12 | CD209 |
| hsa-miR-39 | CD209 |
| hsa-miR-42 | CD209 |
| hsa-miR-42 | CD209 |
| hsa-miR-42 | CD209 |
| hsa-miR-50 | CD226 |
| hsa-miR-51 | CD226 |
| hsa-miR-87 | CD226 |
| hsa-miR-17 | CD226 |
| hsa-miR-18 | CD226 |
| hsa-miR-20 | CD226 |
| hsa-miR-21 | CD226 |
| hsa-miR-44 | CD28 |
| hsa-miR-45 | CD28 |
| hsa-miR-12 | CD28 |
| hsa-miR-12 | CD28 |
| hsa-miR-49 | CD28 |
| hsa-miR-49 | CD28 |
| hsa-miR-49 | CD28 |
| hsa-miR-49 | CD28 |
| hsa-miR-49 | CD28 |
| hsa-miR-49 | CD28 |
| hsa-miR-50 | CD28 |
| hsa-miR-51 | CD28 |
| hsa-miR-51 | CD28 |
| hsa-miR-52 | CD28 |
| hsa-miR-52 | CD28 |
| hsa-miR-52 | CD28 |
| hsa-miR-12 | CD28 |
| hsa-miR-52 | CD28 |
| hsa-miR-52 | CD28 |
| hsa-miR-54 | CD28 |
| hsa-miR-54 | CD28 |
| hsa-miR-54 | CD28 |
| hsa-miR-54 | CD28 |
| hsa-miR-55 | CD28 |
| hsa-miR-57 | CD28 |
| hsa-miR-57 | CD28 |
| hsa-miR-57 | CD28 |
| hsa-miR-58 | CD28 |
| hsa-miR-58 | CD28 |
| hsa-miR-59 | CD28 |
| hsa-miR-59 | CD28 |
| hsa-miR-60 | CD28 |

| | |
|---|---|
| hsa-miR-60 | CD28 |
| hsa-miR-12 | CD28 |
| hsa-miR-61 | CD28 |
| hsa-miR-1 | CD28 |
| hsa-miR-61 | CD28 |
| hsa-miR-62 | CD28 |
| hsa-miR-63 | CD28 |
| hsa-miR-63 | CD28 |
| hsa-miR-65 | CD28 |
| hsa-miR-65 | CD28 |
| hsa-miR-66 | CD28 |
| hsa-miR-10 | CD28 |
| hsa-miR-13 | CD28 |
| hsa-miR-14 | CD28 |
| hsa-miR-14 | CD28 |
| hsa-miR-70 | CD28 |
| hsa-miR-75 | CD28 |
| hsa-miR-88 | CD28 |
| hsa-miR-93 | CD28 |
| hsa-miR-96 | CD28 |
| hsa-miR-15 | CD28 |
| hsa-miR-15 | CD28 |
| hsa-miR-15 | CD28 |
| hsa-miR-16 | CD28 |
| hsa-miR-17 | CD28 |
| hsa-miR-18 | CD28 |
| hsa-miR-10 | CD28 |
| hsa-miR-18 | CD28 |
| hsa-miR-10 | CD28 |
| hsa-miR-19 | CD28 |
| hsa-miR-20 | CD28 |
| hsa-miR-20 | CD28 |
| hsa-miR-20 | CD28 |
| hsa-miR-11 | CD28 |
| hsa-miR-22 | CD28 |
| hsa-miR-24 | CD28 |
| hsa-miR-11 | CD28 |
| hsa-miR-27 | CD28 |
| hsa-miR-27 | CD28 |
| hsa-miR-28 | CD28 |
| hsa-miR-29 | CD28 |
| hsa-miR-30 | CD28 |
| hsa-miR-12 | CD28 |
| hsa-miR-31 | CD28 |
| hsa-miR-12 | CD28 |
| hsa-miR-33 | CD28 |
| hsa-miR-33 | CD28 |
| hsa-miR-34 | CD28 |

| | |
|---|---|
| hsa-miR-12 | CD28 |
| hsa-miR-36 | CD28 |
| hsa-miR-37 | CD28 |
| hsa-miR-37 | CD28 |
| hsa-miR-12 | CD28 |
| hsa-miR-38 | CD28 |
| hsa-miR-38 | CD28 |
| hsa-miR-42 | CD28 |
| hsa-miR-12 | CD28 |
| hsa-miR-49 | CD38 |
| hsa-miR-57 | CD38 |
| hsa-miR-57 | CD38 |
| hsa-miR-62 | CD38 |
| hsa-miR-12 | CD80 |
| hsa-miR-57 | CD80 |
| hsa-let-7e | CD80 |
| hsa-miR-12 | CD80 |
| hsa-let-7f | CD80 |
| hsa-miR-49 | CD80 |
| hsa-miR-51 | CD80 |
| hsa-miR-51 | CD80 |
| hsa-miR-51 | CD80 |
| hsa-let-7g | CD80 |
| hsa-miR-52 | CD80 |
| hsa-miR-54 | CD80 |
| hsa-let-7i | CD80 |
| hsa-miR-58 | CD80 |
| hsa-miR-59 | CD80 |
| hsa-miR-60 | CD80 |
| hsa-miR-13 | CD80 |
| hsa-miR-62 | CD80 |
| hsa-miR-63 | CD80 |
| hsa-miR-13 | CD80 |
| hsa-miR-14 | CD80 |
| hsa-miR-10 | CD80 |
| hsa-miR-92 | CD80 |
| hsa-miR-14 | CD80 |
| hsa-miR-98 | CD80 |
| hsa-let-7a | CD80 |
| hsa-miR-10 | CD80 |
| hsa-miR-21 | CD80 |
| hsa-miR-22 | CD80 |
| hsa-miR-29 | CD80 |
| hsa-miR-29 | CD80 |
| hsa-miR-29 | CD80 |
| hsa-let-7b | CD80 |
| hsa-miR-31 | CD80 |
| hsa-miR-32 | CD80 |

| | |
|---|---|
| hsa-miR-33 | CD80 |
| hsa-miR-12 | CD80 |
| hsa-let-7c | CD80 |
| hsa-let-7c | CD80 |
| hsa-miR-42 | CD80 |
| hsa-miR-43 | CD80 |
| hsa-miR-12 | CD84 |
| hsa-miR-12 | CD84 |
| hsa-miR-12 | CD84 |
| hsa-miR-45 | CD84 |
| hsa-miR-12 | CD84 |
| hsa-miR-45 | CD84 |
| hsa-miR-12 | CD84 |
| hsa-miR-12 | CD84 |
| hsa-miR-12 | CD84 |
| hsa-miR-48 | CD84 |
| hsa-miR-48 | CD84 |
| hsa-miR-49 | CD84 |
| hsa-miR-50 | CD84 |
| hsa-miR-51 | CD84 |
| hsa-miR-51 | CD84 |
| hsa-miR-51 | CD84 |
| hsa-miR-51 | CD84 |
| hsa-miR-51 | CD84 |
| hsa-miR-51 | CD84 |
| hsa-miR-51 | CD84 |
| hsa-miR-51 | CD84 |
| hsa-miR-51 | CD84 |
| hsa-miR-51 | CD84 |
| hsa-miR-51 | CD84 |
| hsa-miR-51 | CD84 |
| hsa-miR-51 | CD84 |
| hsa-miR-51 | CD84 |
| hsa-miR-51 | CD84 |
| hsa-miR-51 | CD84 |
| hsa-miR-51 | CD84 |
| hsa-miR-52 | CD84 |
| hsa-miR-52 | CD84 |
| hsa-miR-52 | CD84 |
| hsa-miR-52 | CD84 |
| hsa-miR-52 | CD84 |
| hsa-miR-52 | CD84 |
| hsa-miR-52 | CD84 |
| hsa-miR-52 | CD84 |
| hsa-miR-52 | CD84 |
| hsa-miR-12 | CD84 |
| hsa-miR-52 | CD84 |
| hsa-miR-52 | CD84 |

| | |
|---|---|
| hsa-miR-52 | CD84 |
| hsa-miR-52 | CD84 |
| hsa-miR-52 | CD84 |
| hsa-miR-53 | CD84 |
| hsa-miR-54 | CD84 |
| hsa-miR-12 | CD84 |
| hsa-miR-54 | CD84 |
| hsa-miR-54 | CD84 |
| hsa-miR-54 | CD84 |
| hsa-miR-54 | CD84 |
| hsa-miR-54 | CD84 |
| hsa-miR-54 | CD84 |
| hsa-miR-54 | CD84 |
| hsa-miR-54 | CD84 |
| hsa-miR-54 | CD84 |
| hsa-miR-55 | CD84 |
| hsa-miR-55 | CD84 |
| hsa-miR-55 | CD84 |
| hsa-miR-56 | CD84 |
| hsa-miR-57 | CD84 |
| hsa-miR-57 | CD84 |
| hsa-miR-58 | CD84 |
| hsa-miR-58 | CD84 |
| hsa-miR-59 | CD84 |
| hsa-miR-59 | CD84 |
| hsa-miR-60 | CD84 |
| hsa-miR-12 | CD84 |
| hsa-miR-13 | CD84 |
| hsa-miR-61 | CD84 |
| hsa-miR-61 | CD84 |
| hsa-miR-62 | CD84 |
| hsa-miR-64 | CD84 |
| hsa-miR-64 | CD84 |
| hsa-miR-65 | CD84 |
| hsa-let-7a | CD84 |
| hsa-miR-13 | CD84 |
| hsa-miR-13 | CD84 |
| hsa-miR-13 | CD84 |
| hsa-miR-14 | CD84 |
| hsa-miR-14 | CD84 |
| hsa-miR-14 | CD84 |
| hsa-miR-70 | CD84 |
| hsa-miR-10 | CD84 |
| hsa-miR-14 | CD84 |
| hsa-miR-7- | CD84 |
| hsa-miR-76 | CD84 |
| hsa-miR-76 | CD84 |
| hsa-miR-76 | CD84 |

| | |
|---|---|
| hsa-miR-76 | CD84 |
| hsa-miR-87 | CD84 |
| hsa-miR-87 | CD84 |
| hsa-miR-89 | CD84 |
| hsa-miR-14 | CD84 |
| hsa-miR-92 | CD84 |
| hsa-miR-92 | CD84 |
| hsa-miR-93 | CD84 |
| hsa-miR-93 | CD84 |
| hsa-miR-94 | CD84 |
| hsa-miR-94 | CD84 |
| hsa-miR-9- | CD84 |
| hsa-miR-15 | CD84 |
| hsa-miR-15 | CD84 |
| hsa-miR-15 | CD84 |
| hsa-miR-17 | CD84 |
| hsa-miR-10 | CD84 |
| hsa-miR-18 | CD84 |
| hsa-miR-18 | CD84 |
| hsa-miR-10 | CD84 |
| hsa-miR-19 | CD84 |
| hsa-miR-19 | CD84 |
| hsa-miR-19 | CD84 |
| hsa-miR-19 | CD84 |
| hsa-miR-19 | CD84 |
| hsa-miR-19 | CD84 |
| hsa-miR-19 | CD84 |
| hsa-miR-19 | CD84 |
| hsa-miR-20 | CD84 |
| hsa-miR-20 | CD84 |
| hsa-miR-20 | CD84 |
| hsa-miR-20 | CD84 |
| hsa-miR-21 | CD84 |
| hsa-miR-21 | CD84 |
| hsa-let-7b | CD84 |
| hsa-miR-21 | CD84 |
| hsa-miR-21 | CD84 |
| hsa-miR-22 | CD84 |
| hsa-miR-22 | CD84 |
| hsa-miR-26 | CD84 |
| hsa-miR-26 | CD84 |
| hsa-miR-26 | CD84 |
| hsa-miR-28 | CD84 |
| hsa-miR-30 | CD84 |
| hsa-miR-30 | CD84 |
| hsa-miR-30 | CD84 |
| hsa-miR-30 | CD84 |
| hsa-miR-30 | CD84 |

| | |
|---|---|
| hsa-miR-11 | CD84 |
| hsa-miR-31 | CD84 |
| hsa-miR-31 | CD84 |
| hsa-miR-31 | CD84 |
| hsa-miR-31 | CD84 |
| hsa-miR-31 | CD84 |
| hsa-miR-12 | CD84 |
| hsa-miR-31 | CD84 |
| hsa-miR-31 | CD84 |
| hsa-miR-31 | CD84 |
| hsa-miR-31 | CD84 |
| hsa-miR-31 | CD84 |
| hsa-miR-31 | CD84 |
| hsa-miR-31 | CD84 |
| hsa-miR-31 | CD84 |
| hsa-miR-31 | CD84 |
| hsa-miR-31 | CD84 |
| hsa-miR-31 | CD84 |
| hsa-miR-31 | CD84 |
| hsa-miR-31 | CD84 |
| hsa-miR-12 | CD84 |
| hsa-miR-31 | CD84 |
| hsa-miR-31 | CD84 |
| hsa-miR-31 | CD84 |
| hsa-miR-31 | CD84 |
| hsa-miR-32 | CD84 |
| hsa-miR-33 | CD84 |
| hsa-miR-33 | CD84 |
| hsa-miR-33 | CD84 |
| hsa-miR-12 | CD84 |
| hsa-miR-34 | CD84 |
| hsa-miR-12 | CD84 |
| hsa-miR-36 | CD84 |
| hsa-miR-36 | CD84 |
| hsa-miR-36 | CD84 |
| hsa-miR-36 | CD84 |
| hsa-miR-36 | CD84 |
| hsa-miR-36 | CD84 |
| hsa-miR-36 | CD84 |
| hsa-miR-36 | CD84 |
| hsa-miR-36 | CD84 |
| hsa-miR-36 | CD84 |
| hsa-miR-36 | CD84 |
| hsa-miR-36 | CD84 |
| hsa-miR-36 | CD84 |
| hsa-miR-36 | CD84 |
| hsa-miR-37 | CD84 |
| hsa-miR-37 | CD84 |

| | |
|---|---|
| hsa-miR-37 | CD84 |
| hsa-miR-12 | CD84 |
| hsa-miR-38 | CD84 |
| hsa-miR-39 | CD84 |
| hsa-miR-39 | CD84 |
| hsa-miR-39 | CD84 |
| hsa-miR-39 | CD84 |
| hsa-miR-39 | CD84 |
| hsa-miR-12 | CD84 |
| hsa-miR-42 | CD84 |
| hsa-miR-42 | CD84 |
| hsa-miR-42 | CD84 |
| hsa-miR-42 | CD84 |
| hsa-miR-42 | CD84 |
| hsa-miR-42 | CD84 |
| hsa-miR-42 | CD84 |
| hsa-miR-42 | CD84 |
| hsa-miR-43 | CD84 |
| hsa-miR-43 | CD84 |
| hsa-miR-43 | CD84 |
| hsa-miR-43 | CD84 |
| hsa-miR-43 | CD84 |
| hsa-miR-43 | CD84 |
| hsa-miR-43 | CD84 |
| hsa-miR-12 | CD8A |
| hsa-miR-58 | CD8A |
| hsa-miR-59 | CD8A |
| hsa-miR-76 | CD8A |
| hsa-miR-14 | CD8A |
| hsa-miR-14 | CD8A |
| hsa-miR-21 | CD8A |
| hsa-miR-30 | CD8A |
| hsa-miR-30 | CD8A |
| hsa-miR-33 | CD8A |
| hsa-miR-42 | CD8A |
| hsa-miR-42 | CD8A |
| hsa-miR-58 | CLEC7A |
| hsa-miR-64 | CLEC7A |
| hsa-miR-65 | CLEC7A |
| hsa-miR-10 | CLEC7A |
| hsa-miR-87 | CLEC7A |
| hsa-miR-36 | CLEC7A |
| hsa-miR-36 | CLEC7A |
| hsa-miR-37 | CLEC7A |
| hsa-miR-54 | CR1 |
| hsa-miR-62 | CR1 |
| hsa-miR-14 | CR1 |
| hsa-miR-45 | CSF2RB |

| | |
|---|---|
| hsa-miR-54 | CSF2RB |
| hsa-miR-54 | CSF2RB |
| hsa-miR-54 | CSF2RB |
| hsa-miR-59 | CSF2RB |
| hsa-miR-67 | CSF2RB |
| hsa-miR-18 | CSF2RB |
| hsa-miR-18 | CSF2RB |
| hsa-miR-19 | CSF2RB |
| hsa-miR-19 | CSF2RB |
| hsa-miR-23 | CSF2RB |
| hsa-miR-31 | CSF2RB |
| hsa-miR-67 | CSF3R |
| hsa-miR-30 | CSF3R |
| hsa-miR-36 | CSF3R |
| hsa-miR-36 | CSF3R |
| hsa-miR-12 | CYBB |
| hsa-miR-48 | CYBB |
| hsa-miR-49 | CYBB |
| hsa-miR-12 | CYBB |
| hsa-miR-52 | CYBB |
| hsa-miR-52 | CYBB |
| hsa-miR-59 | CYBB |
| hsa-miR-60 | CYBB |
| hsa-miR-13 | CYBB |
| hsa-miR-65 | CYBB |
| hsa-miR-13 | CYBB |
| hsa-miR-13 | CYBB |
| hsa-miR-75 | CYBB |
| hsa-miR-14 | CYBB |
| hsa-miR-87 | CYBB |
| hsa-miR-9- | CYBB |
| hsa-miR-10 | CYBB |
| hsa-miR-18 | CYBB |
| hsa-miR-20 | CYBB |
| hsa-miR-27 | CYBB |
| hsa-miR-27 | CYBB |
| hsa-miR-29 | CYBB |
| hsa-miR-30 | CYBB |
| hsa-miR-30 | CYBB |
| hsa-miR-12 | CYBB |
| hsa-miR-37 | CYBB |
| hsa-miR-37 | CYBB |
| hsa-miR-12 | CYBB |
| hsa-miR-42 | CYBB |
| hsa-miR-12 | CYBB |
| hsa-miR-12 | CYBB |
| hsa-miR-12 | CYBB |
| hsa-miR-51 | CYBB |

| | |
|---|---|
| hsa-miR-12 | CYBB |
| hsa-miR-51 | CYBB |
| hsa-miR-52 | CYBB |
| hsa-miR-52 | CYBB |
| hsa-miR-12 | CYBB |
| hsa-miR-52 | CYBB |
| hsa-miR-52 | CYBB |
| hsa-miR-12 | CYBB |
| hsa-miR-54 | CYBB |
| hsa-miR-54 | CYBB |
| hsa-miR-54 | CYBB |
| hsa-miR-54 | CYBB |
| hsa-miR-12 | CYBB |
| hsa-miR-57 | CYBB |
| hsa-miR-58 | CYBB |
| hsa-miR-58 | CYBB |
| hsa-miR-58 | CYBB |
| hsa-miR-12 | CYBB |
| hsa-miR-13 | CYBB |
| hsa-miR-64 | CYBB |
| hsa-miR-64 | CYBB |
| hsa-miR-65 | CYBB |
| hsa-miR-66 | CYBB |
| hsa-miR-13 | CYBB |
| hsa-miR-14 | CYBB |
| hsa-miR-14 | CYBB |
| hsa-miR-76 | CYBB |
| hsa-miR-14 | CYBB |
| hsa-miR-14 | CYBB |
| hsa-miR-87 | CYBB |
| hsa-miR-87 | CYBB |
| hsa-miR-88 | CYBB |
| hsa-miR-14 | CYBB |
| hsa-miR-92 | CYBB |
| hsa-miR-93 | CYBB |
| hsa-miR-93 | CYBB |
| hsa-miR-94 | CYBB |
| hsa-miR-15 | CYBB |
| hsa-miR-15 | CYBB |
| hsa-miR-18 | CYBB |
| hsa-miR-18 | CYBB |
| hsa-miR-18 | CYBB |
| hsa-miR-20 | CYBB |
| hsa-miR-20 | CYBB |
| hsa-miR-20 | CYBB |
| hsa-miR-20 | CYBB |
| hsa-miR-21 | CYBB |
| hsa-miR-21 | CYBB |

| | |
|---|---|
| hsa-miR-26 | CYBB |
| hsa-miR-29 | CYBB |
| hsa-miR-29 | CYBB |
| hsa-miR-30 | CYBB |
| hsa-miR-30 | CYBB |
| hsa-miR-30 | CYBB |
| hsa-miR-30 | CYBB |
| hsa-miR-30 | CYBB |
| hsa-miR-31 | CYBB |
| hsa-miR-31 | CYBB |
| hsa-miR-31 | CYBB |
| hsa-miR-32 | CYBB |
| hsa-miR-34 | CYBB |
| hsa-miR-36 | CYBB |
| hsa-miR-36 | CYBB |
| hsa-miR-36 | CYBB |
| hsa-miR-36 | CYBB |
| hsa-miR-36 | CYBB |
| hsa-miR-36 | CYBB |
| hsa-miR-37 | CYBB |
| hsa-miR-37 | CYBB |
| hsa-miR-12 | CYBB |
| hsa-miR-42 | CYBB |
| hsa-miR-42 | CYBB |
| hsa-miR-42 | CYBB |
| hsa-miR-42 | CYBB |
| hsa-miR-43 | CYBB |
| hsa-miR-50 | CYSLTR2 |
| hsa-miR-12 | CYSLTR2 |
| hsa-miR-14 | CYSLTR2 |
| hsa-miR-92 | CYSLTR2 |
| hsa-miR-93 | CYSLTR2 |
| hsa-miR-93 | CYSLTR2 |
| hsa-miR-11 | CYSLTR2 |
| hsa-miR-31 | CYSLTR2 |
| hsa-miR-31 | CYSLTR2 |
| hsa-miR-36 | CYSLTR2 |
| hsa-miR-43 | CYSLTR2 |
| hsa-miR-12 | FCGR2B |
| hsa-miR-64 | FCGR2B |
| hsa-miR-31 | FCGR2B |
| hsa-miR-39 | FCGR2B |
| hsa-miR-12 | FCGR3A |
| hsa-miR-12 | FCGR3A |
| hsa-miR-52 | FCGR3A |
| hsa-miR-52 | FCGR3A |
| hsa-miR-55 | FCGR3A |
| hsa-miR-12 | FCGR3A |

| | |
|---|---|
| hsa-miR-62 | FCGR3A |
| hsa-miR-63 | FCGR3A |
| hsa-miR-14 | FCGR3A |
| hsa-miR-7- | FCGR3A |
| hsa-miR-89 | FCGR3A |
| hsa-miR-14 | FCGR3A |
| hsa-miR-21 | FCGR3A |
| hsa-miR-33 | FCGR3A |
| hsa-miR-37 | FCGR3A |
| hsa-miR-37 | FCGR3A |
| hsa-miR-40 | FCGR3A |
| hsa-miR-42 | FCGR3A |
| hsa-miR-36 | FCN1 |
| hsa-miR-12 | FCRL5 |
| hsa-miR-12 | FCRL5 |
| hsa-miR-51 | FCRL5 |
| hsa-miR-18 | FCRL5 |
| hsa-miR-33 | FCRL5 |
| hsa-miR-42 | FCRL5 |
| hsa-miR-44 | FCRL5 |
| hsa-miR-12 | FCRL5 |
| hsa-miR-45 | FCRL5 |
| hsa-miR-48 | FCRL5 |
| hsa-miR-48 | FCRL5 |
| hsa-miR-48 | FCRL5 |
| hsa-miR-48 | FCRL5 |
| hsa-miR-49 | FCRL5 |
| hsa-miR-50 | FCRL5 |
| hsa-miR-51 | FCRL5 |
| hsa-miR-51 | FCRL5 |
| hsa-miR-54 | FCRL5 |
| hsa-miR-12 | FCRL5 |
| hsa-miR-12 | FCRL5 |
| hsa-miR-55 | FCRL5 |
| hsa-miR-58 | FCRL5 |
| hsa-miR-58 | FCRL5 |
| hsa-miR-61 | FCRL5 |
| hsa-miR-62 | FCRL5 |
| hsa-miR-62 | FCRL5 |
| hsa-miR-63 | FCRL5 |
| hsa-miR-63 | FCRL5 |
| hsa-miR-13 | FCRL5 |
| hsa-miR-64 | FCRL5 |
| hsa-miR-65 | FCRL5 |
| hsa-miR-67 | FCRL5 |
| hsa-miR-13 | FCRL5 |
| hsa-miR-13 | FCRL5 |
| hsa-miR-75 | FCRL5 |

| | |
|---|---|
| hsa-miR-7( | FCRL5 |
| hsa-miR-7( | FCRL5 |
| hsa-miR-7( | FCRL5 |
| hsa-miR-1( | FCRL5 |
| hsa-miR-14 | FCRL5 |
| hsa-miR-87 | FCRL5 |
| hsa-miR-87 | FCRL5 |
| hsa-miR-92 | FCRL5 |
| hsa-miR-93 | FCRL5 |
| hsa-miR-94 | FCRL5 |
| hsa-miR-15 | FCRL5 |
| hsa-miR-15 | FCRL5 |
| hsa-miR-15 | FCRL5 |
| hsa-miR-1( | FCRL5 |
| hsa-miR-18 | FCRL5 |
| hsa-miR-18 | FCRL5 |
| hsa-miR-1( | FCRL5 |
| hsa-miR-19 | FCRL5 |
| hsa-miR-2( | FCRL5 |
| hsa-miR-21 | FCRL5 |
| hsa-miR-21 | FCRL5 |
| hsa-miR-12 | FCRL5 |
| hsa-miR-31 | FCRL5 |
| hsa-miR-32 | FCRL5 |
| hsa-miR-33 | FCRL5 |
| hsa-miR-33 | FCRL5 |
| hsa-miR-12 | FCRL5 |
| hsa-miR-12 | FCRL5 |
| hsa-miR-37 | FCRL5 |
| hsa-miR-37 | FCRL5 |
| hsa-miR-42 | FCRL5 |
| hsa-miR-12 | FCRL5 |
| hsa-miR-43 | FCRL5 |
| hsa-miR-31 | FCRLA |
| hsa-miR-31 | FCRLA |
| hsa-miR-3( | FCRLA |
| hsa-miR-43 | FCRLA |
| hsa-miR-61 | FPR2 |
| hsa-miR-37 | FPR2 |
| hsa-miR-43 | FPR3 |
| hsa-miR-12 | FPR3 |
| hsa-miR-12 | FPR3 |
| hsa-miR-12 | FPR3 |
| hsa-miR-45 | FPR3 |
| hsa-miR-12 | FPR3 |
| hsa-miR-12 | FPR3 |
| hsa-miR-5( | FPR3 |
| hsa-miR-58 | FPR3 |

| | |
|---|---|
| hsa-miR-61 | FPR3 |
| hsa-miR-62 | FPR3 |
| hsa-miR-64 | FPR3 |
| hsa-miR-13 | FPR3 |
| hsa-miR-75 | FPR3 |
| hsa-miR-76 | FPR3 |
| hsa-miR-76 | FPR3 |
| hsa-miR-93 | FPR3 |
| hsa-miR-94 | FPR3 |
| hsa-miR-18 | FPR3 |
| hsa-miR-18 | FPR3 |
| hsa-miR-18 | FPR3 |
| hsa-miR-18 | FPR3 |
| hsa-miR-19 | FPR3 |
| hsa-miR-19 | FPR3 |
| hsa-miR-11 | FPR3 |
| hsa-miR-12 | FPR3 |
| hsa-miR-45 | FYB |
| hsa-miR-54 | FYB |
| hsa-miR-54 | FYB |
| hsa-miR-57 | FYB |
| hsa-miR-10 | FYB |
| hsa-miR-30 | FYB |
| hsa-miR-32 | FYB |
| hsa-miR-32 | FYB |
| hsa-miR-32 | FYB |
| hsa-miR-32 | FYB |
| hsa-miR-36 | FYB |
| hsa-miR-37 | FYB |
| hsa-miR-39 | FYB |
| hsa-miR-41 | FYB |
| hsa-miR-42 | FYB |
| hsa-miR-50 | GBP5 |
| hsa-miR-54 | GBP5 |
| hsa-miR-57 | GBP5 |
| hsa-miR-59 | GBP5 |
| hsa-miR-13 | GBP5 |
| hsa-miR-33 | GBP5 |
| hsa-miR-34 | GBP5 |
| hsa-miR-38 | GBP5 |
| hsa-miR-41 | GBP5 |
| hsa-miR-18 | GPNMB |
| hsa-miR-33 | GPNMB |
| hsa-miR-43 | GPNMB |
| hsa-miR-12 | HLA-DOA |
| hsa-miR-45 | HLA-DOA |
| hsa-miR-51 | HLA-DOA |
| hsa-miR-12 | HLA-DOA |

| | |
|---|---|
| hsa-miR-51 | HLA-DOA |
| hsa-miR-52 | HLA-DOA |
| hsa-miR-52 | HLA-DOA |
| hsa-miR-53 | HLA-DOA |
| hsa-miR-58 | HLA-DOA |
| hsa-miR-13 | HLA-DOA |
| hsa-miR-64 | HLA-DOA |
| hsa-miR-92 | HLA-DOA |
| hsa-miR-93 | HLA-DOA |
| hsa-miR-17 | HLA-DOA |
| hsa-miR-18 | HLA-DOA |
| hsa-miR-18 | HLA-DOA |
| hsa-miR-20 | HLA-DOA |
| hsa-miR-20 | HLA-DOA |
| hsa-miR-29 | HLA-DOA |
| hsa-miR-11 | HLA-DOA |
| hsa-miR-36 | HLA-DOA |
| hsa-miR-38 | HLA-DOA |
| hsa-miR-42 | HLA-DOA |
| hsa-miR-12 | HLA-DOA |
| hsa-miR-48 | HLA-DOA |
| hsa-miR-48 | HLA-DOA |
| hsa-miR-12 | HLA-DOA |
| hsa-miR-50 | HLA-DOA |
| hsa-miR-51 | HLA-DOA |
| hsa-miR-51 | HLA-DOA |
| hsa-miR-51 | HLA-DOA |
| hsa-miR-52 | HLA-DOA |
| hsa-miR-52 | HLA-DOA |
| hsa-miR-52 | HLA-DOA |
| hsa-miR-52 | HLA-DOA |
| hsa-miR-12 | HLA-DOA |
| hsa-miR-12 | HLA-DOA |
| hsa-miR-54 | HLA-DOA |
| hsa-miR-55 | HLA-DOA |
| hsa-miR-57 | HLA-DOA |
| hsa-miR-58 | HLA-DOA |
| hsa-miR-58 | HLA-DOA |
| hsa-miR-59 | HLA-DOA |
| hsa-miR-61 | HLA-DOA |
| hsa-miR-61 | HLA-DOA |
| hsa-miR-63 | HLA-DOA |
| hsa-miR-13 | HLA-DOA |
| hsa-miR-64 | HLA-DOA |
| hsa-miR-65 | HLA-DOA |
| hsa-miR-13 | HLA-DOA |
| hsa-miR-13 | HLA-DOA |
| hsa-miR-66 | HLA-DOA |

| | |
|---|---|
| hsa-miR-13 | HLA-DOA |
| hsa-miR-13 | HLA-DOA |
| hsa-miR-13 | HLA-DOA |
| hsa-miR-13 | HLA-DOA |
| hsa-miR-13 | HLA-DOA |
| hsa-miR-14 | HLA-DOA |
| hsa-miR-14 | HLA-DOA |
| hsa-miR-70 | HLA-DOA |
| hsa-miR-75 | HLA-DOA |
| hsa-miR-76 | HLA-DOA |
| hsa-miR-76 | HLA-DOA |
| hsa-miR-77 | HLA-DOA |
| hsa-miR-14 | HLA-DOA |
| hsa-miR-87 | HLA-DOA |
| hsa-miR-87 | HLA-DOA |
| hsa-miR-87 | HLA-DOA |
| hsa-miR-88 | HLA-DOA |
| hsa-miR-93 | HLA-DOA |
| hsa-miR-94 | HLA-DOA |
| hsa-miR-15 | HLA-DOA |
| hsa-miR-18 | HLA-DOA |
| hsa-miR-18 | HLA-DOA |
| hsa-miR-10 | HLA-DOA |
| hsa-miR-18 | HLA-DOA |
| hsa-miR-10 | HLA-DOA |
| hsa-miR-19 | HLA-DOA |
| hsa-miR-19 | HLA-DOA |
| hsa-miR-19 | HLA-DOA |
| hsa-miR-19 | HLA-DOA |
| hsa-miR-11 | HLA-DOA |
| hsa-miR-20 | HLA-DOA |
| hsa-miR-22 | HLA-DOA |
| hsa-miR-23 | HLA-DOA |
| hsa-miR-27 | HLA-DOA |
| hsa-miR-27 | HLA-DOA |
| hsa-miR-30 | HLA-DOA |
| hsa-miR-30 | HLA-DOA |
| hsa-miR-30 | HLA-DOA |
| hsa-miR-31 | HLA-DOA |
| hsa-miR-12 | HLA-DOA |
| hsa-miR-31 | HLA-DOA |
| hsa-miR-31 | HLA-DOA |
| hsa-miR-31 | HLA-DOA |
| hsa-miR-32 | HLA-DOA |
| hsa-miR-32 | HLA-DOA |
| hsa-miR-33 | HLA-DOA |
| hsa-miR-12 | HLA-DOA |
| hsa-miR-36 | HLA-DOA |

| | |
|---|---|
| hsa-miR-12 | HLA-DOA |
| hsa-miR-36 | HLA-DOA |
| hsa-miR-36 | HLA-DOA |
| hsa-miR-37 | HLA-DOA |
| hsa-miR-37 | HLA-DOA |
| hsa-miR-37 | HLA-DOA |
| hsa-miR-37 | HLA-DOA |
| hsa-miR-39 | HLA-DOA |
| hsa-miR-39 | HLA-DOA |
| hsa-miR-42 | HLA-DOA |
| hsa-miR-42 | HLA-DOA |
| hsa-miR-42 | HLA-DOA |
| hsa-miR-43 | HLA-DOA |
| hsa-miR-42 | HRH2 |
| hsa-let-7a | ICOS |
| hsa-let-7f | ICOS |
| hsa-let-7g | ICOS |
| hsa-miR-12 | ICOS |
| hsa-let-7g | ICOS |
| hsa-let-7i | ICOS |
| hsa-miR-57 | ICOS |
| hsa-miR-58 | ICOS |
| hsa-miR-60 | ICOS |
| hsa-miR-64 | ICOS |
| hsa-miR-76 | ICOS |
| hsa-miR-89 | ICOS |
| hsa-miR-92 | ICOS |
| hsa-miR-14 | ICOS |
| hsa-let-7a | ICOS |
| hsa-miR-19 | ICOS |
| hsa-miR-19 | ICOS |
| hsa-miR-24 | ICOS |
| hsa-miR-29 | ICOS |
| hsa-miR-29 | ICOS |
| hsa-miR-29 | ICOS |
| hsa-miR-29 | ICOS |
| hsa-let-7b | ICOS |
| hsa-miR-30 | ICOS |
| hsa-miR-30 | ICOS |
| hsa-miR-31 | ICOS |
| hsa-miR-31 | ICOS |
| hsa-miR-31 | ICOS |
| hsa-miR-31 | ICOS |
| hsa-miR-31 | ICOS |
| hsa-miR-32 | ICOS |
| hsa-miR-33 | ICOS |
| hsa-miR-33 | ICOS |
| hsa-miR-36 | ICOS |

| | |
|---|---|
| hsa-let-7c | ICOS |
| hsa-miR-37 | ICOS |
| hsa-miR-39 | ICOS |
| hsa-miR-12 | ICOS |
| hsa-miR-39 | ICOS |
| hsa-miR-39 | ICOS |
| hsa-let-7c | ICOS |
| hsa-miR-44 | IKZF1 |
| hsa-miR-44 | IKZF1 |
| hsa-miR-45 | IKZF1 |
| hsa-miR-45 | IKZF1 |
| hsa-miR-12 | IKZF1 |
| hsa-miR-48 | IKZF1 |
| hsa-miR-51 | IKZF1 |
| hsa-miR-51 | IKZF1 |
| hsa-miR-51 | IKZF1 |
| hsa-miR-51 | IKZF1 |
| hsa-miR-12 | IKZF1 |
| hsa-miR-51 | IKZF1 |
| hsa-miR-52 | IKZF1 |
| hsa-miR-52 | IKZF1 |
| hsa-miR-52 | IKZF1 |
| hsa-miR-52 | IKZF1 |
| hsa-miR-52 | IKZF1 |
| hsa-miR-53 | IKZF1 |
| hsa-miR-54 | IKZF1 |
| hsa-miR-12 | IKZF1 |
| hsa-miR-57 | IKZF1 |
| hsa-miR-58 | IKZF1 |
| hsa-miR-60 | IKZF1 |
| hsa-miR-61 | IKZF1 |
| hsa-miR-62 | IKZF1 |
| hsa-miR-62 | IKZF1 |
| hsa-miR-13 | IKZF1 |
| hsa-miR-13 | IKZF1 |
| hsa-miR-65 | IKZF1 |
| hsa-miR-13 | IKZF1 |
| hsa-miR-13 | IKZF1 |
| hsa-miR-13 | IKZF1 |
| hsa-miR-14 | IKZF1 |
| hsa-miR-70 | IKZF1 |
| hsa-miR-14 | IKZF1 |
| hsa-miR-76 | IKZF1 |
| hsa-miR-76 | IKZF1 |
| hsa-miR-14 | IKZF1 |
| hsa-miR-14 | IKZF1 |
| hsa-miR-87 | IKZF1 |
| hsa-miR-14 | IKZF1 |

| | |
|---|---|
| hsa-miR-89 | IKZF1 |
| hsa-miR-89 | IKZF1 |
| hsa-miR-94 | IKZF1 |
| hsa-miR-94 | IKZF1 |
| hsa-miR-96 | IKZF1 |
| hsa-miR-15 | IKZF1 |
| hsa-miR-10 | IKZF1 |
| hsa-miR-18 | IKZF1 |
| hsa-miR-18 | IKZF1 |
| hsa-miR-18 | IKZF1 |
| hsa-miR-19 | IKZF1 |
| hsa-miR-19 | IKZF1 |
| hsa-miR-19 | IKZF1 |
| hsa-miR-21 | IKZF1 |
| hsa-miR-27 | IKZF1 |
| hsa-miR-27 | IKZF1 |
| hsa-miR-28 | IKZF1 |
| hsa-miR-30 | IKZF1 |
| hsa-miR-30 | IKZF1 |
| hsa-miR-30 | IKZF1 |
| hsa-miR-30 | IKZF1 |
| hsa-miR-30 | IKZF1 |
| hsa-miR-30 | IKZF1 |
| hsa-miR-32 | IKZF1 |
| hsa-miR-32 | IKZF1 |
| hsa-miR-32 | IKZF1 |
| hsa-miR-32 | IKZF1 |
| hsa-miR-32 | IKZF1 |
| hsa-miR-32 | IKZF1 |
| hsa-miR-33 | IKZF1 |
| hsa-miR-33 | IKZF1 |
| hsa-miR-33 | IKZF1 |
| hsa-miR-34 | IKZF1 |
| hsa-miR-34 | IKZF1 |
| hsa-miR-34 | IKZF1 |
| hsa-miR-34 | IKZF1 |
| hsa-miR-12 | IKZF1 |
| hsa-miR-37 | IKZF1 |
| hsa-miR-37 | IKZF1 |
| hsa-miR-37 | IKZF1 |
| hsa-miR-12 | IKZF1 |
| hsa-miR-41 | IKZF1 |
| hsa-miR-42 | IKZF1 |
| hsa-miR-43 | IKZF1 |
| hsa-miR-12 | IKZF1 |
| hsa-miR-44 | IKZF1 |
| hsa-miR-44 | IKZF1 |
| hsa-miR-45 | IKZF1 |

| | |
|---|---|
| hsa-miR-12 | IKZF1 |
| hsa-miR-12 | IKZF1 |
| hsa-miR-12 | IKZF1 |
| hsa-miR-12 | IKZF1 |
| hsa-miR-12 | IKZF1 |
| hsa-miR-48 | IKZF1 |
| hsa-miR-48 | IKZF1 |
| hsa-miR-48 | IKZF1 |
| hsa-miR-49 | IKZF1 |
| hsa-miR-49 | IKZF1 |
| hsa-miR-49 | IKZF1 |
| hsa-miR-49 | IKZF1 |
| hsa-miR-12 | IKZF1 |
| hsa-miR-50 | IKZF1 |
| hsa-miR-12 | IKZF1 |
| hsa-miR-51 | IKZF1 |
| hsa-miR-51 | IKZF1 |
| hsa-miR-51 | IKZF1 |
| hsa-miR-51 | IKZF1 |
| hsa-miR-12 | IKZF1 |
| hsa-miR-51 | IKZF1 |
| hsa-miR-51 | IKZF1 |
| hsa-miR-52 | IKZF1 |
| hsa-miR-52 | IKZF1 |
| hsa-miR-52 | IKZF1 |
| hsa-miR-52 | IKZF1 |
| hsa-miR-52 | IKZF1 |
| hsa-miR-54 | IKZF1 |
| hsa-miR-12 | IKZF1 |
| hsa-miR-54 | IKZF1 |
| hsa-miR-54 | IKZF1 |
| hsa-miR-54 | IKZF1 |
| hsa-miR-54 | IKZF1 |
| hsa-miR-54 | IKZF1 |
| hsa-miR-55 | IKZF1 |
| hsa-miR-55 | IKZF1 |
| hsa-miR-57 | IKZF1 |
| hsa-miR-58 | IKZF1 |
| hsa-miR-58 | IKZF1 |
| hsa-miR-12 | IKZF1 |
| hsa-miR-60 | IKZF1 |
| hsa-miR-13 | IKZF1 |
| hsa-miR-61 | IKZF1 |
| hsa-miR-13 | IKZF1 |
| hsa-miR-62 | IKZF1 |
| hsa-miR-62 | IKZF1 |
| hsa-miR-62 | IKZF1 |
| hsa-miR-62 | IKZF1 |

| | |
|---|---|
| hsa-miR-63 | IKZF1 |
| hsa-miR-63 | IKZF1 |
| hsa-miR-63 | IKZF1 |
| hsa-miR-64 | IKZF1 |
| hsa-miR-64 | IKZF1 |
| hsa-miR-64 | IKZF1 |
| hsa-miR-65 | IKZF1 |
| hsa-miR-65 | IKZF1 |
| hsa-miR-66 | IKZF1 |
| hsa-miR-66 | IKZF1 |
| hsa-miR-66 | IKZF1 |
| hsa-miR-13 | IKZF1 |
| hsa-miR-13 | IKZF1 |
| hsa-miR-13 | IKZF1 |
| hsa-miR-13 | IKZF1 |
| hsa-miR-67 | IKZF1 |
| hsa-miR-13 | IKZF1 |
| hsa-miR-13 | IKZF1 |
| hsa-miR-14 | IKZF1 |
| hsa-miR-14 | IKZF1 |
| hsa-miR-14 | IKZF1 |
| hsa-miR-14 | IKZF1 |
| hsa-miR-87 | IKZF1 |
| hsa-miR-87 | IKZF1 |
| hsa-miR-88 | IKZF1 |
| hsa-miR-89 | IKZF1 |
| hsa-miR-92 | IKZF1 |
| hsa-miR-92 | IKZF1 |
| hsa-miR-92 | IKZF1 |
| hsa-miR-93 | IKZF1 |
| hsa-miR-93 | IKZF1 |
| hsa-miR-14 | IKZF1 |
| hsa-miR-94 | IKZF1 |
| hsa-miR-9- | IKZF1 |
| hsa-miR-15 | IKZF1 |
| hsa-miR-15 | IKZF1 |
| hsa-miR-17 | IKZF1 |
| hsa-miR-18 | IKZF1 |
| hsa-miR-18 | IKZF1 |
| hsa-miR-18 | IKZF1 |
| hsa-miR-18 | IKZF1 |
| hsa-miR-19 | IKZF1 |
| hsa-miR-19 | IKZF1 |
| hsa-miR-19 | IKZF1 |
| hsa-miR-19 | IKZF1 |
| hsa-miR-19 | IKZF1 |
| hsa-miR-19 | IKZF1 |
| hsa-miR-19 | IKZF1 |

| | |
|---|---|
| hsa-miR-19 | IKZF1 |
| hsa-miR-19 | IKZF1 |
| hsa-miR-19 | IKZF1 |
| hsa-miR-19 | IKZF1 |
| hsa-miR-10 | IKZF1 |
| hsa-miR-20 | IKZF1 |
| hsa-miR-11 | IKZF1 |
| hsa-miR-20 | IKZF1 |
| hsa-miR-21 | IKZF1 |
| hsa-miR-21 | IKZF1 |
| hsa-miR-21 | IKZF1 |
| hsa-miR-22 | IKZF1 |
| hsa-miR-24 | IKZF1 |
| hsa-miR-26 | IKZF1 |
| hsa-miR-29 | IKZF1 |
| hsa-miR-29 | IKZF1 |
| hsa-miR-29 | IKZF1 |
| hsa-miR-29 | IKZF1 |
| hsa-miR-30 | IKZF1 |
| hsa-miR-30 | IKZF1 |
| hsa-miR-30 | IKZF1 |
| hsa-miR-11 | IKZF1 |
| hsa-miR-31 | IKZF1 |
| hsa-miR-31 | IKZF1 |
| hsa-miR-31 | IKZF1 |
| hsa-miR-31 | IKZF1 |
| hsa-miR-31 | IKZF1 |
| hsa-miR-31 | IKZF1 |
| hsa-miR-31 | IKZF1 |
| hsa-miR-31 | IKZF1 |
| hsa-miR-31 | IKZF1 |
| hsa-miR-31 | IKZF1 |
| hsa-miR-31 | IKZF1 |
| hsa-miR-31 | IKZF1 |
| hsa-miR-31 | IKZF1 |
| hsa-miR-32 | IKZF1 |
| hsa-miR-32 | IKZF1 |
| hsa-miR-32 | IKZF1 |
| hsa-miR-33 | IKZF1 |
| hsa-miR-33 | IKZF1 |
| hsa-miR-33 | IKZF1 |
| hsa-miR-34 | IKZF1 |
| hsa-miR-34 | IKZF1 |
| hsa-miR-34 | IKZF1 |
| hsa-miR-34 | IKZF1 |
| hsa-miR-34 | IKZF1 |
| hsa-miR-12 | IKZF1 |
| hsa-miR-36 | IKZF1 |

| | |
|---|---|
| hsa-miR-36 | IKZF1 |
| hsa-miR-36 | IKZF1 |
| hsa-miR-36 | IKZF1 |
| hsa-miR-36 | IKZF1 |
| hsa-miR-36 | IKZF1 |
| hsa-miR-36 | IKZF1 |
| hsa-miR-36 | IKZF1 |
| hsa-miR-37 | IKZF1 |
| hsa-miR-37 | IKZF1 |
| hsa-miR-37 | IKZF1 |
| hsa-miR-37 | IKZF1 |
| hsa-miR-12 | IKZF1 |
| hsa-miR-39 | IKZF1 |
| hsa-miR-39 | IKZF1 |
| hsa-miR-39 | IKZF1 |
| hsa-miR-40 | IKZF1 |
| hsa-miR-42 | IKZF1 |
| hsa-miR-42 | IKZF1 |
| hsa-miR-42 | IKZF1 |
| hsa-miR-43 | IKZF1 |
| hsa-miR-43 | IKZF1 |
| hsa-miR-50 | IL2RA |
| hsa-miR-63 | IL2RA |
| hsa-miR-14 | IL2RA |
| hsa-miR-30 | IL2RA |
| hsa-miR-30 | IL2RA |
| hsa-miR-30 | IL2RA |
| hsa-miR-30 | IL2RA |
| hsa-miR-12 | IL2RA |
| hsa-miR-12 | IL2RA |
| hsa-miR-45 | IL2RA |
| hsa-miR-48 | IL2RA |
| hsa-miR-49 | IL2RA |
| hsa-miR-50 | IL2RA |
| hsa-miR-50 | IL2RA |
| hsa-miR-51 | IL2RA |
| hsa-miR-51 | IL2RA |
| hsa-miR-54 | IL2RA |
| hsa-miR-12 | IL2RA |
| hsa-miR-57 | IL2RA |
| hsa-miR-57 | IL2RA |
| hsa-miR-58 | IL2RA |
| hsa-miR-59 | IL2RA |
| hsa-miR-12 | IL2RA |
| hsa-miR-61 | IL2RA |
| hsa-miR-62 | IL2RA |
| hsa-miR-62 | IL2RA |
| hsa-miR-62 | IL2RA |

| | |
|---|---|
| hsa-miR-13 | IL2RA |
| hsa-miR-64 | IL2RA |
| hsa-miR-66 | IL2RA |
| hsa-miR-13 | IL2RA |
| hsa-miR-14 | IL2RA |
| hsa-miR-10 | IL2RA |
| hsa-miR-75 | IL2RA |
| hsa-miR-14 | IL2RA |
| hsa-miR-87 | IL2RA |
| hsa-miR-88 | IL2RA |
| hsa-miR-89 | IL2RA |
| hsa-miR-92 | IL2RA |
| hsa-miR-95 | IL2RA |
| hsa-miR-15 | IL2RA |
| hsa-miR-15 | IL2RA |
| hsa-miR-16 | IL2RA |
| hsa-miR-18 | IL2RA |
| hsa-miR-19 | IL2RA |
| hsa-miR-19 | IL2RA |
| hsa-miR-21 | IL2RA |
| hsa-miR-21 | IL2RA |
| hsa-miR-24 | IL2RA |
| hsa-miR-26 | IL2RA |
| hsa-miR-29 | IL2RA |
| hsa-miR-29 | IL2RA |
| hsa-miR-30 | IL2RA |
| hsa-miR-12 | IL2RA |
| hsa-miR-31 | IL2RA |
| hsa-miR-32 | IL2RA |
| hsa-miR-36 | IL2RA |
| hsa-miR-36 | IL2RA |
| hsa-miR-36 | IL2RA |
| hsa-miR-37 | IL2RA |
| hsa-miR-37 | IL2RA |
| hsa-miR-37 | IL2RA |
| hsa-miR-40 | IL2RA |
| hsa-miR-42 | IL2RA |
| hsa-miR-12 | IL2RA |
| hsa-miR-43 | IL2RA |
| hsa-miR-12 | IRF4 |
| hsa-miR-12 | IRF4 |
| hsa-miR-12 | IRF4 |
| hsa-miR-51 | IRF4 |
| hsa-miR-51 | IRF4 |
| hsa-miR-12 | IRF4 |
| hsa-miR-54 | IRF4 |
| hsa-miR-54 | IRF4 |
| hsa-miR-54 | IRF4 |

| | |
|---|---|
| hsa-miR-54 | IRF4 |
| hsa-miR-12 | IRF4 |
| hsa-miR-57 | IRF4 |
| hsa-miR-57 | IRF4 |
| hsa-miR-58 | IRF4 |
| hsa-miR-59 | IRF4 |
| hsa-miR-61 | IRF4 |
| hsa-miR-65 | IRF4 |
| hsa-miR-67 | IRF4 |
| hsa-miR-14 | IRF4 |
| hsa-miR-10 | IRF4 |
| hsa-miR-18 | IRF4 |
| hsa-miR-10 | IRF4 |
| hsa-miR-11 | IRF4 |
| hsa-miR-21 | IRF4 |
| hsa-miR-27 | IRF4 |
| hsa-miR-27 | IRF4 |
| hsa-miR-30 | IRF4 |
| hsa-miR-30 | IRF4 |
| hsa-miR-30 | IRF4 |
| hsa-miR-30 | IRF4 |
| hsa-miR-12 | IRF4 |
| hsa-miR-32 | IRF4 |
| hsa-miR-33 | IRF4 |
| hsa-miR-37 | IRF4 |
| hsa-miR-12 | IRF4 |
| hsa-miR-42 | IRF4 |
| hsa-miR-12 | IRF4 |
| hsa-miR-44 | IRF4 |
| hsa-miR-44 | IRF4 |
| hsa-miR-45 | IRF4 |
| hsa-miR-45 | IRF4 |
| hsa-miR-12 | IRF4 |
| hsa-let-7f | IRF4 |
| hsa-miR-48 | IRF4 |
| hsa-miR-48 | IRF4 |
| hsa-miR-49 | IRF4 |
| hsa-miR-49 | IRF4 |
| hsa-miR-49 | IRF4 |
| hsa-miR-49 | IRF4 |
| hsa-miR-12 | IRF4 |
| hsa-miR-50 | IRF4 |
| hsa-miR-50 | IRF4 |
| hsa-miR-50 | IRF4 |
| hsa-miR-50 | IRF4 |
| hsa-miR-51 | IRF4 |
| hsa-miR-51 | IRF4 |
| hsa-miR-51 | IRF4 |

| | |
|---|---|
| hsa-miR-51 | IRF4 |
| hsa-miR-51 | IRF4 |
| hsa-miR-51 | IRF4 |
| hsa-miR-12 | IRF4 |
| hsa-let-7g | IRF4 |
| hsa-miR-52 | IRF4 |
| hsa-miR-52 | IRF4 |
| hsa-miR-52 | IRF4 |
| hsa-miR-52 | IRF4 |
| hsa-miR-52 | IRF4 |
| hsa-miR-52 | IRF4 |
| hsa-miR-52 | IRF4 |
| hsa-miR-53 | IRF4 |
| hsa-miR-54 | IRF4 |
| hsa-miR-54 | IRF4 |
| hsa-miR-54 | IRF4 |
| hsa-miR-54 | IRF4 |
| hsa-miR-54 | IRF4 |
| hsa-miR-54 | IRF4 |
| hsa-miR-54 | IRF4 |
| hsa-miR-54 | IRF4 |
| hsa-miR-54 | IRF4 |
| hsa-miR-54 | IRF4 |
| hsa-miR-54 | IRF4 |
| hsa-miR-55 | IRF4 |
| hsa-miR-56 | IRF4 |
| hsa-let-7i | IRF4 |
| hsa-miR-12 | IRF4 |
| hsa-miR-57 | IRF4 |
| hsa-miR-57 | IRF4 |
| hsa-miR-57 | IRF4 |
| hsa-miR-57 | IRF4 |
| hsa-miR-58 | IRF4 |
| hsa-miR-59 | IRF4 |
| hsa-miR-59 | IRF4 |
| hsa-miR-13 | IRF4 |
| hsa-miR-61 | IRF4 |
| hsa-miR-13 | IRF4 |
| hsa-miR-62 | IRF4 |
| hsa-miR-62 | IRF4 |
| hsa-miR-64 | IRF4 |
| hsa-miR-13 | IRF4 |
| hsa-miR-64 | IRF4 |
| hsa-miR-65 | IRF4 |
| hsa-miR-65 | IRF4 |
| hsa-miR-65 | IRF4 |
| hsa-miR-65 | IRF4 |
| hsa-miR-66 | IRF4 |

| | |
|---|---|
| hsa-miR-67 | IRF4 |
| hsa-miR-13 | IRF4 |
| hsa-miR-13 | IRF4 |
| hsa-miR-14 | IRF4 |
| hsa-miR-74 | IRF4 |
| hsa-miR-7- | IRF4 |
| hsa-miR-76 | IRF4 |
| hsa-miR-76 | IRF4 |
| hsa-miR-76 | IRF4 |
| hsa-miR-76 | IRF4 |
| hsa-miR-14 | IRF4 |
| hsa-miR-87 | IRF4 |
| hsa-miR-88 | IRF4 |
| hsa-miR-89 | IRF4 |
| hsa-miR-89 | IRF4 |
| hsa-miR-92 | IRF4 |
| hsa-miR-93 | IRF4 |
| hsa-miR-94 | IRF4 |
| hsa-miR-9- | IRF4 |
| hsa-miR-98 | IRF4 |
| hsa-miR-15 | IRF4 |
| hsa-miR-15 | IRF4 |
| hsa-miR-15 | IRF4 |
| hsa-miR-16 | IRF4 |
| hsa-miR-17 | IRF4 |
| hsa-miR-18 | IRF4 |
| hsa-miR-18 | IRF4 |
| hsa-let-7a | IRF4 |
| hsa-miR-18 | IRF4 |
| hsa-miR-19 | IRF4 |
| hsa-miR-20 | IRF4 |
| hsa-miR-20 | IRF4 |
| hsa-miR-20 | IRF4 |
| hsa-miR-20 | IRF4 |
| hsa-miR-20 | IRF4 |
| hsa-miR-20 | IRF4 |
| hsa-miR-21 | IRF4 |
| hsa-miR-21 | IRF4 |
| hsa-miR-21 | IRF4 |
| hsa-miR-21 | IRF4 |
| hsa-miR-21 | IRF4 |
| hsa-miR-22 | IRF4 |
| hsa-miR-22 | IRF4 |
| hsa-miR-22 | IRF4 |
| hsa-miR-11 | IRF4 |
| hsa-miR-26 | IRF4 |
| hsa-miR-26 | IRF4 |
| hsa-miR-11 | IRF4 |

| | |
|---|---|
| hsa-miR-29 | IRF4 |
| hsa-miR-29 | IRF4 |
| hsa-miR-29 | IRF4 |
| hsa-miR-30 | IRF4 |
| hsa-miR-30 | IRF4 |
| hsa-miR-30 | IRF4 |
| hsa-miR-30 | IRF4 |
| hsa-let-7b | IRF4 |
| hsa-miR-30 | IRF4 |
| hsa-miR-30 | IRF4 |
| hsa-miR-30 | IRF4 |
| hsa-miR-30 | IRF4 |
| hsa-miR-12 | IRF4 |
| hsa-miR-31 | IRF4 |
| hsa-miR-31 | IRF4 |
| hsa-miR-31 | IRF4 |
| hsa-miR-31 | IRF4 |
| hsa-miR-31 | IRF4 |
| hsa-miR-31 | IRF4 |
| hsa-miR-12 | IRF4 |
| hsa-miR-32 | IRF4 |
| hsa-miR-32 | IRF4 |
| hsa-miR-32 | IRF4 |
| hsa-miR-32 | IRF4 |
| hsa-miR-32 | IRF4 |
| hsa-miR-33 | IRF4 |
| hsa-miR-12 | IRF4 |
| hsa-miR-34 | IRF4 |
| hsa-miR-34 | IRF4 |
| hsa-miR-36 | IRF4 |
| hsa-miR-36 | IRF4 |
| hsa-miR-12 | IRF4 |
| hsa-miR-36 | IRF4 |
| hsa-miR-36 | IRF4 |
| hsa-miR-36 | IRF4 |
| hsa-miR-36 | IRF4 |
| hsa-miR-37 | IRF4 |
| hsa-miR-37 | IRF4 |
| hsa-miR-38 | IRF4 |
| hsa-miR-39 | IRF4 |
| hsa-miR-39 | IRF4 |
| hsa-miR-12 | IRF4 |
| hsa-let-7c | IRF4 |
| hsa-miR-42 | IRF4 |
| hsa-miR-42 | IRF4 |
| hsa-miR-42 | IRF4 |
| hsa-miR-42 | IRF4 |
| hsa-miR-12 | IRF4 |

| | |
|---|---|
| hsa-miR-43 | IRF4 |
| hsa-miR-43 | IRF4 |
| hsa-miR-43 | IRF4 |
| hsa-miR-43 | KCNA3 |
| hsa-miR-43 | KCNA3 |
| hsa-miR-44 | KCNA3 |
| hsa-miR-45 | KCNA3 |
| hsa-miR-49 | KCNA3 |
| hsa-let-7g | KCNA3 |
| hsa-miR-51 | KCNA3 |
| hsa-miR-54 | KCNA3 |
| hsa-miR-54 | KCNA3 |
| hsa-miR-54 | KCNA3 |
| hsa-miR-55 | KCNA3 |
| hsa-miR-56 | KCNA3 |
| hsa-miR-57 | KCNA3 |
| hsa-miR-59 | KCNA3 |
| hsa-miR-12 | KCNA3 |
| hsa-miR-12 | KCNA3 |
| hsa-miR-62 | KCNA3 |
| hsa-miR-64 | KCNA3 |
| hsa-miR-64 | KCNA3 |
| hsa-miR-14 | KCNA3 |
| hsa-miR-80 | KCNA3 |
| hsa-miR-14 | KCNA3 |
| hsa-miR-10 | KCNA3 |
| hsa-miR-18 | KCNA3 |
| hsa-miR-20 | KCNA3 |
| hsa-miR-21 | KCNA3 |
| hsa-miR-21 | KCNA3 |
| hsa-miR-29 | KCNA3 |
| hsa-miR-30 | KCNA3 |
| hsa-miR-30 | KCNA3 |
| hsa-miR-31 | KCNA3 |
| hsa-miR-31 | KCNA3 |
| hsa-miR-31 | KCNA3 |
| hsa-miR-12 | KCNA3 |
| hsa-miR-31 | KCNA3 |
| hsa-miR-32 | KCNA3 |
| hsa-miR-32 | KCNA3 |
| hsa-miR-33 | KCNA3 |
| hsa-miR-34 | KCNA3 |
| hsa-miR-36 | KCNA3 |
| hsa-miR-12 | KCNA3 |
| hsa-miR-36 | KCNA3 |
| hsa-miR-36 | KCNA3 |
| hsa-miR-36 | KCNA3 |
| hsa-miR-38 | KCNA3 |

| | |
|---|---|
| hsa-miR-38 | KCNA3 |
| hsa-miR-39 | KCNA3 |
| hsa-miR-39 | KCNA3 |
| hsa-miR-39 | KCNA3 |
| hsa-miR-39 | KCNA3 |
| hsa-miR-42 | KCNA3 |
| hsa-miR-43 | KCNA3 |
| hsa-miR-12 | KCNJ15 |
| hsa-miR-51 | KCNJ15 |
| hsa-miR-1 | KCNJ15 |
| hsa-miR-63 | KCNJ15 |
| hsa-miR-64 | KCNJ15 |
| hsa-miR-71 | KCNJ15 |
| hsa-miR-20 | KCNJ15 |
| hsa-miR-20 | KCNJ15 |
| hsa-miR-31 | KCNJ15 |
| hsa-miR-36 | KCNJ15 |
| hsa-miR-43 | KCNJ15 |
| hsa-let-7e | KLHL6 |
| hsa-miR-45 | KLHL6 |
| hsa-miR-12 | KLHL6 |
| hsa-miR-45 | KLHL6 |
| hsa-miR-12 | KLHL6 |
| hsa-let-7f | KLHL6 |
| hsa-miR-48 | KLHL6 |
| hsa-miR-49 | KLHL6 |
| hsa-miR-50 | KLHL6 |
| hsa-miR-51 | KLHL6 |
| hsa-miR-51 | KLHL6 |
| hsa-let-7g | KLHL6 |
| hsa-miR-12 | KLHL6 |
| hsa-miR-52 | KLHL6 |
| hsa-miR-54 | KLHL6 |
| hsa-miR-54 | KLHL6 |
| hsa-miR-54 | KLHL6 |
| hsa-miR-12 | KLHL6 |
| hsa-miR-12 | KLHL6 |
| hsa-miR-55 | KLHL6 |
| hsa-let-7i | KLHL6 |
| hsa-miR-57 | KLHL6 |
| hsa-miR-57 | KLHL6 |
| hsa-miR-58 | KLHL6 |
| hsa-miR-59 | KLHL6 |
| hsa-miR-61 | KLHL6 |
| hsa-miR-61 | KLHL6 |
| hsa-miR-61 | KLHL6 |
| hsa-miR-62 | KLHL6 |
| hsa-miR-62 | KLHL6 |

| | |
|---|---|
| hsa-miR-63 | KLHL6 |
| hsa-miR-64 | KLHL6 |
| hsa-miR-66 | KLHL6 |
| hsa-miR-13 | KLHL6 |
| hsa-miR-14 | KLHL6 |
| hsa-miR-14 | KLHL6 |
| hsa-miR-76 | KLHL6 |
| hsa-miR-14 | KLHL6 |
| hsa-miR-87 | KLHL6 |
| hsa-miR-98 | KLHL6 |
| hsa-miR-18 | KLHL6 |
| hsa-let-7a | KLHL6 |
| hsa-miR-10 | KLHL6 |
| hsa-miR-19 | KLHL6 |
| hsa-miR-19 | KLHL6 |
| hsa-miR-21 | KLHL6 |
| hsa-miR-22 | KLHL6 |
| hsa-miR-22 | KLHL6 |
| hsa-let-7b | KLHL6 |
| hsa-miR-12 | KLHL6 |
| hsa-miR-12 | KLHL6 |
| hsa-miR-32 | KLHL6 |
| hsa-miR-33 | KLHL6 |
| hsa-let-7c | KLHL6 |
| hsa-miR-36 | KLHL6 |
| hsa-miR-12 | KLHL6 |
| hsa-miR-37 | KLHL6 |
| hsa-miR-37 | KLHL6 |
| hsa-miR-37 | KLHL6 |
| hsa-miR-12 | KLHL6 |
| hsa-miR-12 | KLHL6 |
| hsa-miR-12 | KLHL6 |
| hsa-miR-12 | KLHL6 |
| hsa-miR-12 | KLHL6 |
| hsa-miR-44 | KLHL6 |
| hsa-miR-12 | KLHL6 |
| hsa-miR-12 | KLHL6 |
| hsa-let-7f | KLHL6 |
| hsa-miR-12 | KLHL6 |
| hsa-miR-12 | KLHL6 |
| hsa-miR-50 | KLHL6 |
| hsa-miR-50 | KLHL6 |
| hsa-miR-50 | KLHL6 |
| hsa-miR-50 | KLHL6 |
| hsa-miR-50 | KLHL6 |
| hsa-miR-51 | KLHL6 |
| hsa-miR-51 | KLHL6 |
| hsa-miR-51 | KLHL6 |

| | |
|---|---|
| hsa-miR-51 | KLHL6 |
| hsa-miR-12 | KLHL6 |
| hsa-miR-51 | KLHL6 |
| hsa-miR-51 | KLHL6 |
| hsa-miR-51 | KLHL6 |
| hsa-miR-51 | KLHL6 |
| hsa-miR-51 | KLHL6 |
| hsa-miR-51 | KLHL6 |
| hsa-miR-51 | KLHL6 |
| hsa-miR-51 | KLHL6 |
| hsa-miR-52 | KLHL6 |
| hsa-miR-52 | KLHL6 |
| hsa-miR-52 | KLHL6 |
| hsa-miR-52 | KLHL6 |
| hsa-miR-54 | KLHL6 |
| hsa-miR-54 | KLHL6 |
| hsa-miR-12 | KLHL6 |
| hsa-miR-54 | KLHL6 |
| hsa-miR-54 | KLHL6 |
| hsa-miR-54 | KLHL6 |
| hsa-miR-54 | KLHL6 |
| hsa-miR-54 | KLHL6 |
| hsa-miR-54 | KLHL6 |
| hsa-miR-54 | KLHL6 |
| hsa-miR-56 | KLHL6 |
| hsa-miR-12 | KLHL6 |
| hsa-miR-57 | KLHL6 |
| hsa-miR-57 | KLHL6 |
| hsa-miR-57 | KLHL6 |
| hsa-miR-57 | KLHL6 |
| hsa-miR-58 | KLHL6 |
| hsa-miR-58 | KLHL6 |
| hsa-miR-58 | KLHL6 |
| hsa-miR-59 | KLHL6 |
| hsa-miR-59 | KLHL6 |
| hsa-miR-60 | KLHL6 |
| hsa-miR-12 | KLHL6 |
| hsa-miR-61 | KLHL6 |
| hsa-miR-62 | KLHL6 |
| hsa-miR-62 | KLHL6 |
| hsa-miR-63 | KLHL6 |
| hsa-miR-63 | KLHL6 |
| hsa-miR-13 | KLHL6 |
| hsa-miR-64 | KLHL6 |
| hsa-miR-64 | KLHL6 |
| hsa-miR-65 | KLHL6 |
| hsa-miR-66 | KLHL6 |
| hsa-miR-66 | KLHL6 |

| | |
|---|---|
| hsa-miR-67 | KLHL6 |
| hsa-miR-13 | KLHL6 |
| hsa-miR-13 | KLHL6 |
| hsa-miR-10 | KLHL6 |
| hsa-miR-13 | KLHL6 |
| hsa-miR-13 | KLHL6 |
| hsa-miR-13 | KLHL6 |
| hsa-miR-10 | KLHL6 |
| hsa-miR-7- | KLHL6 |
| hsa-miR-76 | KLHL6 |
| hsa-miR-76 | KLHL6 |
| hsa-miR-76 | KLHL6 |
| hsa-miR-10 | KLHL6 |
| hsa-miR-89 | KLHL6 |
| hsa-miR-89 | KLHL6 |
| hsa-miR-92 | KLHL6 |
| hsa-miR-93 | KLHL6 |
| hsa-miR-93 | KLHL6 |
| hsa-miR-15 | KLHL6 |
| hsa-miR-15 | KLHL6 |
| hsa-miR-18 | KLHL6 |
| hsa-miR-18 | KLHL6 |
| hsa-miR-18 | KLHL6 |
| hsa-miR-19 | KLHL6 |
| hsa-miR-19 | KLHL6 |
| hsa-miR-19 | KLHL6 |
| hsa-miR-19 | KLHL6 |
| hsa-miR-19 | KLHL6 |
| hsa-miR-19 | KLHL6 |
| hsa-miR-20 | KLHL6 |
| hsa-miR-20 | KLHL6 |
| hsa-miR-21 | KLHL6 |
| hsa-miR-21 | KLHL6 |
| hsa-miR-22 | KLHL6 |
| hsa-miR-22 | KLHL6 |
| hsa-miR-26 | KLHL6 |
| hsa-miR-26 | KLHL6 |
| hsa-miR-27 | KLHL6 |
| hsa-miR-29 | KLHL6 |
| hsa-miR-29 | KLHL6 |
| hsa-miR-29 | KLHL6 |
| hsa-miR-29 | KLHL6 |
| hsa-miR-11 | KLHL6 |
| hsa-miR-31 | KLHL6 |
| hsa-miR-31 | KLHL6 |
| hsa-miR-31 | KLHL6 |
| hsa-miR-31 | KLHL6 |
| hsa-miR-31 | KLHL6 |

| | |
|---|---|
| hsa-miR-31 | KLHL6 |
| hsa-miR-31 | KLHL6 |
| hsa-miR-12 | KLHL6 |
| hsa-miR-32 | KLHL6 |
| hsa-miR-32 | KLHL6 |
| hsa-miR-33 | KLHL6 |
| hsa-miR-33 | KLHL6 |
| hsa-miR-33 | KLHL6 |
| hsa-miR-34 | KLHL6 |
| hsa-miR-34 | KLHL6 |
| hsa-miR-34 | KLHL6 |
| hsa-miR-12 | KLHL6 |
| hsa-miR-36 | KLHL6 |
| hsa-miR-36 | KLHL6 |
| hsa-miR-36 | KLHL6 |
| hsa-miR-36 | KLHL6 |
| hsa-miR-36 | KLHL6 |
| hsa-miR-36 | KLHL6 |
| hsa-miR-36 | KLHL6 |
| hsa-miR-36 | KLHL6 |
| hsa-miR-36 | KLHL6 |
| hsa-miR-36 | KLHL6 |
| hsa-miR-36 | KLHL6 |
| hsa-miR-37 | KLHL6 |
| hsa-miR-37 | KLHL6 |
| hsa-miR-37 | KLHL6 |
| hsa-miR-37 | KLHL6 |
| hsa-miR-38 | KLHL6 |
| hsa-miR-38 | KLHL6 |
| hsa-miR-39 | KLHL6 |
| hsa-let-7c | KLHL6 |
| hsa-miR-12 | KLHL6 |
| hsa-miR-41 | KLHL6 |
| hsa-miR-41 | KLHL6 |
| hsa-miR-42 | KLHL6 |
| hsa-miR-12 | KLHL6 |
| hsa-miR-43 | KLHL6 |
| hsa-miR-43 | KLHL6 |
| hsa-miR-12 | LAX1 |
| hsa-miR-54 | LAX1 |
| hsa-miR-54 | LAX1 |
| hsa-miR-45 | LAX1 |
| hsa-miR-12 | LAX1 |
| hsa-miR-12 | LAX1 |
| hsa-miR-51 | LAX1 |
| hsa-miR-52 | LAX1 |
| hsa-miR-52 | LAX1 |
| hsa-miR-52 | LAX1 |

| | |
|---|---|
| hsa-miR-52 | LAX1 |
| hsa-miR-52 | LAX1 |
| hsa-miR-12 | LAX1 |
| hsa-miR-54 | LAX1 |
| hsa-miR-57 | LAX1 |
| hsa-miR-58 | LAX1 |
| hsa-miR-60 | LAX1 |
| hsa-miR-12 | LAX1 |
| hsa-miR-13 | LAX1 |
| hsa-miR-62 | LAX1 |
| hsa-miR-13 | LAX1 |
| hsa-miR-64 | LAX1 |
| hsa-miR-13 | LAX1 |
| hsa-miR-10 | LAX1 |
| hsa-miR-76 | LAX1 |
| hsa-miR-92 | LAX1 |
| hsa-miR-93 | LAX1 |
| hsa-miR-17 | LAX1 |
| hsa-miR-10 | LAX1 |
| hsa-miR-19 | LAX1 |
| hsa-miR-19 | LAX1 |
| hsa-miR-19 | LAX1 |
| hsa-miR-20 | LAX1 |
| hsa-miR-20 | LAX1 |
| hsa-miR-20 | LAX1 |
| hsa-miR-21 | LAX1 |
| hsa-miR-21 | LAX1 |
| hsa-miR-22 | LAX1 |
| hsa-miR-27 | LAX1 |
| hsa-miR-12 | LAX1 |
| hsa-miR-32 | LAX1 |
| hsa-miR-37 | LAX1 |
| hsa-miR-39 | LAX1 |
| hsa-miR-42 | LAX1 |
| hsa-miR-57 | LILRB4 |
| hsa-miR-31 | LILRB4 |
| hsa-miR-58 | LY9 |
| hsa-miR-59 | LY9 |
| hsa-miR-14 | LY9 |
| hsa-miR-15 | LY9 |
| hsa-miR-18 | LY9 |
| hsa-miR-20 | LY9 |
| hsa-miR-12 | MPEG1 |
| hsa-miR-12 | MPEG1 |
| hsa-miR-57 | MPEG1 |
| hsa-miR-58 | MPEG1 |
| hsa-miR-58 | MPEG1 |
| hsa-miR-60 | MPEG1 |

| | |
|---|---|
| hsa-miR-61 | MPEG1 |
| hsa-miR-87 | MPEG1 |
| hsa-miR-94 | MPEG1 |
| hsa-miR-94 | MPEG1 |
| hsa-miR-15 | MPEG1 |
| hsa-miR-21 | MPEG1 |
| hsa-miR-34 | MPEG1 |
| hsa-miR-44 | MPEG1 |
| hsa-miR-12 | MPEG1 |
| hsa-miR-12 | MPEG1 |
| hsa-miR-12 | MPEG1 |
| hsa-miR-12 | MPEG1 |
| hsa-miR-48 | MPEG1 |
| hsa-miR-50 | MPEG1 |
| hsa-miR-50 | MPEG1 |
| hsa-miR-51 | MPEG1 |
| hsa-miR-51 | MPEG1 |
| hsa-miR-51 | MPEG1 |
| hsa-miR-51 | MPEG1 |
| hsa-miR-12 | MPEG1 |
| hsa-miR-54 | MPEG1 |
| hsa-miR-55 | MPEG1 |
| hsa-miR-57 | MPEG1 |
| hsa-miR-59 | MPEG1 |
| hsa-miR-59 | MPEG1 |
| hsa-miR-62 | MPEG1 |
| hsa-miR-65 | MPEG1 |
| hsa-miR-13 | MPEG1 |
| hsa-miR-14 | MPEG1 |
| hsa-miR-88 | MPEG1 |
| hsa-miR-89 | MPEG1 |
| hsa-miR-10 | MPEG1 |
| hsa-miR-18 | MPEG1 |
| hsa-miR-18 | MPEG1 |
| hsa-miR-18 | MPEG1 |
| hsa-miR-19 | MPEG1 |
| hsa-miR-19 | MPEG1 |
| hsa-miR-20 | MPEG1 |
| hsa-miR-20 | MPEG1 |
| hsa-miR-20 | MPEG1 |
| hsa-miR-21 | MPEG1 |
| hsa-miR-29 | MPEG1 |
| hsa-miR-31 | MPEG1 |
| hsa-miR-12 | MPEG1 |
| hsa-miR-12 | MPEG1 |
| hsa-miR-36 | MPEG1 |
| hsa-miR-36 | MPEG1 |
| hsa-miR-37 | MPEG1 |

| | |
|---|---|
| hsa-miR-12 | MPEG1 |
| hsa-miR-38 | MPEG1 |
| hsa-miR-12 | MPEG1 |
| hsa-miR-42 | MPEG1 |
| hsa-miR-42 | MPEG1 |
| hsa-miR-43 | MPEG1 |
| hsa-miR-13 | MRC1 |
| hsa-miR-19 | MRC1 |
| hsa-miR-19 | MRC1 |
| hsa-miR-11 | MRC1 |
| hsa-miR-36 | MRC1 |
| hsa-miR-36 | MRC1 |
| hsa-miR-52 | MSR1 |
| hsa-miR-33 | MSR1 |
| hsa-miR-34 | MSR1 |
| hsa-miR-36 | MSR1 |
| hsa-miR-45 | NUGGC |
| hsa-miR-12 | NUGGC |
| hsa-miR-54 | NUGGC |
| hsa-miR-54 | NUGGC |
| hsa-miR-13 | NUGGC |
| hsa-miR-33 | NUGGC |
| hsa-miR-45 | NUGGC |
| hsa-miR-12 | NUGGC |
| hsa-miR-50 | NUGGC |
| hsa-miR-51 | NUGGC |
| hsa-miR-51 | NUGGC |
| hsa-miR-52 | NUGGC |
| hsa-miR-52 | NUGGC |
| hsa-miR-52 | NUGGC |
| hsa-miR-52 | NUGGC |
| hsa-miR-52 | NUGGC |
| hsa-miR-52 | NUGGC |
| hsa-miR-54 | NUGGC |
| hsa-miR-54 | NUGGC |
| hsa-miR-54 | NUGGC |
| hsa-miR-54 | NUGGC |
| hsa-miR-54 | NUGGC |
| hsa-miR-54 | NUGGC |
| hsa-miR-56 | NUGGC |
| hsa-miR-56 | NUGGC |
| hsa-miR-58 | NUGGC |
| hsa-miR-13 | NUGGC |
| hsa-miR-76 | NUGGC |
| hsa-miR-76 | NUGGC |
| hsa-miR-89 | NUGGC |
| hsa-miR-92 | NUGGC |
| hsa-miR-92 | NUGGC |

| | |
|---|---|
| hsa-miR-93 | NUGGC |
| hsa-miR-17 | NUGGC |
| hsa-miR-10 | NUGGC |
| hsa-miR-10 | NUGGC |
| hsa-miR-20 | NUGGC |
| hsa-miR-24 | NUGGC |
| hsa-miR-37 | NUGGC |
| hsa-let-7c | NUGGC |
| hsa-miR-12 | P2RY13 |
| hsa-miR-12 | P2RY13 |
| hsa-miR-50 | P2RY13 |
| hsa-miR-58 | P2RY13 |
| hsa-miR-62 | P2RY13 |
| hsa-miR-92 | P2RY13 |
| hsa-miR-93 | P2RY13 |
| hsa-miR-19 | P2RY13 |
| hsa-miR-19 | P2RY13 |
| hsa-miR-20 | P2RY13 |
| hsa-miR-21 | P2RY13 |
| hsa-miR-21 | P2RY13 |
| hsa-miR-31 | P2RY13 |
| hsa-miR-31 | P2RY13 |
| hsa-miR-31 | P2RY13 |
| hsa-miR-12 | PDCD1LG2 |
| hsa-miR-51 | PDCD1LG2 |
| hsa-miR-51 | PDCD1LG2 |
| hsa-miR-51 | PDCD1LG2 |
| hsa-miR-51 | PDCD1LG2 |
| hsa-miR-52 | PDCD1LG2 |
| hsa-miR-52 | PDCD1LG2 |
| hsa-miR-52 | PDCD1LG2 |
| hsa-miR-52 | PDCD1LG2 |
| hsa-miR-52 | PDCD1LG2 |
| hsa-miR-52 | PDCD1LG2 |
| hsa-miR-52 | PDCD1LG2 |
| hsa-miR-52 | PDCD1LG2 |
| hsa-miR-52 | PDCD1LG2 |
| hsa-miR-63 | PDCD1LG2 |
| hsa-miR-75 | PDCD1LG2 |
| hsa-miR-93 | PDCD1LG2 |
| hsa-miR-17 | PDCD1LG2 |
| hsa-miR-10 | PDCD1LG2 |
| hsa-miR-10 | PDCD1LG2 |
| hsa-miR-19 | PDCD1LG2 |
| hsa-miR-19 | PDCD1LG2 |
| hsa-miR-19 | PDCD1LG2 |
| hsa-miR-20 | PDCD1LG2 |
| hsa-miR-21 | PDCD1LG2 |

| | |
|---|---|
| hsa-miR-31 | PDCD1LG2 |
| hsa-miR-31 | PDCD1LG2 |
| hsa-miR-37 | PDCD1LG2 |
| hsa-miR-42 | PDCD1LG2 |
| hsa-miR-12 | PIK3CG |
| hsa-miR-12 | PIK3CG |
| hsa-miR-50 | PIK3CG |
| hsa-miR-50 | PIK3CG |
| hsa-miR-55 | PIK3CG |
| hsa-miR-55 | PIK3CG |
| hsa-miR-56 | PIK3CG |
| hsa-miR-13 | PIK3CG |
| hsa-miR-65 | PIK3CG |
| hsa-miR-67 | PIK3CG |
| hsa-miR-13 | PIK3CG |
| hsa-miR-14 | PIK3CG |
| hsa-miR-14 | PIK3CG |
| hsa-miR-89 | PIK3CG |
| hsa-miR-94 | PIK3CG |
| hsa-miR-18 | PIK3CG |
| hsa-miR-21 | PIK3CG |
| hsa-miR-12 | PIK3CG |
| hsa-miR-12 | PIK3CG |
| hsa-miR-12 | PIK3CG |
| hsa-miR-12 | PIK3CG |
| hsa-miR-12 | PIK3CG |
| hsa-miR-12 | PIK3CG |
| hsa-miR-12 | PIK3CG |
| hsa-miR-48 | PIK3CG |
| hsa-miR-49 | PIK3CG |
| hsa-miR-49 | PIK3CG |
| hsa-miR-49 | PIK3CG |
| hsa-miR-50 | PIK3CG |
| hsa-miR-50 | PIK3CG |
| hsa-miR-12 | PIK3CG |
| hsa-miR-51 | PIK3CG |
| hsa-miR-51 | PIK3CG |
| hsa-miR-51 | PIK3CG |
| hsa-miR-51 | PIK3CG |
| hsa-miR-52 | PIK3CG |
| hsa-miR-52 | PIK3CG |
| hsa-miR-54 | PIK3CG |
| hsa-miR-54 | PIK3CG |
| hsa-miR-12 | PIK3CG |
| hsa-miR-54 | PIK3CG |
| hsa-miR-54 | PIK3CG |
| hsa-miR-54 | PIK3CG |
| hsa-miR-54 | PIK3CG |

| | |
|---|---|
| hsa-miR-54 | PIK3CG |
| hsa-miR-54 | PIK3CG |
| hsa-miR-54 | PIK3CG |
| hsa-miR-54 | PIK3CG |
| hsa-miR-54 | PIK3CG |
| hsa-miR-54 | PIK3CG |
| hsa-miR-57 | PIK3CG |
| hsa-miR-58 | PIK3CG |
| hsa-miR-58 | PIK3CG |
| hsa-miR-59 | PIK3CG |
| hsa-miR-59 | PIK3CG |
| hsa-miR-12 | PIK3CG |
| hsa-miR-63 | PIK3CG |
| hsa-miR-13 | PIK3CG |
| hsa-miR-70 | PIK3CG |
| hsa-miR-14 | PIK3CG |
| hsa-miR-87 | PIK3CG |
| hsa-miR-88 | PIK3CG |
| hsa-miR-89 | PIK3CG |
| hsa-miR-92 | PIK3CG |
| hsa-miR-94 | PIK3CG |
| hsa-miR-10 | PIK3CG |
| hsa-miR-19 | PIK3CG |
| hsa-miR-20 | PIK3CG |
| hsa-miR-20 | PIK3CG |
| hsa-miR-21 | PIK3CG |
| hsa-miR-22 | PIK3CG |
| hsa-miR-22 | PIK3CG |
| hsa-miR-11 | PIK3CG |
| hsa-miR-24 | PIK3CG |
| hsa-miR-11 | PIK3CG |
| hsa-miR-28 | PIK3CG |
| hsa-miR-31 | PIK3CG |
| hsa-miR-12 | PIK3CG |
| hsa-miR-12 | PIK3CG |
| hsa-miR-31 | PIK3CG |
| hsa-miR-31 | PIK3CG |
| hsa-miR-32 | PIK3CG |
| hsa-miR-33 | PIK3CG |
| hsa-miR-12 | PIK3CG |
| hsa-miR-34 | PIK3CG |
| hsa-miR-12 | PIK3CG |
| hsa-miR-36 | PIK3CG |
| hsa-miR-36 | PIK3CG |
| hsa-miR-36 | PIK3CG |
| hsa-miR-36 | PIK3CG |
| hsa-miR-37 | PIK3CG |
| hsa-miR-38 | PIK3CG |

| | |
|---|---|
| hsa-miR-39 | PIK3CG |
| hsa-miR-39 | PIK3CG |
| hsa-miR-39 | PIK3CG |
| hsa-miR-42 | PIK3CG |
| hsa-miR-43 | PIK3CG |
| hsa-miR-43 | PIK3CG |
| hsa-miR-12 | PLA2G2D |
| hsa-miR-55 | PLA2G2D |
| hsa-miR-57 | PLA2G2D |
| hsa-miR-65 | PLA2G2D |
| hsa-miR-66 | PLA2G2D |
| hsa-miR-66 | PLA2G2D |
| hsa-miR-76 | PLA2G2D |
| hsa-miR-92 | PLA2G2D |
| hsa-miR-94 | PLA2G2D |
| hsa-miR-38 | PLA2G2D |
| hsa-miR-42 | PLA2G2D |
| hsa-miR-12 | PLA2G2D |
| hsa-miR-48 | PLA2G2D |
| hsa-miR-48 | PLA2G2D |
| hsa-miR-12 | PLA2G2D |
| hsa-miR-50 | PLA2G2D |
| hsa-miR-51 | PLA2G2D |
| hsa-miR-51 | PLA2G2D |
| hsa-miR-55 | PLA2G2D |
| hsa-miR-12 | PLA2G2D |
| hsa-miR-56 | PLA2G2D |
| hsa-miR-58 | PLA2G2D |
| hsa-miR-59 | PLA2G2D |
| hsa-miR-59 | PLA2G2D |
| hsa-miR-60 | PLA2G2D |
| hsa-miR-61 | PLA2G2D |
| hsa-miR-62 | PLA2G2D |
| hsa-miR-62 | PLA2G2D |
| hsa-miR-62 | PLA2G2D |
| hsa-miR-63 | PLA2G2D |
| hsa-miR-63 | PLA2G2D |
| hsa-miR-64 | PLA2G2D |
| hsa-miR-64 | PLA2G2D |
| hsa-miR-64 | PLA2G2D |
| hsa-miR-65 | PLA2G2D |
| hsa-miR-66 | PLA2G2D |
| hsa-miR-93 | PLA2G2D |
| hsa-miR-93 | PLA2G2D |
| hsa-miR-9- | PLA2G2D |
| hsa-miR-15 | PLA2G2D |
| hsa-miR-18 | PLA2G2D |
| hsa-miR-19 | PLA2G2D |

| | |
|---|---|
| hsa-miR-19 | PLA2G2D |
| hsa-miR-19 | PLA2G2D |
| hsa-miR-22 | PLA2G2D |
| hsa-miR-29 | PLA2G2D |
| hsa-miR-11 | PLA2G2D |
| hsa-miR-31 | PLA2G2D |
| hsa-miR-12 | PLA2G2D |
| hsa-miR-31 | PLA2G2D |
| hsa-miR-12 | PLA2G2D |
| hsa-miR-31 | PLA2G2D |
| hsa-miR-36 | PLA2G2D |
| hsa-miR-12 | PLA2G2D |
| hsa-miR-36 | PLA2G2D |
| hsa-miR-12 | PLA2G2D |
| hsa-miR-37 | PLA2G2D |
| hsa-miR-39 | PLA2G2D |
| hsa-miR-39 | PLA2G2D |
| hsa-miR-39 | PLA2G2D |
| hsa-miR-12 | PLA2G2D |
| hsa-miR-42 | PLA2G2D |
| hsa-miR-42 | PLA2G2D |
| hsa-miR-42 | PLA2G2D |
| hsa-miR-12 | PLA2G2D |
| hsa-miR-43 | PLA2G2D |
| hsa-miR-12 | PLEK |
| hsa-miR-12 | PLEK |
| hsa-miR-49 | PLEK |
| hsa-miR-51 | PLEK |
| hsa-miR-53 | PLEK |
| hsa-miR-58 | PLEK |
| hsa-miR-63 | PLEK |
| hsa-miR-64 | PLEK |
| hsa-miR-14 | PLEK |
| hsa-miR-14 | PLEK |
| hsa-miR-18 | PLEK |
| hsa-miR-18 | PLEK |
| hsa-miR-20 | PLEK |
| hsa-miR-21 | PLEK |
| hsa-miR-21 | PLEK |
| hsa-miR-11 | PLEK |
| hsa-miR-11 | PLEK |
| hsa-miR-33 | PLEK |
| hsa-miR-34 | PLEK |
| hsa-miR-12 | PLEK |
| hsa-miR-42 | PLEK |
| hsa-miR-12 | PLEK |
| hsa-miR-12 | PLEK |
| hsa-miR-44 | PLEK |

| | |
|---|---|
| hsa-miR-12 | PLEK |
| hsa-miR-12 | PLEK |
| hsa-miR-12 | PLEK |
| hsa-miR-12 | PLEK |
| hsa-miR-48 | PLEK |
| hsa-miR-48 | PLEK |
| hsa-miR-49 | PLEK |
| hsa-miR-51 | PLEK |
| hsa-miR-51 | PLEK |
| hsa-miR-52 | PLEK |
| hsa-miR-12 | PLEK |
| hsa-miR-52 | PLEK |
| hsa-miR-12 | PLEK |
| hsa-miR-54 | PLEK |
| hsa-miR-12 | PLEK |
| hsa-miR-54 | PLEK |
| hsa-miR-12 | PLEK |
| hsa-miR-55 | PLEK |
| hsa-miR-12 | PLEK |
| hsa-miR-12 | PLEK |
| hsa-miR-57 | PLEK |
| hsa-miR-57 | PLEK |
| hsa-miR-58 | PLEK |
| hsa-miR-58 | PLEK |
| hsa-miR-59 | PLEK |
| hsa-miR-12 | PLEK |
| hsa-miR-13 | PLEK |
| hsa-miR-61 | PLEK |
| hsa-miR-1 | PLEK |
| hsa-miR-61 | PLEK |
| hsa-miR-61 | PLEK |
| hsa-miR-13 | PLEK |
| hsa-miR-62 | PLEK |
| hsa-miR-63 | PLEK |
| hsa-miR-64 | PLEK |
| hsa-miR-13 | PLEK |
| hsa-miR-13 | PLEK |
| hsa-miR-70 | PLEK |
| hsa-miR-76 | PLEK |
| hsa-miR-76 | PLEK |
| hsa-miR-14 | PLEK |
| hsa-miR-14 | PLEK |
| hsa-miR-14 | PLEK |
| hsa-miR-92 | PLEK |
| hsa-miR-15 | PLEK |
| hsa-miR-17 | PLEK |
| hsa-miR-18 | PLEK |
| hsa-miR-18 | PLEK |

| | |
|---|---|
| hsa-miR-18 | PLEK |
| hsa-miR-18 | PLEK |
| hsa-miR-18 | PLEK |
| hsa-miR-20 | PLEK |
| hsa-miR-20 | PLEK |
| hsa-miR-22 | PLEK |
| hsa-miR-24 | PLEK |
| hsa-miR-27 | PLEK |
| hsa-miR-28 | PLEK |
| hsa-miR-31 | PLEK |
| hsa-miR-31 | PLEK |
| hsa-miR-31 | PLEK |
| hsa-miR-32 | PLEK |
| hsa-miR-32 | PLEK |
| hsa-miR-32 | PLEK |
| hsa-miR-32 | PLEK |
| hsa-miR-32 | PLEK |
| hsa-miR-33 | PLEK |
| hsa-miR-34 | PLEK |
| hsa-miR-34 | PLEK |
| hsa-miR-34 | PLEK |
| hsa-miR-36 | PLEK |
| hsa-miR-36 | PLEK |
| hsa-miR-12 | PLEK |
| hsa-miR-37 | PLEK |
| hsa-miR-37 | PLEK |
| hsa-miR-37 | PLEK |
| hsa-miR-37 | PLEK |
| hsa-miR-38 | PLEK |
| hsa-miR-39 | PLEK |
| hsa-miR-39 | PLEK |
| hsa-miR-39 | PLEK |
| hsa-miR-12 | PLEK |
| hsa-miR-42 | PLEK |
| hsa-miR-42 | PLEK |
| hsa-miR-42 | PLEK |
| hsa-miR-44 | PNOC |
| hsa-miR-44 | PNOC |
| hsa-miR-51 | PNOC |
| hsa-miR-59 | PNOC |
| hsa-miR-22 | PNOC |
| hsa-miR-29 | PNOC |
| hsa-miR-31 | PNOC |
| hsa-miR-34 | PNOC |
| hsa-miR-44 | PRKCB |
| hsa-miR-12 | PRKCB |
| hsa-miR-52 | PRKCB |
| hsa-miR-52 | PRKCB |

| | |
|---|---|
| hsa-miR-54 | PRKCB |
| hsa-miR-12 | PRKCB |
| hsa-miR-55 | PRKCB |
| hsa-miR-12 | PRKCB |
| hsa-miR-57 | PRKCB |
| hsa-miR-12 | PRKCB |
| hsa-miR-62 | PRKCB |
| hsa-miR-76 | PRKCB |
| hsa-miR-87 | PRKCB |
| hsa-miR-18 | PRKCB |
| hsa-miR-18 | PRKCB |
| hsa-miR-30 | PRKCB |
| hsa-miR-33 | PRKCB |
| hsa-let-7c | PRKCB |
| hsa-miR-38 | PRKCB |
| hsa-miR-43 | PRKCB |
| hsa-miR-44 | PRKCB |
| hsa-miR-12 | PRKCB |
| hsa-let-7e | PRKCB |
| hsa-miR-44 | PRKCB |
| hsa-miR-45 | PRKCB |
| hsa-miR-12 | PRKCB |
| hsa-miR-12 | PRKCB |
| hsa-miR-12 | PRKCB |
| hsa-let-7f | PRKCB |
| hsa-miR-48 | PRKCB |
| hsa-miR-49 | PRKCB |
| hsa-miR-49 | PRKCB |
| hsa-miR-49 | PRKCB |
| hsa-miR-49 | PRKCB |
| hsa-miR-51 | PRKCB |
| hsa-miR-51 | PRKCB |
| hsa-miR-51 | PRKCB |
| hsa-miR-51 | PRKCB |
| hsa-miR-51 | PRKCB |
| hsa-let-7g | PRKCB |
| hsa-miR-52 | PRKCB |
| hsa-miR-52 | PRKCB |
| hsa-miR-52 | PRKCB |
| hsa-miR-52 | PRKCB |
| hsa-miR-52 | PRKCB |
| hsa-miR-52 | PRKCB |
| hsa-miR-52 | PRKCB |
| hsa-miR-52 | PRKCB |
| hsa-miR-52 | PRKCB |
| hsa-miR-53 | PRKCB |
| hsa-miR-12 | PRKCB |
| hsa-miR-54 | PRKCB |

| | |
|---|---|
| hsa-miR-55 | PRKCB |
| hsa-miR-55 | PRKCB |
| hsa-miR-55 | PRKCB |
| hsa-miR-56 | PRKCB |
| hsa-miR-12 | PRKCB |
| hsa-miR-56 | PRKCB |
| hsa-let-7i | PRKCB |
| hsa-miR-57 | PRKCB |
| hsa-miR-57 | PRKCB |
| hsa-miR-12 | PRKCB |
| hsa-miR-58 | PRKCB |
| hsa-miR-58 | PRKCB |
| hsa-miR-12 | PRKCB |
| hsa-miR-59 | PRKCB |
| hsa-miR-60 | PRKCB |
| hsa-miR-62 | PRKCB |
| hsa-miR-62 | PRKCB |
| hsa-miR-63 | PRKCB |
| hsa-miR-64 | PRKCB |
| hsa-miR-64 | PRKCB |
| hsa-miR-64 | PRKCB |
| hsa-miR-65 | PRKCB |
| hsa-miR-65 | PRKCB |
| hsa-miR-13 | PRKCB |
| hsa-miR-13 | PRKCB |
| hsa-miR-14 | PRKCB |
| hsa-miR-14 | PRKCB |
| hsa-miR-14 | PRKCB |
| hsa-miR-7- | PRKCB |
| hsa-miR-14 | PRKCB |
| hsa-miR-14 | PRKCB |
| hsa-miR-80 | PRKCB |
| hsa-miR-14 | PRKCB |
| hsa-miR-87 | PRKCB |
| hsa-miR-87 | PRKCB |
| hsa-miR-14 | PRKCB |
| hsa-miR-89 | PRKCB |
| hsa-miR-89 | PRKCB |
| hsa-miR-89 | PRKCB |
| hsa-miR-93 | PRKCB |
| hsa-miR-93 | PRKCB |
| hsa-miR-94 | PRKCB |
| hsa-miR-98 | PRKCB |
| hsa-miR-99 | PRKCB |
| hsa-miR-15 | PRKCB |
| hsa-miR-10 | PRKCB |
| hsa-miR-18 | PRKCB |
| hsa-let-7a | PRKCB |

| | |
|---|---|
| hsa-miR-18 | PRKCB |
| hsa-miR-18 | PRKCB |
| hsa-miR-19 | PRKCB |
| hsa-miR-19 | PRKCB |
| hsa-miR-19 | PRKCB |
| hsa-miR-19 | PRKCB |
| hsa-miR-19 | PRKCB |
| hsa-miR-20 | PRKCB |
| hsa-miR-20 | PRKCB |
| hsa-miR-20 | PRKCB |
| hsa-miR-20 | PRKCB |
| hsa-miR-20 | PRKCB |
| hsa-miR-20 | PRKCB |
| hsa-miR-22 | PRKCB |
| hsa-miR-22 | PRKCB |
| hsa-miR-26 | PRKCB |
| hsa-miR-26 | PRKCB |
| hsa-miR-11 | PRKCB |
| hsa-miR-27 | PRKCB |
| hsa-miR-27 | PRKCB |
| hsa-miR-29 | PRKCB |
| hsa-miR-29 | PRKCB |
| hsa-miR-30 | PRKCB |
| hsa-miR-30 | PRKCB |
| hsa-miR-30 | PRKCB |
| hsa-miR-30 | PRKCB |
| hsa-miR-30 | PRKCB |
| hsa-let-7b | PRKCB |
| hsa-miR-30 | PRKCB |
| hsa-miR-30 | PRKCB |
| hsa-miR-30 | PRKCB |
| hsa-miR-30 | PRKCB |
| hsa-miR-30 | PRKCB |
| hsa-miR-11 | PRKCB |
| hsa-miR-30 | PRKCB |
| hsa-miR-31 | PRKCB |
| hsa-miR-12 | PRKCB |
| hsa-miR-31 | PRKCB |
| hsa-miR-31 | PRKCB |
| hsa-miR-12 | PRKCB |
| hsa-miR-12 | PRKCB |
| hsa-miR-31 | PRKCB |
| hsa-miR-32 | PRKCB |
| hsa-miR-32 | PRKCB |
| hsa-miR-32 | PRKCB |
| hsa-miR-32 | PRKCB |
| hsa-miR-12 | PRKCB |
| hsa-miR-32 | PRKCB |

| | |
|---|---|
| hsa-miR-33 | PRKCB |
| hsa-miR-33 | PRKCB |
| hsa-miR-33 | PRKCB |
| hsa-miR-34 | PRKCB |
| hsa-miR-34 | PRKCB |
| hsa-miR-34 | PRKCB |
| hsa-miR-36 | PRKCB |
| hsa-miR-36 | PRKCB |
| hsa-miR-36 | PRKCB |
| hsa-miR-36 | PRKCB |
| hsa-miR-36 | PRKCB |
| hsa-miR-36 | PRKCB |
| hsa-miR-36 | PRKCB |
| hsa-miR-37 | PRKCB |
| hsa-miR-37 | PRKCB |
| hsa-miR-37 | PRKCB |
| hsa-miR-37 | PRKCB |
| hsa-miR-38 | PRKCB |
| hsa-miR-38 | PRKCB |
| hsa-miR-39 | PRKCB |
| hsa-miR-12 | PRKCB |
| hsa-let-7c | PRKCB |
| hsa-miR-42 | PRKCB |
| hsa-miR-42 | PRKCB |
| hsa-miR-42 | PRKCB |
| hsa-let-7a | PTPRC |
| hsa-miR-46 | PTPRC |
| hsa-miR-12 | PTPRC |
| hsa-miR-49 | PTPRC |
| hsa-let-7g | PTPRC |
| hsa-miR-51 | PTPRC |
| hsa-miR-52 | PTPRC |
| hsa-miR-54 | PTPRC |
| hsa-miR-54 | PTPRC |
| hsa-miR-54 | PTPRC |
| hsa-miR-54 | PTPRC |
| hsa-miR-58 | PTPRC |
| hsa-miR-13 | PTPRC |
| hsa-miR-7- | PTPRC |
| hsa-miR-94 | PTPRC |
| hsa-miR-19 | PTPRC |
| hsa-miR-19 | PTPRC |
| hsa-miR-30 | PTPRC |
| hsa-miR-31 | PTPRC |
| hsa-miR-31 | PTPRC |
| hsa-miR-31 | PTPRC |
| hsa-miR-32 | PTPRC |
| hsa-miR-32 | PTPRC |

| | |
|---|---|
| hsa-miR-33 | PTPRC |
| hsa-miR-36 | PTPRC |
| hsa-miR-36 | PTPRC |
| hsa-miR-36 | PTPRC |
| hsa-miR-12 | SELL |
| hsa-miR-48 | SELL |
| hsa-miR-49 | SELL |
| hsa-miR-12 | SELL |
| hsa-miR-50 | SELL |
| hsa-miR-14 | SELL |
| hsa-miR-14 | SELL |
| hsa-miR-42 | SELL |
| hsa-miR-43 | SELL |
| hsa-miR-45 | SELL |
| hsa-miR-48 | SELL |
| hsa-miR-48 | SELL |
| hsa-miR-49 | SELL |
| hsa-miR-50 | SELL |
| hsa-miR-50 | SELL |
| hsa-miR-12 | SELL |
| hsa-miR-54 | SELL |
| hsa-miR-54 | SELL |
| hsa-miR-54 | SELL |
| hsa-miR-54 | SELL |
| hsa-miR-54 | SELL |
| hsa-miR-55 | SELL |
| hsa-miR-57 | SELL |
| hsa-miR-58 | SELL |
| hsa-miR-60 | SELL |
| hsa-miR-60 | SELL |
| hsa-miR-13 | SELL |
| hsa-miR-62 | SELL |
| hsa-miR-63 | SELL |
| hsa-miR-13 | SELL |
| hsa-miR-13 | SELL |
| hsa-miR-13 | SELL |
| hsa-miR-13 | SELL |
| hsa-miR-70 | SELL |
| hsa-miR-14 | SELL |
| hsa-miR-14 | SELL |
| hsa-miR-92 | SELL |
| hsa-miR-93 | SELL |
| hsa-miR-94 | SELL |
| hsa-miR-96 | SELL |
| hsa-miR-15 | SELL |
| hsa-miR-10 | SELL |
| hsa-miR-18 | SELL |
| hsa-miR-20 | SELL |

| | |
|---|---|
| hsa-miR-28 | SELL |
| hsa-miR-30 | SELL |
| hsa-miR-30 | SELL |
| hsa-miR-11 | SELL |
| hsa-miR-31 | SELL |
| hsa-miR-31 | SELL |
| hsa-miR-31 | SELL |
| hsa-miR-32 | SELL |
| hsa-miR-32 | SELL |
| hsa-miR-32 | SELL |
| hsa-miR-32 | SELL |
| hsa-miR-39 | SELL |
| hsa-miR-39 | SELL |
| hsa-miR-43 | SELL |
| hsa-miR-43 | SELL |
| hsa-miR-12 | SH2D1A |
| hsa-miR-50 | SH2D1A |
| hsa-miR-12 | SH2D1A |
| hsa-miR-58 | SH2D1A |
| hsa-miR-13 | SH2D1A |
| hsa-miR-13 | SH2D1A |
| hsa-miR-65 | SH2D1A |
| hsa-miR-18 | SH2D1A |
| hsa-miR-31 | SH2D1A |
| hsa-miR-34 | SH2D1A |
| hsa-miR-36 | SH2D1A |
| hsa-miR-12 | SH2D1A |
| hsa-miR-49 | SH2D1A |
| hsa-miR-49 | SH2D1A |
| hsa-miR-50 | SH2D1A |
| hsa-miR-50 | SH2D1A |
| hsa-miR-51 | SH2D1A |
| hsa-miR-51 | SH2D1A |
| hsa-miR-51 | SH2D1A |
| hsa-miR-51 | SH2D1A |
| hsa-miR-12 | SH2D1A |
| hsa-miR-52 | SH2D1A |
| hsa-miR-52 | SH2D1A |
| hsa-miR-53 | SH2D1A |
| hsa-miR-54 | SH2D1A |
| hsa-miR-54 | SH2D1A |
| hsa-miR-54 | SH2D1A |
| hsa-miR-54 | SH2D1A |
| hsa-miR-59 | SH2D1A |
| hsa-miR-60 | SH2D1A |
| hsa-miR-60 | SH2D1A |
| hsa-miR-64 | SH2D1A |
| hsa-miR-65 | SH2D1A |

| | |
|---|---|
| hsa-miR-66 | SH2D1A |
| hsa-miR-13 | SH2D1A |
| hsa-miR-13 | SH2D1A |
| hsa-miR-14 | SH2D1A |
| hsa-miR-14 | SH2D1A |
| hsa-miR-14 | SH2D1A |
| hsa-miR-77 | SH2D1A |
| hsa-miR-14 | SH2D1A |
| hsa-miR-88 | SH2D1A |
| hsa-miR-89 | SH2D1A |
| hsa-miR-89 | SH2D1A |
| hsa-miR-93 | SH2D1A |
| hsa-miR-94 | SH2D1A |
| hsa-miR-19 | SH2D1A |
| hsa-miR-20 | SH2D1A |
| hsa-miR-21 | SH2D1A |
| hsa-miR-21 | SH2D1A |
| hsa-miR-21 | SH2D1A |
| hsa-miR-11 | SH2D1A |
| hsa-miR-12 | SH2D1A |
| hsa-let-7c | SH2D1A |
| hsa-miR-34 | SH2D1A |
| hsa-miR-36 | SH2D1A |
| hsa-miR-12 | SH2D1A |
| hsa-miR-36 | SH2D1A |
| hsa-miR-37 | SH2D1A |
| hsa-miR-37 | SH2D1A |
| hsa-miR-37 | SH2D1A |
| hsa-miR-12 | SIRPB2 |
| hsa-miR-12 | SIRPB2 |
| hsa-miR-51 | SIRPB2 |
| hsa-miR-13 | SIRPB2 |
| hsa-miR-61 | SIRPB2 |
| hsa-miR-65 | SIRPB2 |
| hsa-miR-13 | SIRPB2 |
| hsa-miR-14 | SIRPB2 |
| hsa-miR-94 | SIRPB2 |
| hsa-miR-18 | SIRPB2 |
| hsa-miR-28 | SIRPB2 |
| hsa-miR-31 | SIRPB2 |
| hsa-miR-31 | SIRPB2 |
| hsa-miR-34 | SIRPB2 |
| hsa-miR-36 | SIRPB2 |
| hsa-miR-36 | SIRPB2 |
| hsa-miR-36 | SIRPB2 |
| hsa-miR-36 | SIRPB2 |
| hsa-miR-37 | SIRPB2 |
| hsa-miR-39 | SIRPB2 |

| | |
|---|---|
| hsa-miR-42 | SIRPB2 |
| hsa-miR-43 | SIRPB2 |
| hsa-miR-67 | SLAMF1 |
| hsa-miR-14 | SLAMF1 |
| hsa-miR-10 | SLAMF1 |
| hsa-miR-15 | SLAMF1 |
| hsa-miR-10 | SLAMF1 |
| hsa-miR-21 | SLAMF1 |
| hsa-miR-11 | SLAMF1 |
| hsa-miR-12 | SLAMF1 |
| hsa-miR-34 | SLAMF1 |
| hsa-miR-45 | SLAMF1 |
| hsa-miR-49 | SLAMF1 |
| hsa-miR-50 | SLAMF1 |
| hsa-miR-51 | SLAMF1 |
| hsa-miR-54 | SLAMF1 |
| hsa-miR-54 | SLAMF1 |
| hsa-miR-54 | SLAMF1 |
| hsa-miR-54 | SLAMF1 |
| hsa-miR-54 | SLAMF1 |
| hsa-miR-57 | SLAMF1 |
| hsa-miR-58 | SLAMF1 |
| hsa-miR-58 | SLAMF1 |
| hsa-miR-62 | SLAMF1 |
| hsa-miR-63 | SLAMF1 |
| hsa-miR-13 | SLAMF1 |
| hsa-miR-13 | SLAMF1 |
| hsa-miR-75 | SLAMF1 |
| hsa-miR-7- | SLAMF1 |
| hsa-miR-18 | SLAMF1 |
| hsa-miR-19 | SLAMF1 |
| hsa-miR-19 | SLAMF1 |
| hsa-miR-19 | SLAMF1 |
| hsa-miR-19 | SLAMF1 |
| hsa-miR-19 | SLAMF1 |
| hsa-miR-20 | SLAMF1 |
| hsa-let-7b | SLAMF1 |
| hsa-miR-26 | SLAMF1 |
| hsa-miR-27 | SLAMF1 |
| hsa-miR-27 | SLAMF1 |
| hsa-miR-28 | SLAMF1 |
| hsa-miR-29 | SLAMF1 |
| hsa-miR-29 | SLAMF1 |
| hsa-miR-30 | SLAMF1 |
| hsa-miR-30 | SLAMF1 |
| hsa-miR-30 | SLAMF1 |
| hsa-miR-31 | SLAMF1 |
| hsa-miR-12 | SLAMF1 |

| | |
|---|---|
| hsa-miR-31 | SLAMF1 |
| hsa-miR-31 | SLAMF1 |
| hsa-miR-31 | SLAMF1 |
| hsa-miR-32 | SLAMF1 |
| hsa-miR-32 | SLAMF1 |
| hsa-miR-12 | SLAMF1 |
| hsa-miR-36 | SLAMF1 |
| hsa-miR-36 | SLAMF1 |
| hsa-miR-37 | SLAMF1 |
| hsa-miR-37 | SLAMF1 |
| hsa-miR-38 | SLAMF1 |
| hsa-miR-42 | SLAMF1 |
| hsa-miR-12 | SLAMF7 |
| hsa-miR-54 | SLAMF7 |
| hsa-miR-63 | SLAMF7 |
| hsa-miR-32 | SLAMF7 |
| hsa-miR-37 | SLAMF7 |
| hsa-miR-12 | SLAMF7 |
| hsa-miR-12 | SLAMF7 |
| hsa-miR-51 | SLAMF7 |
| hsa-miR-51 | SLAMF7 |
| hsa-miR-51 | SLAMF7 |
| hsa-miR-51 | SLAMF7 |
| hsa-miR-52 | SLAMF7 |
| hsa-miR-52 | SLAMF7 |
| hsa-miR-52 | SLAMF7 |
| hsa-miR-52 | SLAMF7 |
| hsa-miR-12 | SLAMF7 |
| hsa-miR-53 | SLAMF7 |
| hsa-miR-12 | SLAMF7 |
| hsa-miR-57 | SLAMF7 |
| hsa-miR-58 | SLAMF7 |
| hsa-miR-12 | SLAMF7 |
| hsa-miR-13 | SLAMF7 |
| hsa-miR-61 | SLAMF7 |
| hsa-miR-61 | SLAMF7 |
| hsa-miR-62 | SLAMF7 |
| hsa-miR-13 | SLAMF7 |
| hsa-miR-13 | SLAMF7 |
| hsa-miR-13 | SLAMF7 |
| hsa-miR-13 | SLAMF7 |
| hsa-miR-87 | SLAMF7 |
| hsa-miR-88 | SLAMF7 |
| hsa-miR-93 | SLAMF7 |
| hsa-miR-14 | SLAMF7 |
| hsa-miR-17 | SLAMF7 |
| hsa-miR-18 | SLAMF7 |
| hsa-miR-18 | SLAMF7 |

| | |
|---|---|
| hsa-miR-18 | SLAMF7 |
| hsa-miR-19 | SLAMF7 |
| hsa-miR-10 | SLAMF7 |
| hsa-miR-19 | SLAMF7 |
| hsa-miR-20 | SLAMF7 |
| hsa-miR-21 | SLAMF7 |
| hsa-miR-21 | SLAMF7 |
| hsa-miR-28 | SLAMF7 |
| hsa-miR-31 | SLAMF7 |
| hsa-miR-31 | SLAMF7 |
| hsa-miR-12 | SLAMF7 |
| hsa-miR-32 | SLAMF7 |
| hsa-miR-33 | SLAMF7 |
| hsa-miR-34 | SLAMF7 |
| hsa-miR-36 | SLAMF7 |
| hsa-miR-36 | SLAMF7 |
| hsa-miR-37 | SLAMF7 |
| hsa-miR-43 | SLAMF7 |
| hsa-miR-43 | SLAMF7 |
| hsa-miR-54 | STAP1 |
| hsa-miR-54 | STAP1 |
| hsa-miR-15 | STAP1 |
| hsa-miR-31 | STAP1 |
| hsa-miR-32 | STAP1 |
| hsa-miR-57 | TBX21 |
| hsa-miR-76 | TBX21 |
| hsa-miR-32 | TBX21 |
| hsa-miR-33 | TBX21 |
| hsa-miR-51 | TBX21 |
| hsa-miR-14 | TBX21 |
| hsa-miR-89 | TBX21 |
| hsa-miR-20 | TBX21 |
| hsa-miR-20 | TBX21 |
| hsa-miR-20 | TBX21 |
| hsa-miR-11 | TBX21 |
| hsa-miR-28 | TBX21 |
| hsa-miR-29 | TBX21 |
| hsa-miR-29 | TBX21 |
| hsa-miR-29 | TBX21 |
| hsa-miR-29 | TBX21 |
| hsa-miR-30 | TBX21 |
| hsa-miR-36 | TBX21 |
| hsa-miR-36 | TBX21 |
| hsa-miR-12 | TBX21 |
| hsa-miR-12 | TBX21 |
| hsa-miR-44 | TFEC |
| hsa-miR-45 | TFEC |
| hsa-miR-12 | TFEC |

| | |
|---|---|
| hsa-miR-12 | TFEC |
| hsa-let-7f | TFEC |
| hsa-miR-48 | TFEC |
| hsa-miR-49 | TFEC |
| hsa-miR-49 | TFEC |
| hsa-miR-50 | TFEC |
| hsa-miR-51 | TFEC |
| hsa-miR-51 | TFEC |
| hsa-miR-51 | TFEC |
| hsa-let-7g | TFEC |
| hsa-miR-51 | TFEC |
| hsa-miR-12 | TFEC |
| hsa-miR-54 | TFEC |
| hsa-miR-54 | TFEC |
| hsa-miR-54 | TFEC |
| hsa-miR-56 | TFEC |
| hsa-miR-56 | TFEC |
| hsa-miR-56 | TFEC |
| hsa-let-7i | TFEC |
| hsa-miR-57 | TFEC |
| hsa-miR-58 | TFEC |
| hsa-miR-59 | TFEC |
| hsa-miR-61 | TFEC |
| hsa-miR-1 | TFEC |
| hsa-miR-62 | TFEC |
| hsa-miR-13 | TFEC |
| hsa-miR-13 | TFEC |
| hsa-miR-13 | TFEC |
| hsa-miR-13 | TFEC |
| hsa-miR-67 | TFEC |
| hsa-miR-13 | TFEC |
| hsa-miR-14 | TFEC |
| hsa-miR-14 | TFEC |
| hsa-miR-14 | TFEC |
| hsa-miR-80 | TFEC |
| hsa-miR-14 | TFEC |
| hsa-miR-88 | TFEC |
| hsa-miR-14 | TFEC |
| hsa-miR-93 | TFEC |
| hsa-miR-98 | TFEC |
| hsa-miR-15 | TFEC |
| hsa-miR-15 | TFEC |
| hsa-miR-15 | TFEC |
| hsa-miR-16 | TFEC |
| hsa-miR-18 | TFEC |
| hsa-let-7a | TFEC |
| hsa-miR-19 | TFEC |
| hsa-miR-19 | TFEC |

| | |
|---|---|
| hsa-miR-19 | TFEC |
| hsa-miR-19 | TFEC |
| hsa-miR-20 | TFEC |
| hsa-miR-20 | TFEC |
| hsa-miR-20 | TFEC |
| hsa-miR-21 | TFEC |
| hsa-miR-21 | TFEC |
| hsa-miR-22 | TFEC |
| hsa-miR-11 | TFEC |
| hsa-miR-29 | TFEC |
| hsa-miR-30 | TFEC |
| hsa-miR-30 | TFEC |
| hsa-miR-30 | TFEC |
| hsa-let-7b | TFEC |
| hsa-miR-30 | TFEC |
| hsa-miR-30 | TFEC |
| hsa-miR-30 | TFEC |
| hsa-miR-30 | TFEC |
| hsa-miR-31 | TFEC |
| hsa-miR-31 | TFEC |
| hsa-miR-31 | TFEC |
| hsa-miR-32 | TFEC |
| hsa-miR-33 | TFEC |
| hsa-miR-33 | TFEC |
| hsa-miR-33 | TFEC |
| hsa-miR-33 | TFEC |
| hsa-miR-36 | TFEC |
| hsa-let-7c | TFEC |
| hsa-miR-36 | TFEC |
| hsa-miR-36 | TFEC |
| hsa-miR-36 | TFEC |
| hsa-miR-36 | TFEC |
| hsa-miR-36 | TFEC |
| hsa-miR-36 | TFEC |
| hsa-miR-36 | TFEC |
| hsa-miR-36 | TFEC |
| hsa-miR-36 | TFEC |
| hsa-miR-36 | TFEC |
| hsa-miR-37 | TFEC |
| hsa-miR-37 | TFEC |
| hsa-miR-37 | TFEC |
| hsa-miR-37 | TFEC |
| hsa-miR-37 | TFEC |
| hsa-miR-38 | TFEC |
| hsa-miR-38 | TFEC |
| hsa-miR-39 | TFEC |
| hsa-let-7c | TFEC |
| hsa-miR-41 | TFEC |

| | |
|---|---|
| hsa-miR-42 | TFEC |
| hsa-miR-42 | TFEC |
| hsa-miR-42 | TFEC |
| hsa-miR-43 | TFEC |
| hsa-miR-48 | THEMIS |
| hsa-miR-54 | THEMIS |
| hsa-miR-54 | THEMIS |
| hsa-miR-54 | THEMIS |
| hsa-miR-55 | THEMIS |
| hsa-miR-13 | THEMIS |
| hsa-miR-7- | THEMIS |
| hsa-miR-15 | THEMIS |
| hsa-miR-18 | THEMIS |
| hsa-miR-18 | THEMIS |
| hsa-miR-18 | THEMIS |
| hsa-miR-29 | THEMIS |
| hsa-miR-30 | THEMIS |
| hsa-miR-31 | THEMIS |
| hsa-miR-36 | THEMIS |
| hsa-miR-12 | TIFAB |
| hsa-miR-49 | TIFAB |
| hsa-miR-54 | TIFAB |
| hsa-miR-54 | TIFAB |
| hsa-miR-54 | TIFAB |
| hsa-miR-54 | TIFAB |
| hsa-miR-54 | TIFAB |
| hsa-miR-54 | TIFAB |
| hsa-miR-54 | TIFAB |
| hsa-miR-54 | TIFAB |
| hsa-miR-55 | TIFAB |
| hsa-miR-62 | TIFAB |
| hsa-miR-14 | TIFAB |
| hsa-miR-14 | TIFAB |
| hsa-miR-15 | TIFAB |
| hsa-miR-15 | TIFAB |
| hsa-miR-15 | TIFAB |
| hsa-miR-19 | TIFAB |
| hsa-miR-45 | TIGIT |
| hsa-miR-48 | TIGIT |
| hsa-miR-50 | TIGIT |
| hsa-miR-54 | TIGIT |
| hsa-miR-61 | TIGIT |
| hsa-miR-76 | TIGIT |
| hsa-miR-94 | TIGIT |
| hsa-miR-12 | TIGIT |
| hsa-miR-12 | TIGIT |
| hsa-miR-12 | TIGIT |
| hsa-miR-48 | TIGIT |

| | |
|---|---|
| hsa-miR-51 | TIGIT |
| hsa-miR-52 | TIGIT |
| hsa-miR-52 | TIGIT |
| hsa-miR-54 | TIGIT |
| hsa-miR-54 | TIGIT |
| hsa-miR-54 | TIGIT |
| hsa-miR-54 | TIGIT |
| hsa-miR-55 | TIGIT |
| hsa-miR-58 | TIGIT |
| hsa-miR-59 | TIGIT |
| hsa-miR-60 | TIGIT |
| hsa-miR-60 | TIGIT |
| hsa-miR-62 | TIGIT |
| hsa-miR-65 | TIGIT |
| hsa-miR-65 | TIGIT |
| hsa-miR-13 | TIGIT |
| hsa-miR-14 | TIGIT |
| hsa-miR-10 | TIGIT |
| hsa-miR-19 | TIGIT |
| hsa-miR-20 | TIGIT |
| hsa-miR-20 | TIGIT |
| hsa-miR-21 | TIGIT |
| hsa-miR-22 | TIGIT |
| hsa-miR-23 | TIGIT |
| hsa-miR-11 | TIGIT |
| hsa-miR-30 | TIGIT |
| hsa-miR-30 | TIGIT |
| hsa-miR-30 | TIGIT |
| hsa-miR-31 | TIGIT |
| hsa-miR-31 | TIGIT |
| hsa-miR-33 | TIGIT |
| hsa-miR-33 | TIGIT |
| hsa-miR-33 | TIGIT |
| hsa-miR-33 | TIGIT |
| hsa-miR-34 | TIGIT |
| hsa-miR-12 | TIGIT |
| hsa-miR-36 | TIGIT |
| hsa-miR-36 | TIGIT |
| hsa-miR-36 | TIGIT |
| hsa-miR-12 | TIGIT |
| hsa-miR-40 | TIGIT |
| hsa-miR-42 | TIGIT |
| hsa-miR-55 | TLR10 |
| hsa-miR-12 | TLR7 |
| hsa-miR-51 | TLR7 |
| hsa-miR-51 | TLR7 |
| hsa-miR-51 | TLR7 |
| hsa-miR-52 | TLR7 |

| | |
|---|---|
| hsa-miR-52 | TLR7 |
| hsa-miR-52 | TLR7 |
| hsa-miR-52 | TLR7 |
| hsa-miR-52 | TLR7 |
| hsa-miR-13 | TLR7 |
| hsa-miR-65 | TLR7 |
| hsa-miR-13 | TLR7 |
| hsa-miR-9- | TLR7 |
| hsa-miR-37 | TLR7 |
| hsa-miR-43 | TLR7 |
| hsa-miR-12 | TLR7 |
| hsa-miR-44 | TLR7 |
| hsa-miR-45 | TLR7 |
| hsa-miR-48 | TLR7 |
| hsa-miR-12 | TLR7 |
| hsa-miR-51 | TLR7 |
| hsa-miR-51 | TLR7 |
| hsa-miR-52 | TLR7 |
| hsa-miR-52 | TLR7 |
| hsa-miR-52 | TLR7 |
| hsa-miR-54 | TLR7 |
| hsa-miR-54 | TLR7 |
| hsa-miR-54 | TLR7 |
| hsa-miR-12 | TLR7 |
| hsa-miR-54 | TLR7 |
| hsa-miR-54 | TLR7 |
| hsa-miR-55 | TLR7 |
| hsa-miR-56 | TLR7 |
| hsa-miR-58 | TLR7 |
| hsa-miR-60 | TLR7 |
| hsa-miR-62 | TLR7 |
| hsa-miR-64 | TLR7 |
| hsa-miR-13 | TLR7 |
| hsa-miR-13 | TLR7 |
| hsa-miR-13 | TLR7 |
| hsa-miR-13 | TLR7 |
| hsa-miR-75 | TLR7 |
| hsa-miR-77 | TLR7 |
| hsa-miR-93 | TLR7 |
| hsa-miR-94 | TLR7 |
| hsa-miR-15 | TLR7 |
| hsa-miR-17 | TLR7 |
| hsa-miR-10 | TLR7 |
| hsa-miR-19 | TLR7 |
| hsa-miR-19 | TLR7 |
| hsa-miR-20 | TLR7 |
| hsa-miR-29 | TLR7 |
| hsa-miR-29 | TLR7 |

| | |
|---|---|
| hsa-miR-30 | TLR7 |
| hsa-miR-30 | TLR7 |
| hsa-miR-30 | TLR7 |
| hsa-miR-30 | TLR7 |
| hsa-miR-31 | TLR7 |
| hsa-miR-12 | TLR7 |
| hsa-miR-34 | TLR7 |
| hsa-miR-36 | TLR7 |
| hsa-miR-37 | TLR7 |
| hsa-miR-37 | TLR7 |
| hsa-miR-42 | TLR7 |
| hsa-miR-42 | TLR7 |
| hsa-miR-12 | TLR8 |
| hsa-miR-22 | TLR8 |
| hsa-miR-31 | TLR8 |
| hsa-miR-32 | TLR8 |
| hsa-miR-39 | TLR8 |
| hsa-miR-42 | TLR8 |
| hsa-miR-43 | TLR8 |
| hsa-miR-12 | TRAT1 |
| hsa-miR-49 | TRAT1 |
| hsa-miR-56 | TRAT1 |
| hsa-miR-70 | TRAT1 |
| hsa-miR-14 | TRAT1 |
| hsa-miR-94 | TRAT1 |
| hsa-miR-94 | TRAT1 |
| hsa-miR-96 | TRAT1 |
| hsa-miR-28 | TRAT1 |
| hsa-miR-12 | ZNF831 |
| hsa-miR-44 | ZNF831 |
| hsa-miR-44 | ZNF831 |
| hsa-miR-44 | ZNF831 |
| hsa-miR-48 | ZNF831 |
| hsa-miR-48 | ZNF831 |
| hsa-miR-49 | ZNF831 |
| hsa-miR-49 | ZNF831 |
| hsa-miR-50 | ZNF831 |
| hsa-miR-50 | ZNF831 |
| hsa-miR-50 | ZNF831 |
| hsa-miR-51 | ZNF831 |
| hsa-miR-51 | ZNF831 |
| hsa-miR-51 | ZNF831 |
| hsa-miR-51 | ZNF831 |
| hsa-miR-51 | ZNF831 |
| hsa-miR-52 | ZNF831 |
| hsa-miR-52 | ZNF831 |
| hsa-miR-53 | ZNF831 |
| hsa-miR-54 | ZNF831 |

| | |
|---|---|
| hsa-miR-54 | ZNF831 |
| hsa-miR-54 | ZNF831 |
| hsa-miR-54 | ZNF831 |
| hsa-miR-54 | ZNF831 |
| hsa-miR-54 | ZNF831 |
| hsa-miR-54 | ZNF831 |
| hsa-miR-54 | ZNF831 |
| hsa-miR-54 | ZNF831 |
| hsa-miR-54 | ZNF831 |
| hsa-miR-54 | ZNF831 |
| hsa-miR-54 | ZNF831 |
| hsa-miR-54 | ZNF831 |
| hsa-miR-55 | ZNF831 |
| hsa-miR-57 | ZNF831 |
| hsa-miR-57 | ZNF831 |
| hsa-miR-57 | ZNF831 |
| hsa-miR-57 | ZNF831 |
| hsa-miR-58 | ZNF831 |
| hsa-miR-59 | ZNF831 |
| hsa-miR-63 | ZNF831 |
| hsa-miR-65 | ZNF831 |
| hsa-miR-13 | ZNF831 |
| hsa-miR-13 | ZNF831 |
| hsa-miR-14 | ZNF831 |
| hsa-miR-14 | ZNF831 |
| hsa-miR-14 | ZNF831 |
| hsa-miR-10 | ZNF831 |
| hsa-miR-76 | ZNF831 |
| hsa-miR-87 | ZNF831 |
| hsa-miR-92 | ZNF831 |
| hsa-miR-93 | ZNF831 |
| hsa-miR-15 | ZNF831 |
| hsa-miR-15 | ZNF831 |
| hsa-miR-15 | ZNF831 |
| hsa-miR-15 | ZNF831 |
| hsa-miR-18 | ZNF831 |
| hsa-miR-18 | ZNF831 |
| hsa-miR-18 | ZNF831 |
| hsa-miR-18 | ZNF831 |
| hsa-miR-18 | ZNF831 |
| hsa-miR-19 | ZNF831 |
| hsa-miR-19 | ZNF831 |
| hsa-miR-19 | ZNF831 |
| hsa-miR-19 | ZNF831 |
| hsa-miR-19 | ZNF831 |
| hsa-miR-19 | ZNF831 |
| hsa-miR-20 | ZNF831 |
| hsa-miR-20 | ZNF831 |

| | |
|---|---|
| hsa-miR-21 | ZNF831 |
| hsa-miR-21 | ZNF831 |
| hsa-miR-21 | ZNF831 |
| hsa-miR-21 | ZNF831 |
| hsa-miR-22 | ZNF831 |
| hsa-miR-25 | ZNF831 |
| hsa-miR-27 | ZNF831 |
| hsa-miR-27 | ZNF831 |
| hsa-miR-29 | ZNF831 |
| hsa-miR-30 | ZNF831 |
| hsa-miR-30 | ZNF831 |
| hsa-miR-31 | ZNF831 |
| hsa-miR-31 | ZNF831 |
| hsa-miR-31 | ZNF831 |
| hsa-miR-31 | ZNF831 |
| hsa-miR-31 | ZNF831 |
| hsa-miR-12 | ZNF831 |
| hsa-miR-31 | ZNF831 |
| hsa-miR-31 | ZNF831 |
| hsa-miR-32 | ZNF831 |
| hsa-miR-33 | ZNF831 |
| hsa-miR-33 | ZNF831 |
| hsa-miR-33 | ZNF831 |
| hsa-miR-33 | ZNF831 |
| hsa-miR-34 | ZNF831 |
| hsa-miR-34 | ZNF831 |
| hsa-miR-34 | ZNF831 |
| hsa-miR-34 | ZNF831 |
| hsa-miR-36 | ZNF831 |
| hsa-miR-36 | ZNF831 |
| hsa-miR-36 | ZNF831 |
| hsa-miR-36 | ZNF831 |
| hsa-miR-36 | ZNF831 |
| hsa-miR-36 | ZNF831 |
| hsa-miR-36 | ZNF831 |
| hsa-miR-37 | ZNF831 |
| hsa-miR-12 | ZNF831 |
| hsa-miR-39 | ZNF831 |
| hsa-miR-39 | ZNF831 |
| hsa-miR-39 | ZNF831 |
| hsa-miR-40 | ZNF831 |
| hsa-miR-42 | ZNF831 |
| hsa-miR-42 | ZNF831 |
| hsa-miR-42 | ZNF831 |
| hsa-miR-42 | ZNF831 |
| hsa-miR-42 | ZNF831 |
| hsa-miR-43 | ZNF831 |
| hsa-miR-43 | ZNF831 |

| | |
|---|---|
| hsa-miR-43 | ZNF831 |
| hsa-miR-43 | ZNF831 |
| hsa-miR-43 | ZNF831 |

\S.
