# Supplementary material for: Transcriptional profile and immune infiltration in colorectal cancer reveal the significance of inducible T‐cell costimulator as a crucial immune checkpoint molecule
Source: Cancer Med. 2024 Mar 20;13(6):e7097. doi: 10.1002/cam4.7097 (PMC10952025; doi:10.1002/cam4.7097)
Supplement: Supplementary file 10 [file CAM4-13-e7097-s010.pdf]

Supplementary file 10. 94 lncRNA-miRNA-mRNA relationship pairs including 4 lncRNAs, 41 miR

lncRNA	miRNA	Gene
LINC00861	hsa-miR-12	IKZF1
RP11-750H9	hsa-miR-12	FCGR2B
RP11-750H9	hsa-miR-12	SLAMF7
RP11-750H9	hsa-miR-12	PLEK
LINC00861	hsa-miR-13	KLHL6
LINC00861	hsa-miR-13	IRF4
LINC00861	hsa-miR-13	SLAMF1
LINC00861	hsa-miR-13	FCRL5
LINC00861	hsa-miR-14	PRKCB
LINC00861	hsa-miR-14	IKZF1
LINC00861	hsa-miR-14	PRKCB
RP11-121A8	hsa-miR-18	IKZF1
RP11-121A8	hsa-miR-18	CYBB
RP11-121A8	hsa-miR-18	KLHL6
RP11-750H9	hsa-miR-18	IKZF1
RP11-750H9	hsa-miR-18	KLHL6
RP11-750H9	hsa-miR-18	CYBB
RP11-750H9	hsa-miR-18	HLA-DOA
RP11-750H9	hsa-miR-18	CD209
RP11-750H9	hsa-miR-18	SLAMF1
RP11-121A8	hsa-miR-22	CD84
RP11-750H9	hsa-miR-22	CD80
LINC00861	hsa-miR-31	SLAMF1
LINC00861	hsa-miR-31	KCNA3
LINC00861	hsa-miR-31	P2RY13
LINC00861	hsa-miR-31	PIK3CG
LINC00861	hsa-miR-31	SLAMF1
LINC00861	hsa-miR-31	IKZF1
LINC00861	hsa-miR-31	PTPRC
LINC00861	hsa-miR-31	IRF4
LINC00861	hsa-miR-31	IKZF1
LINC00861	hsa-miR-32	KCNA3
CTB-114C7.	hsa-miR-36	CLEC7A
CTB-114C7.	hsa-miR-36	PLEK
LINC00861	hsa-miR-37	IKZF1
LINC00861	hsa-miR-37	PLA2G2D
LINC00861	hsa-miR-37	IKZF1
LINC00861	hsa-miR-37	KLHL6
LINC00861	hsa-miR-37	FCRL5
RP11-750H9	hsa-miR-39	PLEK
RP11-750H9	hsa-miR-39	IKZF1
LINC00861	hsa-miR-39	PLA2G2D
RP11-750H9	hsa-miR-39	PLEK
RP11-121A8	hsa-miR-42	CYBB
RP11-121A8	hsa-miR-43	CD84
RP11-750H9	hsa-miR-43	CD80

RP11-750H9	hsa-miR-43	KLHL6
RP11-121A8	hsa-miR-48	IKZF1
RP11-121A8	hsa-miR-50	FPR3
RP11-121A8	hsa-miR-50	CD84
RP11-121A8	hsa-miR-50	TFEC
RP11-121A8	hsa-miR-50	P2RY13
LINC00861	hsa-miR-50	KLHL6
LINC00861	hsa-miR-50	ZNF831
LINC00861	hsa-miR-50	PIK3CG
LINC00861	hsa-miR-51	PIK3CG
LINC00861	hsa-miR-51	KLHL6
RP11-121A8	hsa-miR-52	IKZF1
RP11-121A8	hsa-miR-54	CSF2RB
RP11-121A8	hsa-miR-54	PTPRC
RP11-121A8	hsa-miR-54	PLEK
LINC00861	hsa-miR-57	KLHL6
LINC00861	hsa-miR-57	IRF4
LINC00861	hsa-miR-57	IKZF1
LINC00861	hsa-miR-57	PRKCB
LINC00861	hsa-miR-57	ZNF831
RP11-121A8	hsa-miR-58	C3AR1
LINC00861	hsa-miR-58	CD28
LINC00861	hsa-miR-58	SLAMF1
LINC00861	hsa-miR-58	CCR7
LINC00861	hsa-miR-58	KLHL6
LINC00861	hsa-miR-58	FCRL5
LINC00861	hsa-miR-58	ICOS
LINC00861	hsa-miR-58	P2RY13
LINC00861	hsa-miR-59	PRKCB
LINC00861	hsa-miR-59	CD28
LINC00861	hsa-miR-59	IRF4
LINC00861	hsa-miR-59	KLHL6
LINC00861	hsa-miR-59	ABCD2
LINC00861	hsa-miR-59	IRF4
LINC00861	hsa-miR-59	LY9
LINC00861	hsa-miR-59	MPEG1
LINC00861	hsa-miR-59	PIK3CG
RP11-121A8	hsa-miR-62	KLHL6
RP11-750H9	hsa-miR-76	CD209
RP11-750H9	hsa-miR-76	PLEK
RP11-750H9	hsa-miR-76	KLHL6
RP11-750H9	hsa-miR-76	FPR3
LINC00861	hsa-miR-94	IKZF1
LINC00861	hsa-miR-94	PLA2G2D
LINC00861	hsa-miR-94	MPEG1
LINC00861	hsa-miR-94	PIK3CG
RP11-121A8	hsa-miR-94	CYBB
RP11-121A8	hsa-miR-94	PTPRC

ːNAs, and 31 mRNAs for CD8+ ceRNA network.
