# Supplementary figures and images for: Transcriptional profile and immune infiltration in colorectal cancer reveal the significance of inducible T‐cell costimulator as a crucial immune checkpoint molecule
Source: Cancer Med. 2024 Mar 20;13(6):e7097. doi: 10.1002/cam4.7097 (PMC10952025; doi:10.1002/cam4.7097)

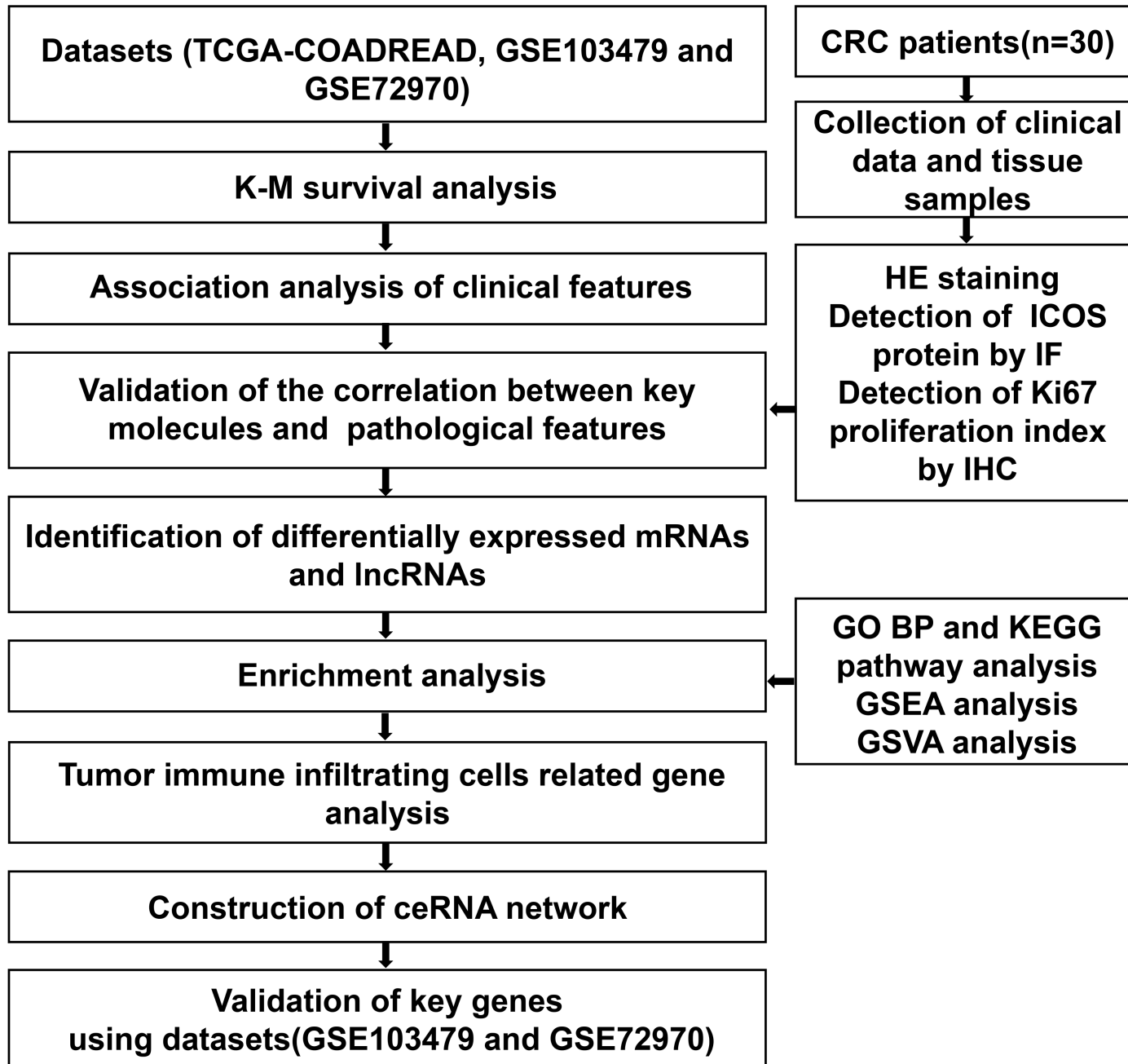

Supplement: Supplementary file 11 — Figure S1. [file CAM4-13-e7097-s013.pdf]
