# Supplementary material for: Transcriptional profile and immune infiltration in colorectal cancer reveal the significance of inducible T‐cell costimulator as a crucial immune checkpoint molecule
Source: Cancer Med. 2024 Mar 20;13(6):e7097. doi: 10.1002/cam4.7097 (PMC10952025; doi:10.1002/cam4.7097)
Supplement: Supplementary file 12 — Figure S2. [file CAM4-13-e7097-s001.pdf]

OS survival curve in TCGA cohort (cutpoint as cutoff)

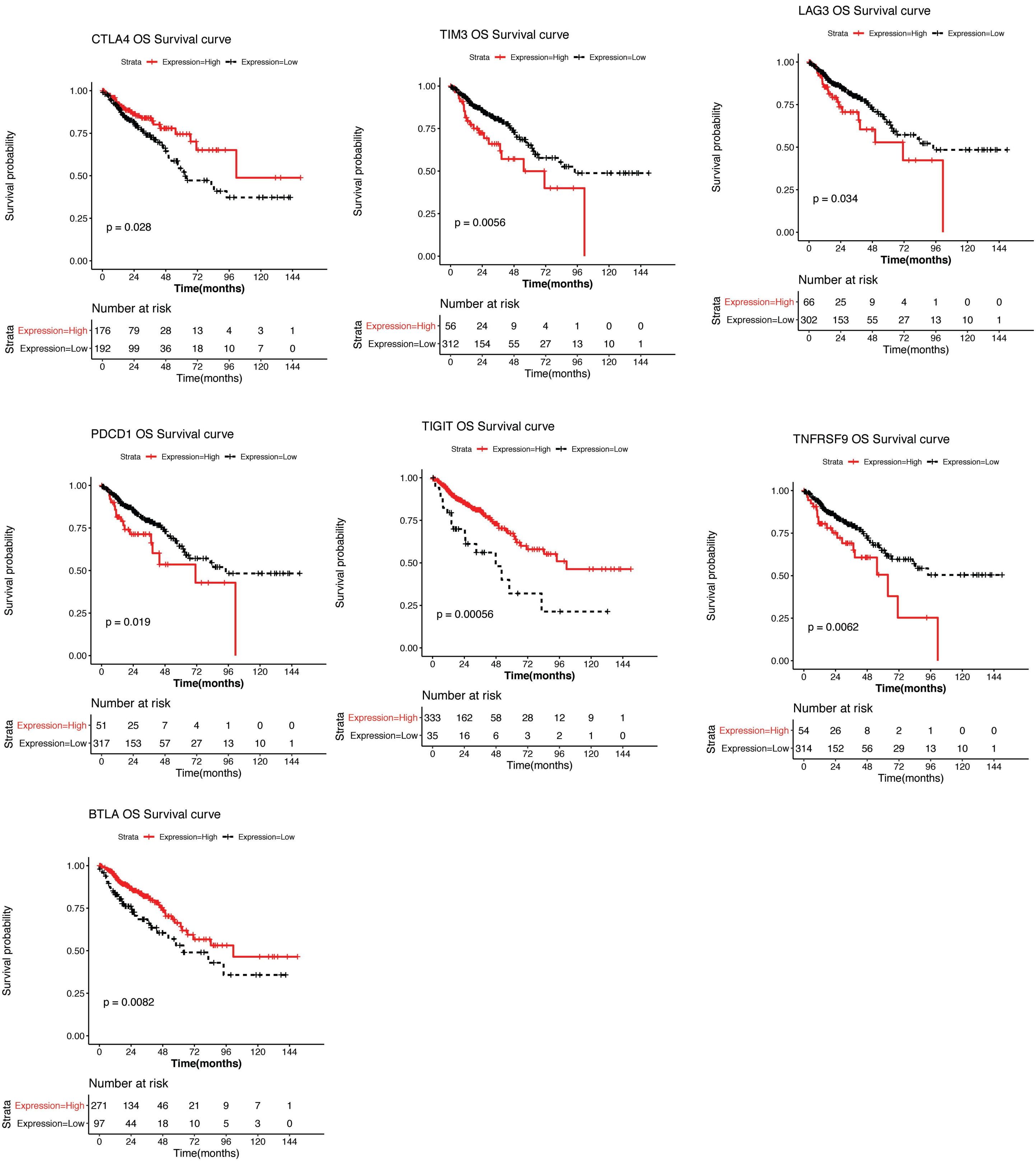

OS survival curve in GSE103479 (cutpoint as cutoff)

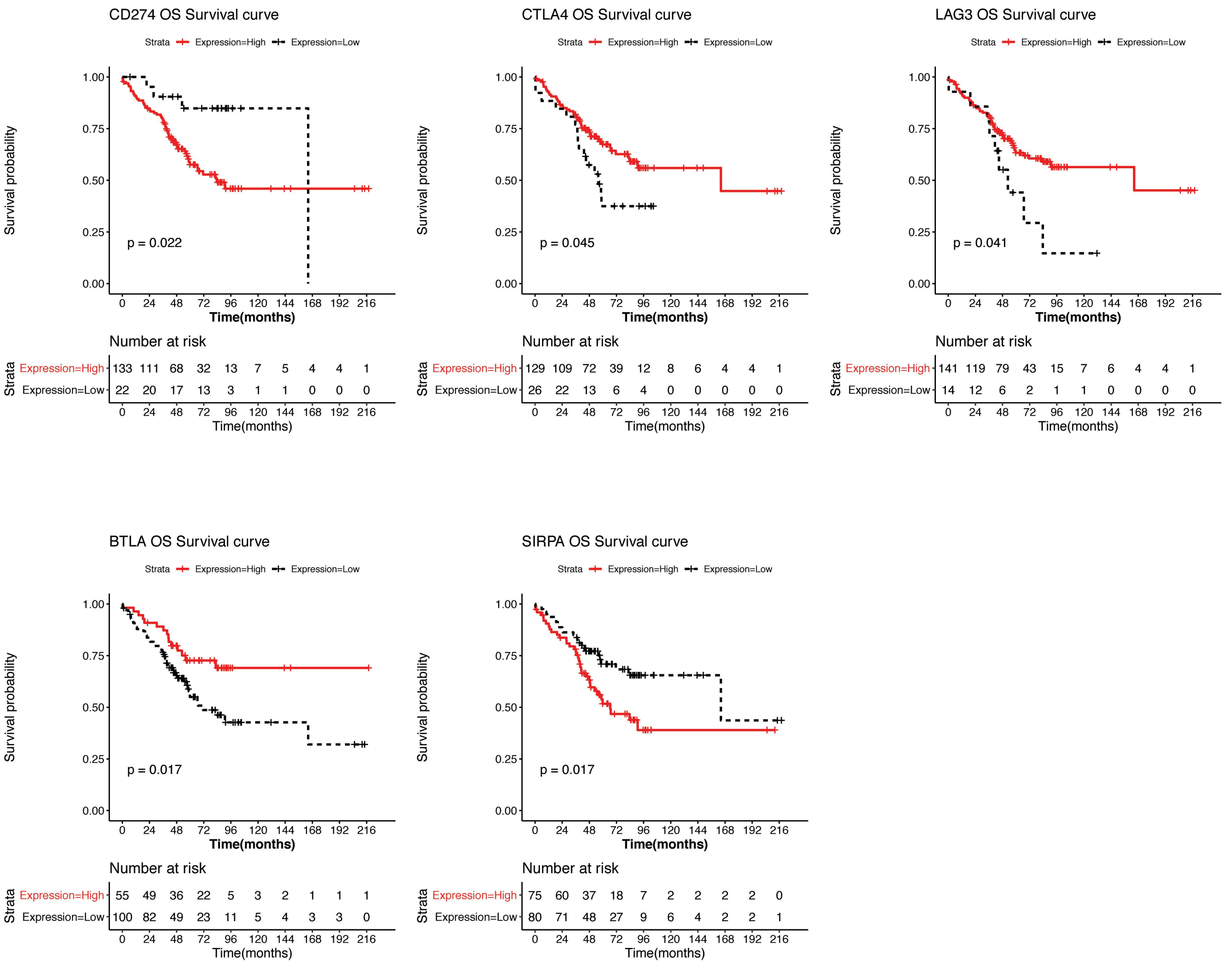

OS survival curve in GSE103479 (median as cutoff)

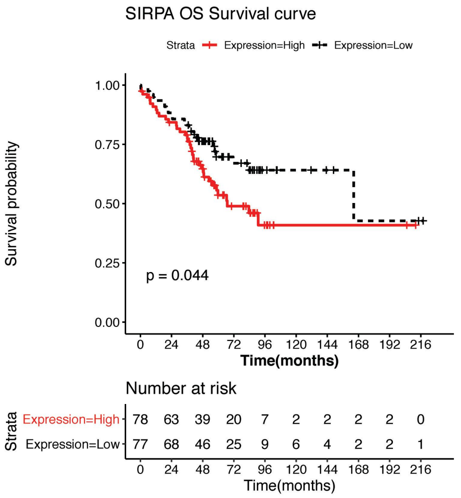

PFS survival curve in GSE103479 (cutpoint as cutoff)

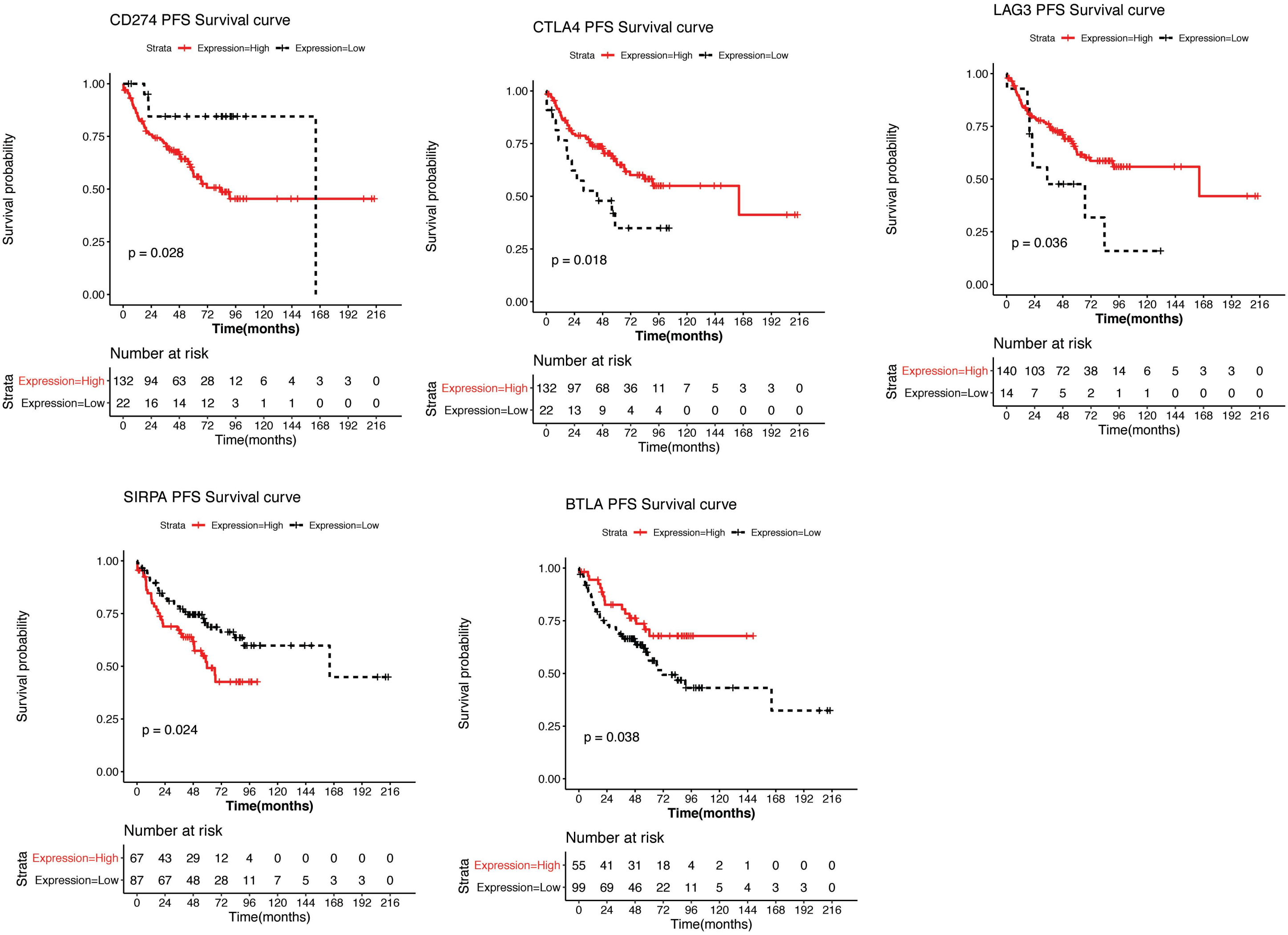

OS survival curve in GSE72970 (cutpoint as cutoff)

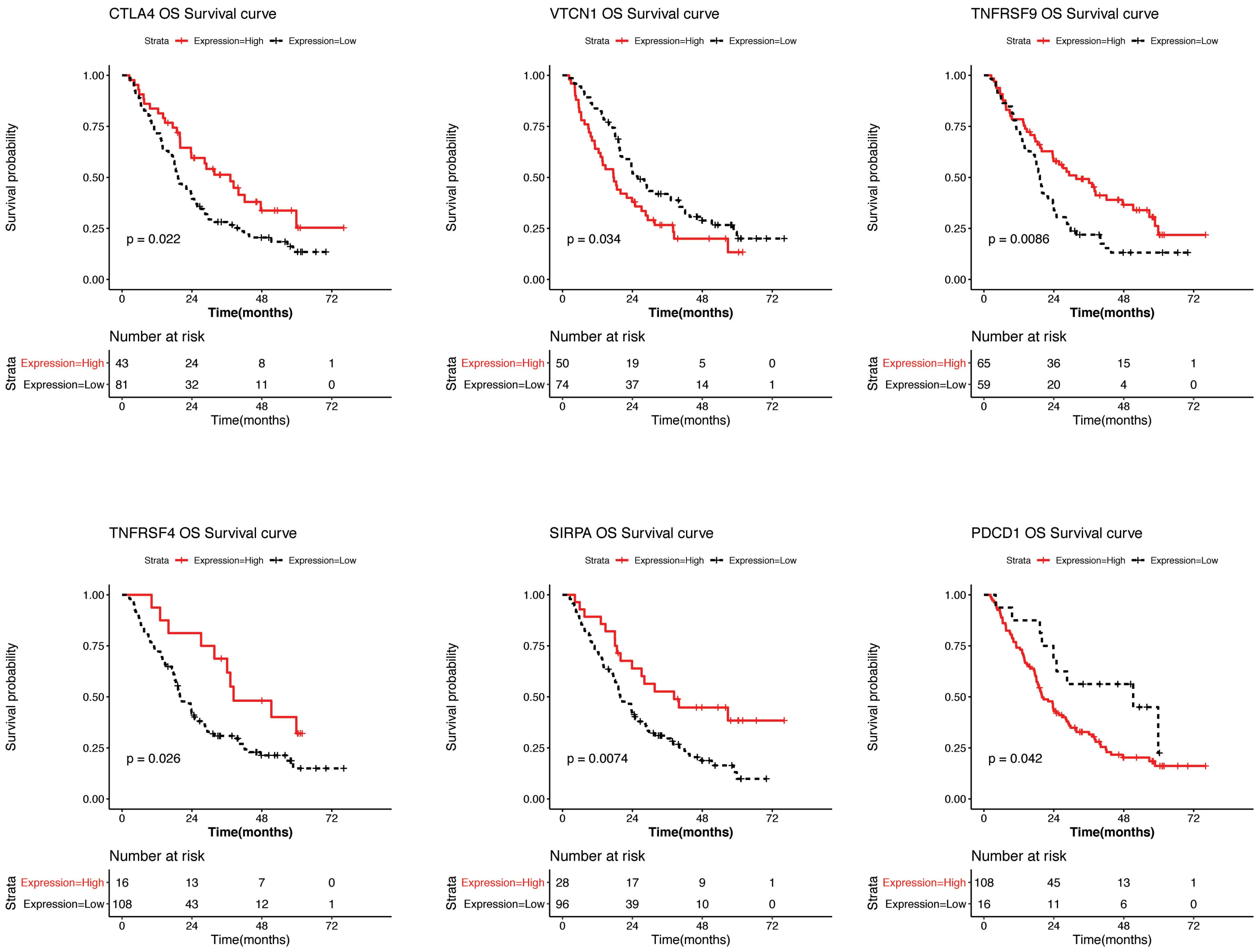

OS survival curve in GSE72970 (median as cutoff)

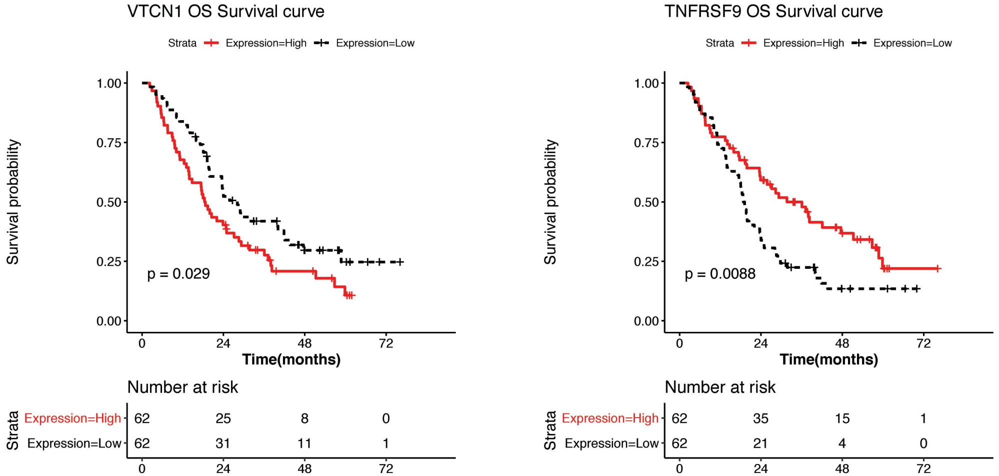

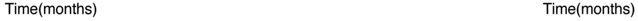

PFS survival curve in GSE72970 (cutpoint as cutoff)

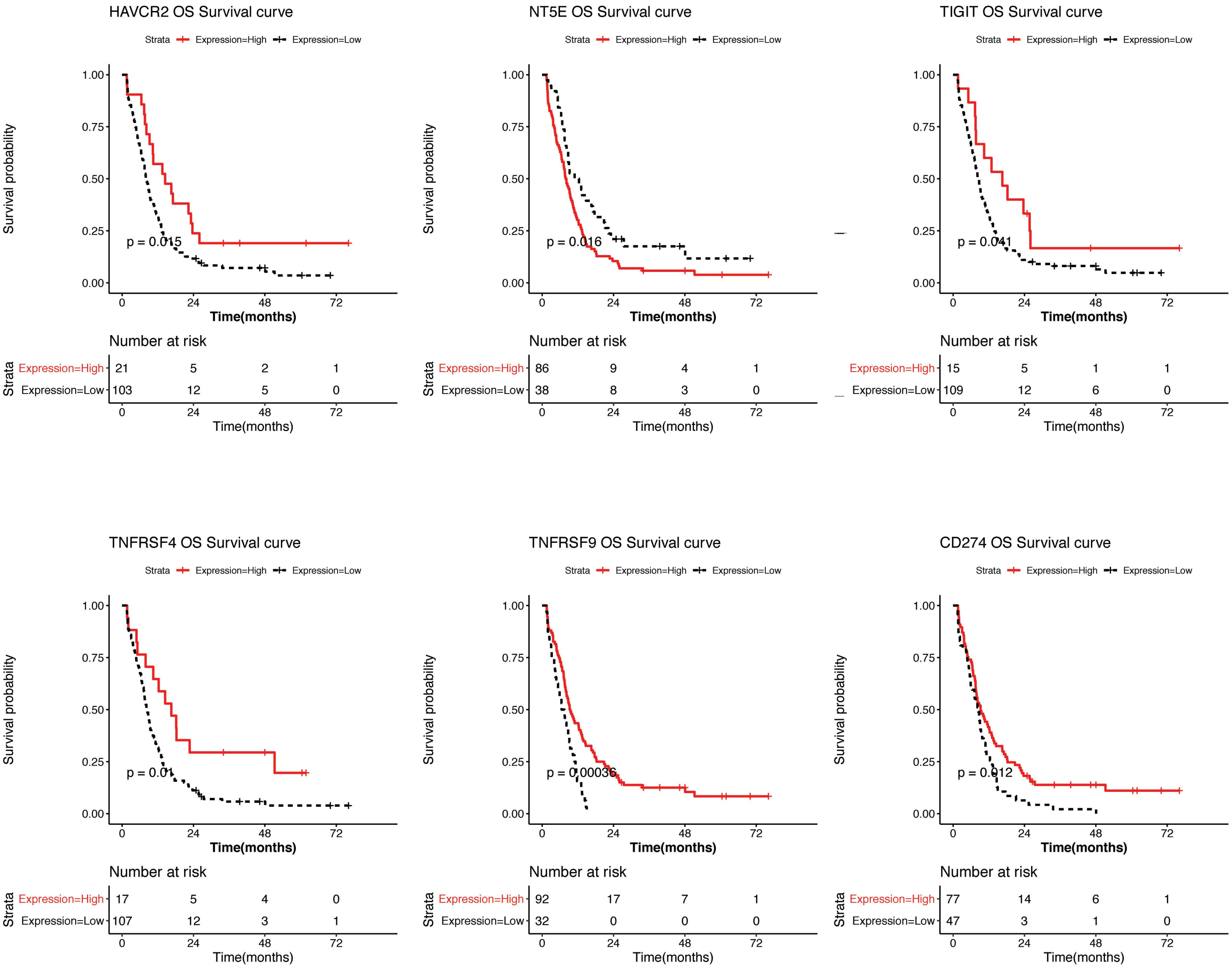

PFS survival curve in GSE72970 (median as cutoff)

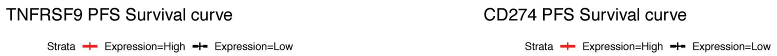

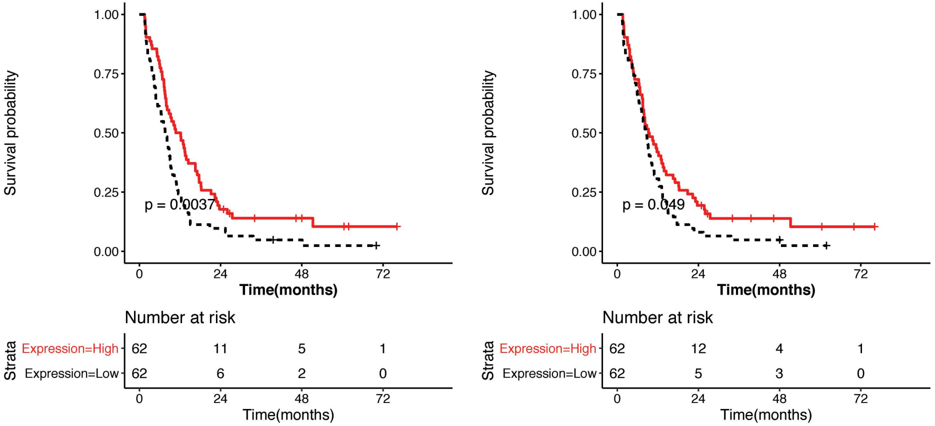

Supplementary figure 1. K-M plots of 5-year overall survival (OS) and progression free survival (PFS) for other immune checkpoints. K-M plots using group classified with cut point value of CTLA4's expressions for OS in TCGA-COADREAD, K-M plots using group classified with cut point value of TIM3's expressions for OS in TCGA-COADREAD, K-M plots using group classified with cut point value of LAG3's expressions for OS in TCGA-COADREAD, K-M plots using group classified with cut point value of PD1's expressions for OS in TCGA-COADREAD, K-M plots using group classified with cut point value of TIGIT's expressions for OS in TCGA-COADREAD, K-M plots using group classified with cut point value of TNFRSF9's expressions for OS in TCGA-COADREAD, K-M plots using group classified with cut point value of BTLA's expressions for OS in TCGA-COADREAD, K-M plots using group classified with cut point value of PDL1's expressions for OS in GSE103479, K-M plots using group classified with cut point value of CTLA4's expressions for OS in GSE103479, K-M plots using group classified with cut point value of LAG3's expressions for OS in GSE103479, K-M plots using group classified with cut point value of BTLA's expressions for OS in GSE103479, K-M plots using group classified with cut point value of SIRPA's expressions for OS in GSE103479, K-M plots using group classified with median value of SIRPA's expressions for OS in GSE103479, K-M plots using group classified with cut point value of PDL1's expressions for PFS in GSE103479, K-M plots using group classified with cut point value of CTLA4's expressions for PFS in GSE103479, K-M plots using group classified with cut point value of LAG3's expressions for PFS in GSE103479, K-M plots using group classified with cut point value of SIRPA's expressions for PFS in GSE103479, K-M plots using group classified with cut point value of BTLA's expressions for PFS in GSE103479, K-M plots using group classified with cut point value of CTLA4's expressions for OS in GSE72970, K-M plots using group classified with cut point value of VTCN1's expressions for OS in GSE72970, K-M plots using group classified with cut point value of TNFRSF9's expressions for OS in GSE72970, K-M plots using group classified with cut point value of TNFRSF4's expressions for OS in GSE72970, K-M plots using group classified with cut point value of SIRPA's expressions for OS in GSE72970, K-M plots using group classified with cut point value of PD1's expressions for OS in GSE72970, K-M plots using group classified with median value of VTCN1's expressions for OS in GSE72970, K-M plots using group classified with median value of TNFRSF9's expressions for OS in GSE72970, K-M plots using group classified with cut point value of TIM3's expressions for PFS in GSE72970, K-M plots using group classified with cut point value of CD73's expressions for PFS in GSE72970, K-M plots using group classified with cut point value of TIGIT's expressions for PFS in GSE72970, K-M plots using group classified with cut point value of TNFRSF4's expressions for PFS in GSE72970, K-M plots using group classified with cut point value of TNFRSF9's expressions for PFS in GSE72970, K-M plots using group classified with cut point value of PDL1's expressions for PFS in GSE72970, K-M plots using group classified with median value of TNFRSF9's expressions for PFS in GSE72970, K-M plots using group classified with median value of PDL1's expressions for PFS in GSE72970 (order from top left to bottom right).
