# Supplementary material for: Transcriptional profile and immune infiltration in colorectal cancer reveal the significance of inducible T‐cell costimulator as a crucial immune checkpoint molecule
Source: Cancer Med. 2024 Mar 20;13(6):e7097. doi: 10.1002/cam4.7097 (PMC10952025; doi:10.1002/cam4.7097)
Supplement: Supplementary file 13 — Figure S3. [file CAM4-13-e7097-s006.pdf]

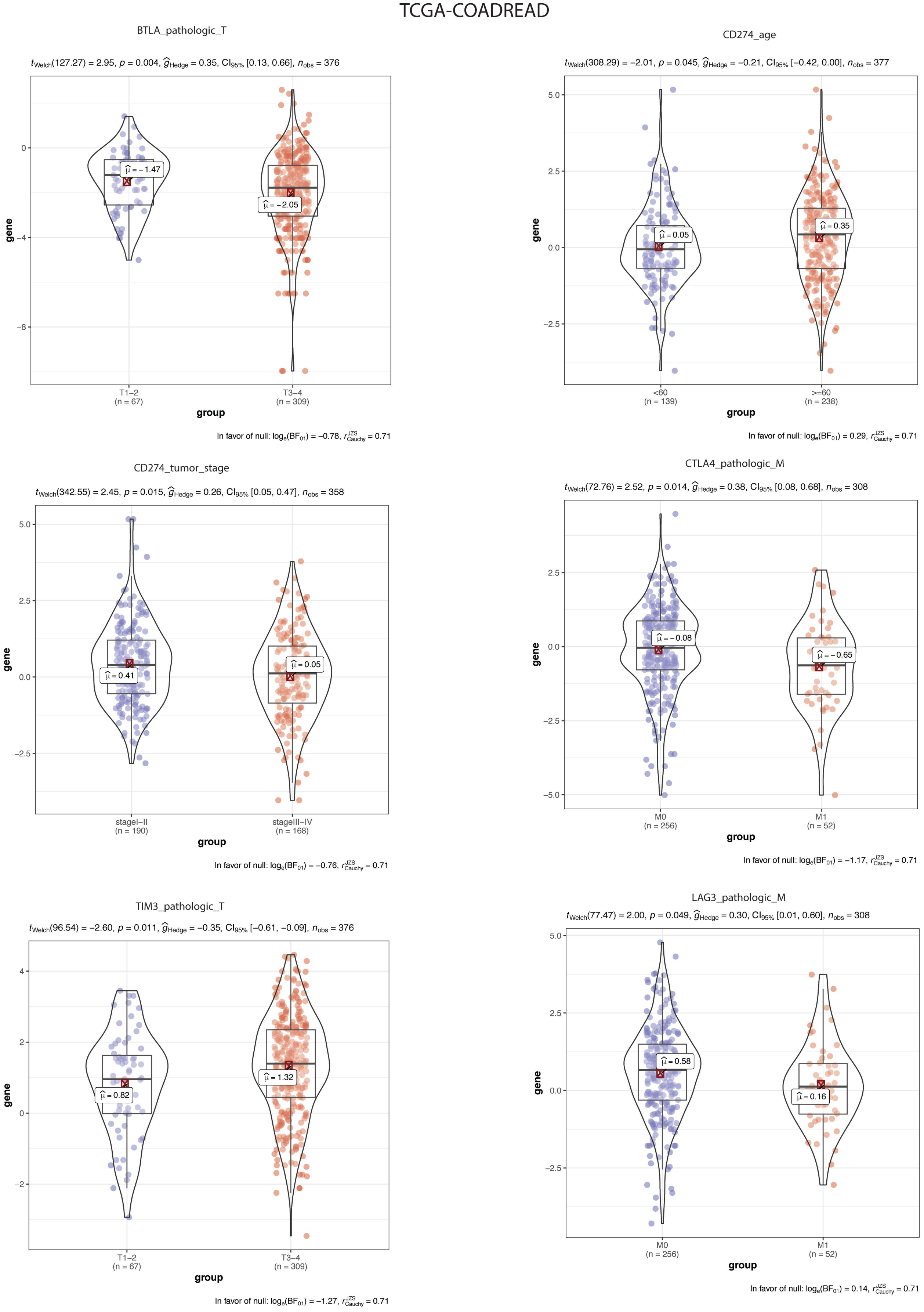
TCGA-COADREAD
BTLA_pathologic_T
$t_{Welch}(127.27) = 2.95$, $p = 0.004$, $\widehat{g}_{Hedge} = 0.35$, $CI_{95\%}$ [0.13, 0.66], $n_{obs} = 376$
$\widehat{\mu} = -1.47$
$\widehat{\mu} = -2.05$
gene
0
−4
−8
T1−2
(n = 67)
T3−4
(n = 309)
group
In favor of null: $\log_e(BF_{01}) = -0.78$, $r^{JZS}_{Cauchy} = 0.71$
CD274_age
$t_{Welch}(308.29) = -2.01$, $p = 0.045$, $\widehat{g}_{Hedge} = -0.21$, $CI_{95\%}$ [−0.42, 0.00], $n_{obs} = 377$
$\widehat{\mu} = 0.05$
$\widehat{\mu} = 0.35$
gene
5.0
2.5
0.0
−2.5
<60
(n = 139)
>=60
(n = 238)
group
In favor of null: $\log_e(BF_{01}) = 0.29$, $r^{JZS}_{Cauchy} = 0.71$
CD274_tumor_stage
$t_{Welch}(342.55) = 2.45$, $p = 0.015$, $\widehat{g}_{Hedge} = 0.26$, $CI_{95\%}$ [0.05, 0.47], $n_{obs} = 358$
$\widehat{\mu} = 0.41$
$\widehat{\mu} = 0.05$
gene
5.0
2.5
0.0
−2.5
stageI−II
(n = 190)
stageIII−IV
(n = 168)
group
In favor of null: $\log_e(BF_{01}) = -0.76$, $r^{JZS}_{Cauchy} = 0.71$
CTLA4_pathologic_M
$t_{Welch}(72.76) = 2.52$, $p = 0.014$, $\widehat{g}_{Hedge} = 0.38$, $CI_{95\%}$ [0.08, 0.68], $n_{obs} = 308$
$\widehat{\mu} = -0.08$
$\widehat{\mu} = -0.65$
gene
2.5
0.0
−2.5
−5.0
M0
(n = 256)
M1
(n = 52)
group
In favor of null: $\log_e(BF_{01}) = -1.17$, $r^{JZS}_{Cauchy} = 0.71$
TIM3_pathologic_T
$t_{Welch}(96.54) = -2.60$, $p = 0.011$, $\widehat{g}_{Hedge} = -0.35$, $CI_{95\%}$ [−0.61, −0.09], $n_{obs} = 376$
$\widehat{\mu} = 0.82$
$\widehat{\mu} = 1.32$
gene
4
2
0
−2
T1−2
(n = 67)
T3−4
(n = 309)
group
In favor of null: $\log_e(BF_{01}) = -1.27$, $r^{JZS}_{Cauchy} = 0.71$
LAG3_pathologic_M
$t_{Welch}(77.47) = 2.00$, $p = 0.049$, $\widehat{g}_{Hedge} = 0.30$, $CI_{95\%}$ [0.01, 0.60], $n_{obs} = 308$
$\widehat{\mu} = 0.58$
$\widehat{\mu} = 0.16$
gene
5.0
2.5
0.0
−2.5
M0
(n = 256)
M1
(n = 52)
group
In favor of null: $\log_e(BF_{01}) = 0.14$, $r^{JZS}_{Cauchy} = 0.71$

LAG3_pathologic_N

$F_{\text{Welch}}(2,162.88) = 3.16$, $p = 0.045$, $\widehat{\omega_p^2} = 0.01$, $\text{CI}_{95\%}$ $[-0.01, 0.04]$, $n_{\text{obs}} = 374$

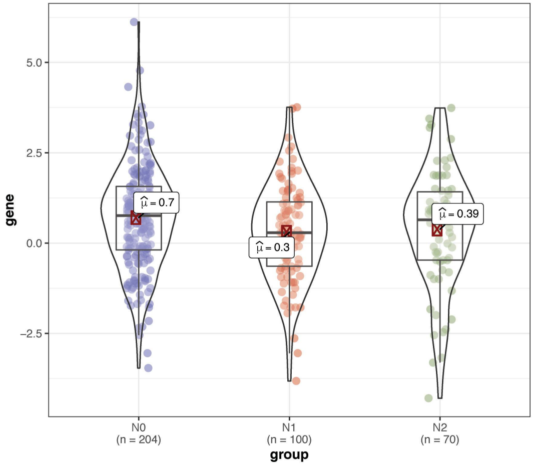

In favor of null: $\log_e(\text{BF}_{01}) = 1.14$, $r^{\text{JZS}}_{\text{Cauchy}} = 0.71$

LAG3_site_of_resection

$F_{\text{Welch}}(2,192.24) = 5.84$, $p = 0.003$, $\widehat{\omega_p^2} = 0.03$, $\text{CI}_{95\%}$ $[0.00, 0.08]$, $n_{\text{obs}} = 313$

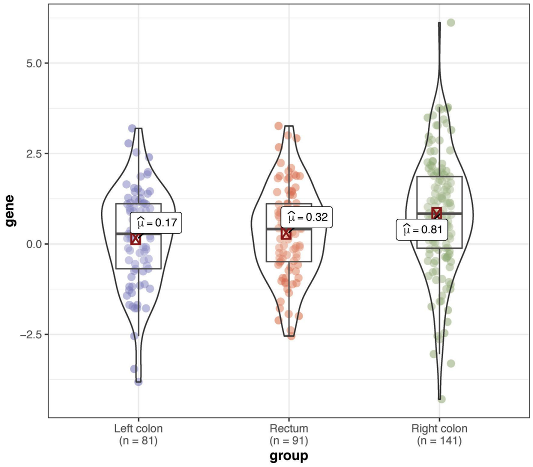

In favor of null: $\log_e(\text{BF}_{01}) = -1.84$, $r^{\text{JZS}}_{\text{Cauchy}} = 0.71$

LAG3_tumor_stage

$t_{\text{Welch}}(352.04) = 2.55$, $p = 0.011$, $\widehat{g}_{\text{Hedge}} = 0.27$, $\text{CI}_{95\%}$ $[0.06, 0.48]$, $n_{\text{obs}} = 358$

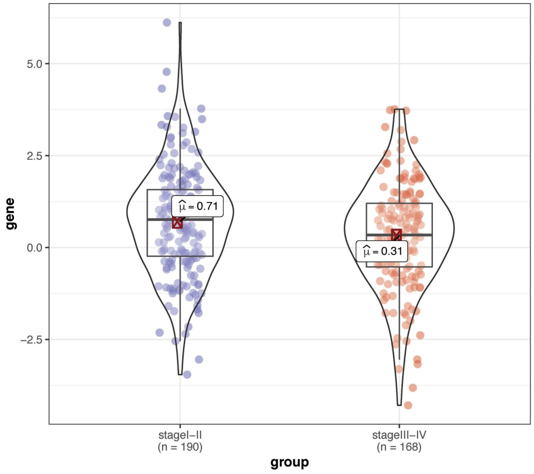

In favor of null: $\log_e(\text{BF}_{01}) = -0.96$, $r^{\text{JZS}}_{\text{Cauchy}} = 0.71$

PDCD1_site_of_resection

$F_{\text{Welch}}(2,183.31) = 5.74$, $p = 0.004$, $\widehat{\omega_p^2} = 0.03$, $\text{CI}_{95\%}$ $[0.00, 0.08]$, $n_{\text{obs}} = 313$

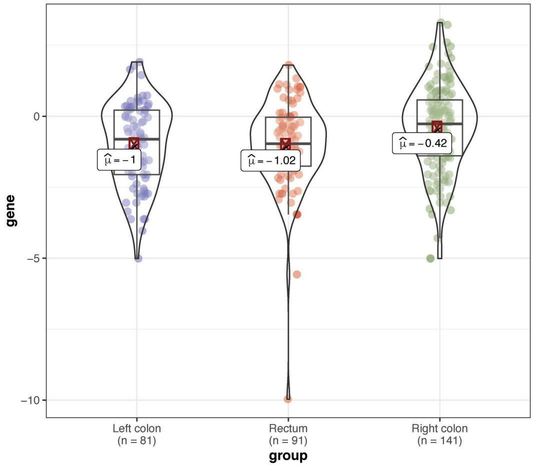

In favor of null: $\log_e(\text{BF}_{01}) = -1.48$, $r^{\text{JZS}}_{\text{Cauchy}} = 0.71$

SIRPA_age

$t_{\text{Welch}}(309.47) = 2.55$, $p = 0.011$, $\widehat{g}_{\text{Hedge}} = 0.27$, $\text{CI}_{95\%}$ $[0.06, 0.48]$, $n_{\text{obs}} = 377$

SIRPA_pathologic_N

$F_{\text{Welch}}(2,170.06) = 3.10$, $p = 0.048$, $\widehat{\omega_p^2} = 0.01$, $\text{CI}_{95\%}$ $[-0.01, 0.04]$, $n_{\text{obs}} = 374$

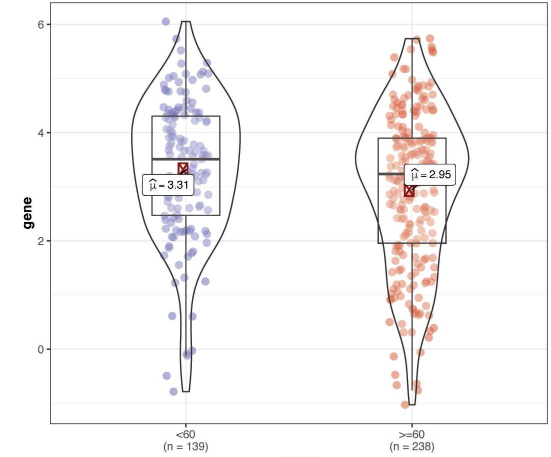

In favor of null: $\log_e(BF_{01}) = -0.84$, $r^{JZS}_{Cauchy} = 0.71$

SIRPA_pathologic_T

$t_{Welch}(89.23) = -2.35$, $p = 0.021$, $\widehat{g}_{Hedge} = -0.33$, $CI_{95\%}$ [−0.58, −0.05], $n_{obs} = 376$

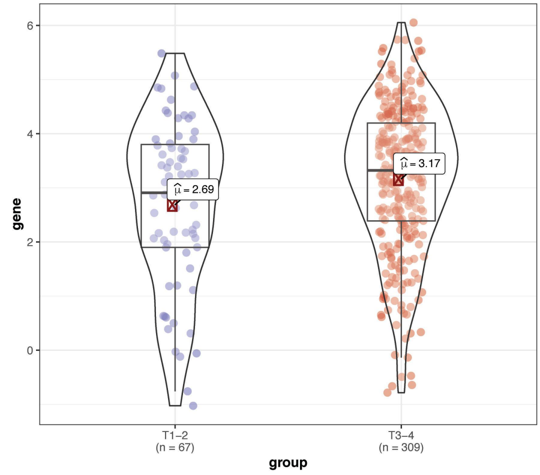

In favor of null: $\log_e(BF_{01}) = -1.14$, $r^{JZS}_{Cauchy} = 0.71$

TIGIT_pathologic_M

$t_{Welch}(75.96) = 2.51$, $p = 0.014$, $\widehat{g}_{Hedge} = 0.37$, $CI_{95\%}$ [0.08, 0.68], $n_{obs} = 308$

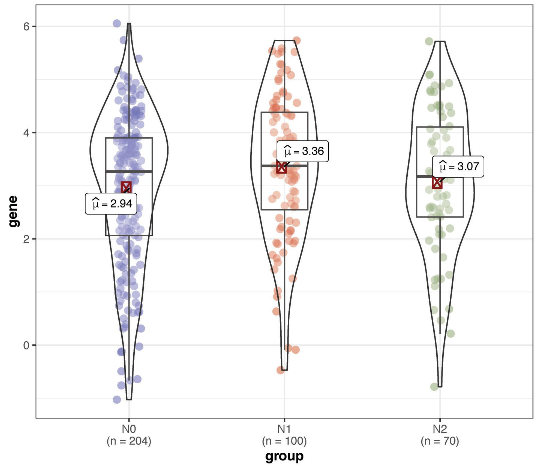

In favor of null: $\log_e(BF_{01}) = 1.13$, $r^{JZS}_{Cauchy} = 0.71$

SIRPA_tumor_stage

$t_{Welch}(355.31) = -2.15$, $p = 0.032$, $\widehat{g}_{Hedge} = -0.23$, $CI_{95\%}$ [−0.44, −0.02], $n_{obs} = 358$

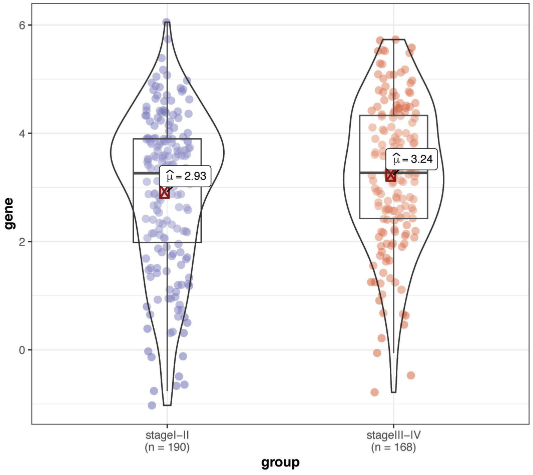

In favor of null: $\log_e(BF_{01}) = -0.05$, $r^{JZS}_{Cauchy} = 0.71$

TNFRSF4_age

$t_{Welch}(319.52) = 2.54$, $p = 0.011$, $\widehat{g}_{Hedge} = 0.27$, $CI_{95\%}$ [0.06, 0.48], $n_{obs} = 377$

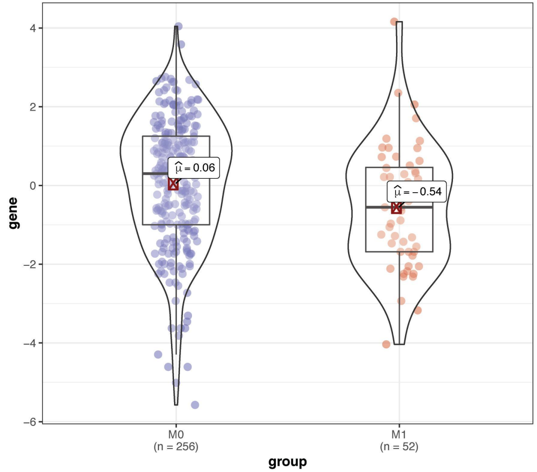

In favor of null: $\log_e(BF_{01}) = -0.90$, $r^{JZS}_{Cauchy} = 0.71$

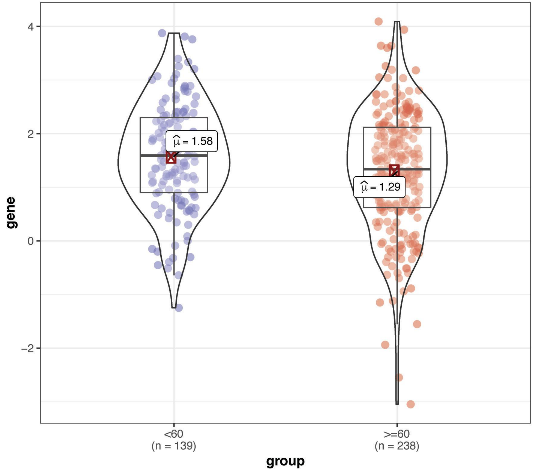

In favor of null: $\log_e(BF_{01}) = -0.75$, $r^{JZS}_{Cauchy} = 0.71$

TNFRSF4_pathologic_T

$t_{Welch}(105.91) = -2.46$, $p = 0.015$, $\hat{g}_{Hedge} = -0.32$, $CI_{95\%}$ [−0.60, −0.07], $n_{obs} = 376$

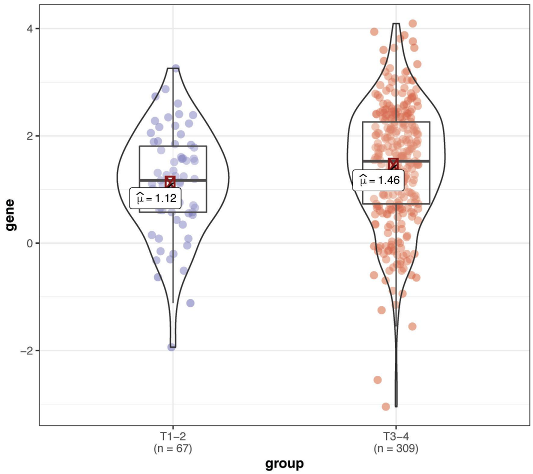

In favor of null: $\log_e(BF_{01}) = -0.51$, $r^{JZS}_{Cauchy} = 0.71$

VTCN1_pathologic_N

$F_{Welch}(2,169.93) = 5.53$, $p = 0.005$, $\widehat{\omega^2_p} = 0.02$, $CI_{95\%}$ [0.00, 0.06], $n_{obs} = 374$

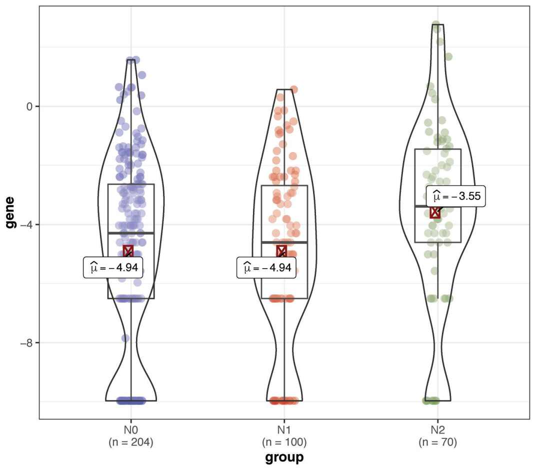

In favor of null: $\log_e(BF_{01}) = -1.09$, $r^{JZS}_{Cauchy} = 0.71$

VTCN1_site_of_resection

$F_{Welch}(2,177.81) = 5.33$, $p = 0.006$, $\widehat{\omega^2_p} = 0.03$, $CI_{95\%}$ [0.00, 0.07], $n_{obs} = 313$

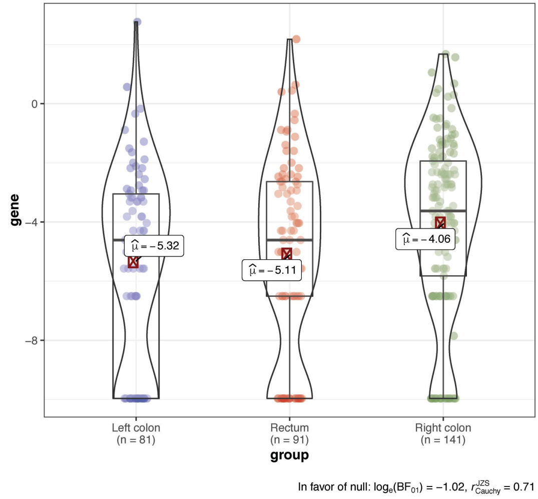

GSE103479

BTLA_Adjuvanttreatment

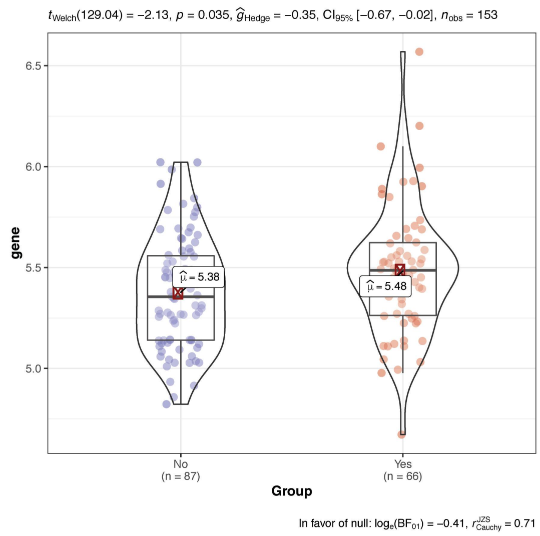

CD47_BRAF

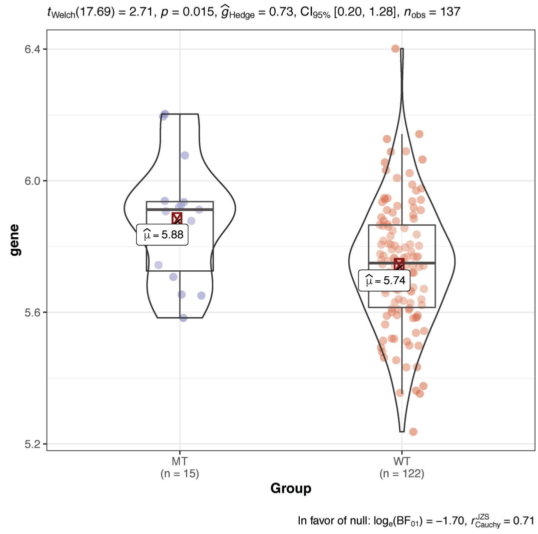

CD47_Cris.subgroup

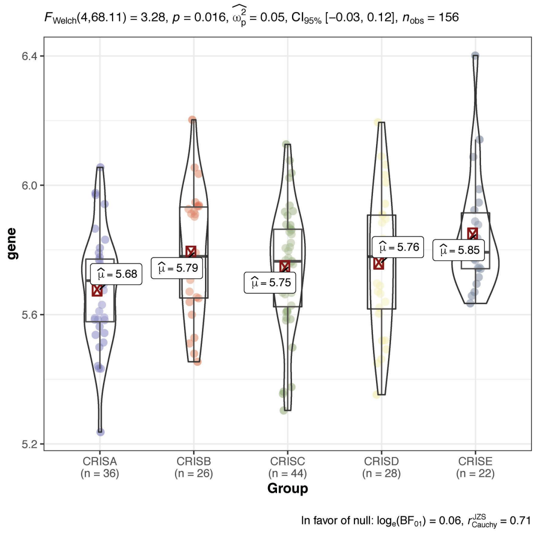

CD274_Cms.subgroup

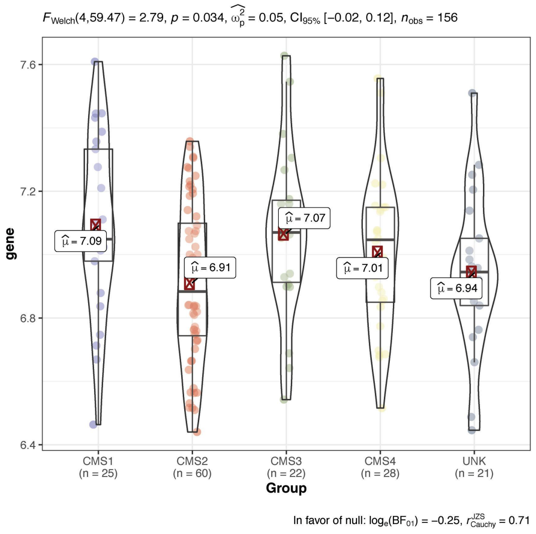

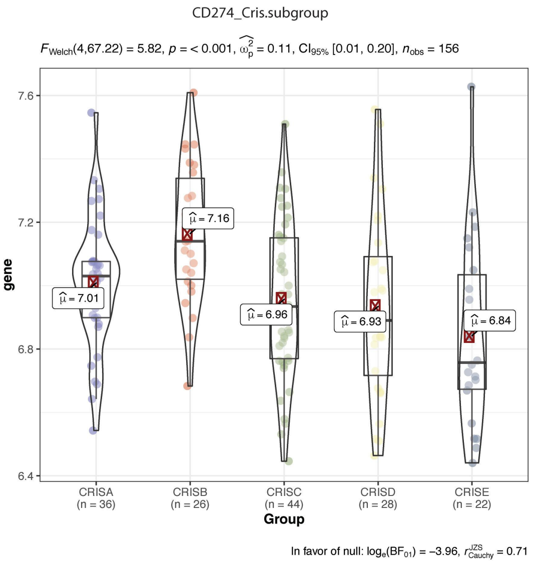
CD274_Cris.subgroup
$F_{\text{Welch}}(4,67.22) = 5.82$, $p = < 0.001$, $\widehat{\omega_p^2} = 0.11$, $\text{CI}_{95\%}$ [0.01, 0.20], $n_{\text{obs}} = 156$
gene
Group
CRISA (n = 36)
CRISB (n = 26)
CRISC (n = 44)
CRISD (n = 28)
CRISE (n = 22)
$\hat{\mu} = 7.01$
$\hat{\mu} = 7.16$
$\hat{\mu} = 6.96$
$\hat{\mu} = 6.93$
$\hat{\mu} = 6.84$
In favor of null: $\log_e(\text{BF}_{01}) = -3.96$, $r_{\text{Cauchy}}^{\text{JZS}} = 0.71$

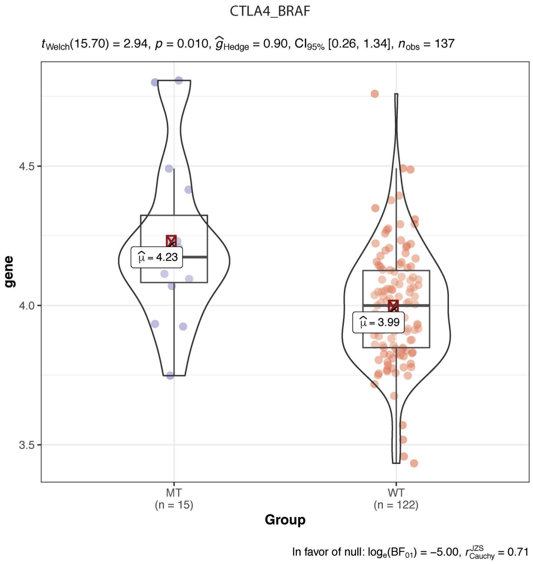
CTLA4_BRAF
$t_{\text{Welch}}(15.70) = 2.94$, $p = 0.010$, $\widehat{g}_{\text{Hedge}} = 0.90$, $\text{CI}_{95\%}$ [0.26, 1.34], $n_{\text{obs}} = 137$
gene
Group
MT (n = 15)
WT (n = 122)
$\hat{\mu} = 4.23$
$\hat{\mu} = 3.99$
In favor of null: $\log_e(\text{BF}_{01}) = -5.00$, $r_{\text{Cauchy}}^{\text{JZS}} = 0.71$

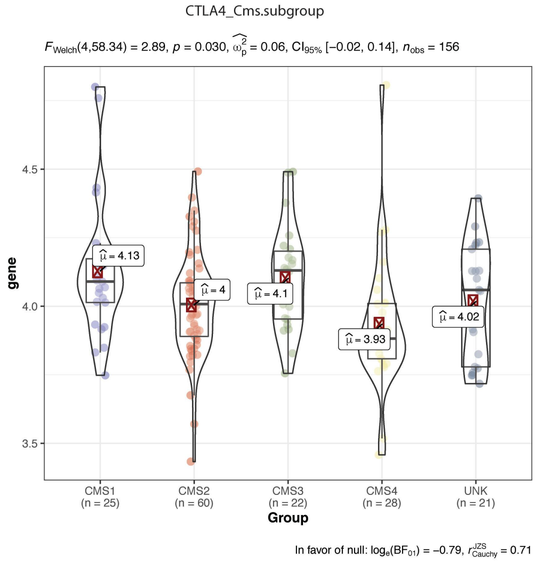
CTLA4_Cms.subgroup
$F_{\text{Welch}}(4,58.34) = 2.89$, $p = 0.030$, $\widehat{\omega_p^2} = 0.06$, $\text{CI}_{95\%}$ [−0.02, 0.14], $n_{\text{obs}} = 156$
gene
Group
CMS1 (n = 25)
CMS2 (n = 60)
CMS3 (n = 22)
CMS4 (n = 28)
UNK (n = 21)
$\hat{\mu} = 4.13$
$\hat{\mu} = 4$
$\hat{\mu} = 4.1$
$\hat{\mu} = 3.93$
$\hat{\mu} = 4.02$
In favor of null: $\log_e(\text{BF}_{01}) = -0.79$, $r_{\text{Cauchy}}^{\text{JZS}} = 0.71$

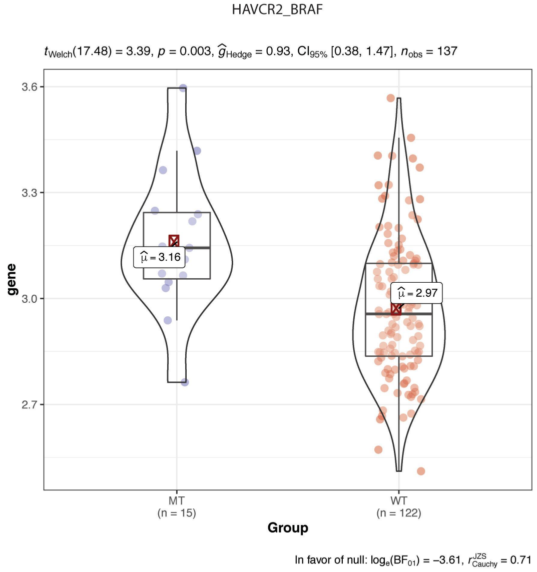
HAVCR2_BRAF
$t_{\text{Welch}}(17.48) = 3.39$, $p = 0.003$, $\widehat{g}_{\text{Hedge}} = 0.93$, $\text{CI}_{95\%}$ [0.38, 1.47], $n_{\text{obs}} = 137$
gene
Group
MT (n = 15)
WT (n = 122)
$\hat{\mu} = 3.16$
$\hat{\mu} = 2.97$
In favor of null: $\log_e(\text{BF}_{01}) = -3.61$, $r_{\text{Cauchy}}^{\text{JZS}} = 0.71$

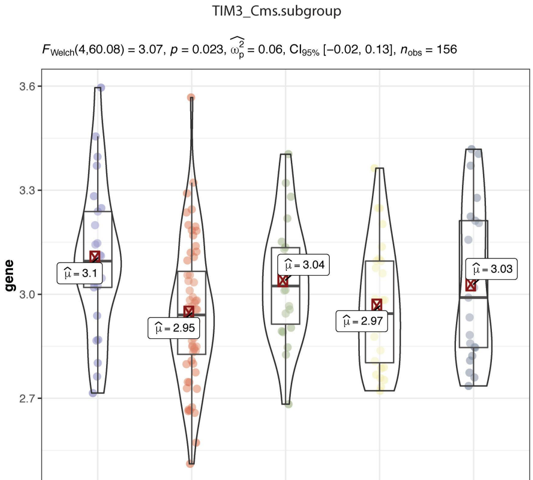
TIM3_Cms.subgroup
$F_{\text{Welch}}(4,60.08) = 3.07$, $p = 0.023$, $\widehat{\omega_p^2} = 0.06$, $\text{CI}_{95\%}$ [−0.02, 0.13], $n_{\text{obs}} = 156$
gene
$\hat{\mu} = 3.1$
$\hat{\mu} = 2.95$
$\hat{\mu} = 3.04$
$\hat{\mu} = 2.97$
$\hat{\mu} = 3.03$

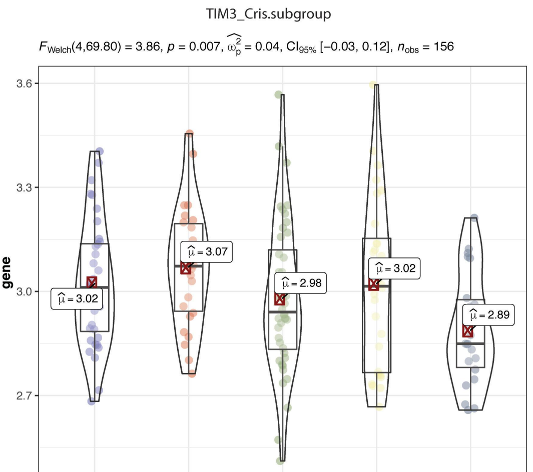
TIM3_Cris.subgroup
$F_{\text{Welch}}(4,69.80) = 3.86$, $p = 0.007$, $\widehat{\omega_p^2} = 0.04$, $\text{CI}_{95\%}$ [−0.03, 0.12], $n_{\text{obs}} = 156$
gene
$\hat{\mu} = 3.02$
$\hat{\mu} = 3.07$
$\hat{\mu} = 2.98$
$\hat{\mu} = 3.02$
$\hat{\mu} = 2.89$

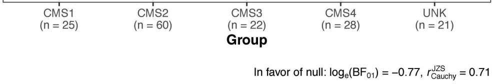

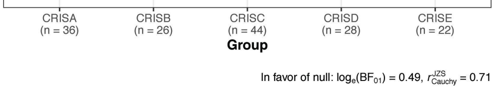

NT5E_BRAF

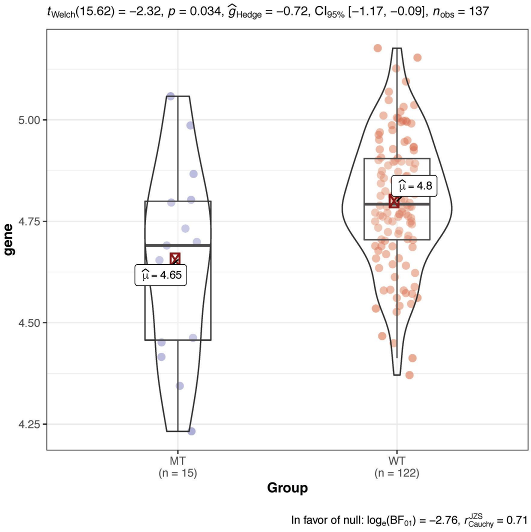

NT5E_Cms.subgroup

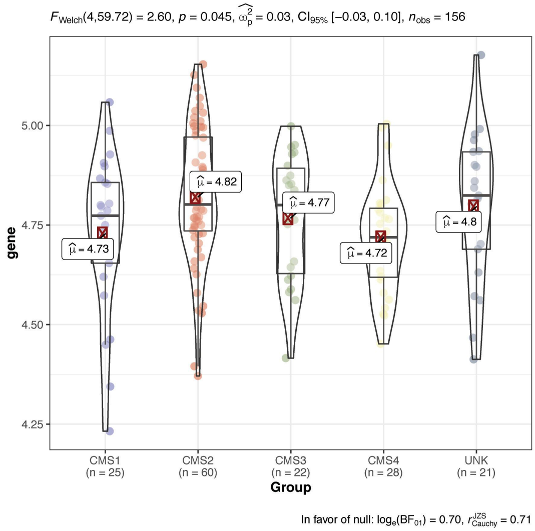

NT5E_Cris.subgroup

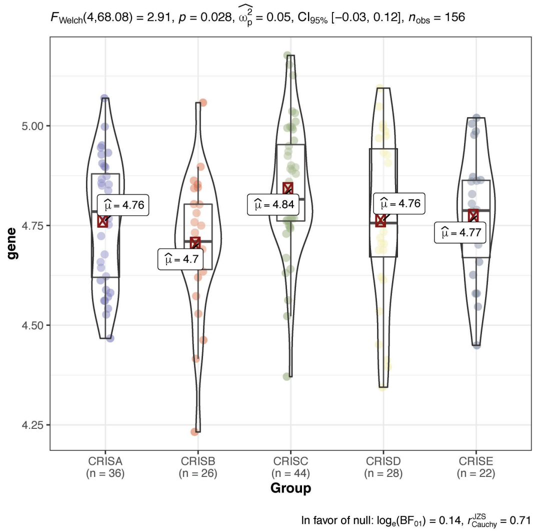

PDCD1_Lvi

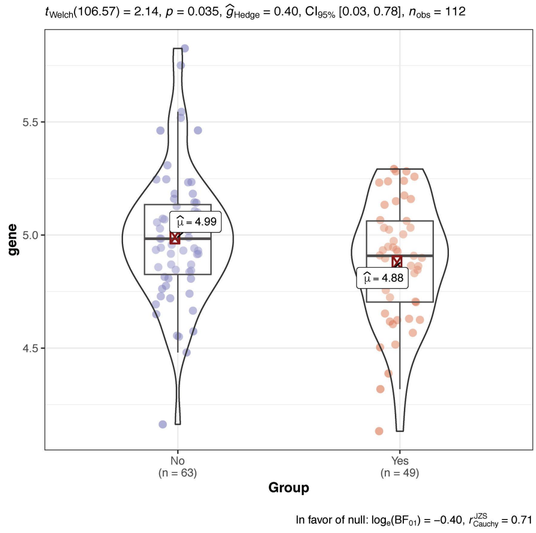

SIRPA_Adjuvanttreatment

$t_{\text{Welch}}(134.72) = 2.16$, $p = 0.033$, $\widehat{g}_{\text{Hedge}} = 0.35$, $\text{CI}_{95\%}$ [0.03, 0.67], $n_{\text{obs}} = 153$

SIRPA_Age

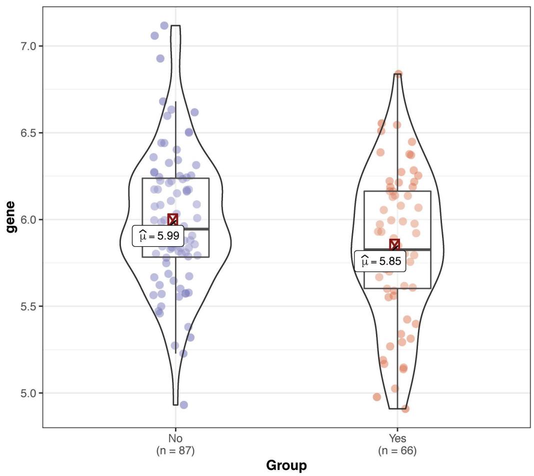

In favor of null: $\log_e(BF_{01}) = -0.42$, $r^{JZS}_{Cauchy} = 0.71$

$t_{Welch}(37.09) = -2.48$, $p = 0.018$, $\widehat{g}_{Hedge} = -0.53$, $CI_{95\%}$ [−0.93, −0.10], $n_{obs} = 156$

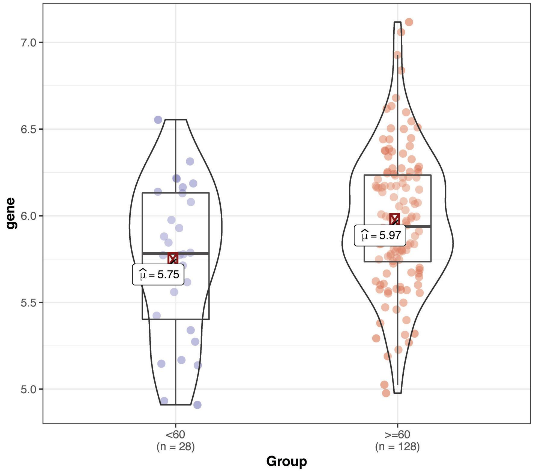

In favor of null: $\log_e(BF_{01}) = -1.57$, $r^{JZS}_{Cauchy} = 0.71$

SIRPA_Cms.subgroup

$F_{Welch}(4,60.53) = 3.71$, $p = 0.009$, $\widehat{\omega^2_p} = 0.06$, $CI_{95\%}$ [−0.02, 0.14], $n_{obs} = 156$

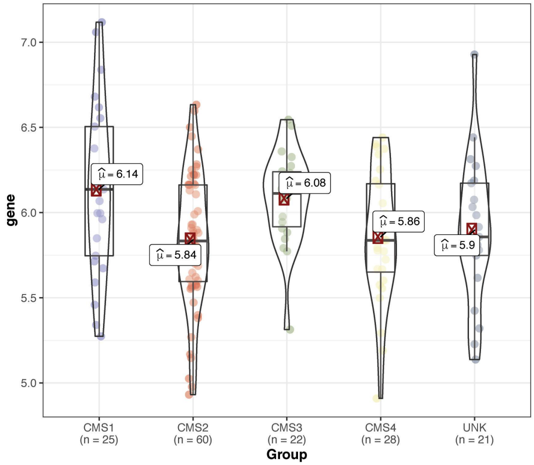

In favor of null: $\log_e(BF_{01}) = -0.92$, $r^{JZS}_{Cauchy} = 0.71$

SIRPA_Cris.subgroup

$F_{Welch}(4,65.81) = 4.77$, $p = 0.002$, $\widehat{\omega^2_p} = 0.08$, $CI_{95\%}$ [−0.01, 0.17], $n_{obs} = 156$

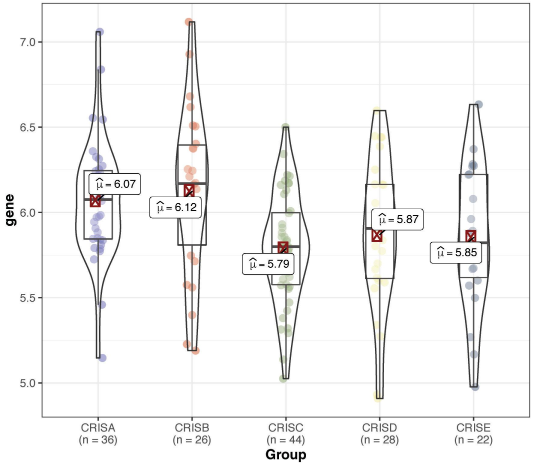

In favor of null: $\log_e(BF_{01}) = -2.55$, $r^{JZS}_{Cauchy} = 0.71$

SIRPA_Gender

$t_{Welch}(144.94) = 2.10$, $p = 0.038$, $\widehat{g}_{Hedge} = 0.34$, $CI_{95\%}$ [0.02, 0.65], $n_{obs} = 156$

SIRPA_P53

$t_{Welch}(129.67) = -1.97$, $p = 0.051$, $\widehat{g}_{Hedge} = -0.34$, $CI_{95\%}$ [−0.68, 0.00], $n_{obs} = 137$

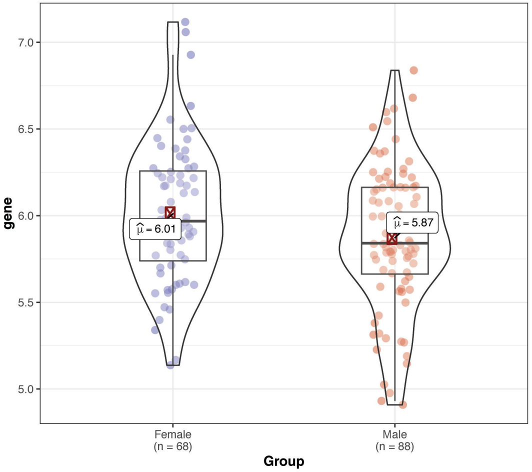

In favor of null: $\log_e(BF_{01}) = -0.25$, $r^{JZS}_{Cauchy} = 0.71$

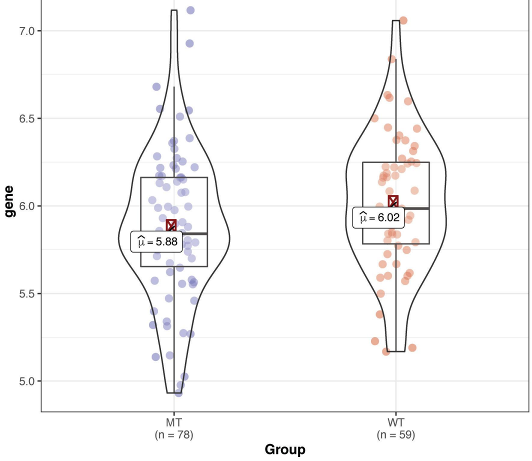

In favor of null: $\log_e(BF_{01}) = -0.02$, $r^{JZS}_{Cauchy} = 0.71$

SIRPA_T_stage

$F_{Welch}(2,16.73) = 3.91$, $p = 0.041$, $\widehat{\omega^2_p} = 0.02$, $CI_{95\%}$ [−0.01, 0.09], $n_{obs} = 156$

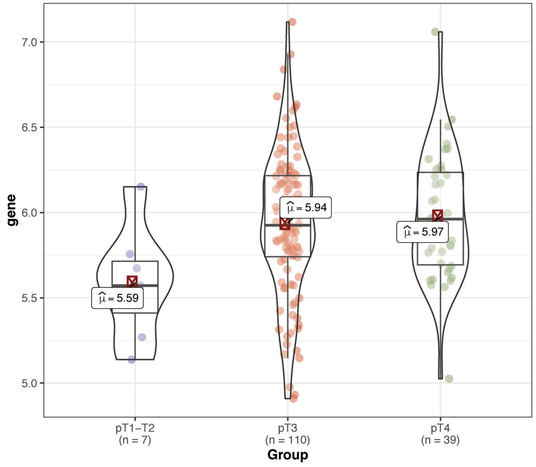

In favor of null: $\log_e(BF_{01}) = 0.75$, $r^{JZS}_{Cauchy} = 0.71$

TIGIT_BRAF

$t_{Welch}(15.65) = -4.01$, $p = 0.001$, $\widehat{g}_{Hedge} = -1.23$, $CI_{95\%}$ [−1.64, −0.54], $n_{obs} = 137$

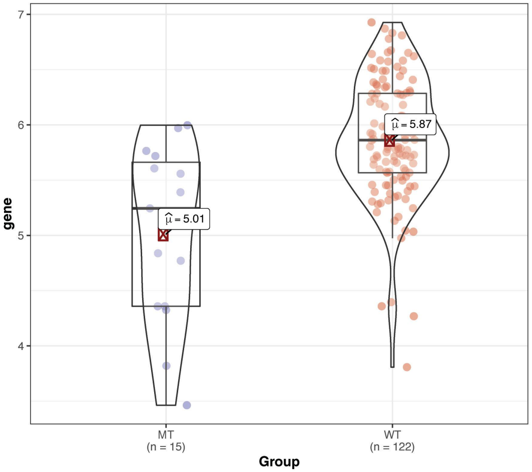

In favor of null: $\log_e(BF_{01}) = -10.39$, $r^{JZS}_{Cauchy} = 0.71$

TIGIT_Cms.subgroup

$F_{Welch}(4,56.15) = 6.48$, $p = < 0.001$, $\widehat{\omega^2_p} = 0.18$, $CI_{95\%}$ [0.06, 0.28], $n_{obs} = 156$

TIGIT_Cris.subgroup

$F_{Welch}(4,64.88) = 7.20$, $p = < 0.001$, $\widehat{\omega^2_p} = 0.13$, $CI_{95\%}$ [0.03, 0.23], $n_{obs} = 156$

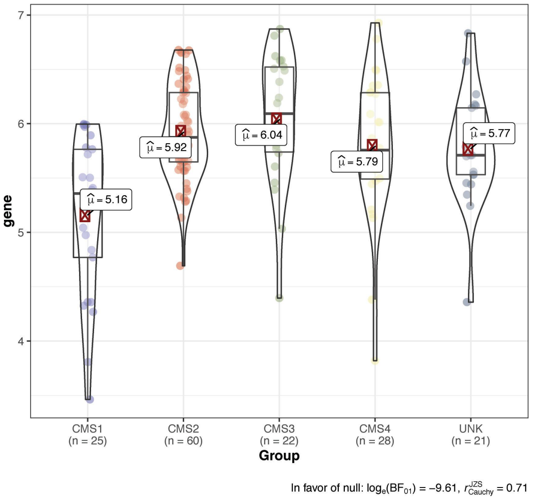
gene
μ̂ = 5.16
μ̂ = 5.92
μ̂ = 6.04
μ̂ = 5.79
μ̂ = 5.77
CMS1 (n = 25)
CMS2 (n = 60)
CMS3 (n = 22)
CMS4 (n = 28)
UNK (n = 21)
Group
In favor of null: $\log_e(BF_{01}) = -9.61$, $r^{JZS}_{Cauchy} = 0.71$

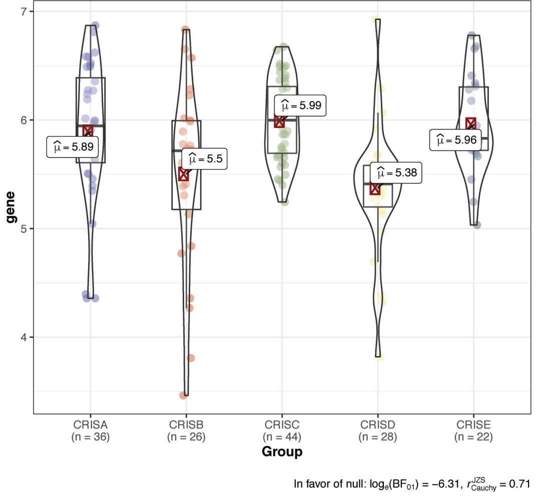
gene
μ̂ = 5.89
μ̂ = 5.5
μ̂ = 5.99
μ̂ = 5.38
μ̂ = 5.96
CRISA (n = 36)
CRISB (n = 26)
CRISC (n = 44)
CRISD (n = 28)
CRISE (n = 22)
Group
In favor of null: $\log_e(BF_{01}) = -6.31$, $r^{JZS}_{Cauchy} = 0.71$

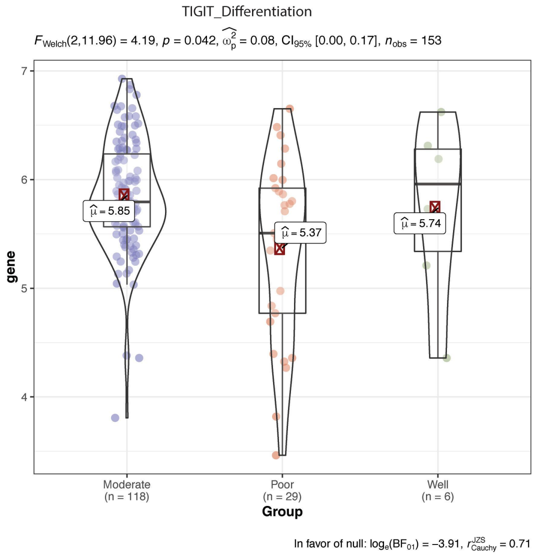
TIGIT_Differentiation
$F_{Welch}(2,11.96) = 4.19$, $p = 0.042$, $\widehat{\omega^2_p} = 0.08$, $CI_{95\%}$ [0.00, 0.17], $n_{obs} = 153$
gene
μ̂ = 5.85
μ̂ = 5.37
μ̂ = 5.74
Moderate (n = 118)
Poor (n = 29)
Well (n = 6)
Group
In favor of null: $\log_e(BF_{01}) = -3.91$, $r^{JZS}_{Cauchy} = 0.71$

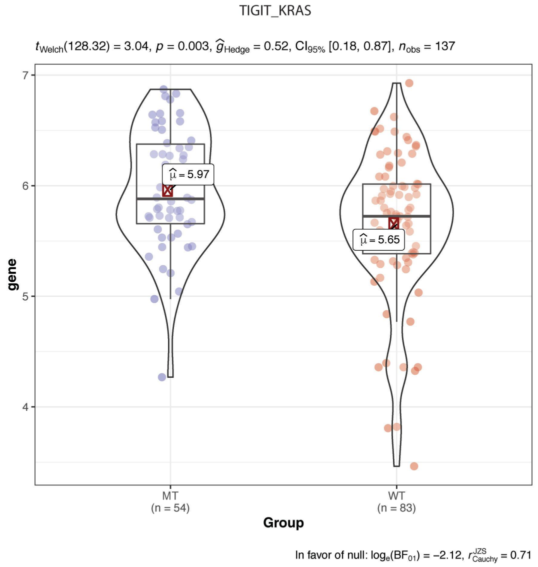
TIGIT_KRAS
$t_{Welch}(128.32) = 3.04$, $p = 0.003$, $\widehat{g}_{Hedge} = 0.52$, $CI_{95\%}$ [0.18, 0.87], $n_{obs} = 137$
gene
μ̂ = 5.97
μ̂ = 5.65
MT (n = 54)
WT (n = 83)
Group
In favor of null: $\log_e(BF_{01}) = -2.12$, $r^{JZS}_{Cauchy} = 0.71$

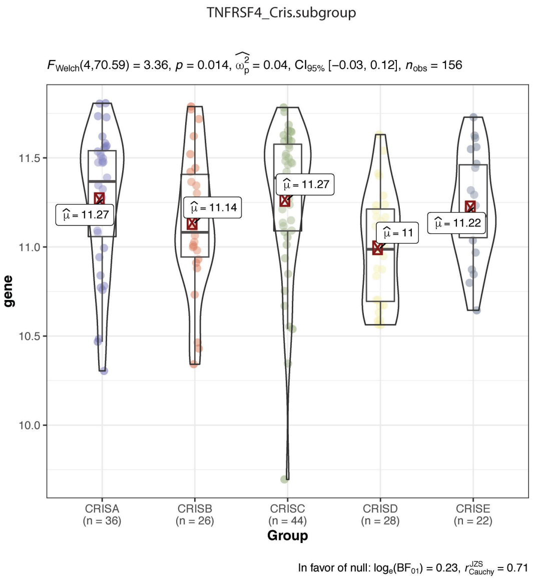
TNFRSF4_Cris.subgroup
$F_{Welch}(4,70.59) = 3.36$, $p = 0.014$, $\widehat{\omega^2_p} = 0.04$, $CI_{95\%}$ [−0.03, 0.12], $n_{obs} = 156$
gene
μ̂ = 11.27
μ̂ = 11.14
μ̂ = 11.27
μ̂ = 11
μ̂ = 11.22
CRISA (n = 36)
CRISB (n = 26)
CRISC (n = 44)
CRISD (n = 28)
CRISE (n = 22)
Group
In favor of null: $\log_e(BF_{01}) = 0.23$, $r^{JZS}_{Cauchy} = 0.71$

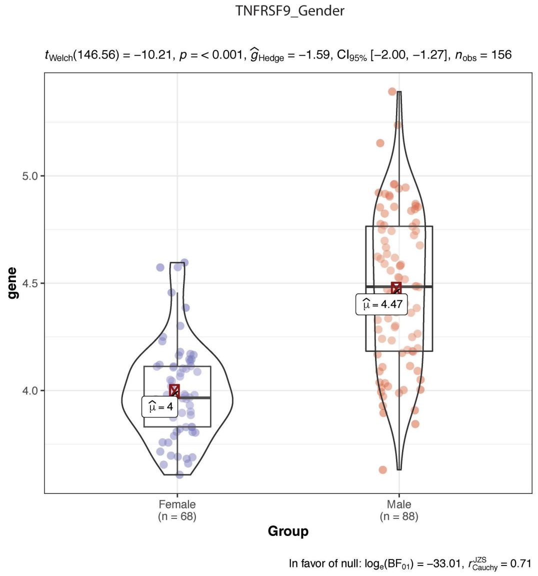
TNFRSF9_Gender
$t_{Welch}(146.56) = -10.21$, $p = < 0.001$, $\widehat{g}_{Hedge} = -1.59$, $CI_{95\%}$ [−2.00, −1.27], $n_{obs} = 156$
gene
μ̂ = 4
μ̂ = 4.47
Female (n = 68)
Male (n = 88)
Group
In favor of null: $\log_e(BF_{01}) = -33.01$, $r^{JZS}_{Cauchy} = 0.71$

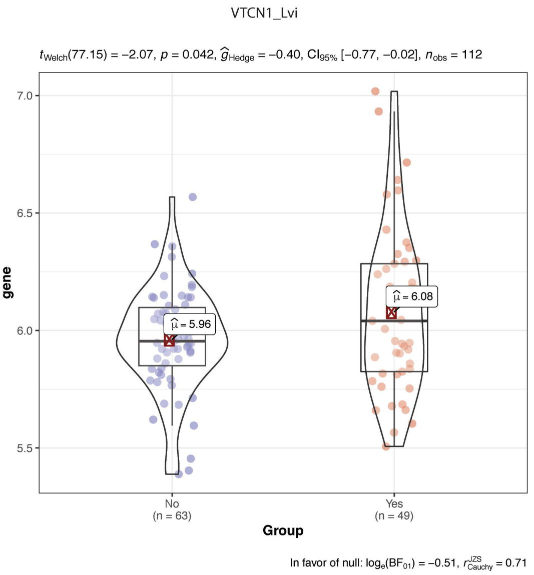

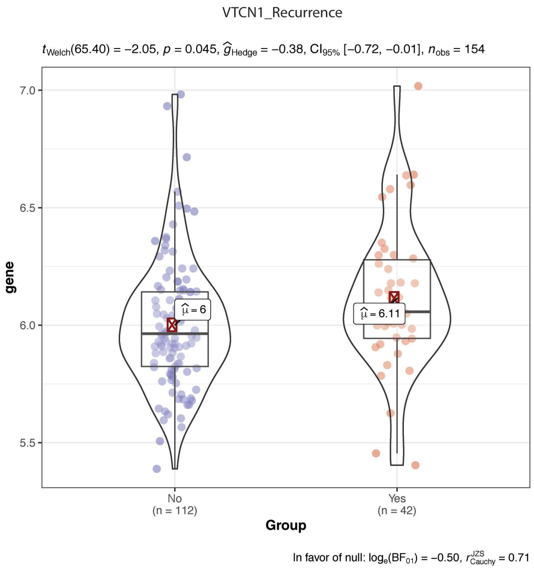

GSE72970

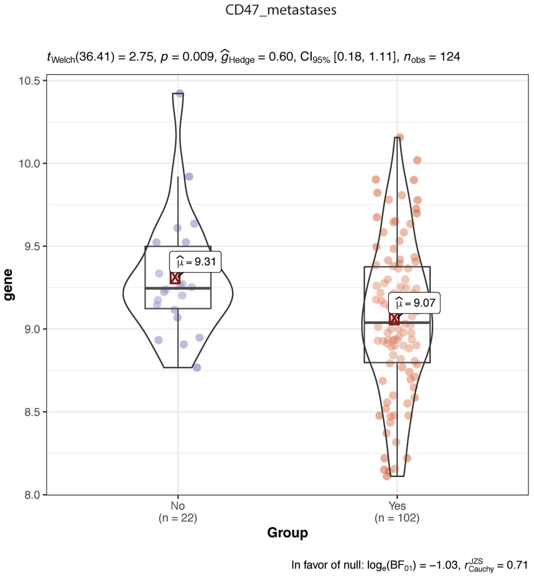

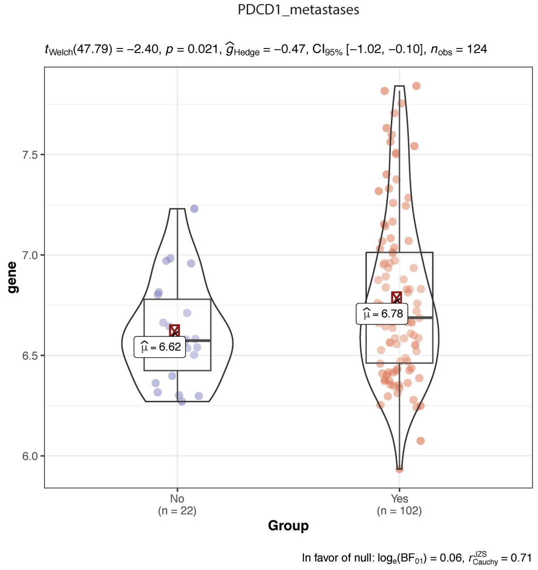

Supplementary figure 2. Distribution of immune checkpoints under clinical features. Expression of BTLA in TCGA-COADREAD under feature pathologic_T, expression of CD274 in TCGA-COADREAD under feature age, expression of CD274 in TCGA-COADREAD under feature tumor_stage, expression of CTLA4 in TCGA-COADREAD under feature pathologic_M, expression of TIM3 in TCGA-COADREAD under feature pathologic_T, expression of LAG3 in TCGA-COADREAD under feature pathologic_M, expression of LAG3 in TCGA-COADREAD under feature pathologic_N, expression of LAG3 in TCGA-COADREAD under feature site_of_resection, expression of LAG3 in TCGA-COADREAD under feature tumor_stage, expression of PDCD1 in TCGA-COADREAD under feature site_of_resection, expression of SIRPA in TCGA-COADREAD under feature age, expression of SIRPA in TCGA-COADREAD under feature pathologic_N, expression of SIRPA in TCGA-COADREAD under feature pathologic_T, expression of SIRPA in TCGA-COADREAD under feature tumor_stage, expression of TIGIT in TCGA-COADREAD under feature pathologic_M, expression of TNFRSF4 in TCGA-COADREAD under feature age, expression of TNFRSF4 in TCGA-COADREAD under feature pathologic_T, expression of VTCN1 in TCGA-COADREAD under feature pathologic_N, expression of VTCN1 in TCGA-COADREAD under feature site_of_resection, expression of BTLA in GSE103479 under feature Adjuvanttreatment, expression of CD47 in GSE103479 under feature BRAF, expression of CD47 in GSE103479 under feature Cris_subgroup, expression of CD274 in GSE103479 under feature Cms_subgroup, expression of CD274 in GSE103479 under feature Cris_subgroup, expression of CTLA4 in GSE103479 under feature BRAF, expression of CTLA4 in GSE103479 under feature Cms_subgroup, expression of TIM3 in GSE103479 under feature BRAF, expression of TIM3 in GSE103479 under feature Cms_subgroup, expression of TIM3 in GSE103479 under feature Cris_subgroup, expression of CD73 in GSE103479 under feature BRAF, expression of CD73 in GSE103479 under feature Cms_sub-

group, expression of CD73 in GSE103479 under feature Cris_subgroup, expression of PDCD1 in GSE103479 under feature Lvi, expression of SIRPA in GSE103479 under feature Adjuvanttreatment, expression of SIRPA in GSE103479 under feature Age, expression of SIRPA in GSE103479 under feature Cms_subgroup, expression of SIRPA in GSE103479 under feature Cris_subgroup, expression of SIRPA in GSE103479 under feature Gender, expression of SIRPA in GSE103479 under feature P53, expression of SIRPA in GSE103479 under feature T_stage, expression of TIGIT in GSE103479 under feature BRAF, expression of TIGIT in GSE103479 under feature Cms_subgroup, expression of TIGIT in GSE103479 under feature Cris_subgroup, expression of TIGIT in GSE103479 under feature Differentiation, expression of TIGIT in GSE103479 under feature KRAS, expression of TNFRSF4 in GSE103479 under feature Cris_subgroup, expression of TNFRSF9 in GSE103479 under feature Gender, expression of VTCN1 in GSE103479 under feature Lvi, expression of VTCN1 in GSE103479 under feature Recurrence, expression of CD47 in GSE72970 under feature metastases, expression of PDCD1 in GSE72970 under feature metastases (order from top left to bottom right)
